# Trityl-Containing Alcohols—An Efficient Chirality Transmission Process from Inductor to the Stereodynamic Propeller and their Solid-State Structural Diversity

**DOI:** 10.3390/molecules25030707

**Published:** 2020-02-06

**Authors:** Sylwia Górczyńska, Aleksandra Brzdonkiewicz, Maciej Jelecki, Agnieszka Czapik, Bartosz Stasiak, Marcin Kwit

**Affiliations:** 1 Department of Chemistry Adam Mickiewicz University, Uniwersytetu Poznańskiego 8, 61614 Poznań, Poland; sylwia.gorczynska@amu.edu.pl (S.G.); ola.brzdonkiewicz@gmail.com (A.B.); maciej.jelecki@gmail.com (M.J.); agnieszka.czapik@amu.edu.pl (A.C.); bartosz.stasiak@amu.edu.pl (B.S.); 2 Centre for Advanced Technologies Adam Mickiewicz University, Uniwersytetu Poznańskiego 10, 61614 Poznań, Poland

**Keywords:** chirality transmission, induced circular dichroism, stereoselective synthesis, trityl

## Abstract

The cascade process of a dynamic chirality transmission from the permanent chirality center to the stereodynamic triphenylmethyl group has been studied for series of optically active trityl derivatives. The structural analysis, carried out with the use of complementary methods, enabled us to determine the mechanism of chirality transfer. The process of chirality transmission involves a set of weak but complementary electrostatic interactions. The induction of helicity in a trityl propeller is revealed by rising non-zero cotton effects in the area of trityl UV-absorption. The presence of an additional stereogenic center in close proximity to the trityl-containing stereogenic center significantly affects the sign and, to a lesser extent, magnitude of the respective cotton effects. Despite the bulkiness of the trityl, in the crystalline phase, the molecules under study strictly fill the space. In the crystal, molecules form aggregates stabilized by OH•••O hydrogen bonds. However, the presence of two trityl groups precludes formation of OH•••O hydrogen bonding. Additionally, the trityl group seems to be responsible for the formation of the solid solutions by e.g., racemates of *trans*- and *cis*-2-tritylcyclohexanol. Therefore, the trityl group acts as a supramolecular protective group, which in turn can be used in the crystal engineering.

## 1. Introduction

The triphenylmethyl group (trityl, Tr) and its congeners are frequently used in synthetic organic chemistry for protecting alcohols, thiols, and amines [1]. Steric requirements make trityl particularly useful in carbohydrate chemistry, where a selective protection of primary alcohol(s) functionality is needed.

Very recently, the trityl-containing compounds have attracted attention in medicinal and, especially, in material chemistry [2]. *N*-Tritylated amino acids and the other molecules containing a trityl fragment, have been tested as promising therapeutic agents against many civilization diseases. The triarylmethane motifs are often found in the structure of functional materials, probes, and dyes. Akazome has proven that *N*-tritylated amino acids have the ability to form inclusion compounds and therefore an aptitude for enantiodiscrimination [3]. The steric properties of the trityl moiety have been recognized as responsible for precluding the formation of intermolecular hydrogen bonds between secondary chiral triphenylacetic acid amides and between secondary chiral ureas and thioureas [4,5].

The structural and electronic properties of the trityl moiety offer the possibility to use trityl-containing compounds in catalysis and in stereoselective synthesis. For example, the presence of trityl in cinchona alkaloid-based catalysts reverses the diastereoselectivity of asymmetric cycloadditions between aldehydes and enolizable anhydrides with respect to that usually observed. The stability of trityl cations allowed for their efficient use as Lewis acids in catalytic versions of Diels-Alder and aldol reactions [6,7,8,9].

Apart from usability in synthesis, the trityl, and related groups of the general Ar_3_X type, represents a unique example of a molecular fragment whose structural dynamics and mode of action resemble that of the macroscopic propeller. The stereochemistry of such molecular propellers has been extensively explored in the past, independently, by Mislow and Iwamura [10,11]. In the parent triphenylmethane (TrH) and its derivatives of the TrY type (where Y is a spherical substituent), the twisted phenyl rings connected to the central sp^3^-hybridized carbon atom adopt a chiral, *C*_3_–symmetrical conformation, characterized by ring twists of either *P* or *M* helicity. However, due to the low enantiomerization barrier, triphenylmethane and its TrY derivatives exist as non-separable dynamic racemates, hence no optical activity is observed in the region of UV absorption of the trityl chromophore.

On the other hand, the recently demonstrated ability of trityl to adapt to disturbances in the local symmetry of the chromophore’s chiral environment, has led to new applications of molecular propellers in dynamic stereochemistry [12]. The observed chirality transfer from the permanent chirality element to the stereodynamic probe has been observed by us and others for *O*-trityl alcohols, *N*-trityl amines, sulfides and selenides, triphenylacetic acid derivatives, and related compounds [13,14,15,16,17,18,19,20]. The phenomenon is revealed by the appearance of non-zero cotton effects (CEs) in electronic circular dichroism (ECD) spectra and in the region of the trityl UV absorption (usually between 220 nm and 180 nm). However, despite some similarities in the ECD spectra patterns, the mechanism of the chirality transmission is rather specific for a given class of compounds. For the prototypical example of *O*-trityl alcohols, the transfer of structural information proceeds through “a bevel gear” mechanism, which assumes direct steric interaction between the permanently chiral part of the system (the inductor) and the stereodynamic trityl unit (the reporter part). The direct gearing between trityl and inductor fragment is possible by the bent structure of the whole molecule. In all other cases, the chirality transfer is driven by more subtle (mostly electrostatic) interactions. As expected, the structural information transfer is more efficient for inductors characterized by high structural diversity, i.e., the substituents flanking the stereogenic center significantly differ in size and/or electronic properties.

In general, the chiroptical spectroscopy, mainly electronic and vibrational circular dichroism spectroscopy (ECD and VCD, respectively) is the method of choice for investigating structure and structural dynamics of chiral molecules in solution. Among the chiroptical methods, ECD spectroscopy, supported by theoretical DFT calculations, seems to be the most suitable for testing the dynamic chirality induction process in the trityl chromophore [21]. This combination of experimental and theoretical approaches enables an accurate perception of the structure of the molecular entity and the mechanism of the optical activity induction. Due to the wider availability of commercial spectrometers, VCD spectroscopy emerges now as a complementary source of structural information for compounds lacking the appropriate UV-Vis active chromophore.

Very recently, Monde, Abbate, Polavarapu, and others expanded the exciton chirality concept, fundamental for ECD spectroscopy, to vibrational circular dichroism (the vibrational circular dichroism exciton chirality (VCDEC) rule) [22,23]. In brief, the interactions through space between electronic transition moments associated with particular stretching vibrations for a given chromophoric system (mainly C=O, C=C and C=N) may lead to bisignate VCD couplets of the sequence of the cotton effects signs that reflect chirality in the interacting system [24].

In contrast, the X-ray crystallography provides the answer to the questions regarding the role of particular molecular fragments in the formation of the crystals [25]. As a result, these complementary methods allow for establishing the “supramolecular behavior” of the molecule and its individual fragments.

In this study, we put the emphasis on the chirality transfer in derivatives in which the chromophoric group is directly attached to the stereogenic center, the inductor being characterized by a low structural diversity and/or an additional chirality element being present in the close proximity. In the first case, the trityl group will constitute the inherent part of the molecule and it does not have to be artificially attached. In other cases, the direct chirality transfer from the stereogenic center to the propeller, could be disturbed by the presence of an additional stereogenic center within close proximity and further by the possibility of trityl–trityl interactions. The combination of two chiral molecules with a fragment of oxalic acid may enforce non-planar conformation of the linker. The interaction between electronic transition moments associated with C=O stretches will induce dynamic optical activity in vibrational circular dichroism and lead to bisignate exciton-type Cotton effects in the region 1700–1800 cm^−1^.

In this work, we will demonstrate the usefulness of the experimental/theoretical approach to determine the absolute stereochemistry of compounds being the products of asymmetric synthesis.

Also, to date, the influence of the small, permanently chiral part of the molecule on the conformational preference of trityls in the solid state has not been the subject of interests. Therefore, we will expand our study on the behavior of trityls to its association modes in the crystalline phase and to possible crystalline forms differing in densities and capable of hosting inclusions.

## 2. Results and Discussion

### 2.1. Synthesis

**Scheme 1:**
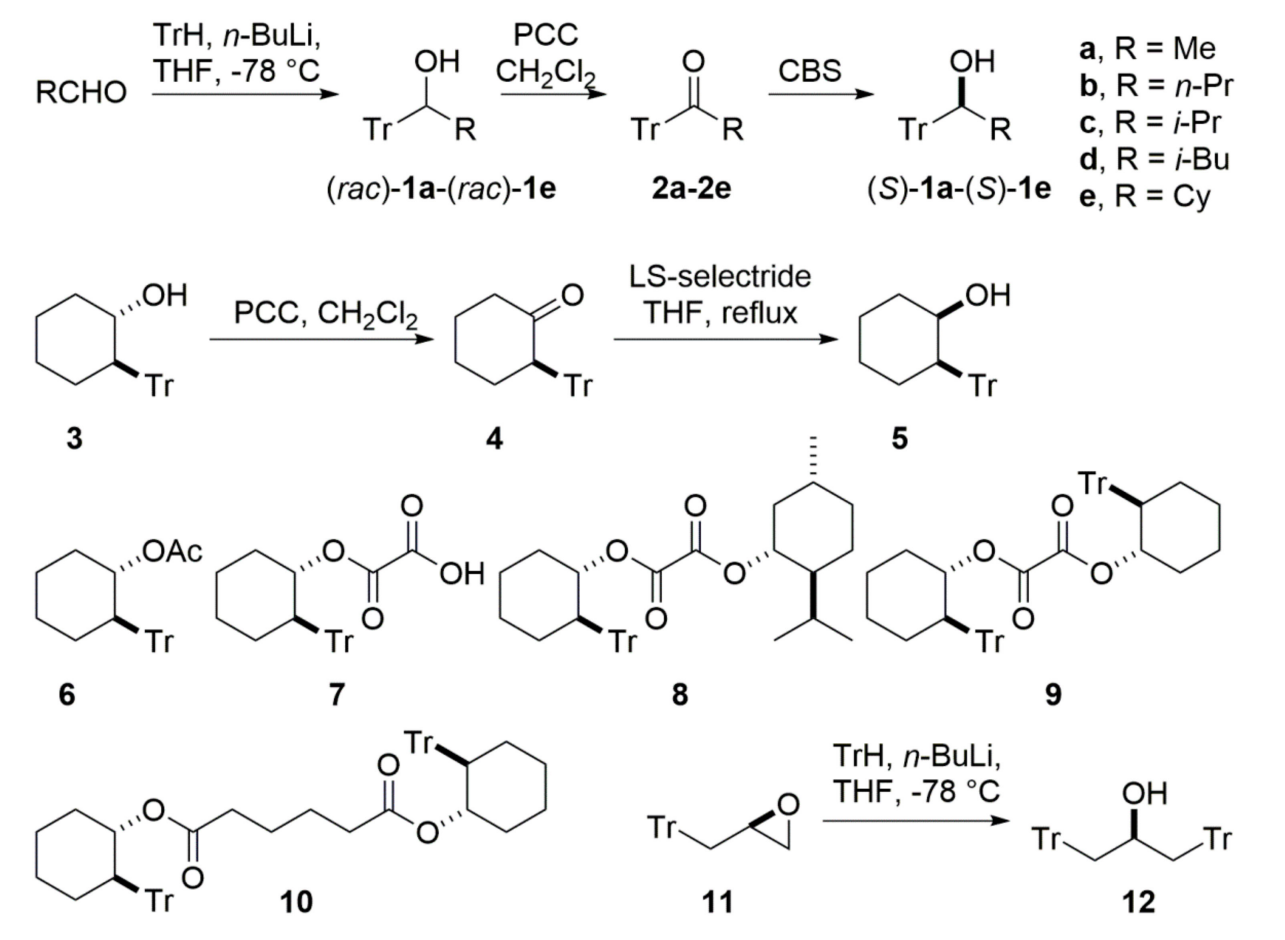
The general synthetic routes that provide optically active derivatives **1**, **3**–**5**, and *meso*-**12** and structures of the other compounds under study. In the case of **3**, **8**, and **9**, only one enantiomeric or diastereoisomeric form was shown.

In these studies, we have focused on derivatives **1**–**12** (Scheme 1) which, in principle, may be conveniently obtained by addition of a carbon nucleophile to a suitable electrophile and then further functionalized. However, the initially tested stereoselective addition of aliphatic *C*-nucleophiles to the triphenylacetaldehyde did not yield alcohols **1a**–**1e**. Thus, we have taken advantage of a facile generation and stability of trityl anion obtained through a direct reaction between triphenylmethane and *n*-buthyllithium. We have selected compounds **1**, **3**, **11,** and **12** as the basic structures, which might be obtained through the addition/substitution of the trityl anion to the respective electrophile (see Scheme 1).

With the exception of **11**, the applied synthetic method did not allow us to obtain the desired compounds in the optically pure form. In the case of **1a**–**1e**, we were forced to extend the synthetic procedure to the step of oxidation of racemic alcohols and asymmetric Corey–Bakshi–Shibata (CBS) reduction of the resulting ketones **2a**–**2e**, which, in turn, provided the enantiomerically enriched **1a**–**1e** [26,27,28]. The obtained enantiomeric excesses of **1a**–**1e** did not exceed 85%, but were sufficient to allow us to proceed with the study.

The racemic *trans*-2-tritylcyclohexanol (*rac*–**3**) was obtained from cyclohexene oxide through opening the epoxide ring with a trityl anion. The optically active **3**, in both enantiomeric forms, has been obtained from the racemate via oxalate **8** and by employing the procedure previously described by Hazmi et al [29]. The reactions provided both diasteroisomers of **8**, differing in the absolute configurations of the *trans*-2-tritylcyclohexanol moiety, denoted here as **8a** ((1*S*,2*R*)) and **8b** ((1*R*,2*S*)), respectively.

Oxidation of racemic and optically enriched (1*S*,2*R*)–**3** by PCC led to the ketone **4**, respectively, as the racemate and as the optically active form ((*R*)-**5**, ee = 80%). The ketone *rac*- or (*R*)-**5** may be further diastereoselectively reduced to the *cis*-2-tritylcyclohexanol (*rac*-**6** or (1*R*,2*R*)-**6**, if the enantiomerically enriched **5** was used), with the use of LS-Selectride. To complete the reduction reaction, a prolongated heating was required, and (1*R*,2*R*)-**5** was obtained with moderate yield (60%) and enantioselectivity (ee = 49%).

To enhance the steric bulkiness at the neighboring stereogenic center, and to show the influence of an external chiral unit on the possible chirality transfer, the optically pure **3** was acylated with various acid chlorides. While simple acetylation of **3** with acetyl chloride proceeded smoothly and gave acetate **6**, the initial attempt at synthesis of the diester **9,** which consists of two *trans*-2-tritylcyclohexanol units linked by the oxalyl acid moiety, failed. As the only product, we have obtained the monoester **7**, having one free carboxyl group. It is worth noting that the ESI-MS mass spectrum of **7** does not show peaks that might be ascribed to structure of isolated **7**. Instead, we observed a sodiated dimeric structure of the general formula [2 × **7** + Na]^+^. Surprisingly, the replacement of methylene chloride by freshly distilled THF and by performing the reaction at elevated temperature within 20 h led to the diester **9** with a yield of 41% after column chromatography. Despite the fact that the starting **3** was not optically pure (ee = 45%), the reaction was stereoselective and provided only chiral products. For the purpose of this study, we obtained both enantiomers **9** and *ent*-**9**. For no obvious reason, *ent*-**9**, which consists of two units characterized by the 1*R*,2*S* absolute configuration of each *trans*-2-tritylcyclohexanol moiety, crystallized better than **9** and provided crystals of a sufficient quality for X-ray structure determination.

The oxirane **11** was obtained according the procedure published by Zhang and coworkers [30]. In principle, oxirane **11** was to be the starting point for the further synthesis of an achiral stereodynamic reporter system consisting of two trityl fragments and an anchoring group enabling covalent or non-covalent binding of an inducer molecule. However, while the opening of the oxirane by the trityl anion led to an achiral *meso*-**12** with good yield, the further modification of the hydroxyl group turned out not to be possible, probably due to the steric congestion around the OH group.

### 2.2. Structure and Induced Circular Dichroism of **1**, **3**–**11**

The complementary use of experimental CD measurements and DFT calculations allows a study of the structure/chiroptical properties and mechanism of chirality transmission within the selected compounds. To achieve this goal, ECD spectra measurements are required in order to compare them to the results of DFT calculations, carried out at a sufficiently high level of theory (calculation details can be found in Supplementary Information). In this study, we have decided to discuss experimental and theoretical results together to preserve the logical stream.

**Figure 1:**
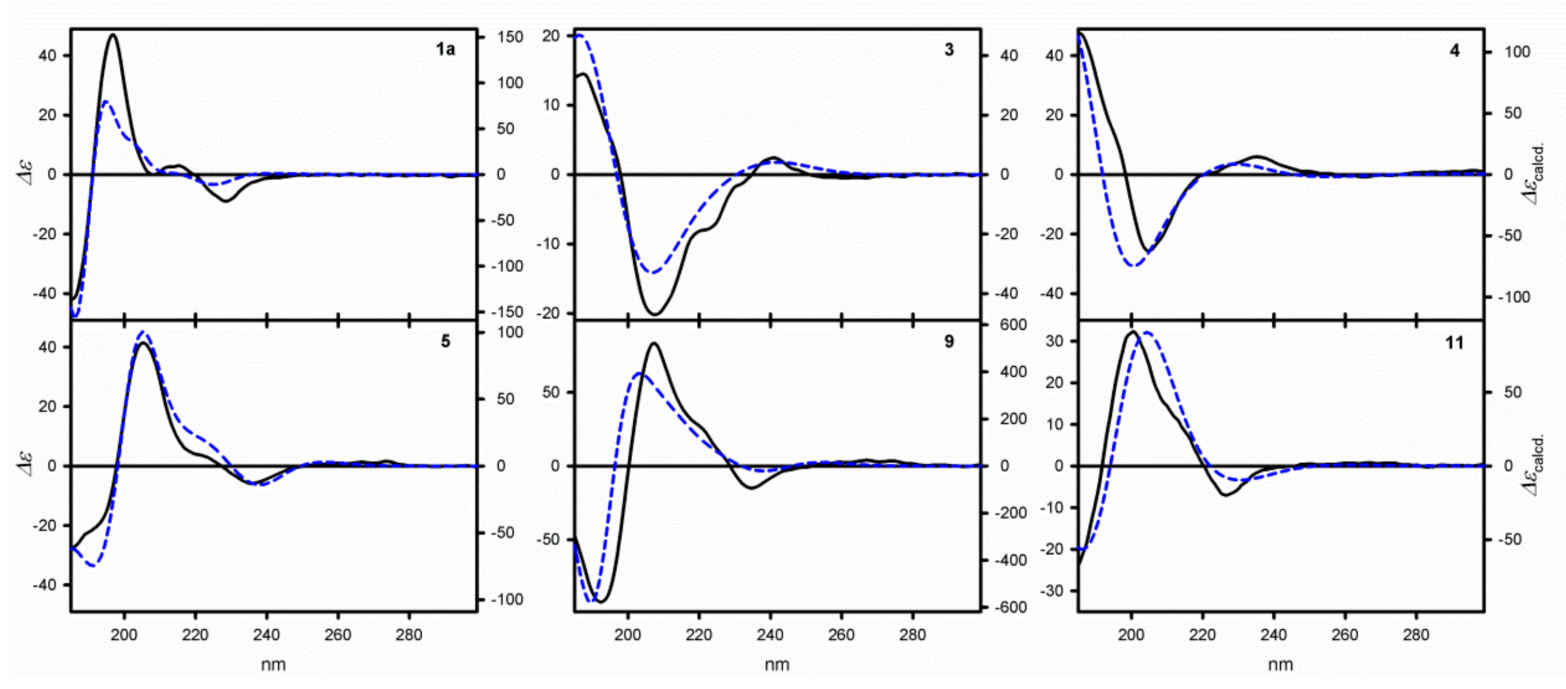
Exemplary ECD spectra of **1a**, **3**–**5**, **9,** and **11** measured in cyclohexane (solid black lines) and calculated at the TD-CAM-B3LYP/6-311++G(d,p) level for geometries optimized at the B3LYP/6-311++G(d,p) level (dashed blue lines). With the exception of **3**, the calculated ECD spectra were Boltzmann-averaged based on *ΔΔG* values. Wavelengths were corrected to match experimental UV maxima.

**Figure 2:**
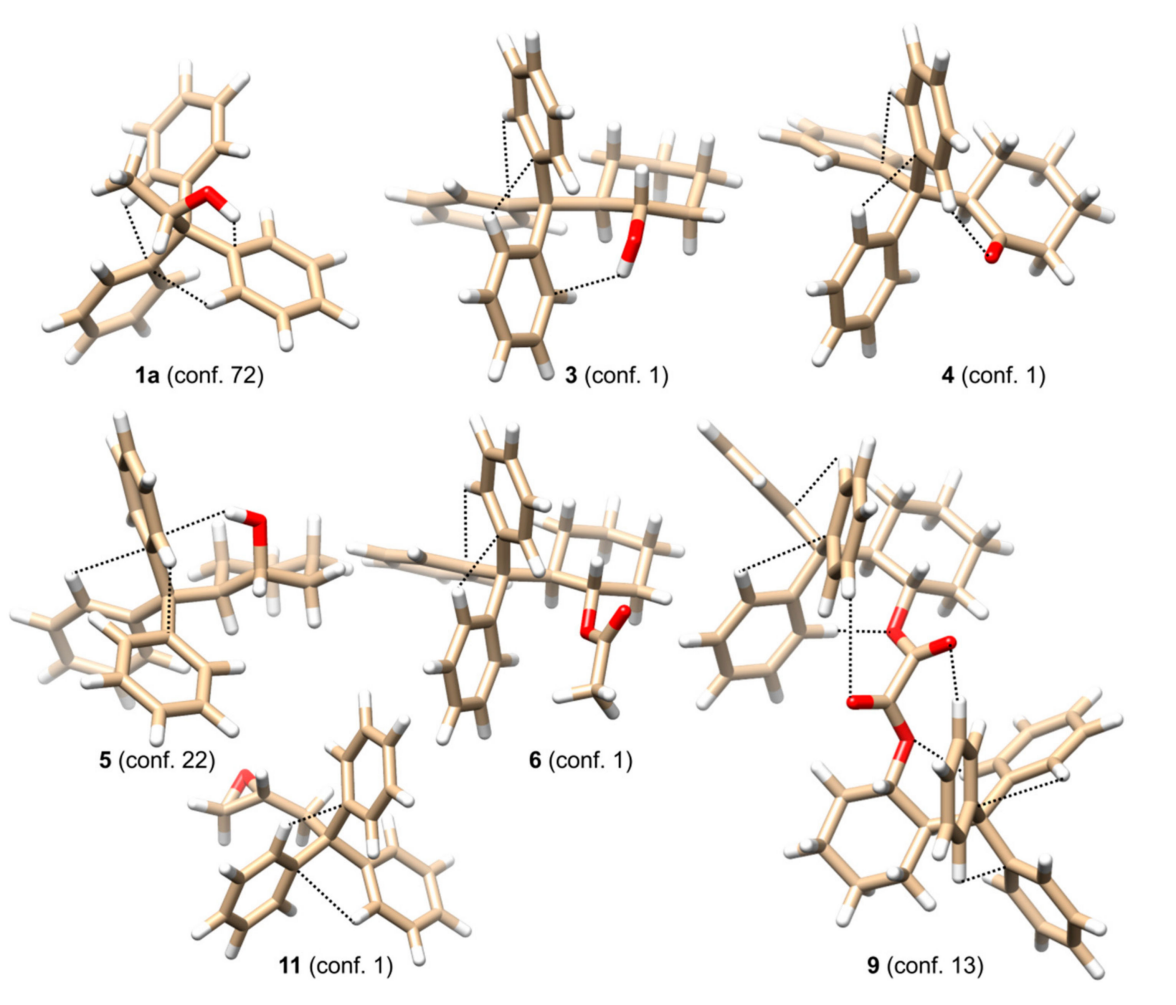
The structures of the lowest energy conformers of **1a**, **3**–**6**, **9**, and **11** calculated at the B3LYP/6-311++G(d,p) or B3LYP/6-311G(d,p) (compound **9**) level of theory. The dashed lines indicate possible attractive interactions (the remaining structures are deposited as Supplementary Information).

The ECD data for **1**–**11** are juxtaposed in Table 1, whereas the exemplary ECD spectra measured in non-polar cyclohexane for **1a**, **3**–**5**, **9,** and **11** are shown in Figure 1. The structure of the compounds under study can be described by providing the sets of torsion angles strictly determining the structure of the particular species. The angle *α* (X-C(=O)-C(H_2_)-C) characterizes the conformation of the carbon chain. The angles *β_1_* – *β_3_* ((O=C)-C(H_2_)-C-C*_ipso_*) describes the conformation of the phenyl groups in the relation to the inductor carbon chain. The sign, and to the same extent, the magnitude of observed CE’s depends on the helicity of the trityl group. The angles *γ_1_* – *γ_3_* (C-C-C*_ipso_*-C*_ortho_*) values may be used quantitatively for a precise description of the trityl helicity. However, for the needs of this work, it seems more convenient to use a qualitive descriptor, namely helicity of the blades: *M* (−90° < *γ* < 0°), *P* (0° < *γ* < 90°) or *0* (for *γ* angles deviating from zero by less than |5°|). Since, for the majority of the cases, the chiroptical properties are dominated by the most abundant conformer, we have limited our structural discussion to the *ΔΔG*-based the lowest energy species only. The structures of the lowest energy conformers of selected compounds **1a**, **3**–**6**, **9,** and **11** are shown in Figure 2, whereas Table 2 collects the essential information extracted from the theoretical results (the remaining data can be found in Supplementary Information).

The data listed in Table 1 clearly indicates large chiroptical response of the trityl chromophore in the presence of chirality element in its neighborhood. The measured ECD spectra are characterized by the presence of at least three well-defined absorption bands typical of trityl chromophore. The low-energy absorption band appearing at around 220 nm, is preceded by two bands with much higher intensity, which appear around 200 and 185 nm. In the case of the monoester **7**, the presence of an oxalyl acid fragment makes the ECD spectrum more complex.

Generally, the –COOR ester group is recognized as the weak UV active chromphore of the π-π* transition separated from the strong ^1^B band (195–210 nm) in phenyl rings. However, in the case of **7,** we are dealing not only with the presence of two different directly connected groups (COOH and COOR), but also with the possibility to form various associates [17].

As expected, the direct connection between the chirality element and the stereodynamic part of the molecule for **1a**–**1e** leads to the CEs of the highest intensity within the whole series. In tritylcarbinols **1a**–**1e**, the amplitudes (*A*) that are estimated for the higher-energy CEs reach values between 94 to 195. In fact, these *A* values are the highest for all the monotrityl derivatives studied so far. The signal patterns observed in all the spectra are identical: Negative/positive/negative (−/+/−), which suggests the same absolute configuration at the stereogenic centers. To confirm this hypothesis, for all of the alcohols **1a**–**1e**, DFT calculations (B3LYP/6-311++G(d,p)) were carried out [31]. Note that the choice of a calculation method was based on our previous studies. We have previously shown that the use of hybrid functionals, B3LYP for geometry and CAM-B3LYP for excited states calculations and for compounds containing trityl groups, gives results closest to the experimental ones [17,18]. Hence, we have decided to use these functionals in conjunction with the enhanced 6-311++G(d,p) basis set (or 6-311G(d,p) for larger systems) for calculations. The empirical correction for dispersion interactions does not improve the quality of the results obtained. In other words, the trityl–trityl interactions are usually overestimated, which ultimately leads to large discrepancies between the experiment and calculations [20].

The use of B3LYP hybrid functional is not recommended for excited state calculations, which is in accordance with our observations [17,18,20,32]. We received comparable results (let us say—of the same quality) to those obtained with the use of CAM-B3LYP functional, when the Thrular’s M06–2X hybrid functional was employed. In this work, only the results obtained with the use of TD-CAM-B3LYP hybrid functional were discussed.

In the simplest case of **1a**, only two thermally accessible conformers have been found. However, the number of thermally accessible conformers increases in alcohols with longer or more flexible alkyl backbones. Despite the structure of the alkyl substituent flanking the stereogenic center, two general factors can be distinguished as responsible for inducing helicity and, thus, the optical activity in the trityl chromophore. The first of them are attractive interactions between an oxygen atom from the hydroxyl group and a hydrogen atom in *ortho* position from one of the phenyl rings. The proton from the hydroxyl group is directed towards the plane of the second phenyl group. The O-H∙∙∙π interactions together with C-H∙∙∙π interactions stabilize the conformation of this particular phenyl ring. The third phenyl ring adjusts its conformation to the conformation of the remaining two.

The −/+/− signal pattern of CEs, observed for **1a**–**1e**, are due to the predominance of *PPP* or *PP0* helical conformers in conformational equilibria. This experimental/theoretical analysis provides an additional reward—the absolute configuration, determined for **1a**–**1e**, at the stereogenic centers is uniformly *S* for all of these compounds.

(1*S*,2*R*)-*trans*-2-Tritylcyclohexanol (**3**), its diastereoisomer (1*R*,2*R*)-*cis*-2-Tritylcyclohexanol (**5**), and intermediate ketone (*R*)-2-tritylcyclohexanone (**4**) constitute interesting examples showing the influence of a neighboring group (hydroxyl group or carbonyl oxygen atom) on trityl conformation. The lowest-energy conformations of **3** and **4** are stabilized mostly by C*_ortho_*-H∙∙∙O or O-H∙∙∙π attractive interactions and to a lesser extent by C*_ortho_*-H∙∙∙π and steric interactions between axial proton(s) attached to C1 and C3 carbon atoms in cyclohexane ring. The importance of C*_ortho_*-H∙∙∙O interactions is particularly seen for the lowest-energy conformer of **4**. The calculated (C*_ortho_*-)H∙∙∙O(=C) distance is 2.210 Å. The case of **5** is different. The axial position of hydroxyl group prevents direct C*_ortho_*-H∙∙∙O interactions. Therefore, the primordial factor that determines the conformation of **5** seems to be O-H∙∙∙π interactions.

As mentioned previously, the process of optical induction in the trityl chromophore is cascading. The electrostatic C*_ortho_*-H∙∙∙O (if applicable) interactions constitute the primary factor that fixes the conformation of the phenyl rings that faces the axial protons at C1 and C3 and enforces a (+)-*synclinal* ((+)-*sc*) conformation of associated angle *β*. Then a second ring is positioned in such a way as to allow O-H∙∙∙π interactions with the hydroxyl proton, and C*_ortho_*-H∙∙∙π interactions with the first phenyl ring. Finally, the third ring adjusts its conformation to the first two in a way to maximize C*_ortho_*-H∙∙∙π interactions. The opposite absolute configuration reached by the change in the position of hydroxyl group from equatorial to axial is reflected in the change of CE sequence. For **3** and **4,** the CE sequence which appears in the region of trityl absorption is +/−/+, whereas for **5** it is reversed (−/+/−). These sequences correspond to the preferred helicities of the trityl group, namely *MPM* and *PPM* for the most abundant conformers of **3** and **5**, respectively. It is worth mentioning that the presence of the trityl, which acts as a protective umbrella, is responsible for the non-reactivity of the axial hydroxyl group, particularly in reactions requiring OH activation prior nucleophilic substitution.

Having compared the ECD spectra of **3** and **6,** we may conclude that the acetylation of the equatorial hydroxyl group does not have any significant effect on the amplitude and the sequence of the respective CEs. In both cases, the same +/−/+ sequence is observed. However, the mechanism of optical activity induction, deducted from our theoretical results, is slightly different from that established for the free alcohol. In the case of **6**, the C*_ortho_*-H∙∙∙O attractive interactions lose their importance in favor of sterical interactions between the acetyl group and the adjacent phenyl ring of trityl.

Due to the unrestricted rotation around the C-C bond in the linker, the adipinic acid derivative **10** is characterized by a great conformational flexibility. While the shape of the ECD spectrum of **10** resembles that of acetate **6** and of the free alcohol **3**, the magnitudes of its CE doubles. This is not surprising, given that the observed effects are a linear combination of contributions from individual (non-interacting) trityl chromophores.

Compounds **7**–**9** represent systems whose common denominator is the presence of an oxalyl moiety. Despite structural differences, these compounds display the same −/+/− sequence of CE observed in the region of the trityl UV absorption. This is apparently due to the dominant influence of conformers of *PPM* helicity of the trityl group(s) on the overall ECD spectra.

Among oxalates **7**–**9**, the most interesting one is represented by the symmetrical *bis*((1*S*,2*R*)-2-tritylcyclohexyl) oxalate (**9**). Conformation of the *C*_2_-symmetrical, lowest-energy conformer of **9** is stabilized by a set of weak C*_ortho_*-H∙∙∙O interactions that involve all C=O and C-O oxygen atoms present in the linker. Moreover, the C*_ortho_*-H∙∙∙O=C interactions involve the phenyl group characterized by the (+)-*sc* conformation of the *β* angle and a more distant oxygen atom, while C*_ortho_*-H∙∙∙O-C interactions take place between the phenyl group of (−)-*sc* conformation for the *β* angle and with a closer oxygen atom. The third phenyl group of (+)-*antiperiplanar* ((+)-*ap*) conformation for the *β* angle adjusts its position in a way to minimize steric repulsion while allowing some C*_ortho_*-H∙∙∙π interactions.

**Figure 3:**
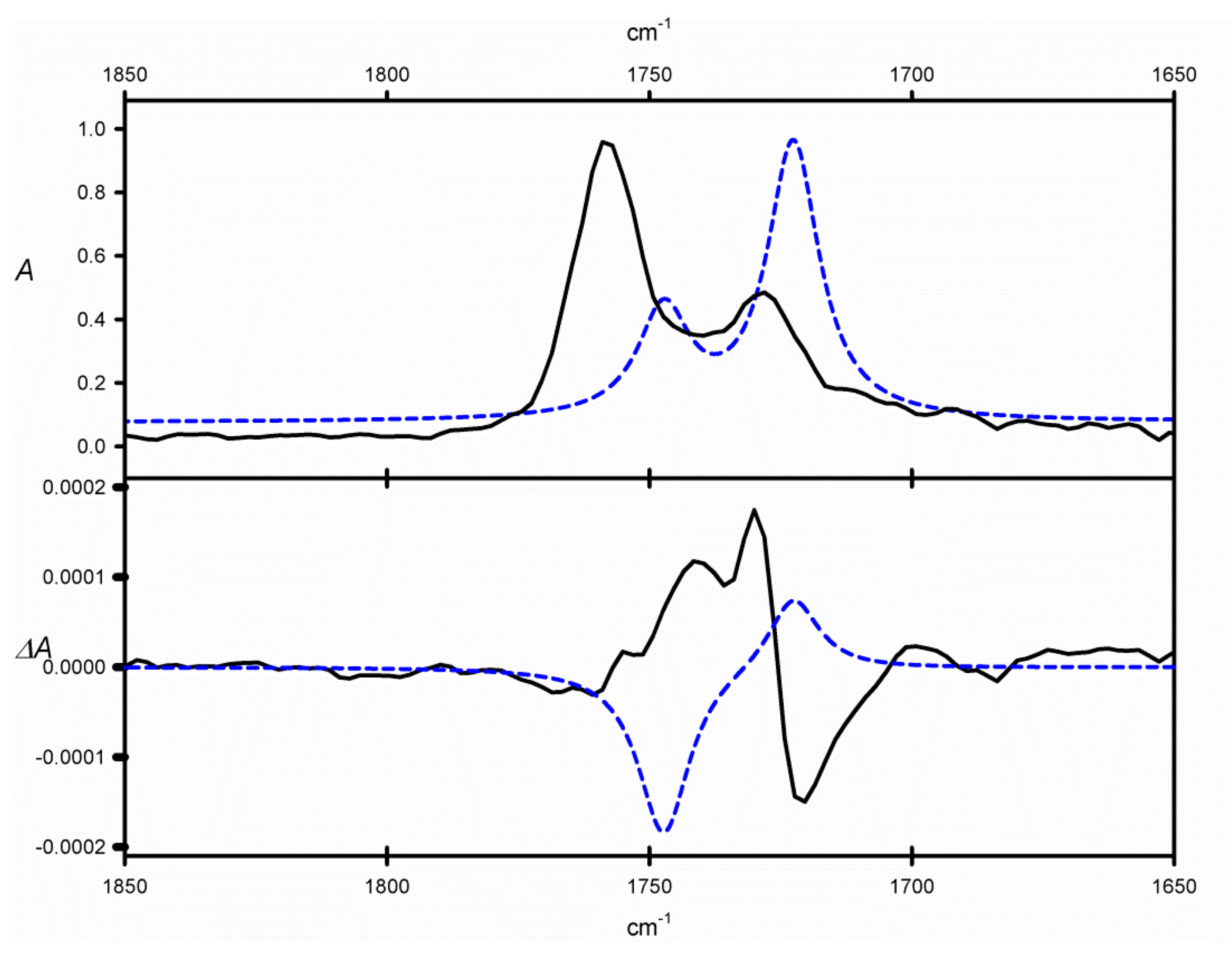
IR (upper panel) and vibrational circular dichroism (VCD) (lower panel) spectra of **9**, measured in CCl_4_ (solid black lines) and calculated (blue dashed lines) for geometries optimized at the IEFPCM (CCl_4_)/B3LYP/6-311G(d,p) level. The calculated VCD spectra were Boltzmann-averaged based on *ΔΔG* values and were not scaled. Only the region of C=O absorption (1850–1650 cm^−1^) is shown. The experimental, measured in the region between 850 and 2000 cm^−1^ and calculated IR and VCD spectra are available in Supplementary Information.

The C*_ortho_*-H∙∙∙O interactions not only stabilize the conformation of the trityl groups, but also cause a bending of the oxalate linker. In the lowest-energy conformer of **9**, both COOR fragments are arranged almost perpendicularly (*ϕ* = 91.4°, where *ϕ* is the O=C-C=O angle), whereas the higher energy conformer number 26 retains the (−)-*ap* orientation (*ϕ* = −162.9°). The non-planarity of the *ϕ* angle gives a chance for observation of non-zero CE in the region of C=O stretches in the VCD spectrum of **9**. In fact, the VCD spectrum of **9** measured in CCl_4,_ shows a bisignate couplet in the region of C=O absorption (1700–1800 cm^−1^, see Figure 3) that may be ascribed to through-space interactions of two C=O chromophores. However, the negative sign of this couplet remains inconsistent with the twist of the neighboring vicinal C=O bonds. Similarly, the DFT calculations gave results that are opposite to the experimental ones, regardless of the calculation method used. A possible explanation for this discrepancy could follow from the underestimation of structures characterized by (+)-*sp* or (+)-*synclinal* ((+)-*sc*) conformations of the O=C-C=O angle. Despite these disappointing results, this particular compound is an interesting example of shortcomings in the experimental/theoretical approach to structural studies with the use of VCD spectroscopy. Further studies on this and related compounds will be undertaken in the near future.

The oxirane **11** represents a somewhat different system, in which the trityl group is connected indirectly to the stereogenic center. In this particular case, the reporter part of the molecule should be sensitive enough to distinguish distant substituents of the stereogenic center. Among the thermally accessible conformers, two of them, number 1 and number 73, dominate in conformational equilibrium and are characterized by *PP0* and *PPP* helicities of the trityl fragment, respectively. The ECD spectra, calculated for these conformers, exhibit the same −/+/− sequence of CE, in perfect agreement with the experimental ECD spectrum. The structure of the trityl moiety in low-energy conformers of **11**, is stabilized mostly by C*_ortho_*-H∙∙∙π interactions and to a lesser extent by electrostatic repulsion between the oxygen atom and the π-electron cloud of the phenyl ring facing the oxirane moiety.

### 2.3. X-ray Diffraction Study

Taking advantage of the ability of some compounds to form crystal phases, we have extended our work to the solid phases of the trityl-containing molecules. In particular, we were interested in the possibilities to form inclusion compounds, which in turn may lead to enantiodiscrimination. The crystal structure of compounds *rac*-**3**, *rac*-**4**, (*R*)-**4**, *rac*-**5**, *rac*-**7**, **8**, **9**, **11,** and **12** has been determined and studied by means of the X-ray diffraction method (detailed information is included in Supplementary Information). The derivatives obtained, with the exception of compound *rac*-**7**, crystallize without solvent. Some structural gaps are observed in the crystals of the *rac*-**4**, (*R*)-**4,** and **9**, however the accessible volume is too small to include the solvent molecules. Nevertheless, in crystals of *rac*-**3**, *rac*-**5**, **8**, **11,** and **12,** the molecules strictly fill the space, and despite the tests carried out, inclusion crystals containing at least solvent molecules have not been obtained.

**Figure 4:**
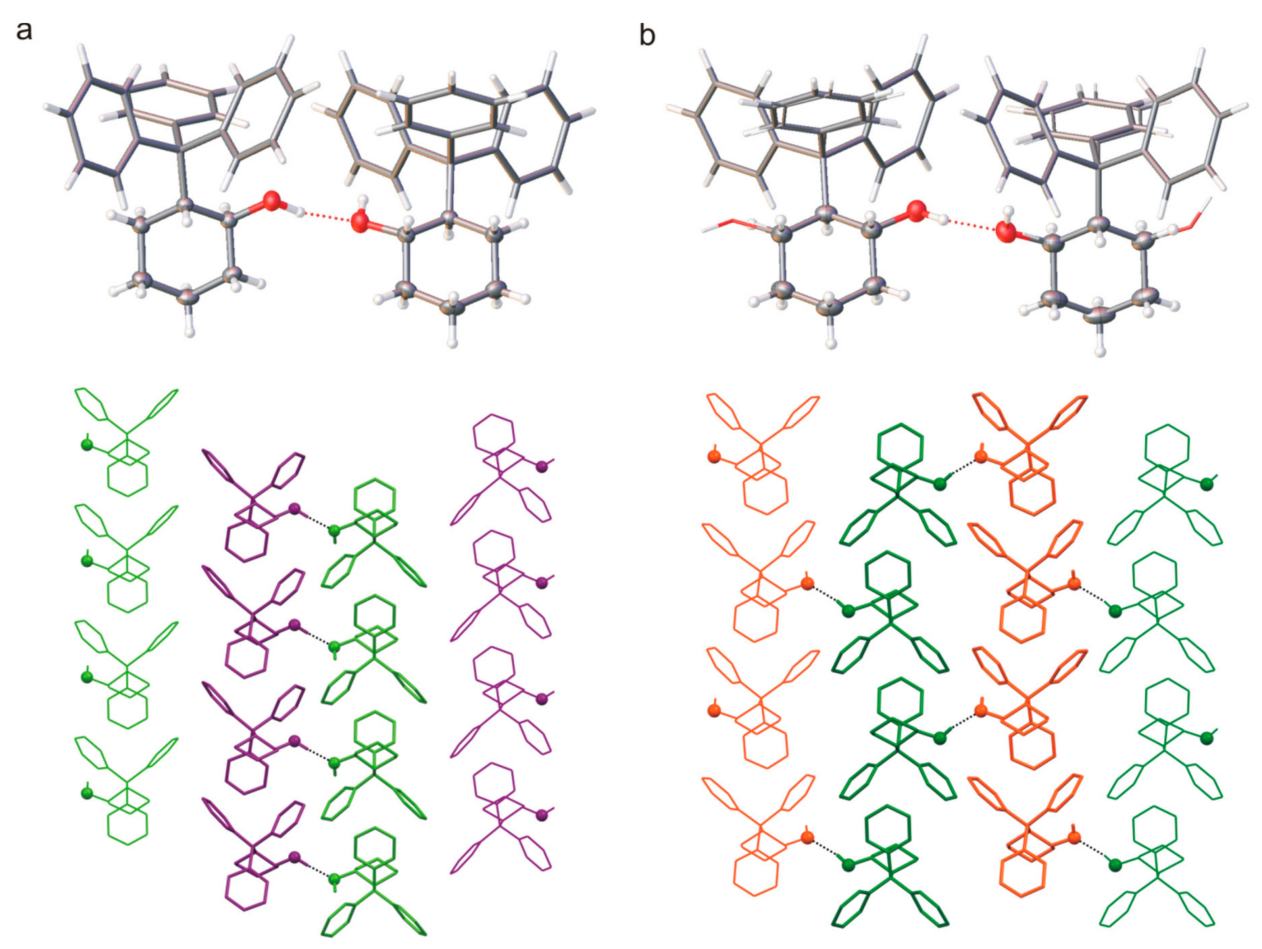
The molecular structure of asymmetric unit and supramolecular column in crystal structure of compound *rac*-**3**: (**a**) polymorph α,( **b**) polymorph *β*. The oxygen atoms are presented as balls and hydrogen bonds are shown as dashed lines.

Interestingly, the two types of crystals of compound *rac*-**3** (orthorhombic (*rac*-**3**-*α*) and monoclinic (*rac*-**3**-*β*) were obtained in one crystallization, even though the structure of the molecule is the same. Furthermore, the structural analysis showed that the polymorph *rac*-3-*β* is a solid solution of enantiomers (Figure 4b), and the ratio of refined occupancy factors for oxygen atoms is 0.71: 0.29. In both orthorhombic and monoclinic crystals, the basic structural unit is a pair of (1*S*,2*R*) or (1*R*,2*S*) molecules interacting through the O-H∙∙∙O hydrogen bond. The helicity of the trityl group for all molecules observed in the crystal phase, both in *α* and *β* forms, is *MPM*, which corresponds to the conformation of the calculated low-energy structure. A hydrogen atom of the OH group that is not involved in this hydrogen bond, is further involved in the O-H∙∙∙*π* interaction, which stabilizes the structure of the molecule. In the *rac*-3-*α* crystals, pairs of hydrogen bonded molecules form columns stabilized by C-H∙∙∙*π* interactions (Figure 4a). However, similar columns are observed in the beta form structure where the molecules from adjacent columns interact with each other through O-H∙∙∙O hydrogen bonds (Figure 4b). The three-dimensional structure is stabilized by weak interactions involving *π*-electron systems.

Crystals of (*R*)-**4**, where the compound crystallizes with two molecules in the asymmetric unit cell (helicity *MPM* and *PMP*) where one of them is disordered (the ratio of refined occupancy factors for oxygen atoms is 0.88: 0.12) turned out to be an unusual case. For the disordered molecule, the position of the cyclohexane ring is undefined, and it mimics the phenyl ring.

**Figure 5:**
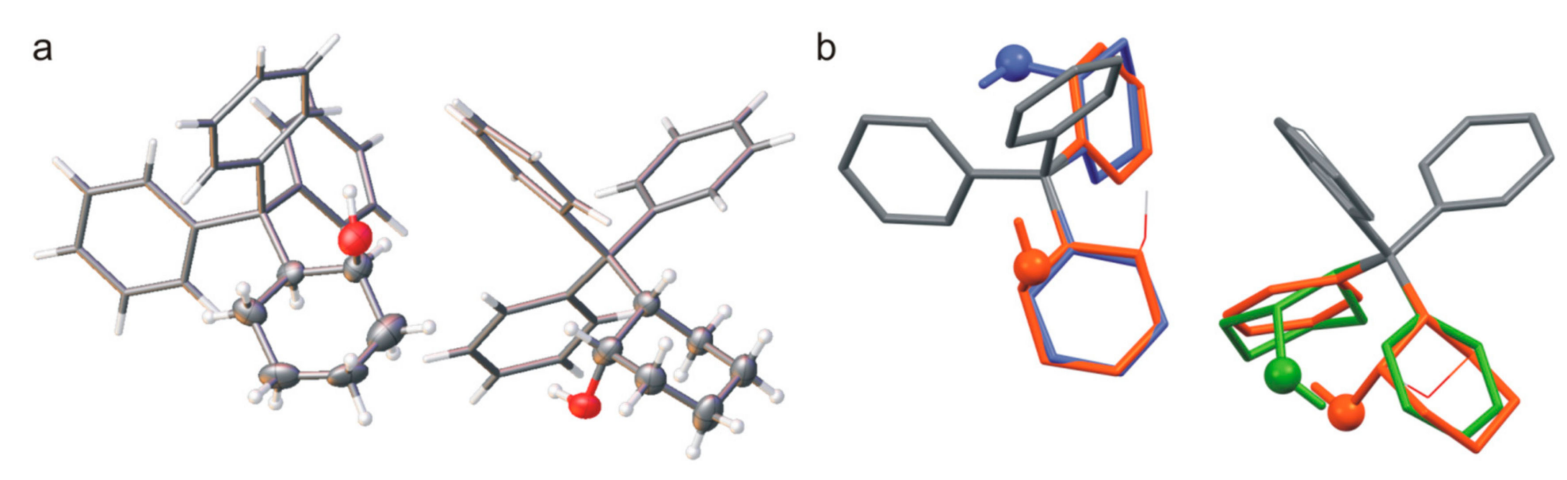
(**a**) Molecular structure of compound *ra*c-**5**, (**b**) molecular is represented by the superposition of different conformation in different colors.

Molecular disorder is also observed in the crystals of *rac*-**5** (Figure 5). The compound crystallizes in the triclinic system (symmetry *P* $\bar{1}$), with two molecules in the asymmetric unit cell where both of them are disordered—the cyclohexyl ring mimics the phenyl ring in the crystal structure. In addition, the enantiomer molecules can occupy the same positions in the crystal, and the *cis*-2-tritylcyclohexanol crystallizes as a solid solution of enantiomers, as in the case of **3**-*β*. The helicity of the trityl group for all molecules observed in crystal is *MPM* (or *PMP*), which corresponds to the conformation calculated for the low-energy structure. The hydrogen atoms of the OH groups do not form strong intermolecular hydrogen bonds, as they participate in intramolecular O-H∙∙∙*π* interactions stabilizing the structure of the molecule. The formation of solid solutions by compounds containing a bulky trityl group has previously been observed once and may be due to supramolecular protective properties of the substituent [33].

**Figure 6:**
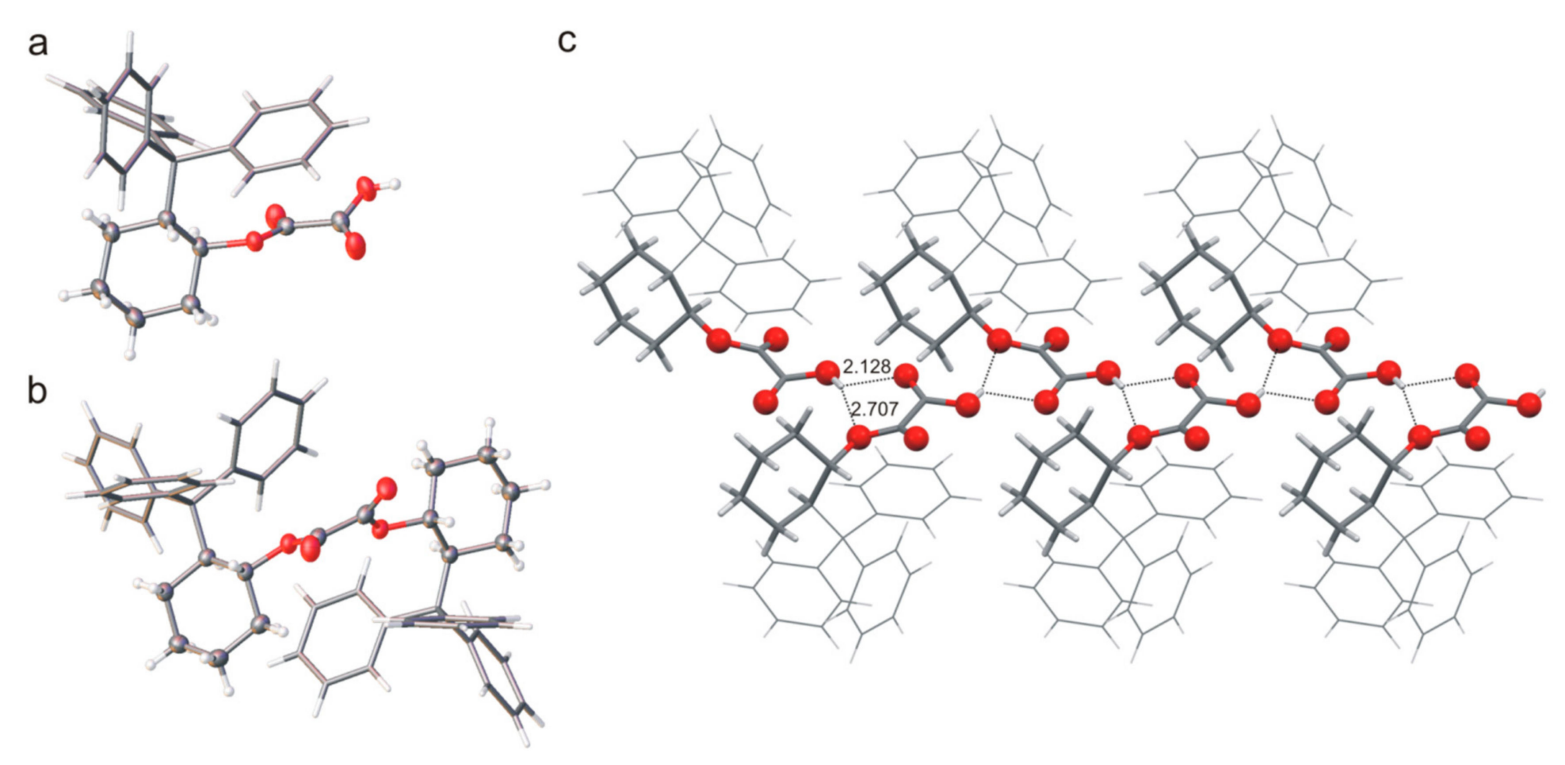
Molecular structure of (**a**) compound **7**, (**b**) compound **9,** and (**c**) supramolecular chain via hydrogen bonds in structure **7** (hydrogen bonds showed as a dashed line). The oxygen atoms are shown as balls.

The common feature of compounds **7**, **8,** and **9** is the presence of an oxalyl fragment in the molecule. The conformation of this fragment is described by angle *ϕ* (defined as O=C-C=O), which is −152.6(2)° for the monoester **7**, 127.8(2)° for **8,** and −32.1 (4)° for the diester **9**. The molecular structure of monoester **7** is stabilized by intramolecular interaction of the phenyl ring and the almost parallel oxalyl part (Figure 6). The free carboxyl group forms the bifurcate O-H∙∙∙O hydrogen bonds and participates in the formation of the supramolecular tape observed in the crystal structure. As mentioned, the ability to form inclusions was confirmed only for the compound **7** and the crystals obtained contain molecules of the solvent (chloroform). The stacking-like arrangement of phenyl ring and oxalyl part is not preserved in the structure of the diester **9**, where the presence of a second *trans*-2-tritylcyclohexanol unit forces a change in molecules geometry.

**Figure 7:**
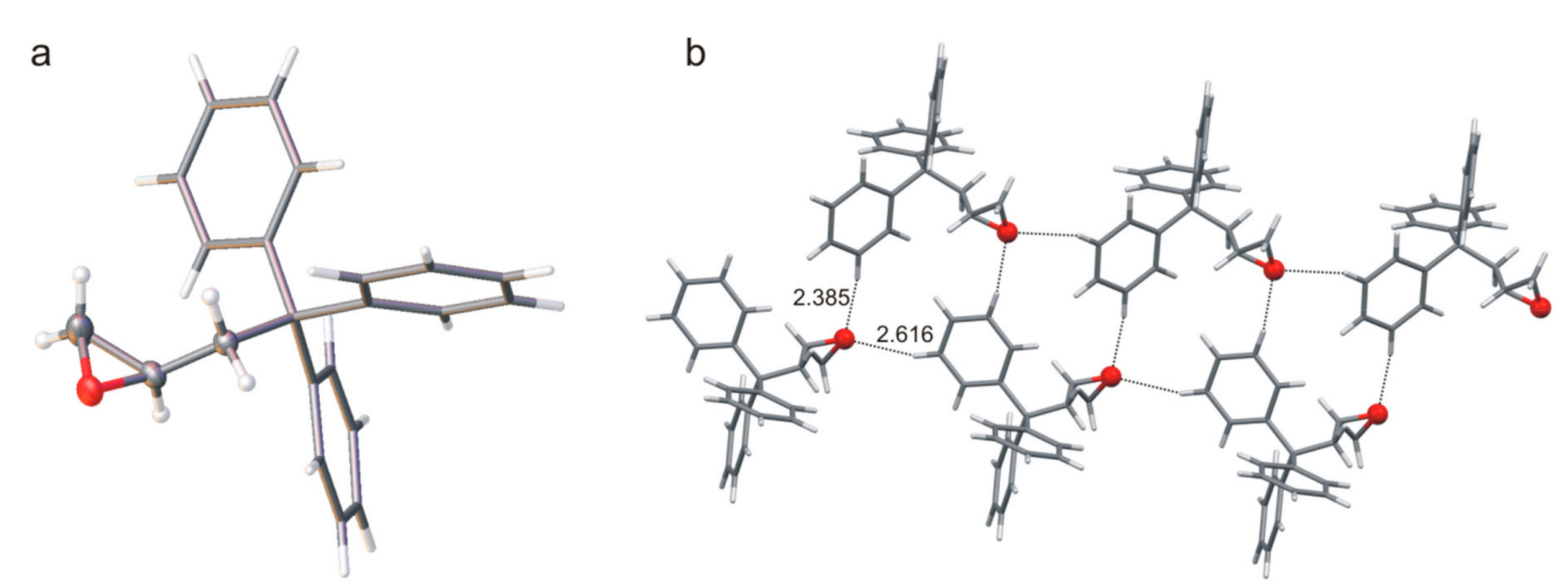
(**a**) Molecular structure of compound **11** and (**b**) intermolecular interaction in crystal structure. The oxygen atoms are shown as balls. The hydrogen bonds are shown as dashed line.

In the crystal structure of compound **11**, the asymmetric unit cell consists of one molecule with *MMM* helicity of trityl group (*PP0* for calculated the low-energy structure). The oxygen atom is involved in C-H∙∙∙O hydrogen bond and molecules form a ladder-like structural motif (Figure 7). These C-H∙∙∙O interactions stabilize the structure of the molecule as well. The whole three-dimensional crystal structure is stabilized by weak interactions involving aromatic systems.

Compound **12** crystallizes with two molecules in the asymmetric unit cell. Interestingly, OH groups do not form strong hydrogen bonds. C_ortho_-H∙∙∙O interactions are observed between the molecules, while the OH groups are involved in O-H∙∙∙*π* interactions. The OH group has a great potential for hydrogen bonding and constitute a reliable feature in planning the crystal structure. As shown, the presence of a trityl group in close proximity to the hydroxyl group limits its availability for forming hydrogen bonds. This observation confirms that the trityl group can act as a protective group in crystal engineering.

## 3. Materials and Methods

Detailed experimental procedures, details regarding X-ray diffraction studies, and theoretical calculations are given in Supplementary Information.

## 4. Conclusions

In this work, we have proposed a complementary approach to the problem of chiral derivatives, in which the sterodynamic part of the molecule is directly connected to the stereogenic center or is one CH_2_ group removed. Despite high conformational freedom of some derivatives, we have shown the possibility of chirality transfer from the permanent chirality element to the stereodynamic trityl group, through the set of relatively weak but complementary electrostatic interactions. The presence of OH and/or C=O in the molecule is a factor that organizes and stabilizes the conformation. As a result, the trityl group adopts helical conformation, which is revealed by the appearance of non-zero CE in the region of trityl absorption. In the cases of more complex derivatives that contain more than one chirality element, their respective CE signs sequences appear between 220 and 185 nm are not a simple function of the absolute configuration at the stereogenic center. In these cases, the shapes of ECD spectra were found to depend on the configuration of the additional stereogenic center, not directly connected with the trityl group.

Due to the presence of two chiral fragments, the oxalyl linker in diester **9**, adopts non-planar conformation. Such a geometry allows, in principle, the observation of exciton-type CE’s in the region of C=O stretches in VCD spectrum of **9**. The bisignate couplet in the region of C=O absorption, observed in the VCD spectrum of **9**, may be ascribed to through-space interactions of 2 C=O chromophores. However, further studies on this and related compounds will be carried out in the near future.

The presence of the trityl group allowed, to some extent, for the control over the supramolecular assembly of alcohol derivatives. The molecules in the crystal form various aggregates stabilized by O-H∙∙∙O hydrogen bonding. On the other hand, the presence of two trityl groups in the neighborhood of OH hydroxyl group preclude formation of OH···O hydrogen bonding. Therefore, in this case, the trityl group acts as a supramolecular protective group. Additionally, the presence of the “protecting umbrella” is a decisive factor that is responsible for the formation of solid solutions by racemates of *trans*- and *cis*-2-tritylcyclohexanol. Strictly speaking, the packing of the molecules in the crystal is determined by the trityl, not by the absolute configuration of the chirality element. The protective function of the trityl group can, in turn, be used in crystal engineering.

## Figures and Tables

**Table 1:** Electronic circular dichroism (ECD) (*Δε*) and UV (*ε*) data for derivatives **1**, **3**–**11** measured in cyclohexane solution.

Compd.	ECD [*Δε* (nm)]	UV [*ε* (nm)]
**1a**	−9.0 (228); 2.9 (215); −0.1 (208); 47.0 (197); −41.8 (185)	70,500 (194)
**1b**	−8.0 (229); 1.9 (214); 50.4 (197); −41.8 (186)	67,500 (194)
**1c**	−6.3 (232); 45.6 (200); −52.8 (185)^a^	67,800 (194)
**1d**	−5.0 (229); 2.6 (217); −9.1 (207); 43.4 (196); −31.1 (186)	69,700 (194)
**1e**	−6.2 (233); 52.5 (201); −62.3 (186)	67,400 (194)
**3**	2.4 (241); −20.2 (207); 14.0 (185)^a^	8,000 (236); 73,300 (193)
**4**	1.4 (296); −0.8 (265); 6.0 (235); −25.7 (205); 47.5 (185)^a^	1,200 (265); 74,500 (190)
**5**	−5.9 (235); 41.4 (205); −28.0 (185)^a^	70,600 (194)
**6**	1.0 (240); −8.5 (222); −14.7 (204); 20.9 (185)^a^	8,400 (236); 76,100 (193)
**7** ^b^	−2.9 (238); −3.7 (220); 4.3 (207); −2.9 (197); 8.5 (185)^a^	59,700 (193)
**8**	−2.5 (238); 3.2 (220); 11.4 (207); −6.5 (191)	5,500 (248); 83,600 (193)
**9**	−14.6 (235); 83.3 (207); −92.4 (192)	147,000 (192)
**10**	+5.0 (238); −24.0 (204); 45.0 (185)^a^	20,000 (238); 135,000 (193)
**11**	−7.0 (226); 32.3 (200); −24.0 (185)^a^	77,900 (197)

[a] end of measuring range; [b] partially insoluble in cyclohexane.

**Table 2:** The *ΔΔG*-based percentage populations (Pop.) and helicities of trityl chromophore calculated for the lowest energy conformers of **1a**–**1e**, **3**–**9,** and **11** at the B3LYP/6-311++G(d,p) level of theory.

Compound ^a^	Pop.	Helicity
**1a** (conf. 72)	55	*PPP*
**1b** (conf. 27)	54	*PPP*
**1c** (conf. 9)	45	*PP0*
**1d** (conf. 25)	42	*PPP*
**1e** (conf. 14)	85	*PP0*
**3** (conf. 1)	46	*MPM*
**4** (conf. 1)	100	*MM0*
**5** (conf. 22)	97	*PPM*
**6** (conf. 1)	100	*MPM*
**7** (conf. 13)	42	*PPM*
**8** (conf. 5)	78	*PPM*
**9** (conf. 13) ^b^	100	*PPM*
**11** (conf. 1)	50	*PP0*

[a] The conformers are numbered according to their appearance during conformational search; [b] calculations were carried out at the B3LYP/6-311G(d,p) level.

## Supplementary Materials

The following are available online at https://www.mdpi.com/1420-3049/25/3/707/s1, Experimental details, Calculation details, X-ray diffraction study details, copies of ^1^H and ^13^C NMR spectra, Figures S1-S102; Tables S1-S4. CCDC 1977921-1977930 contains the supplementary crystallographic data for this paper. These data can be obtained free of charge via www.ccdc.cam.ac.uk/data_request/cif, or by emailing data_request@ccdc.cam.ac.uk, or by contacting The Cambridge Crystallographic Data Centre, 12 Union Road, Cambridge CB2 1EZ, UK; fax: +44 1223 336033.
