# Supplementary material for: Trityl-Containing Alcohols—An Efficient Chirality Transmission Process from Inductor to the Stereodynamic Propeller and their Solid-State Structural Diversity
Source: Molecules. 2020 Feb 6;25(3):707. doi: 10.3390/molecules25030707 (PMC7036902; doi:10.3390/molecules25030707)
Supplement: Supplementary file 1 [file molecules-25-00707-s001.pdf]

Supplementary Material for:

Trityl-containing alcohols – an efficient chirality transmission process from inductor to the stereodynamic propeller and their solid-state structural diversity

Sylwia Górczyńska,[1,2] Aleksandra Brzdonkiewicz,[1] Maciej Jelecki,[1,2] Agnieszka Czapik,[1,2] Bartosz Stasiak,[1] and Marcin Kwit[1,2]*

1 Department of Chemistry Adam Mickiewicz University, Uniwersytetu Poznańskiego 8, 61 614 Poznań, Poland; sylwia.gorczynska@amu.edu.pl (SG); ola.brzdonkiewicz@gmail.com (AB); maciej.jelecki@gmail.com (MJ); aga_cz@amu.edu.pl (AC); bartosz.stasiak@amu.edu.pl (BS); marcin.kwit@amu.edu.pl (MK)

2 Centre for Advanced Technologies Adam Mickiewicz University, Uniwersytetu Poznańskiego 10, 61 614 Poznań, Poland; sylwia.gorczynska@amu.edu.pl (SG); maciej.jelecki@gmail.com (MJ); aga_cz@amu.edu.pl (AC); marcin.kwit@amu.edu.pl (MK)

Table of contents

Experimental details 10

Syntheses of racemic alcohols 1a-1e 10

Syntheses of ketones 2a-2e 10

Asymetric reductions of ketones 2a-2e 11

(*rac*)-*trans*-2-Tritylcyclohexan-1-ol ((*rac*)-3) 15

(1*S*,2*R*)-*trans*-2-Tritylcyclohexan-1-ol (3) 15

(*rac*)-2-Tritylcyclohexan-1-one ((*rac*)-4) 15

(*R*)-2-Tritylcyclohexan-1-one (4) 16

(1*R*,2*R*)-*cis*-2-Tritylcyclohexan-1-ol (5) 16

(1*S*,2*R*)-2-Tritylcyclohexyl acetate (6) 17

Menthyl ester 7 17

2-Oxo-2-(((1*R*,2*S*)-2-tritylcyclohexyl)oxy)acetic acid (8) 18

Bis((1*R*,2*S*)-2-tritylcyclohexyl) oxalate (9) 18

Bis((1*S*,2*R*)-2-tritylcyclohexyl) adipinate (10) 19

(*R*)-2-(2,2,2-Triphenylethyl)oxirane (11) 19

1,1,1,5,5,5-Hexaphenylpentan-3-ol (12) 20

Calculation details 21

Table S_1. Total energies (in Hartree), relative energies (*ΔE*, *ΔΔG* in kcal mol^{-1}), percentage populations and number of imaginary frequencies (#ImFq) calculated at the B3LYP/6-311++G(d,p) level for individual conformers of 1a-1e, 3-9 and 11. 22

Table S_2. Dihedral angles *α*, *β*, *γ* and *δ* (in degrees) and selected interatomic distances l_1 and l_2 (in Å) calculated at the B3LYP/6-311++G(d,p) level for individual low-energy conformers of 1a-1e, 3-9 and 11. 24

Figure S_1. Structures of individual, low-energy conformers of 1a, calculated at the B3LYP/6-311++G(d,p) level of theory. 27

Figure S_2. Structures of individual, low-energy conformers of 1b, calculated at the B3LYP/6-311++G(d,p) level of theory. 28

Figure S_3. Structures of individual, low-energy conformers of 1c, calculated at the B3LYP/6-311++G(d,p) level of theory. 29

Figure S_4. Structures of individual, low-energy conformers of 1d, calculated at the B3LYP/6-311++G(d,p) level of theory. 30

Figure S_5. Structures of individual, low-energy conformers of 1e, calculated at the B3LYP/6-311++G(d,p) level of theory. 31

Figure S_6. Structures of individual, low-energy conformers of 3, calculated at the B3LYP/6-311++G(d,p) level of theory. 32

Figure S_7. Structures of individual, low-energy conformers of 4-6, calculated at the B3LYP/6-311++G(d,p) level of theory. 33

Figure S_8. Structures of individual, low-energy conformers of 7, calculated at the B3LYP/6-311++G(d,p) level of theory. 34

Figure S_9. Structures of individual, low-energy conformers of 8, calculated at the B3LYP/6-311++G(d,p) level of theory. 35

Figure S_10. Structures of individual, low-energy conformers of 9, calculated at the B3LYP/6-311++G(d,p) level of theory. 36

Figure S_11. Structures of individual, low-energy conformers of 11, calculated at the B3LYP/6-311++G(d,p) level of theory. 37

Figure S_12. UV and ECD spectra of the low-energy conformers of compound 1a calculated at the TD-CAM-B3LYP/6-311++G(d,p) level for structures optimized at the B3LYP/6-311++G(d,p) level. Wavelengths have not been corrected. 38

Figure S_13. UV and ECD spectra of the low-energy conformers of compound 1a calculated at the TD-M06-2X/6-311++G(d,p) level for structures optimized at the B3LYP/6-311++G(d,p) level. Wavelengths have not been corrected. 38

Figure S_14. UV and ECD spectra of the low-energy conformers of compound 1b calculated at the TD-CAM-B3LYP/6-311++G(d,p) level for structures optimized at the B3LYP/6-311++G(d,p) level. Wavelengths have not been corrected. 39

Figure S_15. UV and ECD spectra of the low-energy conformers of compound 1b calculated at the TD-M06-2X/6-311++G(d,p) level for structures optimized at the B3LYP/6-311++G(d,p) level. Wavelengths have not been corrected. 39

Figure S_16. UV and ECD spectra of the low-energy conformers of compound 1c calculated at the TD-CAM-B3LYP/6-311++G(d,p) level for structures optimized at the B3LYP/6-311++G(d,p) level. Wavelengths have not been corrected. 40

Figure S_17. UV and ECD spectra of the low-energy conformers of compound 1c calculated at the TD-M06-2X/6-311++G(d,p) level for structures optimized at the B3LYP/6-311++G(d,p) level. Wavelengths have not been corrected. 40

Figure S_18. UV and ECD spectra of the low-energy conformers of compound 1d calculated at the TD-CAM-B3LYP/6-311++G(d,p) level for structures optimized at the B3LYP/6-311++G(d,p) level. Wavelengths have not been corrected. 41

Figure S_19. UV and ECD spectra of the low-energy conformers of compound 1d calculated at the TD-M06-2X/6-311++G(d,p) level for structures optimized at the B3LYP/6-311++G(d,p) level. Wavelengths have not been corrected. 41

Figure S_20. UV and ECD spectra of the low-energy conformers of compound 1e calculated at the TD-CAM-B3LYP/6-311++G(d,p) level for structures optimized at the B3LYP/6-311++G(d,p) level. Wavelengths have not been corrected. 42

Figure S_21. UV and ECD spectra of the low-energy conformers of compound 1e calculated at the TD-M06-2X/6-311++G(d,p) level for structures optimized at the B3LYP/6-311++G(d,p) level. Wavelengths have not been corrected. 42

Figure S_22. UV and ECD spectra of the low-energy conformers of compound 3 calculated at the TD-CAM-B3LYP/6-311++G(d,p) level for structures optimized at the B3LYP/6-311++G(d,p) level. Wavelengths have not been corrected. 43

Figure S_23. UV and ECD spectra of the low-energy conformers of compound 3 calculated at the TD-M06-2X/6-311++G(d,p) level for structures optimized at the B3LYP/6-311++G(d,p) level. Wavelengths have not been corrected. 43

Figure S_24. UV and ECD spectra of the low-energy conformers of compound 4 calculated at the TD-CAM-B3LYP/6-311++G(d,p) level for structures optimized at the B3LYP/6-311++G(d,p) level. Wavelengths have not been corrected. 44

Figure S_25. UV and ECD spectra of the low-energy conformers of compound 4 calculated at the TD-M06-2X/6-311++G(d,p) level for structures optimized at the B3LYP/6-311++G(d,p) level. Wavelengths have not been corrected. 44

Figure S_26. UV and ECD spectra of the low-energy conformers of compound 5 calculated at the TD-CAM-B3LYP/6-311++G(d,p) level for structures optimized at the B3LYP/6-311++G(d,p) level. Wavelengths have not been corrected. 45

Figure S_27. UV and ECD spectra of the low-energy conformers of compound 5 calculated at the TD-M06-2X/6-311++G(d,p) level for structures optimized at the B3LYP/6-311++G(d,p) level. Wavelengths have not been corrected. 45

Figure S_28. UV and ECD spectra of the low-energy conformers of compound 6 calculated at the TD-CAM-B3LYP/6-311++G(d,p) level for structures optimized at the B3LYP/6-311++G(d,p) level. Wavelengths have not been corrected. 46

Figure S_29. UV and ECD spectra of the low-energy conformers of compound 6 calculated at the TD-M06-2X/6-311++G(d,p) level for structures optimized at the B3LYP/6-311++G(d,p) level. Wavelengths have not been corrected. 46

Figure S_30. UV and ECD spectra of the low-energy conformers of compound 7 calculated at the TD-CAM-B3LYP/6-311++G(d,p) level for structures optimized at the B3LYP/6-311++G(d,p) level. Wavelengths have not been corrected. 47

Figure S_31. UV and ECD spectra of the low-energy conformers of compound 7 calculated at the TD-M06-2X/6-311++G(d,p) level for structures optimized at the B3LYP/6-311++G(d,p) level. Wavelengths have not been corrected. 47

Figure S_32. UV and ECD spectra of the low-energy conformers of compound 8 calculated at the TD-CAM-B3LYP/6-311++G(d,p) level for structures optimized at the B3LYP/6-311++G(d,p) level. Wavelengths have not been corrected. 48

Figure S_33. UV and ECD spectra of the low-energy conformers of compound 8 calculated at the TD-M06-2X/6-311++G(d,p) level for structures optimized at the B3LYP/6-311++G(d,p) level. Wavelengths have not been corrected. 48

Figure S_34. UV and ECD spectra of the low-energy conformers of compound 9 calculated at the TD-CAM-B3LYP/6-311++G(d,p) level for structures optimized at the B3LYP/6-311G(d,p) level. Wavelengths have not been corrected. 49

Figure S_35. UV and ECD spectra of the low-energy conformers of compound 9 calculated at the TD-M06-2X/6-311++G(d,p) level for structures optimized at the B3LYP/6-311G(d,p) level. Wavelengths have not been corrected. 49

Figure S_36. UV and ECD spectra of the low-energy conformers of compound 11 calculated at the TD-CAM-B3LYP/6-311++G(d,p) level for structures optimized at the B3LYP/6-311++G(d,p) level. Wavelengths have not been corrected. 50

Figure S_37. UV and ECD spectra of the low-energy conformers of compound 11 calculated at the TD-M06-2X/6-311++G(d,p) level for structures optimized at the B3LYP/6-311++G(d,p) level. Wavelengths have not been corrected. 50

Figure S_38. UV (upper panel) and ECD (lower panel) spectra of 1a, measured in cyclohexane (solid black lines), calculated at the TD-CAM-B3LYP/6-311++G(d,p) level and *ΔE*-based (solid red lines) and *ΔΔG*-based (solid red lines) Boltzmann averaged. Wavelengths have been corrected to match experimental UV maximum. 51

Figure S_39. UV (upper panel) and ECD (lower panel) spectra of 1a, measured in cyclohexane (solid black lines), calculated at the TD-M06-2X/6-311++G(d,p) level and *ΔE*-based (solid red lines) and *ΔΔG*-based (solid red lines) Boltzmann averaged. Wavelengths have been corrected to match experimental UV maximum. 52

Figure S_40. UV (upper panel) and ECD (lower panel) spectra of 1b, measured in cyclohexane (solid black lines), calculated at the TD-CAM-B3LYP/6-311++G(d,p) level and *ΔE*-based (solid red lines) and *ΔΔG*-based (solid red lines) Boltzmann averaged. Wavelengths have been corrected to match experimental UV maximum. 53

Figure S_41. UV (upper panel) and ECD (lower panel) spectra of 1b, measured in cyclohexane (solid black lines), calculated at the TD-M06-2X/6-311++G(d,p) level and *ΔE*-based (solid red lines) and *ΔΔG*-based (solid red lines) Boltzmann averaged. Wavelengths have been corrected to match experimental UV maximum. 54

Figure S_42. UV (upper panel) and ECD (lower panel) spectra of 1c, measured in cyclohexane (solid black lines), calculated at the TD-CAM-B3LYP/6-311++G(d,p) level and *ΔE*-based (solid red lines) and *ΔΔG*-based (solid red lines) Boltzmann averaged. Wavelengths have been corrected to match experimental UV maximum. 55

Figure S_43. UV (upper panel) and ECD (lower panel) spectra of 1c, measured in cyclohexane (solid black lines), calculated at the TD-M06-2X/6-311++G(d,p) level and *ΔE*-based (solid red lines) and *ΔΔG*-based (solid red lines) Boltzmann averaged. Wavelengths have been corrected to match experimental UV maximum. 56

Figure S_44. UV (upper panel) and ECD (lower panel) spectra of 1d, measured in cyclohexane (solid black lines), calculated at the TD-CAM-B3LYP/6-311++G(d,p) level and *ΔE*-based (solid red lines) and *ΔΔG*-based (solid red lines) Boltzmann averaged. Wavelengths have been corrected to match experimental UV maximum. 57

Figure S_45. UV (upper panel) and ECD (lower panel) spectra of 1d, measured in cyclohexane (solid black lines), calculated at the TD-M06-2X/6-311++G(d,p) level and *ΔE*-based (solid red lines) and *ΔΔG*-based (solid red lines) Boltzmann averaged. Wavelengths have been corrected to match experimental UV maximum. 58

Figure S_46. UV (upper panel) and ECD (lower panel) spectra of 1e, measured in cyclohexane (solid black lines), calculated at the TD-CAM-B3LYP/6-311++G(d,p) level and *ΔE*-based (solid red lines) and *ΔΔG*-based (solid red lines) Boltzmann averaged. Wavelengths have been corrected to match experimental UV maximum........ 59

Figure S_47. UV (upper panel) and ECD (lower panel) spectra of 1e, measured in cyclohexane (solid black lines), calculated at the TD-M06-2X/6-311++G(d,p) level and *ΔE*-based (solid red lines) and *ΔΔG*-based (solid red lines) Boltzmann averaged. Wavelengths have been corrected to match experimental UV maximum........ 60

Figure S_48. UV (upper panel) and ECD (lower panel) spectra of 3, measured in cyclohexane (solid black lines), calculated at the TD-CAM-B3LYP/6-311++G(d,p) level and *ΔE*-based (solid red lines) and *ΔΔG*-based (solid red lines) Boltzmann averaged. Wavelengths have been corrected to match experimental UV maximum........ 61

Figure S_49. UV (upper panel) and ECD (lower panel) spectra of 3, measured in cyclohexane (solid black lines), calculated at the TD-M06-2X/6-311++G(d,p) level and *ΔE*-based (solid red lines) and *ΔΔG*-based (solid red lines) Boltzmann averaged. Wavelengths have been corrected to match experimental UV maximum........ 62

Figure S_50. UV (upper panel) and ECD (lower panel) spectra of 4, measured in cyclohexane (solid black lines), calculated at the TD-CAM-B3LYP/6-311++G(d,p) level and *ΔE*-based (solid red lines) and *ΔΔG*-based (solid red lines) Boltzmann averaged. Wavelengths have been corrected to match experimental UV maximum........ 63

Figure S_51. UV (upper panel) and ECD (lower panel) spectra of 4, measured in cyclohexane (solid black lines), calculated at the TD-M06-2X/6-311++G(d,p) level and *ΔE*-based (solid red lines) and *ΔΔG*-based (solid red lines) Boltzmann averaged. Wavelengths have been corrected to match experimental UV maximum........ 64

Figure S_52. UV (upper panel) and ECD (lower panel) spectra of 5, measured in cyclohexane (solid black lines), calculated at the TD-CAM-B3LYP/6-311++G(d,p) level and *ΔE*-based (solid red lines) and *ΔΔG*-based (solid red lines) Boltzmann averaged. Wavelengths have been corrected to match experimental UV maximum........ 65

Figure S_53. UV (upper panel) and ECD (lower panel) spectra of 5, measured in cyclohexane (solid black lines), calculated at the TD-M06-2X/6-311++G(d,p) level and *ΔE*-based (solid red lines) and *ΔΔG*-based (solid red lines) Boltzmann averaged. Wavelengths have been corrected to match experimental UV maximum........ 66

Figure S_54. UV (upper panel) and ECD (lower panel) spectra of 6, measured in cyclohexane (solid black lines), calculated at the TD-CAM-B3LYP/6-311++G(d,p) level and *ΔE*-based (solid red lines) and *ΔΔG*-based (solid red lines) Boltzmann averaged. Wavelengths have been corrected to match experimental UV maximum........ 67

Figure S_55. UV (upper panel) and ECD (lower panel) spectra of 6, measured in cyclohexane (solid black lines), calculated at the TD-M06-2X/6-311++G(d,p) level and *ΔE*-based (solid red lines) and *ΔΔG*-based (solid red lines) Boltzmann averaged. Wavelengths have been corrected to match experimental UV maximum........ 68

Figure S_56. UV (upper panel) and ECD (lower panel) spectra of 7, measured in cyclohexane (solid black lines), calculated at the TD-CAM-B3LYP/6-311++G(d,p) level and *ΔE*-based (solid red lines) and

$\Delta\Delta G$-based (solid red lines) Boltzmann averaged. Wavelengths have been corrected to match experimental UV maximum........ 69

Figure S_57. UV (upper panel) and ECD (lower panel) spectra of 7, measured in cyclohexane (solid black lines), calculated at the TD-M06-2X/6-311++G(d,p) level and ΔE-based (solid red lines) and $\Delta\Delta G$-based (solid red lines) Boltzmann averaged. Wavelengths have been corrected to match experimental UV maximum........ 70

Figure S_58. UV (upper panel) and ECD (lower panel) spectra of 8, measured in cyclohexane (solid black lines), calculated at the TD-CAM-B3LYP/6-311++G(d,p) level and ΔE-based (solid red lines) and $\Delta\Delta G$-based (solid red lines) Boltzmann averaged. Wavelengths have been corrected to match experimental UV maximum........ 71

Figure S_59. UV (upper panel) and ECD (lower panel) spectra of 8, measured in cyclohexane (solid black lines), calculated at the TD-M06-2X/6-311++G(d,p) level and ΔE-based (solid red lines) and $\Delta\Delta G$-based (solid red lines) Boltzmann averaged. Wavelengths have been corrected to match experimental UV maximum........ 72

Figure S_60. UV (upper panel) and ECD (lower panel) spectra of 9, measured in cyclohexane (solid black lines), calculated at the TD-CAM-B3LYP/6-311++G(d,p) level and ΔE-based (solid red lines) and $\Delta\Delta G$-based (solid red lines) Boltzmann averaged. Wavelengths have been corrected to match experimental UV maximum........ 73

Figure S_61. UV (upper panel) and ECD (lower panel) spectra of 9, measured in cyclohexane (solid black lines), calculated at the TD-M06-2X/6-311++G(d,p) level and ΔE-based (solid red lines) and $\Delta\Delta G$-based (solid red lines) Boltzmann averaged. Wavelengths have been corrected to match experimental UV maximum........ 74

Figure S_62. UV (upper panel) and ECD (lower panel) spectra of 11, measured in cyclohexane (solid black lines), calculated at the TD-CAM-B3LYP/6-311++G(d,p) level and ΔE-based (solid red lines) and $\Delta\Delta G$-based (solid red lines) Boltzmann averaged. Wavelengths have been corrected to match experimental UV maximum........ 75

Figure S_63. UV (upper panel) and ECD (lower panel) spectra of 11, measured in cyclohexane (solid black lines), calculated at the TD-M06-2X/6-311++G(d,p) level and ΔE-based (solid red lines) and $\Delta\Delta G$-based (solid red lines) Boltzmann averaged. Wavelengths have been corrected to match experimental UV maximum........ 76

Figure S_64. IR (upper panel) and VCD (lower panel) spectra of 9, calculated at the IEFPCM/B3LYP/6-311G(d,p) level of theory. 77

Figure S_65. IR (upper panel) and VCD (lower panel) spectra of 9, measured in CCl_4 (solid black lines) and calculated (blue dashed lines) for geometries optimized at the IEFPCM (CCl_4)/B3LYP/6-311G(d,p) level. The calculated VCD spectra were Boltzmann-averaged based on $\Delta\Delta G$ values and were not scaled........ 78

Copies of ^{1}H and ^{13}C NMR spectra........ 79

Figure S_66. Copy of ^{1}H NMR spectrum of 1a measured in $CDCl_3$. 79

Figure S_67. Copy of 13C NMR spectrum of 1a measured in $CDCl_3$........ 79

Figure S_68. Copy of ^{1}H NMR spectrum of 1b measured in $CDCl_3$. 80

Figure S_69. Copy of ^{13}C NMR spectrum of 1b measured in $CDCl_3$. 80
Figure S_70. Copy of ^{1}H NMR spectrum of 1c measured in $CDCl_3$. 81
Figure S_71. Copy of ^{13}C NMR spectrum of 1c measured in $CDCl_3$. 81
Figure S_72. Copy of ^{1}H NMR spectrum of 1d measured in $CDCl_3$. 82
Figure S_73. Copy of ^{13}C NMR spectrum of 1d measured in $CDCl_3$. 82
Figure S_74. Copy of ^{1}H NMR spectrum of 1e measured in $CDCl_3$. 83
Figure S_75. Copy of ^{13}C NMR spectrum of 1e measured in $CDCl_3$. 83
Figure S_76. Copy of ^{1}H NMR spectrum of alcohol 3 measured in $CDCl_3$. 84
Figure S_77. Copy of ^{13}C NMR spectrum of alcohol 3 measured in $CDCl_3$. 84
Figure S_78. Copy of ^{1}H NMR spectrum of ketone 4 measured in $CDCl_3$. 85
Figure S_79. Copy of ^{13}C NMR spectrum of ketone 4 measured in $CDCl_3$. 85
Figure S_80. Copy of ^{1}H NMR spectrum of alcohol 5 measured in $CDCl_3$. 86
Figure S_81. Copy of ^{13}C NMR spectrum of alcohol 5 measured in $CDCl_3$. 86
Figure S_82. Copy of ^{1}H NMR spectrum of ester 6 measured in $CDCl_3$. 87
Figure S_83. Copy of ^{13}C NMR spectrum of ester 6 measured in $CDCl_3$. 87
Figure S_84. Copy of ^{1}H NMR spectrum of diester 7 measured in $CDCl_3$. 88
Figure S_85. Copy of ^{13}C NMR spectrum of diester 7 measured in $CDCl_3$. 88
Figure S_86. Copy of 1H NMR spectrum of ester 8 measured in $CDCl_3$. 89
Figure S_87. Copy of ^{13}C NMR spectrum of ester 8 measured in $CDCl_3$. 89
Figure S_88. Copy of ^{1}H NMR spectrum of diester 9 measured in $CDCl_3$. 90
Figure S_89. Copy of ^{13}C NMR spectrum of diester 9 measured in $CDCl_3$. 90
Figure S_90. Copy of ^{1}H NMR spectrum of diester 10 measured in $CDCl_3$. 91
Figure S_91. Copy of ^{13}C NMR spectrum of diester 10 measured in $CDCl_3$. 91
Figure S_92. Copy of ^{1}H NMR spectrum of oxirane 11 measured in $CDCl_3$. 92
Figure S_93. Copy of ^{13}C NMR spectrum of oxirane 11 measured in $CDCl_3$. 92
Figure S_94. Copy of ^{1}H NMR spectrum of alcohol 12 measured in $CDCl_3$. 93
Figure S_95. Copy of ^{13}C NMR spectrum of alcohol 12 measured in $CDCl_3$. 93
Cartesian coordinates for all calculated structures 94
Single crystals X-ray analysis 139
Figure S_94. a) The molecular structure of asymmetric unit of compound *rac*-3: a) and polymorph α, b) polymorph *β*. c) Molecular packing (view along b-axis) and d) supramolecular column in crystal structure of *rac*-3- α and e) molecular packing (view along b-axis) and d) supramolecular column in crystal structure of *rac*-3- *β*. Symmetrically independent molecules are indicated with different colors and the O-atoms are shown as balls. 140

Figure S_95. a) The molecular structure of compound *rac*-4 and b) molecular packing (view along c-axis). The cyclic motif *via* hydrogen bonds is indicated with blue colored spacefill model. The O-atoms are shown as balls and hydrogen bonds are shown as dashed lines. 141

Figure S_97. a) Molecular structure of asymmetric unit of compound *rac*-5, b) molecules are disordered and different orientations of the molecule are represented by different colors and O atoms are shown as balls; c) molecular packing in crystal structure (view along a-axis). 143

Figure S_98. a) Molecular structure of asymmetric unit of compound *rac*-7; b) supramolecular chain *via* hydrogen bonds; c) molecular packing in crystal structure (view along b-axis), the solvent accessible space is indicated with bluish-colour surface. Hydrogen bonds are shown as dashed lines and the O atoms are shown as balls. 144

Figure S_99. a) Molecular structure of compound 8; b) supramolecular chain *via* C-H···O interactions; c) molecular packing in crystal structure (view along b-axis), hydrogen bonds are shown as dashed lines and the O atoms are shown as balls and hydrogen atoms are omitted for clarity. 145

Figure S_100. a) Molecular structure of compound 9; b) molecular packing in crystal structure (view along a-axis; The O atoms are shown as balls and hydrogen atoms are omitted for clarity). 145

Figure S_101. a) Molecular structure of asymmetric unit of compound 11, b) molecular ladder-like structural motif *via* hydrogen bonds (hydrogen bonds are shown as dashed lines, the O-atoms are shown as balls) and c) molecular packing in crystal structure (view along a-axis) – supramolecular ladder-like motif is indicated with blue colored spacefill model. 146

Figure S_102. a) Molecular structure of asymmetric unit of compound 12 (hydrogen bonds are shown as dashed lines), and b) molecular packing (view along a-axis) in crystal structure (symmetrically independent molecules are indicated with different colors and the O-atoms are shown as balls). .. 147

Table S_3. Selected crystal data and structure refinement details. 148

Table S_4. Dihedral angles α, β, γ and δ (in degrees) and selected interatomic distances l_1 and l_2 (in Å) observed in the crystal structures 3α, 3β, 4a, 4b, 5, 7, 8, 9, 11 and 12. 149

References 151

Experimental details

^{1}H and ^{13}C NMR spectra were recorded on Bruker Ultrashield 300 MHz or Varian VNMR-S 400 MHz instruments. Chemical shifts (δ) are reported in ppm relative to $SiMe_4$. HR-MS spectra were obtained with a Bruker Impact HD, QTOF MS spectrometer. UV and ECD spectra were recorded in spectroscopic grade cyclohexane using a JASCO J-810 instrument. FT-IR spectra were measured on a Nicolet iS 50 spectrometer using ATR module. A JASCO P-2000 polarimeter was used for optical rotation ($[\alpha]_D$) measurements (carried out at ca. 20 °C). IR and VCD spectra were measured on Jasco FVS-6000 spectrometer, in BaF_2 cell with 50 µm path lengths in dry carbontetrachloride. A total of 7200 scans were taken for each spectrum, which then was subtracted from the solvent.

Column chromatography was performed on J.T. Baker Silica Gel 40µm (chromatography grade). Merck Kieselgel type $60F_{254}$ analytical plates were used for TLC analyses. Melting points were measured on Büchi Melting Point B-545 and uncorrected. All reagents were used as purchased from commercial suppliers. All solvents were provided by a local supplier and were purified by conventional methods prior to use.

Syntheses of racemic alcohols 1a-1e

General procedure for synthesis of racemic alcohols, using **1a** as an example:

Triphenylmethane (1.83 g, 7.5 mmol, 1 eq) was dissolved in 20 mL of anhydrous THF, and solution was chilled to -30°C. To vigorously stirred solution a 3.3 mL of hexane solution (2.5M) of *n*-BuLi (8.3 mmol, 1.1 eq) was added dropwise. Resulting solution had deep red colour. Reaction mixture was then stirred for 45 minutes, then let to achieve room temperature, and stirred further for 1 hour. After this time mixture was chilled to -78°C. To the reaction mixture acetaldehyde (661 mg, 15 mmol, 2 eq.) solution in THF (5 mL) was very slowly introduced. After addition of prepared solution, the mixture was stirred for 40 minutes in -78°C. Subsequently, mixture was heated until it achieved 5°C, and saturated solution of ammonium chloride was introduced. Mixture was then stirred for next 15 minutes. Organic phase was collected, inorganic was extracted three times with diethyl ether. Obtained organic extracts were dried over magnesium sulfate, filtered and evaporated to dryness. Crude reaction mixture was purified using column chromatography utilizing hexane:diethyl ether as an eluent.

Syntheses of ketones 2a-2e

Oxidation procedure for alcohols except **1c** (synthesis of **2a** as an example of general procedure)

Chromium trioxide (295 mg, 1.94 mmol, 1.5 eq) was dissolved in distilled water (1.5 mL) and chilled to 0°C. Concentrated sulfuric acid (0.14 mL) was added dropwise. Oxidized alcohol was dissolved (374 mg, 1.30 mmol, 1 eq) in acetone (7 mL) and chilled to 0°C, then oxidizing mixture was added dropwise. Reaction was monitored using TLC. In case of any solid precipitated - it was filtered, washed three times with cold distilled water and dried; otherwise the reaction after completion was quenched with saturated sodium bicarbonate solution. Resulting solution was extracted with dichloromethane three times. Organic extracts were collected, dried using anhydrous sodium sulfate

and evaporated to dryness using rotary evaporator. Purification was conducted using column chromatography with hexane:ethyl acetate (8:1, v/v) mixture as an eluent.

Oxidation procedure for alcohol **1c**

Mixture of PCC (682 mg, 3.16 mmol, 2 eq) and sodium acetate (324 mg, 3.95 mmol, 2.5 eq) in dichloromethane (10 mL) was prepared. Oxidized alcohol (500 mg, 1.58 mmol, 1 eq) was dissolved in dichloromethane (10 mL) and poured in one portion to oxidizing mixture. Reaction was monitored using TLC. After complete conversion of substrate brown solid was filtered and washed three times with dichloromethane. Organic phase was washed with 5% sodium hydroxide aqueous solution, distilled water and brine. After that organic extracts were dried using anhydrous sodium sulfate and concentrated using rotary evaporator. Crude product was purified using column chromatography with hexane:diethyl ether (50:1, v/v) mixture as an eluent.

Asymetric reductions of ketones 2a-2e

General procedure exemplified by reduction of **2a**

Ketone (150 mg, 0.52 mmol, 1 eq.) was dissolved in anhydrous toluene (4.5 mL). Solution was stirred and heated to 80°C in argon atmosphere. Borane in THF solution (1M) (0.52 mL, 0.52 mmol, 1 eq.), (*R*)-5,5-diphenyl-2-methyl-3,4-propano-1,3,2-oxazaborolidine in toluene solution (1M) (0.105 mL, 0.105 mmol, 0.2 eq) were mixed in anhydrous toluene (2 mL) and added dropwise to heated solution of ketone. After addition of whole reducing solution reaction mixture was heated to boiling point. Reaction was monitored by thin-layer chromatography and conducted from 1h to two days depending on substrate. After the full substrate conversion reaction was quenched by chilling it to room temperature and subsequent addition of methanol. Mixture was evaporated to obtain residual oil, which was dissolved in dichloromethane and washed with 2M hydrochloric acid aqueous solution, water, saturated aqueous sodium bicarbonate solution and brine. Organic extracts were dried using anhydrous sodium sulfate and evaporated to dryness. Crude product was purified by column chromatography using hexane:diethyl ether or hexane:ethyl acetate mixture as an eluent.

1a

e.e. = 71 %

^{1}H NMR (600 MHz, $CDCl_3$) δ 7.34 (d, 6H, J = 7.8 Hz), 7.27 (dd, 7H, J = 14.2, 6.5 Hz), 7.20 (t, 2H, J = 7.4 Hz,), 5.48 (qt, 1H, J = 6.3 Hz), 1.47 (d, 1H, 6.6 Hz), 1.12 (d, 3H, J = 6.1 Hz);

^{13}C NMR (151 MHz, $CDCl_3$) δ 130, 127.9, 126.2, 69.7, 62.6, 19.9;

ATR-IR 3543, 3438, 3085, 3055, 3031, 2978, 2928, 1596, 1492, 1445, 1393, 1375, 1330, 1262, 1109, 1084, 1050, 1032, 890, 745, 698, 645, 585, 531 cm^{-1};

ESI HRMS *m/z* calcd for $C_{21}H_{20}ONa$ $[M+H]^+$: 311.1412, found 311.1405.

$[\alpha]_D$ = +9 (c = 0.51, $CHCl_3$).

1b

e.e. = 83 %

^{1}H NMR (600 MHz, $CDCl_3$) *δ* 7.35 (m, 6H), 7.32 – 7.28 (m, 7H), 7.24-7.20 (m, 2H), 5.18 (m, 1H), 1.70 (q, 2H, *J* = 10.7 Hz), 1.60 – 1.52 (m, 2H), 1.41 (d, 1H, *J* = 6.8 Hz), 0.94 (t, 3H, *J* = 6.8 Hz);

^{13}C NMR (151 MHz, $CDCl_3$) *δ* 129.9, 127.9, 126.1, 74.2, 62.5, 36.4, 20.4, 14.2;

ATR-IR 3567, 3466, 3086, 3055, 3031, 2955, 2928, 2869, 1711, 1596, 1493, 1463, 1444, 1398, 1377, 1330, 1273, 1219, 1118, 1086, 1065, 1033, 1010, 745, 698, 652, 635, 612 cm^{-1};

ESI HRMS *m/z* calcd for $C_{23}H_{24}ONa$ $[M+H]^+$: 339.1725, found 339.1713.

$[\alpha]_D$ = +37.9 (c = 0.5, $CHCl_3$).

1c

e.e. = 66 %

^{1}H NMR (600 MHz, $CDCl_3$) *δ* 7.42 (d, *J* = 7.7 Hz, 6H), 7.32 – 7.27 (m, 7H), 7.21 (t, *J* = 7.1 Hz, 2H), 5.23 (d, 1H, *J* = 5.7 Hz), 2.03 – 1.96 (m, 1H), 1.55 (d, 2H, *J* = 11.9 Hz), 1.20 (d, 3H, *J* = 6.8 Hz), -0.04 (d, 3H, *J* = 6.6 Hz).

^{13}C NMR (151 MHz, $CDCl_3$) *δ*129.8, 127.9, 126.1, 76.5, 63, 29.3, 24.2, 15.5;

ATR-IR 3578, 3477, 3089, 3055, 3032, 2954, 2926, 2870, 1710, 1596, 1493, 1477, 1445, 1384, 1362, 1245, 1169, 1127, 1087, 1033, 1007, 758, 744, 698, 657, 636, 595 cm^{-1};

ESI HRMS *m/z* calcd for $C_{23}H_{24}ONa$ $[M+H]^+$: 339.1725, found 339.1716.

$[\alpha]_D$ = +54.2 (c = 0.61, $CHCl_3$).

1d

e.e. = 79 %

^{1}H NMR (600 MHz, $CDCl_3$) *δ* 7.36 (s, 6H), 7.33 – 7.28 (m, 7H), 7.22 (s, 2H), 5.37 – 5.32 (m, 1H), 2.00 – 1.90 (m, 1H), 1.51 (dd, 1H, *J* = 14.1, 9.9 Hz), 1.31 (d, 1H, *J* = 7.4 Hz), 1.11 (d, 3H, *J* = 6.5 Hz), 0.89 (d, 3H, *J* = 6.7 Hz), 0.83 – 0.75 (m, 1H);

^{13}C NMR (151 MHz, $CDCl_3$) *δ* 129.88, 127.82, 126.12, 72.0, 62.5, 42.9, 25.5, 24.0, 21.9;

ATR-IR 3566, 3055, 3032, 2952, 2866, 1723, 1675, 1655, 1493, 1465, 1444, 1384, 1364, 1320, 1263, 1219, 1169, 1130, 1110, 1061, 1034, 1017, 973, 929, 896, 845, 822, 802, 760, 743, 698, 653, 635 614 cm^{-1};

ESI HRMS *m/z* calcd for $C_{24}H_{26}ONa$ $[M+H]^+$: 353.1881, found 353.1874.

$[\alpha]_D$ = +6 (c = 0.5, $CHCl_3$).

1e

e.e. = 68 %

Mp. 121-124 °C

^1H NMR (600 MHz, $CDCl_3$) δ 7.39 (d, 6H, *J* = 7.7 Hz), 7.26 (t, 7H, *J* = 7.8 Hz), 7.18 (t, 3H, *J* = 7.4 Hz), 5.11 (d, 1H, *J* = 6.3 Hz), 1.90 (d, 1H, *J* = 10.4 Hz), 1.73 (d, 1H, *J* = 13.3 Hz), 1.50 (d, 1H, *J* = 13.0 Hz), 1.31 (d, 1H, *J* = 13.4 Hz), 1.21 (td, 1H, *J* = 13.3, 12.7, 4.2 Hz), 1.10 – 0.98 (m, 1H), 0.76 (q, 1H, *J* = 13.1 Hz), 0.63 (q, 1H, *J* = 12.0, 10.9 Hz), -0.22 (d, 1H, *J* = 12.7 Hz).

^{13}C NMR (151 MHz, $CDCl_3$) δ 129.8, 127.9, 126.1 63.2, 40.0, 34.5, 29.7, 27.2, 26.7, 26.4, 26.2;

ATR-IR 3570, 3477, 3088, 3059, 3034, 2922, 2850, 1594, 1493, 1444, 1269, 1235, 1159, 1102, 1034, 996, 893, 762, 744, 703, 652, 634, 600 cm^{-1};

ESI HRMS *m/z* calcd for $C_{26}H_{28}ONa$ $[M+H]^+$: 379.2038, found 379.2021.

$[\alpha]_D$ = +46.8 (c = 0.56, $CHCl_3$).

2a

Mp. 130-134 °C

^1H NMR (300 MHz, $CDCl_3$) δ 7.44 – 7.17 (m, 15H), 2.10 (s, 3H);

^{13}C NMR (75 MHz, $CDCl_3$) δ 206, 142.3, 130.4, 128.1, 126.8, 73.1, 29.8, 29.7;

ATR-IR 3079, 3057, 3017, 2981, 2924, 1739, 1710, 1597, 1491, 1444, 1348, 1265, 1209, 1185, 1151, 1081, 1035, 1001, 882, 746, 696, 638, 582, 565, 487 cm^{-1};

ESI HRMS *m/z* calcd for $C_{21}H_{18}ONa$ $[M+H]^+$: 309.1255, found 309.1249.

2b

Mp. 108-110 °C

^1H NMR (300 MHz, $CDCl_3$) δ 7.35 – 7.17 (m, 15H), 2.41 – 2.29 (m, 2H), 1.49 – 1.30 (m, 2H), 0.71 (t, 3H, *J* = 7.4 Hz);

^{13}C NMR (75 MHz, $CDCl_3$) δ 208.6, 142.4, 130.5, 128.1, 126.7, 77.5, 77.1, 76.6, 73.2, 43.7, 19.2, 13.8;

ATR-IR 3061, 3017, 2963, 2928, 2871, 1708, 1595, 1491, 1462, 1442, 1407, 1376, 1356, 1279, 1184, 1119, 1084, 1057, 1034, 1001, 756, 725, 698, 665, 628, 541, 496 cm^{-1};

ESI HRMS *m/z* calcd for $C_{23}H_{22}ONa$ $[M+H]^+$: 337.1568, found 337.1565 .

2c

Mp. 55-57 °C

^{1}H NMR (300 MHz, $CDCl_3$) δ 7.47 – 7.13 (m, 15H), 3.03 (hept, 1H, *J* = 6.5 Hz), 0.75 (d, 6H, *J* = 6.6 Hz);

^{13}C NMR (75 MHz, $CDCl_3$) δ 213.2, 142.4, 130.6, 128.1, 127.4, 126.7, 74.3, 38.5 22;

ATR-IR 3084, 3058, 3029, 2974, 2935, 2874, 1702, 1595, 1491, 1463, 1442, 1378, 1361, 1337, 1274, 1188, 1159, 1102, 1086, 1050, 1035, 1000, 890, 769, 744, 699, 659, 629, 601, 489 cm^{-1};

ESI HRMS *m/z* calcd for $C_{23}H_{22}ONa$ $[M+H]^+$: 337.1568, found 337.1564 .

2d

Mp. 71-74 °C

^{1}H NMR (300 MHz, $CDCl_3$) δ 7.38 – 7.17 (m, 15H), 2.36 (d, 2H, *J* = 6.7 Hz), 1.84 (dp, 1H, *J* = 13.3, 6.7 Hz), 0.68 (d, 6H, *J* = 6.7 Hz);

^{13}C NMR (75 MHz, $CDCl_3$) δ 207.6, 142.5, 130.5, 128.1, 126.7, 73.4, 49.7, 25.4, 22.5;

ATR-IR 3054, 3029, 2972, 2954, 2922, 2868, 1743, 1707, 1595, 1491, 1464, 1446, 1408, 1381, 1365, 1352, 1333, 1281, 1138, 1090, 1063, 1032, 733, 699, 673, 622 cm^{-1};

ESI HRMS *m/z* calcd for $C_{24}H_{24}ONa$ $[M+H]^+$: 351.1725, found 351.1729.

2e

Mp. 102-104 °C

^{1}H NMR (300 MHz, $CDCl_3$) δ 7.47 – 7.11 (m, 15H), 2.72 (tt, 1H, *J* = 11.4, 2.9 Hz), 1.54 (t, 3H, *J* = 6.6 Hz), 1.33 (ddd, 2H, *J* = 15.7, 13.6, 2.9 Hz), 1.20 – 0.84 (m, 5H);

^{13}C NMR (75 MHz, $CDCl_3$) δ 211.7, 142.4, 130.5, 128.1, 126.7, 74.4, 49.2, 31.7, 25.8, 25.7;

ATR-IR 3062, 3030, 2964, 2923, 2856, 1745, 1700, 1594, 1491, 1444, 1360, 1301, 1238, 1140, 1088, 1065, 1032, 999, 740, 707, 696, 671, 631, 619, 601 cm^{-1};

ESI HRMS *m/z* calcd for $C_{26}H_{26}ONa$ $[M+H]^+$: 377.1881, found 377.1564.

(*rac*)-*trans*-2-Tritylcyclohexan-1-ol ((*rac*)-3)

The title compound was obtained according to procedure previously described.[1]

Mp. 168–170 °C

^{1}H NMR (400 MHz, $CDCl_3$) δ 7.52-7.05 (m, 15H), 3.29 (t, 1H, 10.0 Hz), 3.11 (td, 1H, 10.0, 3.3 Hz), 2.02 (d, 1H, 11.0 Hz), 1.91 (d, 1H, 11.3 Hz), 1.77-1.63 (m, 1H), 1.60-1.49 (m, 2H), 1.55-1.44 (m, 1H), 1.41-1.32 (m, 1H), 1.18-0.97 (m, 1H), 0.5 (td, 1H, 13.5, 2.3 Hz);

^{13}C NMR (101 MHz, $CDCl_3$) δ 129.6, 127.7, 125.8,73.6, 60.7, 48.8, 37, 28.9, 26.2, 25.3;

ATR-IR 3558, 3462, 3086, 3053, 3033, 2946, 2919, 2845, 1980, 1594, 1490, 1445, 710 cm^{-1}.

(1*S*,2*R*)-*trans*-2-Tritylcyclohexan-1-ol (3)

The title compound was obtained according to procedure previously described.[1] All spectral data are in accordance to (*rac*)-*trans*-2-tritylcyclohexan-1-ol.

ESI HRMS *m/z* calcd for $C_{25}H_{26}NaO$ $[M+H]^+$: 365.1881, found 365.1873.

e.e. = 45.7 %

(*rac*)-2-Tritylcyclohexan-1-one ((*rac*)-4)

In a flask were placed (1 g, 2.9 mmol) (*rac*)-2-tritylcyclohexanol, anhydrous methylene chloride (20 mL), Celit (1.3 g) and PCC (1.17 g, 5.4 mmol). The mixture was stirred vigorously for 24 hours in argon atmosphere in room temperature. After completion of reaction diethyl ether (20 mL) was added, and the solution was filtered and washed three times with methylene chloride (3 x 10 mL). The combined organic solutions were evaporated on vacum and the residue was separated with silica gel column chromatography (methylene chloride:hexane, 1:1) to give the product as 0.97 g of a white solid, 97.5 %.

Mp. 160-167 °C

^{1}H NMR (400 MHz, $CDCl_3$) δ 7.32-7.11 (m, 15H), 4.32 (dd, 1H, 12.9, 3.6 Hz), 2.54 (td, 1H, 13.1, 6.2 Hz), 2.34-2,21 (m, 2H), 2.12-2.02 (m, 1H) 1.93-1.84 (m, 2H), 1.6-1.5 (m, 1H), 1.1-1.0 (m, 1H);

^{13}C NMR (101 MHz, $CDCl_3$) δ 209, 129.3, 127.5, 125.7, 58.4, 56.6, 44.4, 32.6, 28, 26.2;

ATR-IR 3052, 3033, 3019, 2936, 2867, 1708, 1596, 1495, 1446, 1128, 1034, 749, 709, 698, 539 cm^{-1}.

(*R*)-2-Tritylcyclohexan-1-one (4)

In a flask were placed (1 g, 2.9 mmol) (1*R*,2*S*)-2-tritylcyclohexanol, anhydrous methylene chloride (20 mL), Celit (1.3 g) and PCC (1.26 g, 5.85 mmol). The mixture was stirred vigorously for 24 hours in argon atmosphere in room temperature. After completion of reaction diethyl ether (20 mL) was added. After stirring, the solution was filtered and washed three times with methylene chloride (3 x 10 mL). The combined organic solutions were evaporated on vacum and the residue was separated with silica gel column chromatography (methylene chloride:hexane, 1:1) to give the product as 0.82 g of a white solid, 83 %.

e.e. = 79.6 %

Mp. 164-167 °C

^{1}H NMR (400 MHz, $CDCl_3$) δ 7.32-7.11 (m, 15H), 4.32 (dd, 1H, 12.9, 3.6 Hz), 2.54 (td, 1H, 13.1, 6.2 Hz), 2.34-2,21 (m, 2H), 2.12-2.02 (m, 1H) 1.93-1.84 (m, 2H), 1.6-1.5 (m, 1H), 1.1-1.0 (m, 1H);

^{13}C NMR (101 MHz, $CDCl_3$) δ 209, 129.3, 127.5, 125.7, 58.4, 56.6, 44.4, 32.6, 28, 26.2;

ATR-IR 3052, 3033, 3019, 2936, 2867, 1708, 1596, 1495, 1446, 1128, 1034, 749, 709, 698, 539 cm^{-1};

ESI HRMS *m/z* calcd for $C_{25}H_{24}NaO$ $[M+H]^+$: 363.169, found: 363.1718;

$[\alpha]_D$ = +38 (c = 1.00, $CHCl_3$).

(1*R*,2*R*)-*cis*-2-Tritylcyclohexan-1-ol (5)

To a flask under argon was added (*R*)-2-triphenylmethylcyclohexan-1-one **4** (0.3 g, 0.88 mmol) in anhydrous THF (10 mL). This solution was cooled to -78°C. LS-Selectride (Aldrich Chemical Co.) (1M in THF, 1.32 mL, 1.32 mmol) was added and mixture was stirred for 2 hours at -78 ˚C and 18 hours at room temperature. After that was washed with water (5 mL) and reaction mixture was extracted with ethyl acetate (2 X 25 mL). The solvent was removed in vacuo and the residue was separated with silica gel column chromatography (methylene chloride:hexane, 1:1) to give the product as 0.24 g of a white solid, 60 %.

e.e. = 49.2 %

Mp. 30-34 °C

^{1}H NMR (400 MHz, $CDCl_3$) δ 7.4-7.18 (m, 15H), 4.49 (s, 1H), 3.32 – 3.25 (m, 1H), 1.9-1.8 (m, 2H), 1.75-1.5 (m, 5H), 0.21 (s, 1H);

^{13}C NMR (101 MHz, $CDCl_3$) δ 146, 129.4, 128, 126, 66.8, 60.4, 47.2, 34.3, 27.6, 22, 20;

ATR-IR 3597, 3475, 3086, 3054, 3032, 2925, 2853, 1594, 1492, 1445, 1035, 968, 756, 741, 718, 704, 656 cm^{-1};

ESI HRMS *m/z* calcd for $C_{25}H_{26}NaO$ $[M+H]^+$:365.1881, found 365.1879;

$[\alpha]_D$ = +25 (c = 1.07, $CHCl_3$).

(1*S*,2*R*)-2-Tritylcyclohexyl acetate (6)

(-)-(1*S*,2*R*)- Tritylcyclohexan-1-ol **3** (0.27 g, 0.8 mmol) was dissolved in methylene chloride (5 mL) under argon atmosphere. Next were added DMAP (0.122 g, 1 mmol), NEt_3 (0.16 mL, 1.15 mmol) and dropped acetyl chloride (0.08 mL, 1.15 mmol). The mixture was stirred at room temperature for 20 hours and after that washed with water (5 mL). The inorganic layer was extracted with methylene chloride (3 × 15 mL). The combined organic layers were dried over anhydrous Na_2SO_4. The solvent was removed in vacuo and the residue was separated with silica gel column chromatography (methylene chloride:hexane, 1:1) to give the product as 0.095 g of a white solid, 30 %.

Mp. 63−66 °C

^{1}H NMR (400 MHz, $CDCl_3$) δ 7.5-7.05 (m, 15H), 4.14 (td, 1H, 10.4, 4.0 Hz), 3.54-3.39 (m, 1H), 2.09 (d, 1H, 13.8 Hz), 1.98 (d, 1H, 9.9 Hz) 1.7-1.58 (m, 2H), 1.5-1.35 (m, 2H), 1.38 (s, 3H), 1.2-1.1 (m, 1H), 0.6 (m, 1H);

^{13}C NMR (101 MHz, $CDCl_3$) δ 170, 129.1, 127.3, 125.2, 75.1, 60.8, 46.2, 33.4, 29, 26, 24.8, 20.6;

ATR-IR 3087, 3054, 3032, 2938, 2858, 1730, 1491, 1446, 1363, 1239, 1033, 705 cm^{-1};

ESI HRMS *m/z* calcd for $C_{27}H_{28}NaO_2$ $[M+H]^+$: 407.1987, found: 407.1979;

$[\alpha]_D$ = -39.8 (c = 1.01, $CHCl_3$).

Menthyl ester 7

The title compound was obtained according to procedure previously described.[1]

d.r. = 89.8 %

Mp. 183−186 °C

^{1}H NMR (300 MHz, $CDCl_3$) δ 7,41-7,05 (m, 15H), 4,68-4.56 (m, 1H), 4.19-4.07 (m, 1H), 3.57 (t, 1H, 10.6 Hz), 2.10 (d, 1H, 13.5 Hz), 1.97 (d, 1H, 12.4 Hz), 1.9-1.8 (m, 1H), 1.8-1.6 (m, 6H), 1.5-1.15 (m, 4H), 1.1-0.97 (m, 1H), 0.93-0.8 (m, 9H), 0.71 (dd, 3H, 13.0, 6.9 Hz);

^{13}C NMR (101 MHz, $CDCl_3$) δ 157.3, 129.6, 127.4, 125.6, 78.6, 77.2, 60.8, 46.3, 40, 34, 32.6, 31.4, 28.9, 26.2, 26, 24.6, 23.5, 22, 20.7, 16.4;

ATR-IR 3057, 2947, 2868, 1759, 1732, 1491, 1446, 1315, 1175, 1152, 1125, 708, 695 cm^{-1};

ESI HRMS *m/z* calcd for $C_{37}H_{44}NaO_4$ $[M+H]^+$: 575.3137, found: 575.3124;

$[\alpha]_D$ = +38.8 (c = 1.08, $CHCl_3$).

2-Oxo-2-(((1*R*,2*S*)-2-tritylcyclohexyl)oxy)acetic acid (8)

(-)-(1*S*,2*R*)-Triphencyclohexanol (0.16 g, 0.5 mmol) was dissolved in freshly distilled methylene chloride (3 mL) and then oxalyl chloride was added (0.016 mL, 0.19 mmol). The mixture was heated at the boiling point of the solvent for 20 hours. After this time, the flask was cooled to room temperature and mixture was washed with a saturated Na_2CO_3 (2 × 5 mL) and dried over anhydrous Na_2SO_4. The solvent was removed in vacuo and the residue was separated with silica gel column chromatography (methylene chloride:hexane, 1:1) to give the product as 0.116 g of a white solid, 35%.

Mp. 82-87 °C

^{1}H NMR (300 MHz, $CDCl_3$) δ 7.45-7.08 (m, 15H),5.32 (s, 1H), 4.31 (td, 1H, 10.4, 3.9 Hz), 3.7-3.61 (m, 1H), 2.25-2.16 (m, 1H), 2.05 (m, 1H, 18.5 Hz), 1.83-1.62 (m, 3H), 1.53-1.4 (m, 1H), 1.3-1.2 (m, 1H), 0.86-0.75 (m, 1H);

^{13}C NMR (101 MHz, $CDCl_3$) δ 156.2, 129.5, 127.7, 126.1, 80.4, 60.7, 46.3, 32.7, 29, 25.8, 24.7;

ATR-IR 3409, 3087, 3056, 3032, 2927, 2860, 1736, 1492, 1446, 1197, 1060, 1034, 705 cm^{-1};

ESI HRMS *m/z* calcd for $C_{27}H_{26}NaO_4$ $[M+H]^+$: 437.1729, found: 437.1719 ;

$[\alpha]_D$ = +2.6 (c = 1.01, $CHCl_3$).

Bis((1*R*,2*S*)-2-tritylcyclohexyl) oxalate (9)

Under argon atmosphere to the flask were added: freshly distilled THF (6 mL), (-)-(1*S*,2*R*)-triphencyclohexanol (0.4 g, 1.17 mmol), DMAP (0.0036 g, 0.03 mmol) and pyridine (0.1 mL, 1.24 mmol). Mixture was cooled to 0 ˚C and was added oxalyl chloride (0.016 mL, 0.19 mmol). The reaction was mixing for 20 h. After this time was washed with water (1 × 10 mL), extracted by diethyl ether (3 × 20 mL), washed with sodium hydrogen carbonate (1 × 10 mL), water (1 × 10 mL) and sat. brine (1 × 10 mL) and dried over anhydrous Na_2SO_4. The solvent was removed in vacuo and the residue was separated with silica gel column chromatography (hexane) to give the product as 0.32 g of a white solid, 41%.

Mp. 212-218 °C

^{1}H NMR (400 MHz, $CDCl_3$) δ 7.4-7.0 (m, 30H), 3.93 (td, 2H, 10.3, 3.5 Hz), 3.55-3.44 (m, 2H), 2.08 (d, 2H, 13.6 Hz), 1.72-1.57 (m, 6H), 1.42-1.24 (m, 4H), 1.22-1.00 (m, 2H), 0.83 (q, 2H, 11.4);

^{13}C NMR (101 MHz, $CDCl_3$) δ 157, 129.5, 127.5, 125.5, 78.3, 60.8, 46.2, 32.6, 29, 26, 24.7;

ATR-IR 3086, 3052, 3034, 2957, 2926, 2849, 1754, 1730, 1595, 1492, 1447, 1316, 1166, 1121, 1032, 979, 913, 721, 704, 692 cm^{-1};

ESI HRMS *m/z* calcd for $C_{52}H_{50}NaO_4$ $[M+H]^+$: 761.3607, found 761.3588;

$[\alpha]_D$ = -29.8 (c = 1.04, $CHCl_3$).

Bis((1*S*,2*R*)-2-tritylcyclohexyl) adipinate (10)

To a round bottomed flask with a reflux and drying tube was added adipic acid (1.08 g, 7.4 mmol). Thionyl chloride (2 mL, 27.6 mmol) was added to the flask and the system warmed at reflux for 15 hours. The residue was cooled and evaporated excess thionyl chloride, washed three times with hexane to give the product as 0.9 mL of liquid, 71.3%.

(-)-(1*S*,2*R*)-Triphencyclohexanol (0.2 g, 0.585 mmol) was dissolved in freshly distilled methylene chloride (5 mL) and then adipoyl dichloride was added (0.04 mL, 0.23 mmol). The mixture was heated at the boiling point of the solvent for 20 h. After this time, the flask was cooled to rt and mixture was washed with a saturated sodium bicarbonate solution (2 × 5 mL) and dried over anhydrous Na_2SO_4. The solvent was removed in vacuo and the residue was separated with silica gel column chromatography (methylene chloride:hexane, 1:1) to give the product as 0.116 g of a white solid, 32%.

Mp. 71-95 °C

^{1}H NMR (300 MHz, $CDCl_3$) δ 7.9-7.6 (m, 30H), 4.13 (td, 2H, 10.3, 3.8 Hz), 3.48 (t, 2H,9.9 Hz), 2.09 (d, 2H, 13.6 Hz), 1.97 (m, 2H), 1.66 (m, 4H), 1.54 (m, 2H), 1.48-1.27 (m, 6H), 1.12 (m, 6H), 0.6 (q, 2H, 11.4 Hz);

^{13}C NMR (101 MHz, $CDCl_3$) δ 172.2, 129, 127.3, 125.3, 75, 60.8, 46.2, 33.7, 33.4, 29, 26, 24.8, 24;

ATR-IR 3087, 3055, 3033, 2937, 2858, 1725, 1491, 1446, 1175, 1033, 704 cm^{-1};

ESI HRMS *m/z* calcd for $C_{56}H_{58}NaO_4$ $[M+H]^+$: 817.4233, found 817.4240;

$[\alpha]_D$ = -8.9 (c = 1.01, $CHCl_3$).

(*R*)-2-(2,2,2-Triphenylethyl)oxirane (11)

The title compound was obtained according to procedure previously described.[2]

Mp. 126–127 °C

^{1}H NMR (300 MHz, $CDCl_3$) δ 7.32-7.29 (m, 15H), 3.30 (dd, 1H, 14.3, 3.7 Hz), 2.90 (m, 1H), 2.49 (t, 1H, 4.5 Hz, 2.41 (dd, 1H, 14.3, 6.8 Hz), 2.13 (dd, 1H, 4.9, 2.7 Hz);

^{13}C NMR (101 MHz, $CDCl_3$) δ 146.8, 129, 128, 126.2, 55.8, 50.3, 48.9, 43.7;

ATR-IR 3085, 3053, 3035, 2998, 2962, 2917, 2868, 1595, 1488, 1442, 1406, 1197, 1001, 922, 839, 755, 700, 639, 606 cm^{-1};

ESI HRMS *m/z* calcd for $C_{22}H_{20}NaO$ $[M+H]^+$: 323.1334, found 323.1408;

$[\alpha]_D$ = +22.5 (c = 1.01, $CHCl_3$).

1,1,1,5,5,5-Hexaphenylpentan-3-ol (12)

Triphenylmethane (0.632 g, 2.6 mmol) was dissolved in anhydrous THF (8 mL) under argon atmosphere and cooled with liquid nitrogen to -78 ˚C. After cooling was added dropwise 1.76 mL of hexane solution (2.5 M) of *n*-butyllithium (2.8 mmol) and stirred for 40 minutes at -78 ˚C and the next hour at room temperature. In the same time to the second flask was dared (*R*)-2-(2,2,2-triphenylethyl)oxirane **11** (0.545 g, 1.9 mmol) and dissolved in anhydrous THF (6 mL) and then cooled to -78 ˚C. To this solution was added dropwise the triphenylmethyl anion from the first flask. The mixture was stirred at rt for 24 hours and after that washed with water (2 mL). The inorganic layer was extracted with diethyl ether (3 × 15 mL). The combined organic layers were dried over anhydrous Na_2SO_4. The solvent was removed in vacuo and the residue was separated with silica gel column chromatography (methylene chloride:hexane, 1:1) to give the product as 0.683 g of a white solid, 66.1%.

Mp. 180-181 °C

^{1}H NMR (300 MHz, $CDCl_3$) δ 7.25-7.08 (m, 30H), 3.31 (m, 1H), 3.08 (dd, 2H, 14.6, 7.9 Hz), 2.43 (dd, 2H, 14.6, 2.7 Hz), 0.55 (d, 1H, 3.2 Hz);

^{13}C NMR (101 MHz, $CDCl_3$) δ 147, 129.2, 127.8, 125.8, 67.4, 56.4, 48.8;

ATR-IR 3587, 3575, 3054, 3030, 2961, 2926, 1594, 1491, 1445, 1077, 1026, 745, 700, 623, 611 cm^{-1};

ESI HRMS *m/z* calcd for $C_{41}H_{36}NaO$ $[M+H]^+$: 567.2664, found 567.2653.

Calculation details

Starting geometries of **1a**-**1e**, **3**-**9**, and **11** were obtained by conformational search with the use of a Scigress[3] software and pre-optimization of all conformers at the B3LYP/6-31G(d) level. The conformational searches were done by systematic changes of all rotatable torsion angles with 30° steps. This allowed to identify the minimum energy structures which were further re-optimized with the use of B3LYP functional[4] in conjunction with the enhanced 6-311++G(d,p) or smaller 6-311G(d,p) basis set.[5] The structures thus obtained were the real minimum energy conformers (no imaginary frequencies were found). The total and free energy values were used to obtain the Boltzmann population of conformers at 298.15 K. For density functional theory calculations, only the results for conformers that differ from the most stable one by less than 2 kcal mol^{-1} were taken into account, following a generally accepted protocol. Relative energies (unit kcal mol^{-1}) discussed in the main text refer to Gibbs free energies (*ΔΔG*) computed at the B3LYP/6-311++G(d,p) or B3LYP/6-311G(d,p) level of theory.

ECD spectra for all structures optimized at the B3LYP/6-311++G(d,p) or B3LYP/6-311G(d,p) level were calculated employing M06-2X[6] and CAM-B3LYP,[7] hybrid functionals, all in conjunction with 6-311++G(d,p) basis set.**[5]** The calculated ECD spectra were Boltzmann averaged by taking into account conformers ranging from 0 to 2.0 kcal mol^{-1} in relative energies, following a generally accepted protocol.[8] Rotatory strengths were calculated using both length and velocity representations. In the present study, the differences between the length and velocity calculated values of rotatory strengths were quite small, and for this reason, only the velocity representations were further used. The ECD spectra were simulated by overlapping Gaussian functions for each transition, according to the procedure previously described by Harada and Stephens.[9]

The solvent effect on structure and ECD spectra was not taken into account, since the experimental ECD measurements were done in non-polar cyclohexane.

Both functionals gave very similar results and the results obtained with the use of both of them can be discussed. Very careful analysis leads to the conclusion that the M06-2X hybrid functional reproduces experimental ECD spectra of the compounds under study slightly better than the other. Therefore, we limited our discussion to the results obtained with the use of M06-2X functional only.

VCD calculations were performed for compound **9** with the use of IEFPCM/B3LYP/6-311G(d,p) method. The VCD spectra were simulated by overlapping Lorenzian functions for each transition. VCD spectra were calculated with 8 cm^{-1} half-height width with a program resident on the JASCO VCD software package. Additionally, geometry optimization and VCD calculations were performed with the use of IEFPCM/B3LYP-GD3BJ/6-311G(d,p) and IEFPCM/M06-2X/6-311G(d,p) methods. However, the obtained results were not better than these obtained with the use of B3LYP hybrid functional.

Table S_1. Total energies (in Hartree), relative energies (ΔE, $\Delta\Delta G$ in kcal mol^{-1}), percentage populations and number of imaginary frequencies (#ImFq) calculated at the B3LYP/6-311++G(d,p) level for individual conformers of **1a**-**1e**, **3**-**9** and **11**.

Compound[a]	Energy	ΔE	Pop.	$\Delta\Delta G$	Pop	#ImFq
**1a** (conf. 1)	-887.718232	0.00	57	0.11	45	0
**1a** (conf. 72)	-887.717949	0.18	43	0.00	55	0
**1b** (conf. 1)	-966.366073	0.07	42	0.19	38	0
**1b** (conf. 27)	-966.366189	0.00	47	0.00	54	0
**1b** (conf. 40)	-966.363604	1.62	3	1.83	2	0
**1b** (conf. 82)	-966.364512	1.05	8	1.29	6	0
**1c** (conf. 4)	-966.355068	2.54		1.70	3	0
**1c** (conf. 9)	-966.35912	0.00	60	0.01	45	0
**1c** (conf. 24)	-966.356727	1.50	5	1.60	3	0
**1c** (conf. 25)	-966.356974	1.35	6	1.59	3	0
**1c** (conf. 36)	-966.358411	0.44	29	0.00	46	0
**1d** (conf. 2)	-1005.68939	0.01	45	0.01	42	0
**1d** (conf. 20)	-1005.68799	0.89	10	0.61	15	0
**1d** (conf. 25)	-1005.6894	0.00	45	0.00	42	0
**1d** (conf. 26)	-1005.68605	2.11		1.97	1	0
**1e** (conf. 1)	-1083.12089	1.49	7	1.23	10	0
**1e** (conf. 14)	-1083.12326	0.00	93	0.00	85	0
**1e** (conf. 29)	-1083.11934	2.46		1.73	5	0
**3** (conf. 1)	-1043.79731	0.00	71	0.00	46	0
**3** (conf. 6)	-1043.79616	0.72	21	0.18	34	0
**3** (conf. 19)	-1043.79529	1.26	8	0.51	20	0
**4** (conf. 1)	-1042.59099	0.00	100	0.00	100	0
**5** (conf. 1)	-1043.79395	2.49		1.99	3	0
**5** (conf. 22)	-1043.79791	0.00	100	0.00	97	0

**6** (conf. 1)	-1196.48907	0.00	100	0.00	100	0
**7** (conf. 1)	-1345.74598	0.90	12	0.34	23	0
**7** (conf. 13)	-1345.74556	1.16	8	0.00	42	0
**7** (conf. 24)	-1345.74741	0.00	55	0.24	28	0
**7** (conf. 37)	-1345.74665	0.48	25	1.07	7	0
**8** (conf. 5)	-1737.81962	0.00	48	0.00	78	0
**8** (conf. 6)	-1737.81758	1.28	5	1.79	4	0
**8** (conf. 11)	-1737.81883	0.49	22	1.53	6	0
**8** (conf. 16)	-1737.81894	0.43	23	1.11	12	0
**8** (conf. 17)	-1737.81679	1.77	2	3.20		0
**9** (conf. 13)[b]	-2313.10553	0.00	89	0.00	100	0
**9** (conf. 26)[b]	-2313.10358	1.22	11	2.44		0
**11** (conf. 1)	-925.788161	0.22	37	0.00	50	0
**11** (conf. 25)	-925.786378	1.34	5	1.58	3	0
**11** (conf. 49)	-925.786383	1.34	5	1.15	7	0
**11** (conf. 73)	-925.788513	0.00	53	0.14	40	0

[a] Conformers are numbered according to their appearance during conformational search; [b] calculations were carried out at the B3LYP/6-311G(d,p) level.

Table S_2. Dihedral angles α, β, γ and δ (in degrees) and selected interatomic distances l_1 and l_2 (in Å) calculated at the B3LYP/6-311++G(d,p) level for individual low-energy conformers of **1a**-**1e**, **3**-**9** and **11**.

Compound[a]	α_1[b]	α_2[b]	α_3[b]	β_1[c]	β_2[c]	β_3[c]	γ_1[d]	γ_2[d]	γ_3[d]	δ[e]	ζ[f]	ϕ[g]	l_1[h]	l_2[i]
**1a** (conf. 1)	-56.2	64.3	180.0	70.4	-169.0	-53.4	-77.0	-4.2	-44.6	60.2			2.485	2.294
**1a** (conf. 72)	46.0	160.9	-76.2	166.2	-78.8	44.0	50.3	6.3	64.1	-61.8			2.447	
**1b** (conf. 1)	-57.0	63.5	179.1	69.8	-169.6	-54.0	-77.3	-2.7	-44.6	57.9			2.468	2.307
**1b** (conf. 27)	45.5	160.5	-76.7	166.1	-78.8	43.9	55.1	8.2	62.8	-63.2			2.456	2.467
**1b** (conf. 40)	44.8	160.0	-77.2	166.0	-78.8	44.0	52.4	10.2	60.9	-60.3			2.407	2.434
**1b** (conf. 82)	-57.0	63.4	178.9	69.4	-170.1	-54.6	-77.8	-2.3	-44.4	59.2			2.477	2.307
**1c** (conf. 4)	51.3	-71.0	165.7	177.5	55.2	-68.1	40.3	72.9	-6.6	-170.0				2.512
**1c** (conf. 9)	50.3	164.4	-72.1	174.5	-71.3	52.1	45.4	0.4	70.4	-63.3			2.483	2.600
**1c** (conf. 24)	42.3	155.4	-78.8	161.6	-85.3	40.4	51.1	-4.7	62.7	-58.9			2.336	2.445
**1c** (conf. 25)	-62.5	57.5	171.7	62.8	-177.1	-62.9	-78.6	6.1	-48.7	59.0			2.469	2.316
**1c** (conf. 36)	-59.7	60.8	175.7	70.1	-169.4	-54.4	-82.2	1.7	-42.4	60.3			2.537	2.320
**1d** (conf. 2)	-56.7	63.7	179.2	69.7	-169.9	-54.4	-77.6	-2.2	-44.7	58.5			2.469	2.313
**1d** (conf. 20)	-57.4	63.1	178.6	70.1	-169.3	-53.8	-77.3	-1.5	-44.7	60.0			2.474	2.317
**1d** (conf. 25)	46.1	-76.1	160.8	166.2	44.0	-79.1	50.7	64.2	6.2	-62.4			2.441	2.488
**1d** (conf. 26)	-57.6	62.7	178.2	69.4	-170.3	-54.8	-78.0	-1.3	-45.2	61.3			2.486	2.310

**1e** (conf. 1)	-63.1	56.9	171.0	62.2	-177.7	-63.7	-79.5	7.3	-47.7	60.3			2.480	2.328
**1e** (conf. 14)	50.6	-71.8	164.7	175.2	52.8	-70.7	46.3	70.2	0.2	-63.2			2.477	2.589
**1e** (conf. 29)	50.5	-71.9	164.9	177.0	54.5	-68.6	41.9	72.4	-5.1	-173.3				2.511
**3** (conf. 1)	45.4			61.6	-175.7	-63.0	-78.4	18.9	-44.4	44.4			2.580	2.438
**3** (conf. 6)	53.1			44.1	167.5	-79.0	70.8	50.0	-3.9	-73.9				2.474
**3** (conf. 19)	53.0			49.6	172.9	-74.1	79.6	41.9	-18.0	-177.8				2.594
**4** (conf. 1)	-9.9			81.3	-157.7	-43.7	-74.1	0.2	-45.4					2.210
**5** (conf. 1)	-65.6			84.8	-154.7	-41.4	-77.6	0.5	-47.8	-52.4				2.378
**5** (conf. 22)	-78.2			57.9	-178.7	-65.0	76.3	40.7	-12.1	67.7			2.425	
**6** (conf. 1)	58.7			65.4	-172.7	-59.9	-77.5	16.3	-46.8		-27.0			2.737
**7** (conf. 1)	55.2			-72.5	51.8	174.6	-22.4	80.4	39.2	-178.0	-36.0	-109.8		2.656
**7** (conf. 13)	52.9			-74.9	49.3	172.4	-18.7	77.8	42.2	178.7	-36.1	68.5		2.652
**7** (conf. 24)	64.2			-56.9	68.4	-170.2	-47.7	-77.6	14.1	-0.7	-17.7	-171.6		2.585
**7** (conf. 37)	54.8			-66.6	58.7	-179.0	-40.1	-83.6	24.7	-2.1	-37.9	-8.3		2.714
**8** (conf. 5)	55.7			-73.8	50.4	173.4	-19.4	79.4	41.1		-37.7	-122.4		2.600
**8** (conf. 6)	55.8			-73.8	50.4	173.5	-19.2	79.3	41.3		-37.5	-126.7		2.593
**8** (conf. 11)	52.8			-75.4	49.0	172.2	-18.9	78.2	41.8		-37.2	92.8		2.678

**8** (conf. 16)	51.5			-77.1	47.0	170.3	-15.1	76.1	44.6		-37.0	104.4		2.636
**8** (conf. 17)	51.9			-77.4	46.6	170.0	-14.5	76.1	45.0		-37.0	103.1		2.628
**9** (conf. 13)[i,j]	51.6			47.6	170.9	-76.3	16.8	43.6	-15.8		-36.4	91.4		2.653
**9** (conf. 26)[i,j]	56.1			47.2	170.7	-76.5	77.2	45.7	-11.9		-40.9	-162.9		2.470
**11** (conf. 1)	179.5			-74.3	48.4	168.9	-0.7	66.8	42.0					
**11** (conf. 25)	-165.8			-179.0	-59.8	64.6	0.1	81.4	42.1					
**11** (conf. 49)	106.4			77.6	-165.7	-45.0	-8.9	-48.7	-60.1					
**11** (conf. 73)	101.8			-77.7	45.5	165.4	12.3	59.3	51.3					2.159

[a] – conformers are numbered according to their appearance during conformational search; [b] – α = O-C*-C-C_{ipso} or O-C*-C*-C(Ph_3); [c] – β = C-C*-C-C_{ipso}; [d] – γ = C-C_{Tr}-C_{ipso}-C_{ortho} (of the two possibilities the absolute values ≤90° has been chosen); [e] – δ = H-O-C*-C; [f] – ζ = H-C*-O-C or (O=)C-C(=O)-O-H [g] – ϕ = O=C-C=O; [h] – l_1 = (O)H···C_{ipso}; [h] – l_2 = C_{ortho}H···O-C or C_{ortho}H···O=C; [i] – optimized at the B3LYP/6-311G(d,p) level; [j] – C_2 symmetry.

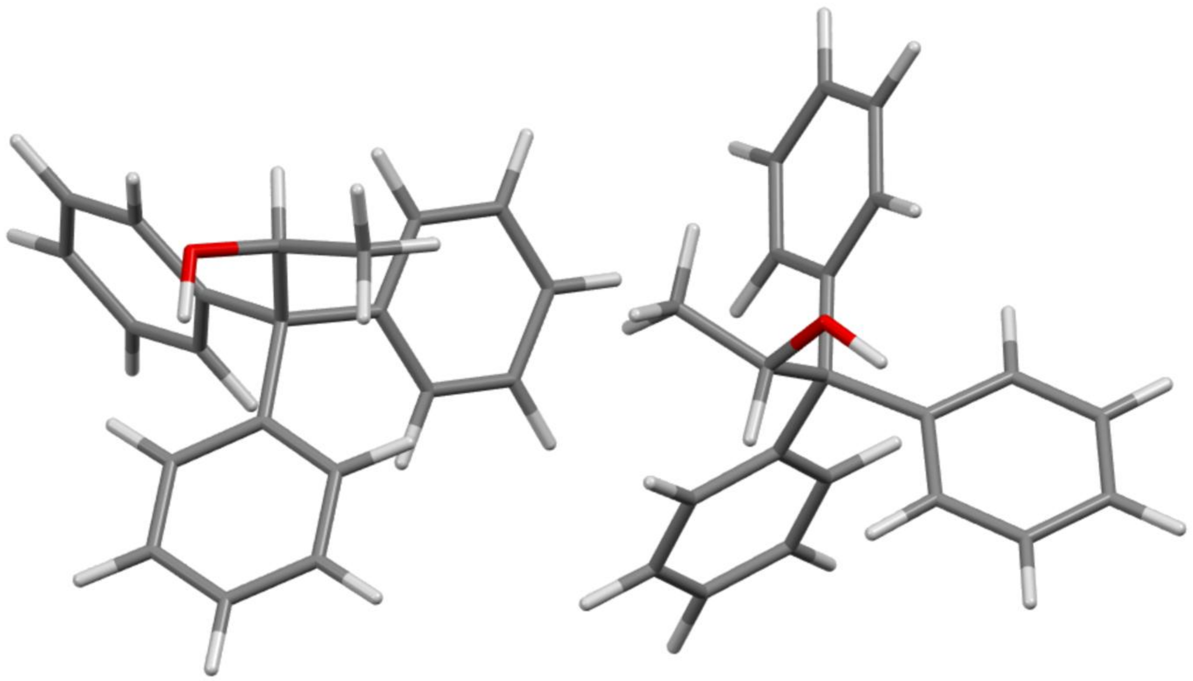

1a (conf. 1) **1a** (conf. 72)

Figure S_1. Structures of individual, low-energy conformers of **1a**, calculated at the B3LYP/6-311++G(d,p) level of theory.

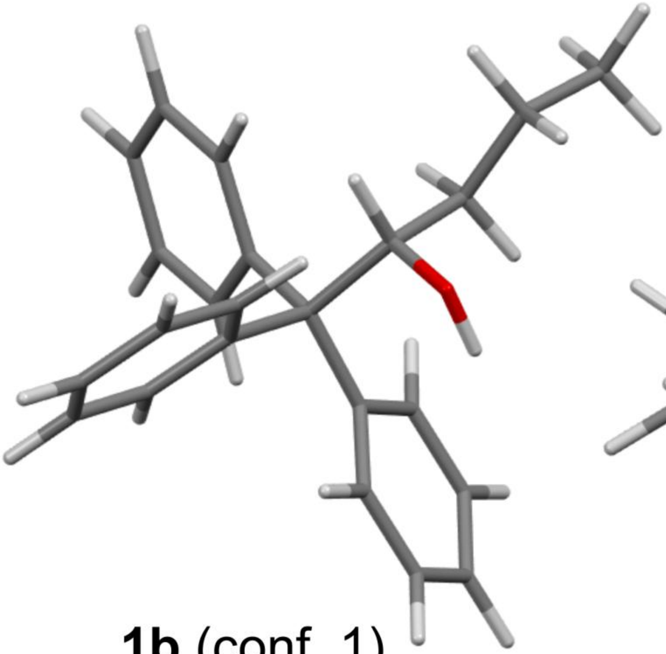

1b (conf. 1)

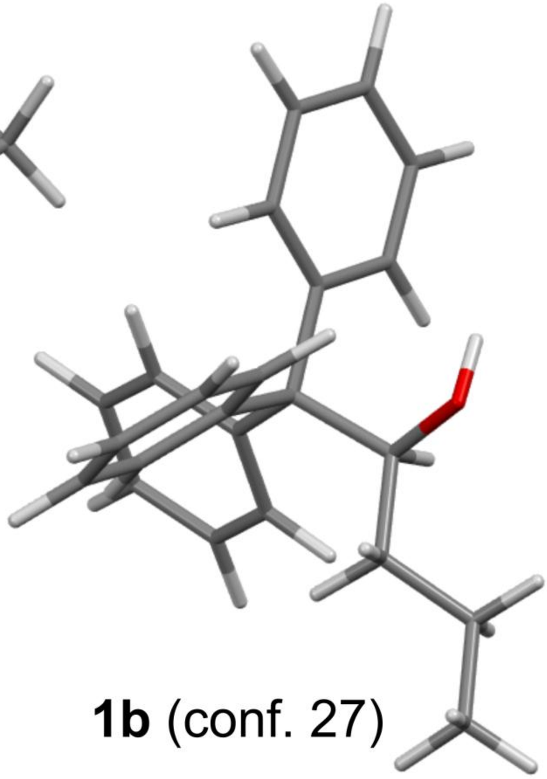

1b (conf. 27)

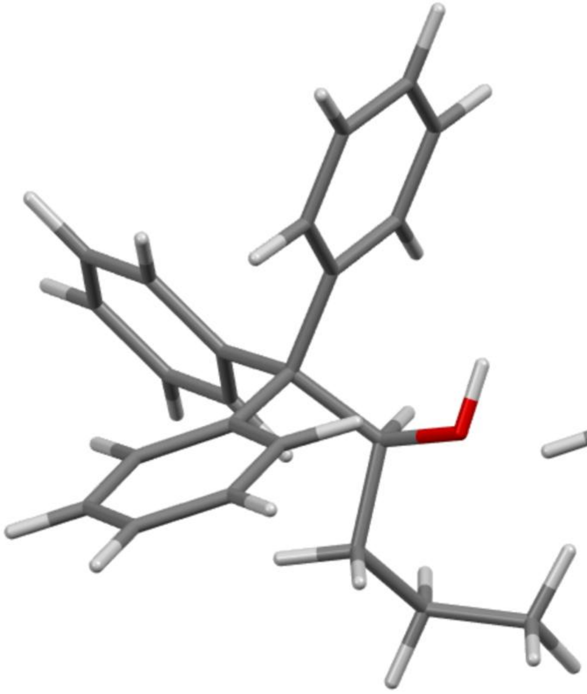

1b (conf. 40)

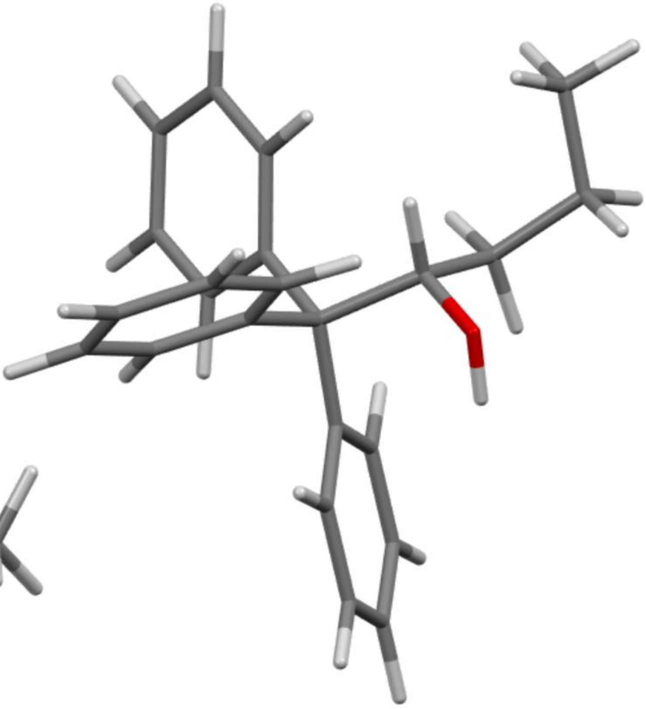

1b (conf. 82)

Figure S_2. Structures of individual, low-energy conformers of **1b**, calculated at the B3LYP/6-311++G(d,p) level of theory.

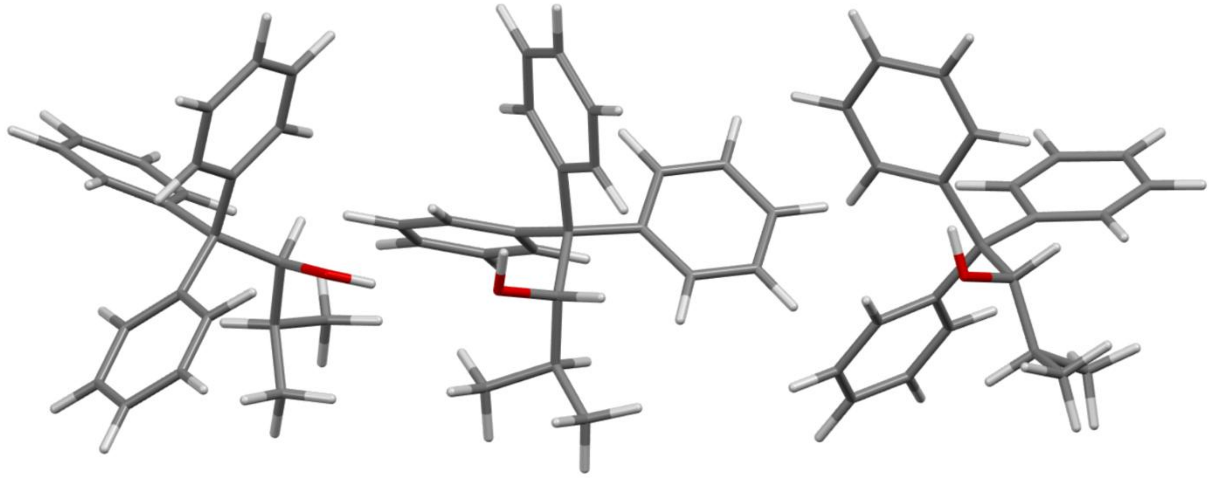

1c (conf. 4) **1c** (conf. 9) **1c** (conf. 24)

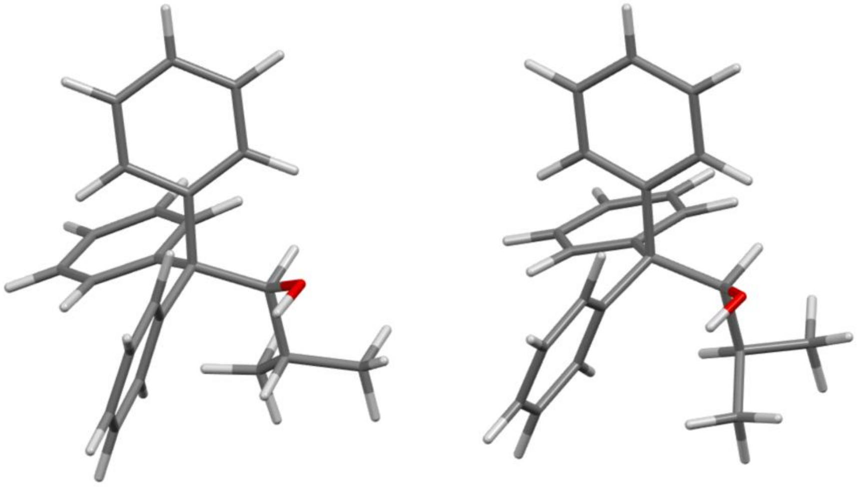

1c (conf. 25) **1c** (conf. 36)

Figure S_3. Structures of individual, low-energy conformers of **1c**, calculated at the B3LYP/6-311++G(d,p) level of theory.

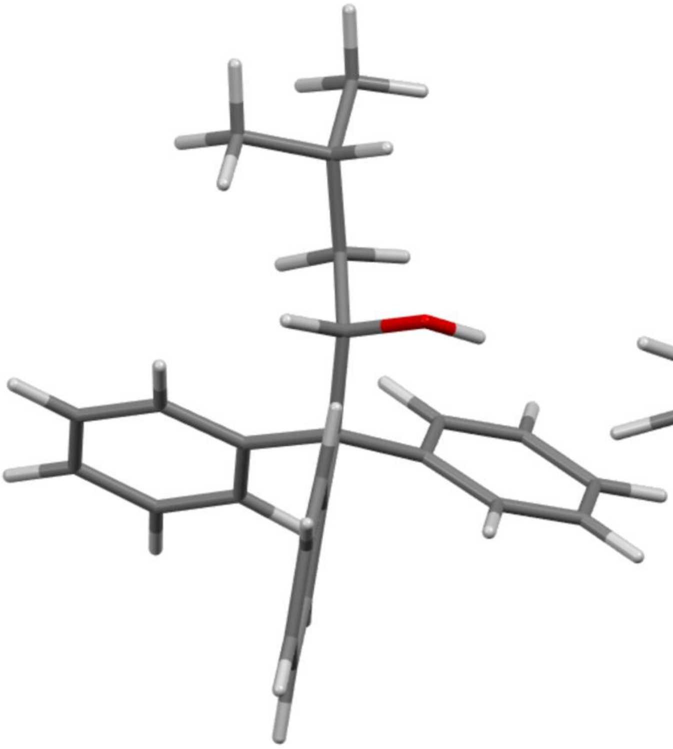

1d (conf. 2)

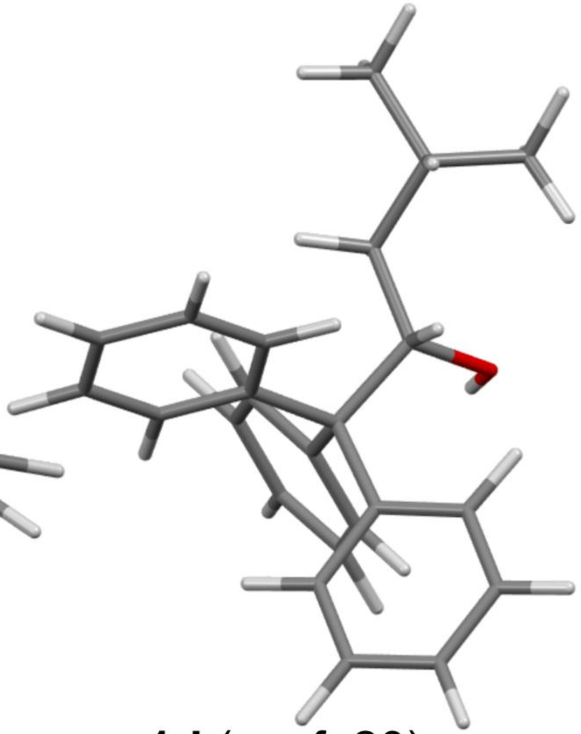

1d (conf. 20)

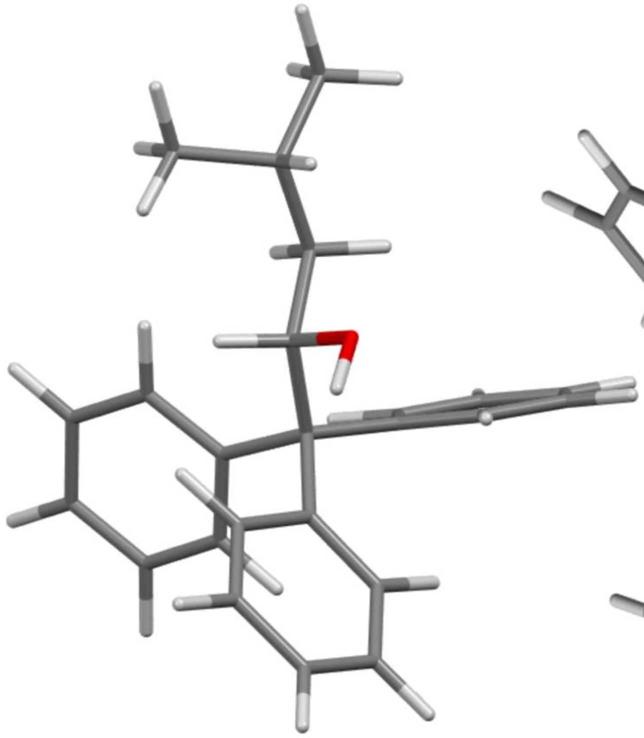

1d (conf. 25)

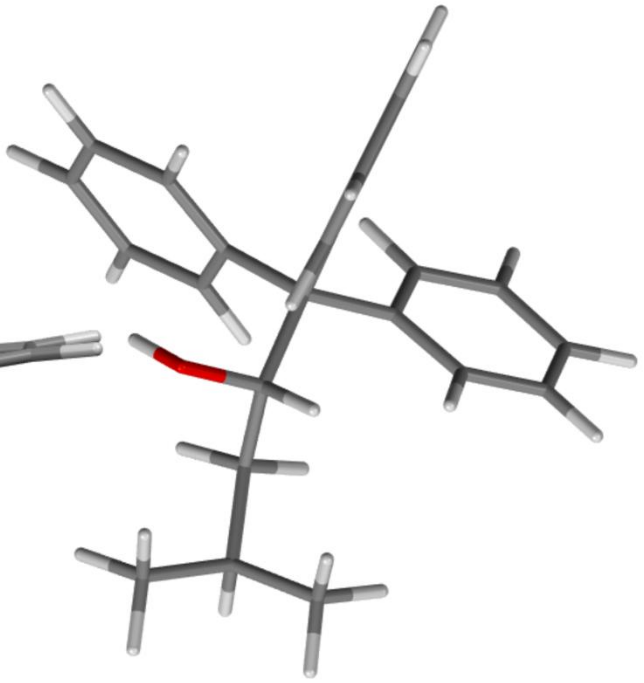

1d (conf. 26)

Figure S_4. Structures of individual, low-energy conformers of **1d**, calculated at the B3LYP/6-311++G(d,p) level of theory.

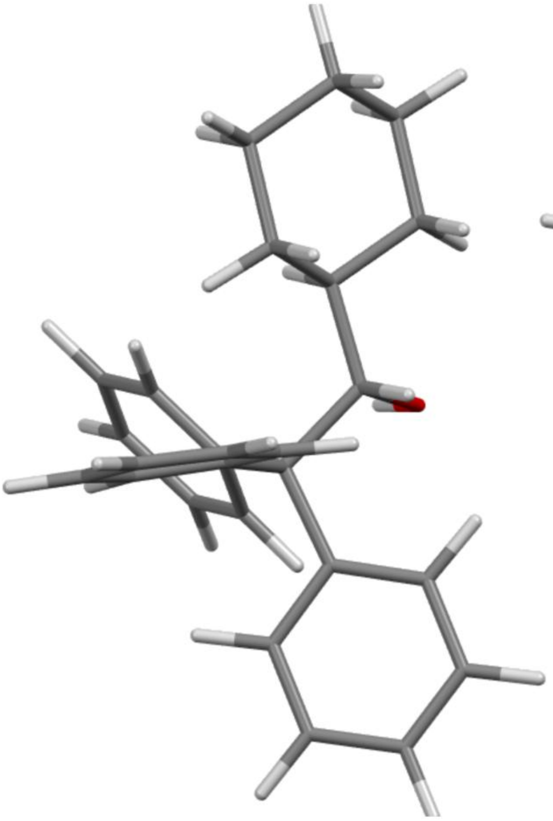

1e (conf. 1)

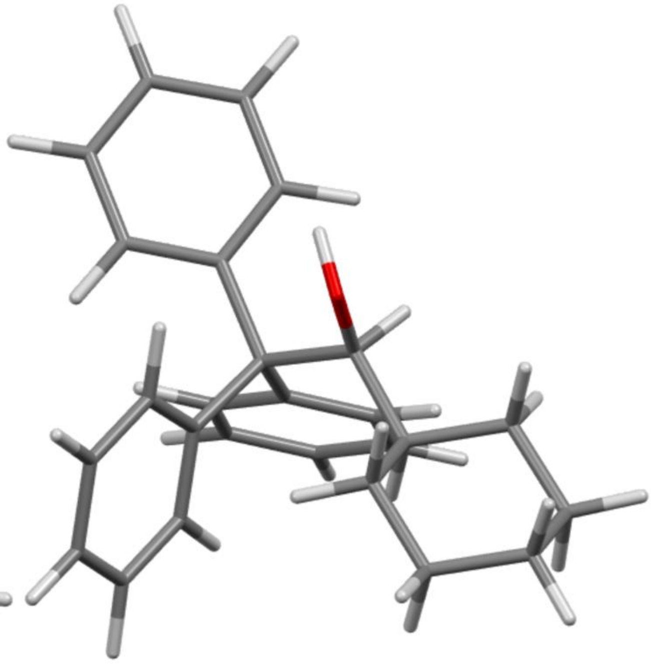

1e (conf. 14)

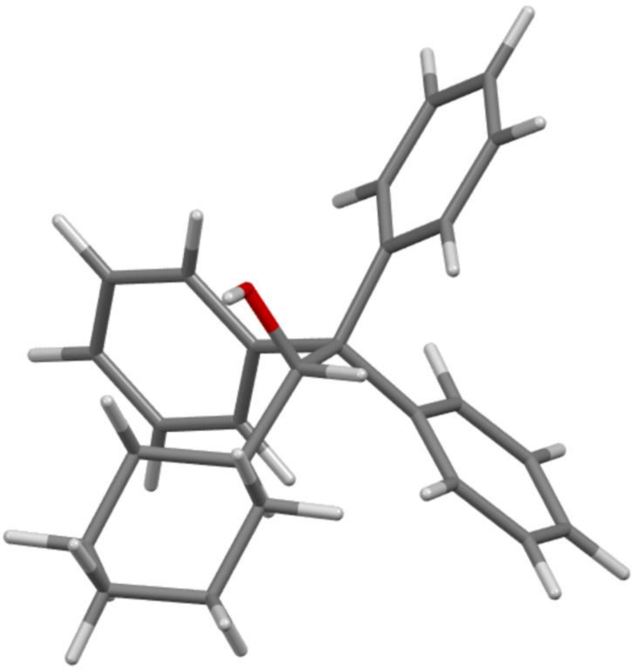

1e (conf. 29)

Figure S_5. Structures of individual, low-energy conformers of **1e**, calculated at the B3LYP/6-311++G(d,p) level of theory.

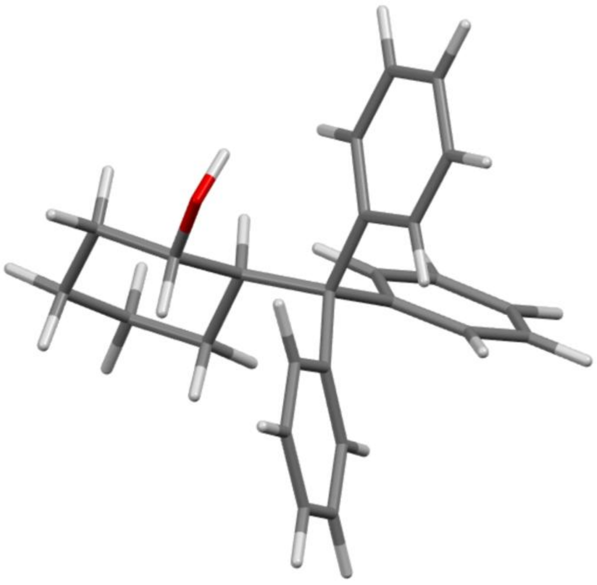

3 (conf. 1)

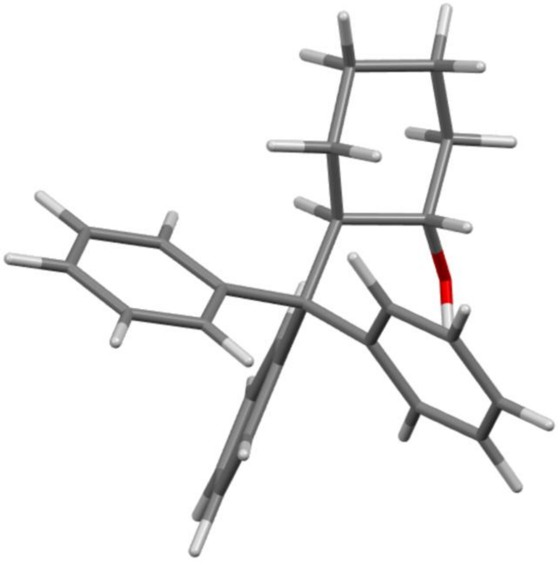

3 (conf. 6)

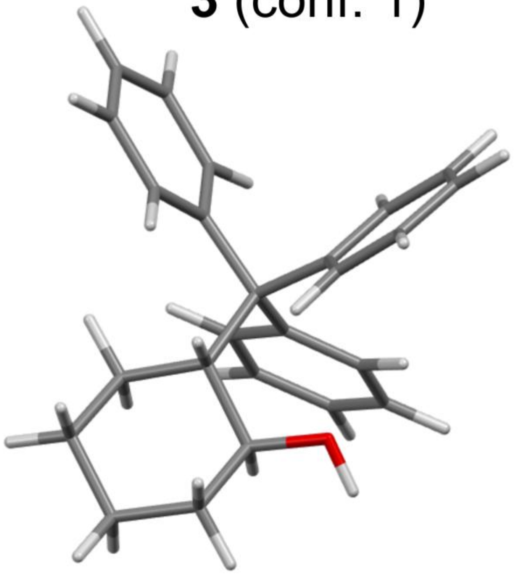

3 (conf. 19)

Figure S_6. Structures of individual, low-energy conformers of **3**, calculated at the B3LYP/6-311++G(d,p) level of theory.

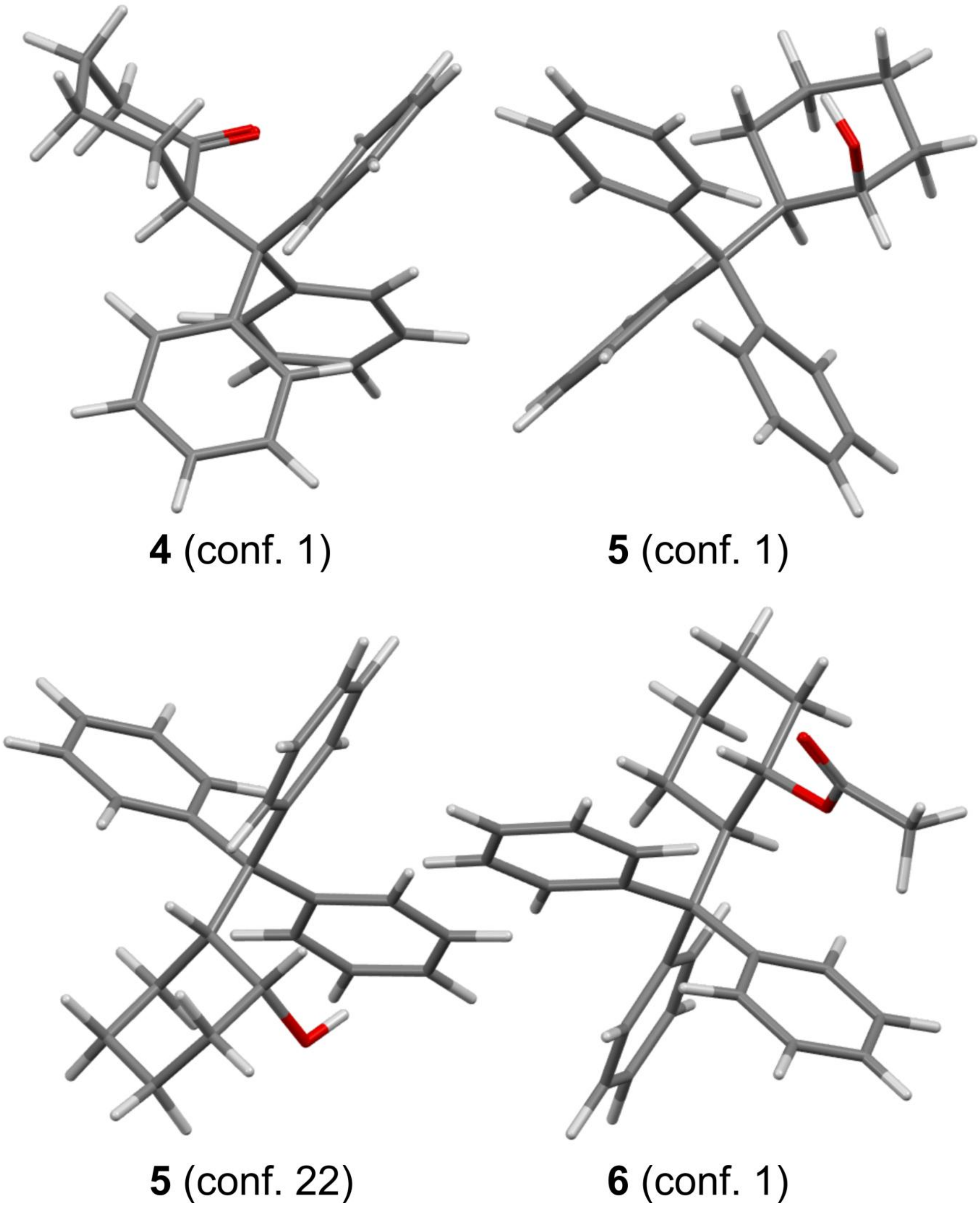

Figure S_7. Structures of individual, low-energy conformers of **4**-**6**, calculated at the B3LYP/6-311++G(d,p) level of theory.

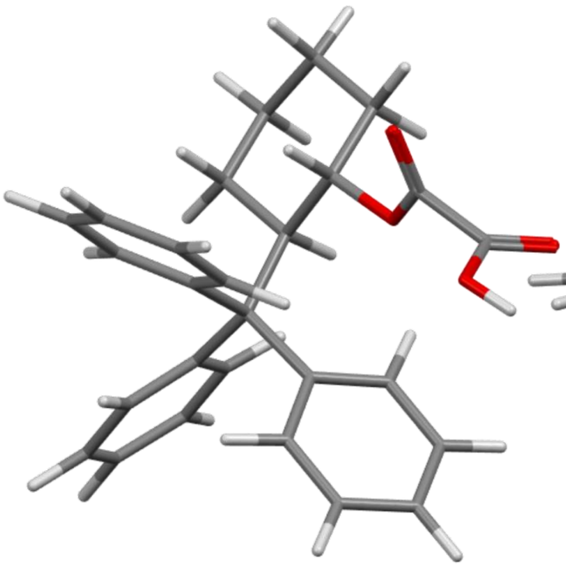

7 (conf. 1)

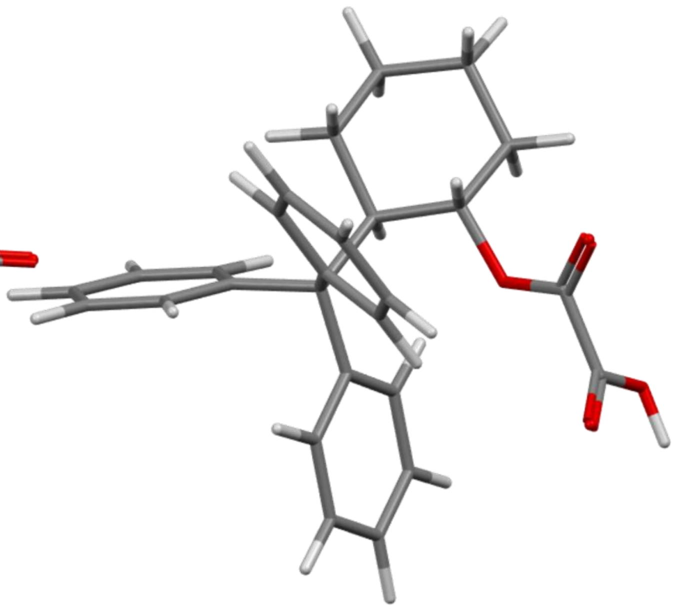

7 (conf. 13)

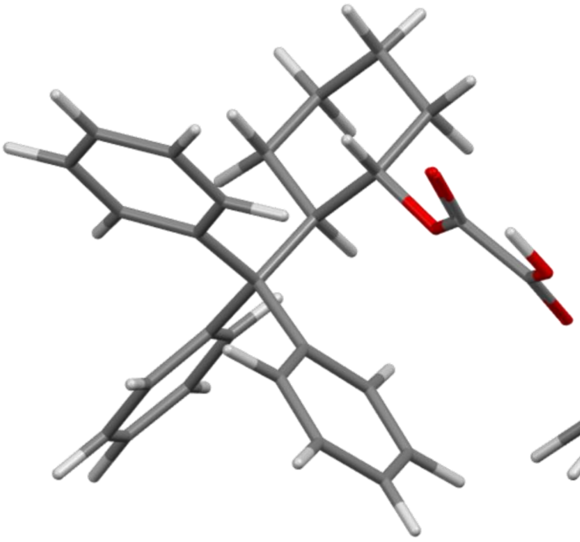

7 (conf. 24)

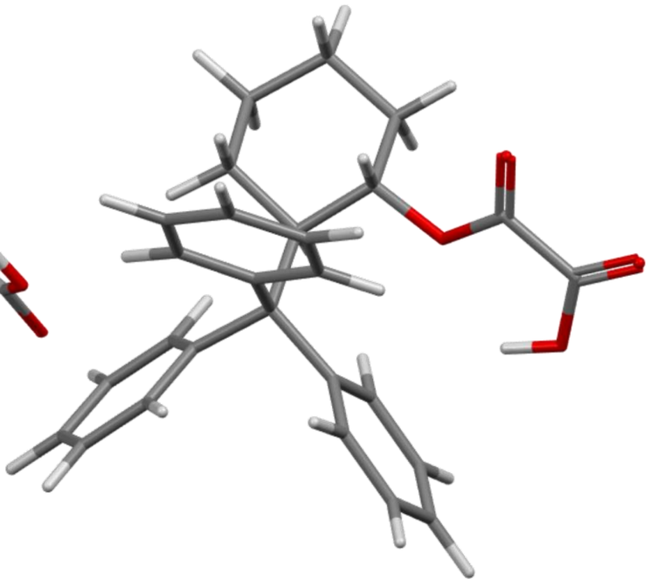

7 (conf. 37)

Figure S_8. Structures of individual, low-energy conformers of **7**, calculated at the B3LYP/6-311++G(d,p) level of theory.

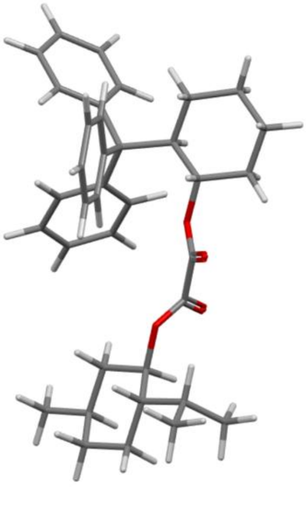

8 (conf. 5)

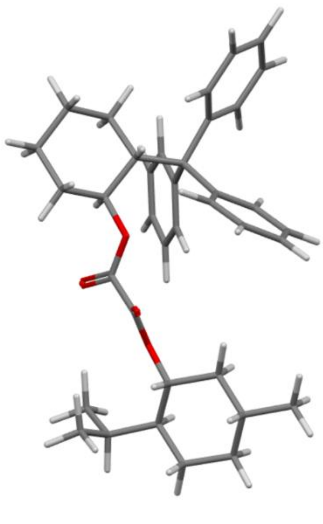

8 (conf. 6)

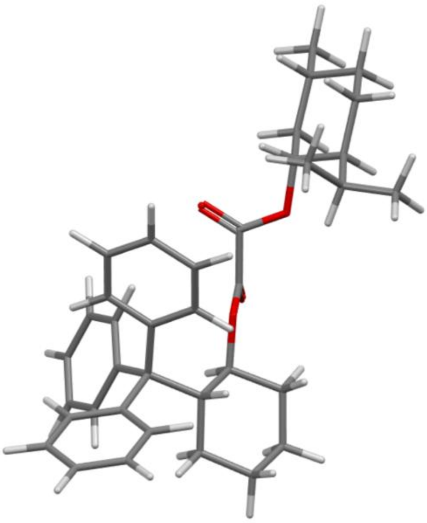

8 (conf. 11)

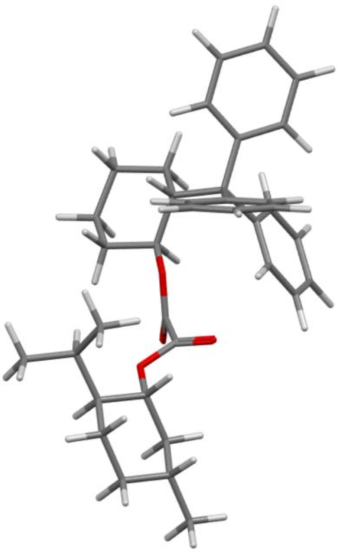

8 (conf. 16)

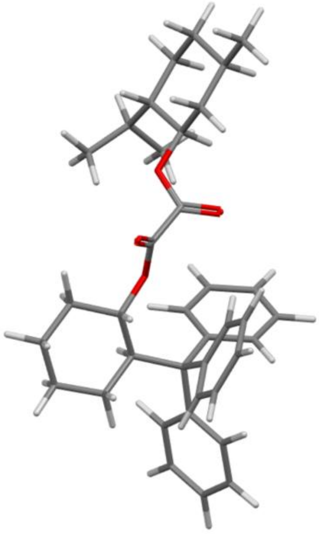

8 (conf. 17)

Figure S_9. Structures of individual, low-energy conformers of **8**, calculated at the B3LYP/6-311++G(d,p) level of theory.

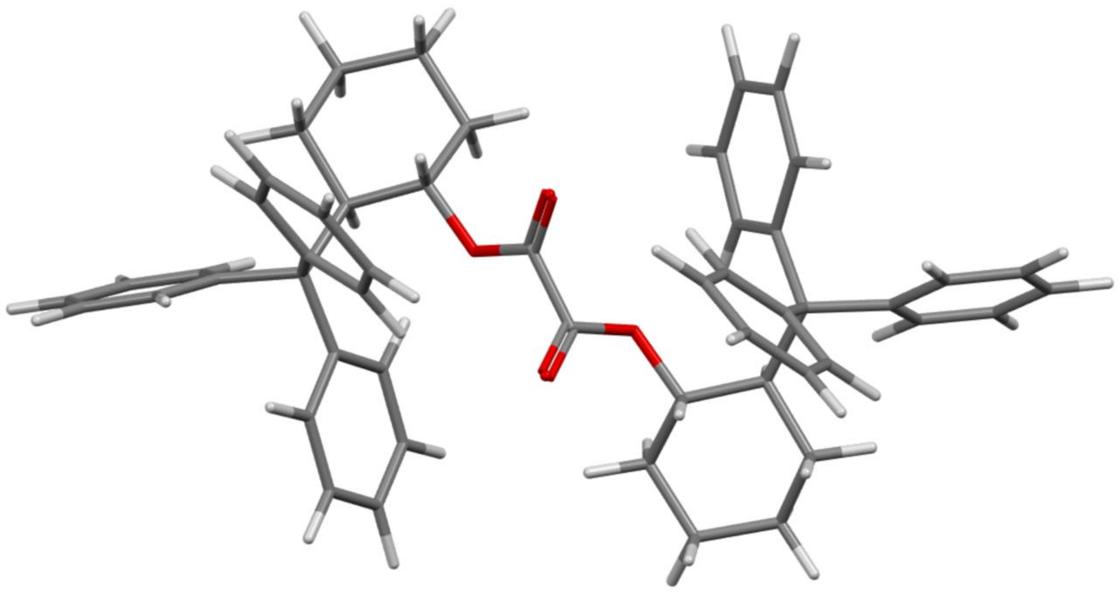

9 (conf. 13)

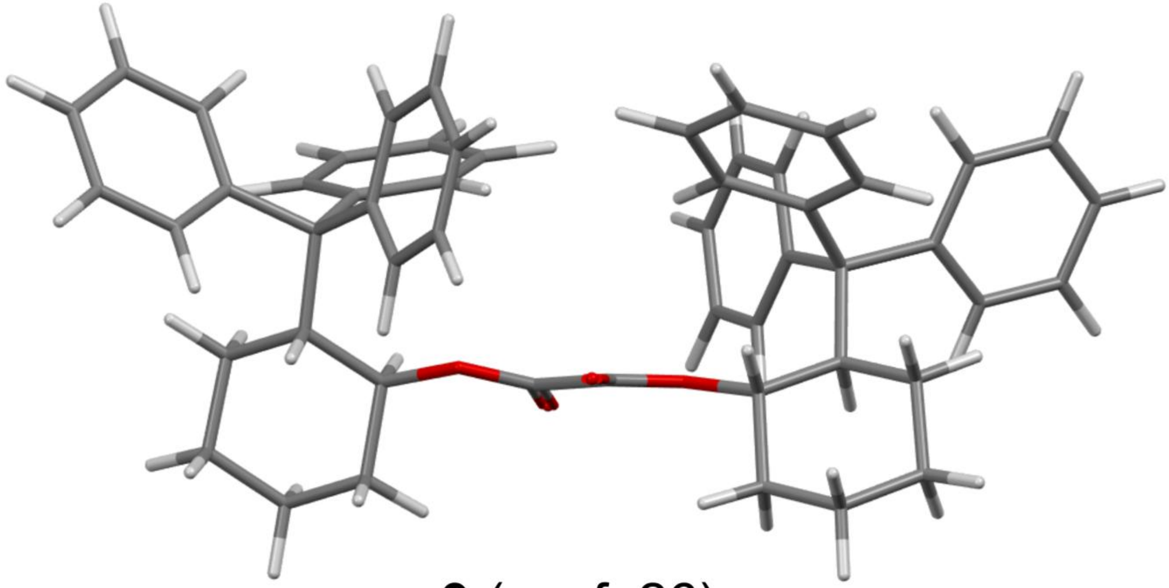

9 (conf. 26)

Figure S_10. Structures of individual, low-energy conformers of **9**, calculated at the B3LYP/6-311++G(d,p) level of theory.

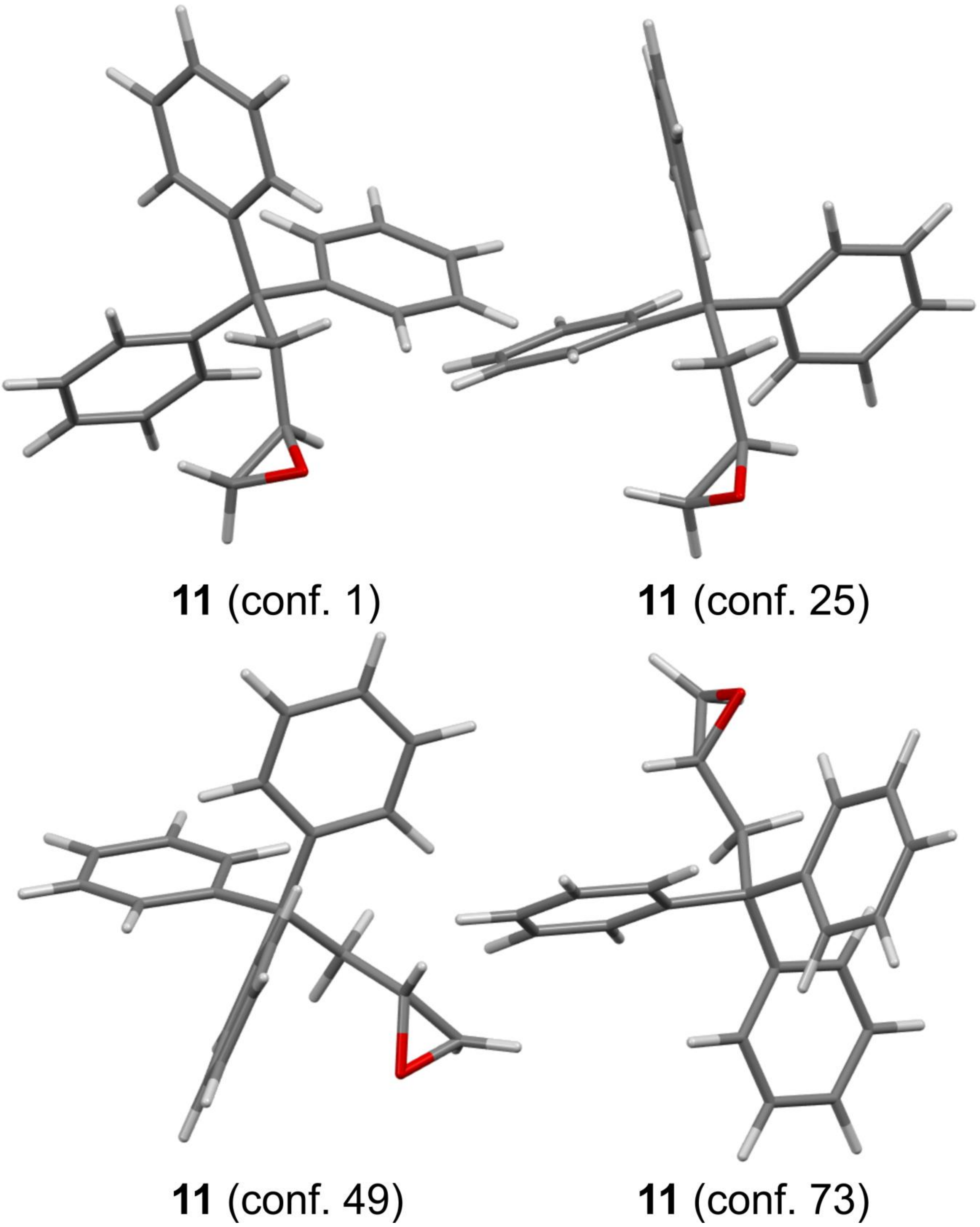

Figure S_11. Structures of individual, low-energy conformers of **11**, calculated at the B3LYP/6-311++G(d,p) level of theory.

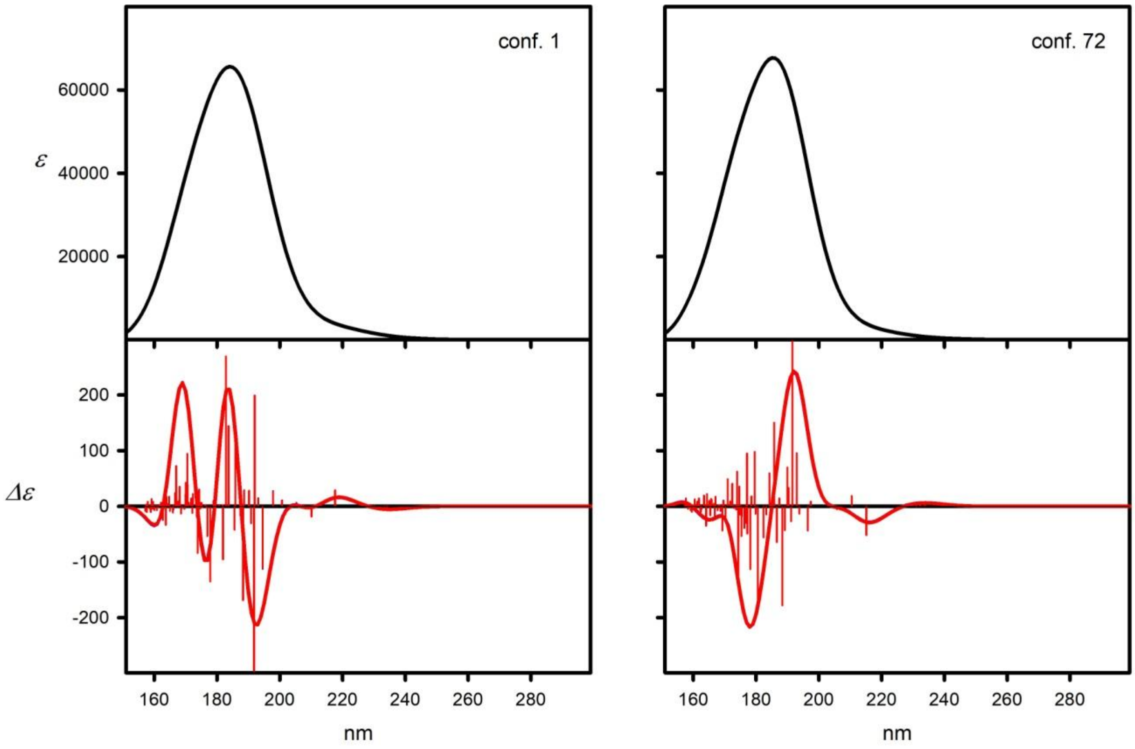

Figure S_12. UV and ECD spectra of the low-energy conformers of compound **1a** calculated at the TD-CAM-B3LYP/6-311++G(d,p) level for structures optimized at the B3LYP/6-311++G(d,p) level. Wavelengths have not been corrected.

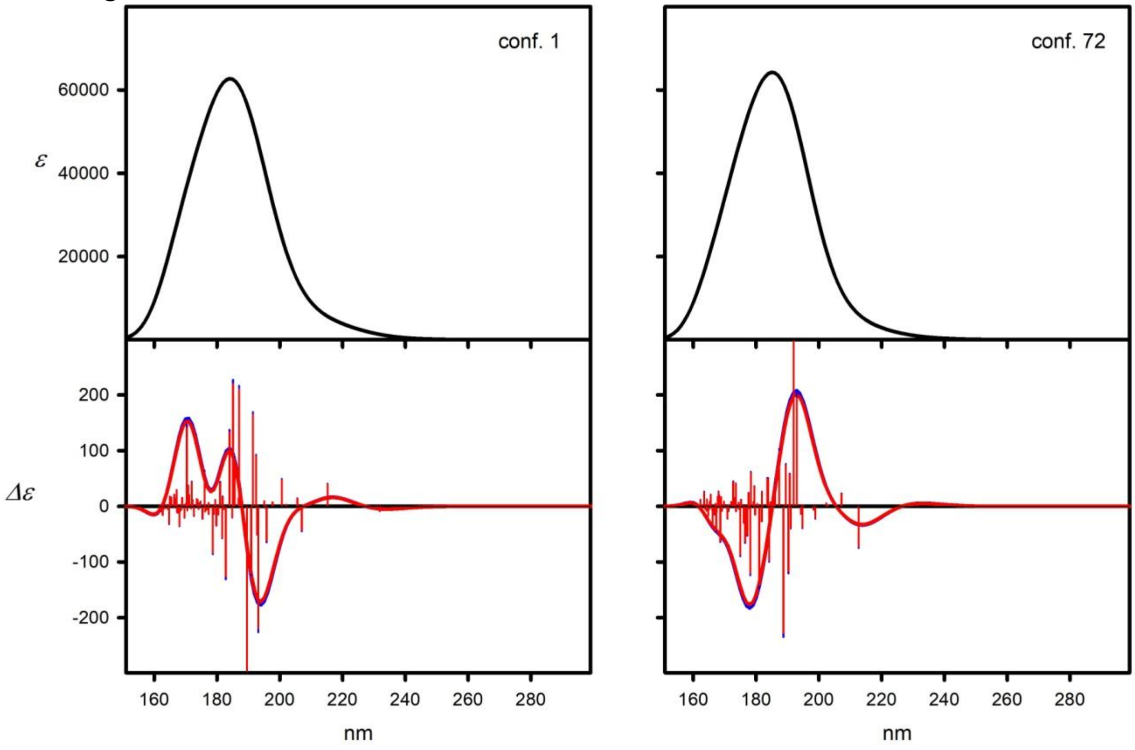

Figure S_13. UV and ECD spectra of the low-energy conformers of compound **1a** calculated at the TD-M06-2X/6-311++G(d,p) level for structures optimized at the B3LYP/6-311++G(d,p) level. Wavelengths have not been corrected.

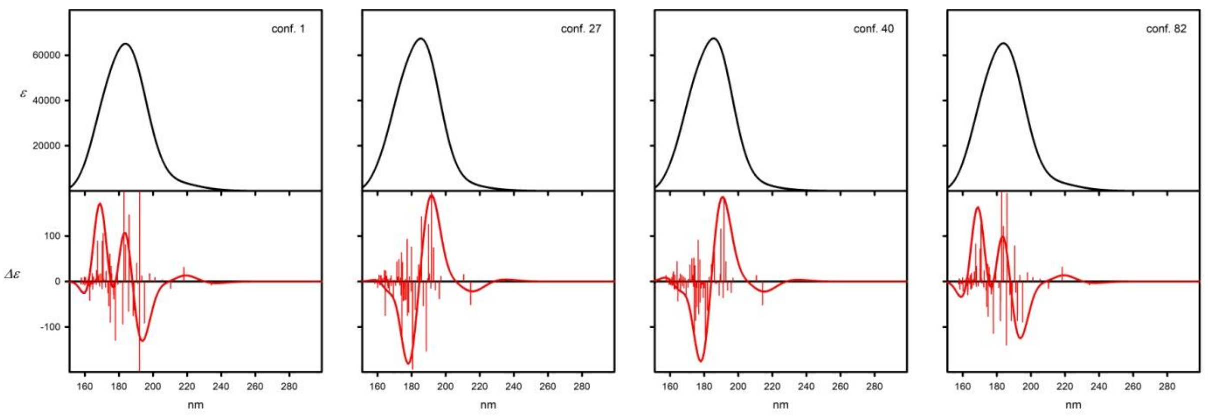

Figure S_14. UV and ECD spectra of the low-energy conformers of compound **1b** calculated at the TD-CAM-B3LYP/6-311++G(d,p) level for structures optimized at the B3LYP/6-311++G(d,p) level. Wavelengths have not been corrected.

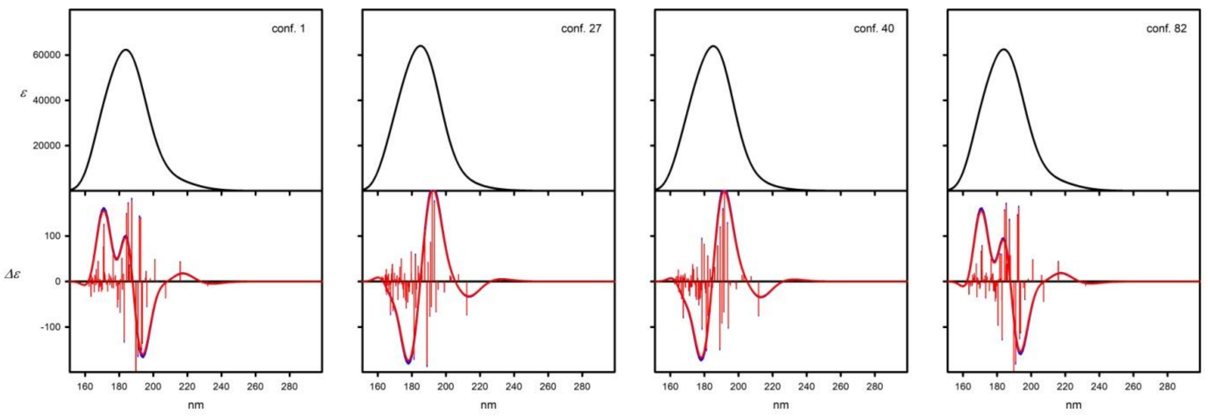

Figure S_15. UV and ECD spectra of the low-energy conformers of compound **1b** calculated at the TD-M06-2X/6-311++G(d,p) level for structures optimized at the B3LYP/6-311++G(d,p) level. Wavelengths have not been corrected.

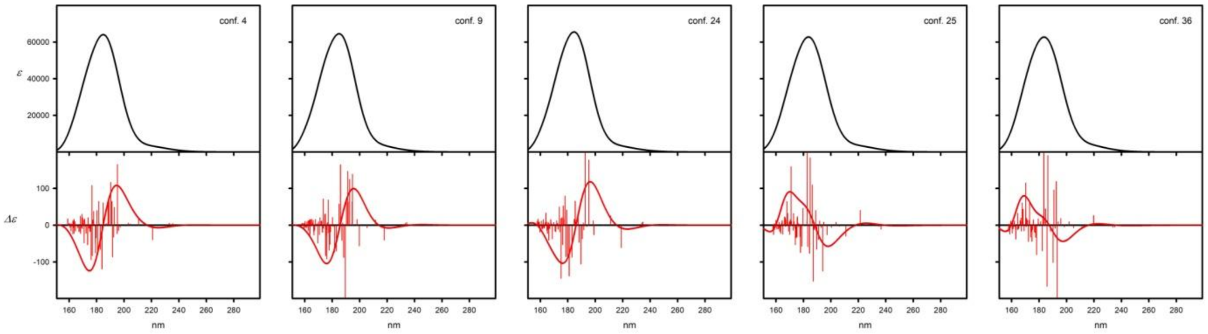

Figure S_16. UV and ECD spectra of the low-energy conformers of compound **1c** calculated at the TD-CAM-B3LYP/6-311++G(d,p) level for structures optimized at the B3LYP/6-311++G(d,p) level. Wavelengths have not been corrected.

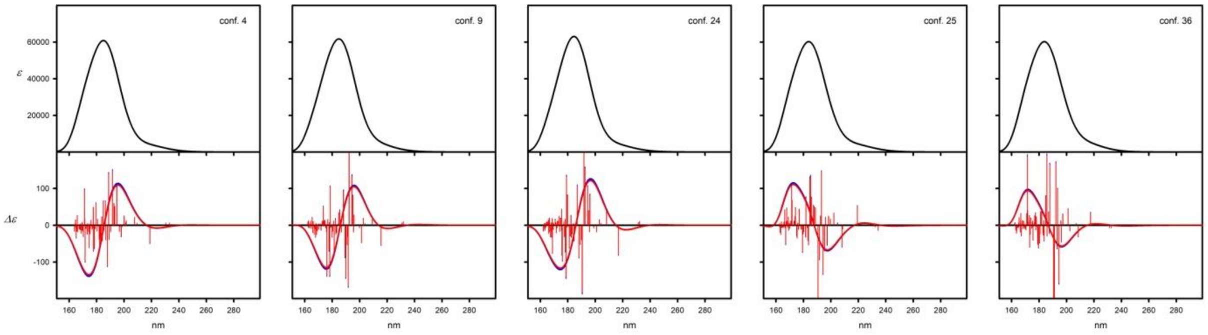

Figure S_17. UV and ECD spectra of the low-energy conformers of compound **1c** calculated at the TD-M06-2X/6-311++G(d,p) level for structures optimized at the B3LYP/6-311++G(d,p) level. Wavelengths have not been corrected.

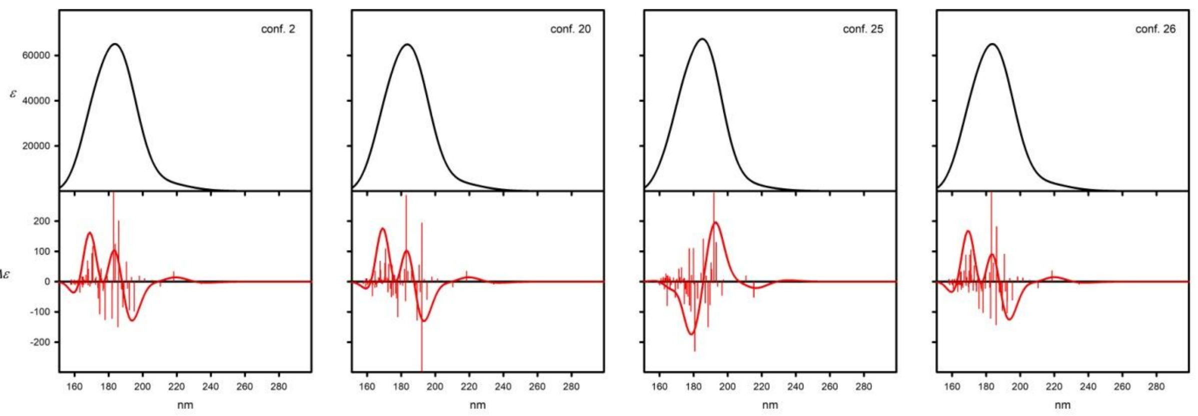

Figure S_18. UV and ECD spectra of the low-energy conformers of compound **1d** calculated at the TD-CAM-B3LYP/6-311++G(d,p) level for structures optimized at the B3LYP/6-311++G(d,p) level. Wavelengths have not been corrected.

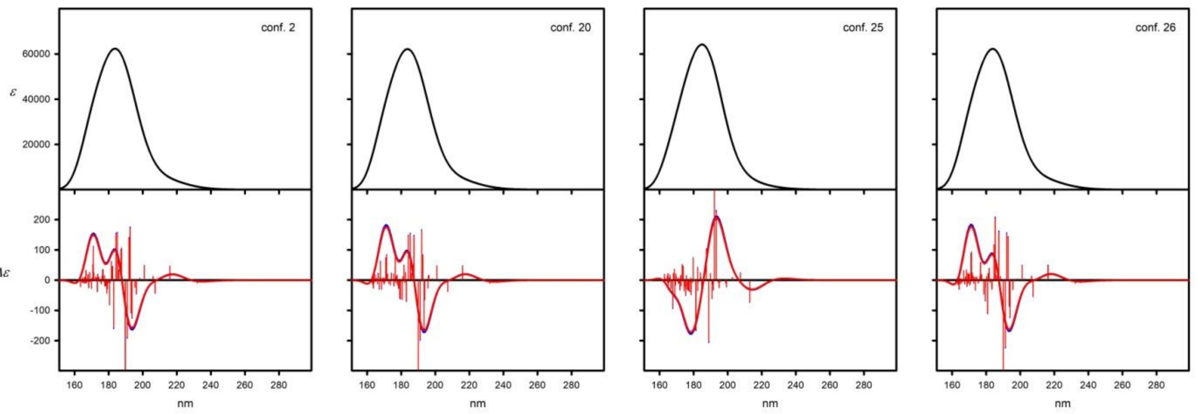

Figure S_19. UV and ECD spectra of the low-energy conformers of compound **1d** calculated at the TD-M06-2X/6-311++G(d,p) level for structures optimized at the B3LYP/6-311++G(d,p) level. Wavelengths have not been corrected.

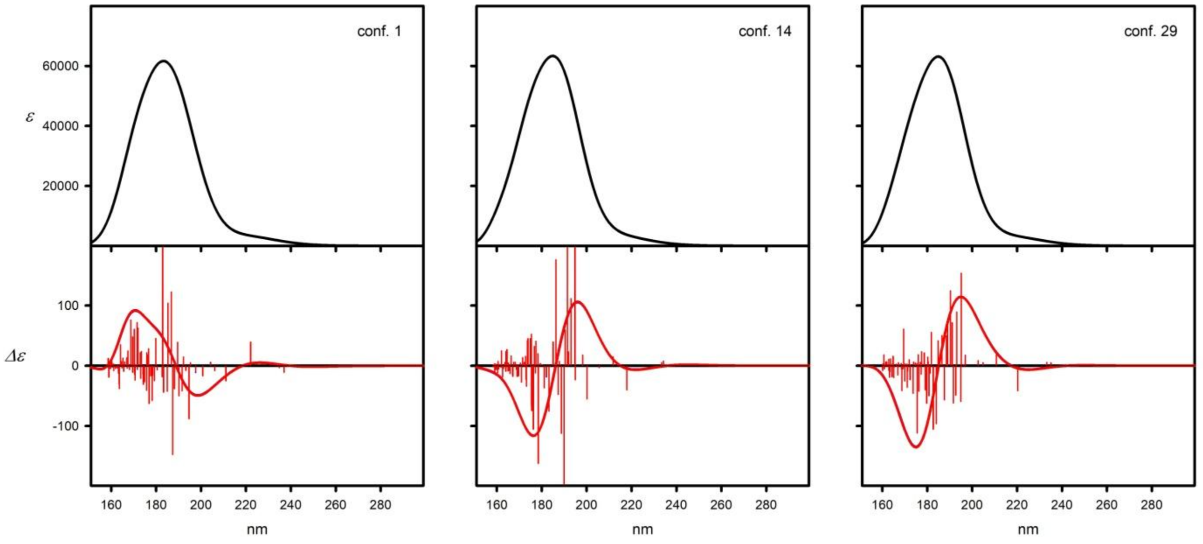

Figure S_20. UV and ECD spectra of the low-energy conformers of compound **1e** calculated at the TD-CAM-B3LYP/6-311++G(d,p) level for structures optimized at the B3LYP/6-311++G(d,p) level. Wavelengths have not been corrected.

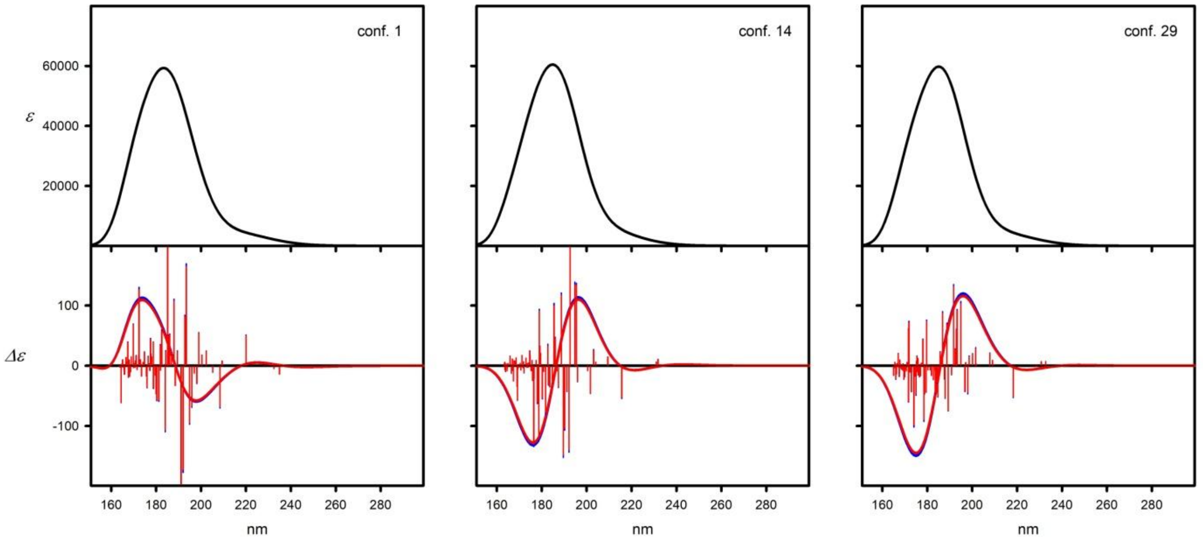

Figure S_21. UV and ECD spectra of the low-energy conformers of compound **1e** calculated at the TD-M06-2X/6-311++G(d,p) level for structures optimized at the B3LYP/6-311++G(d,p) level. Wavelengths have not been corrected.

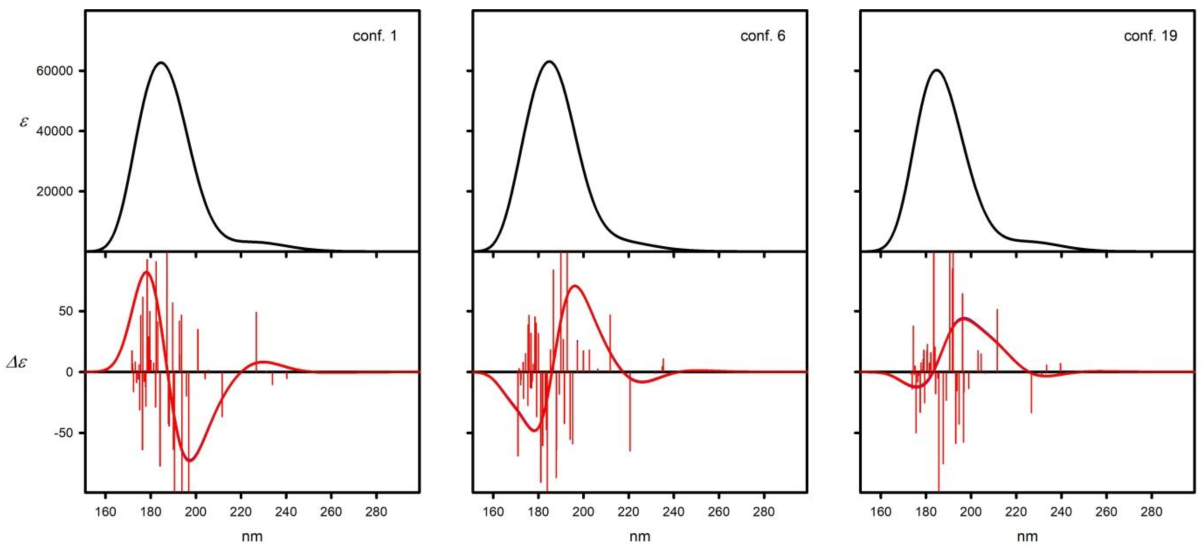

Figure S_22. UV and ECD spectra of the low-energy conformers of compound **3** calculated at the TD-CAM-B3LYP/6-311++G(d,p) level for structures optimized at the B3LYP/6-311++G(d,p) level. Wavelengths have not been corrected.

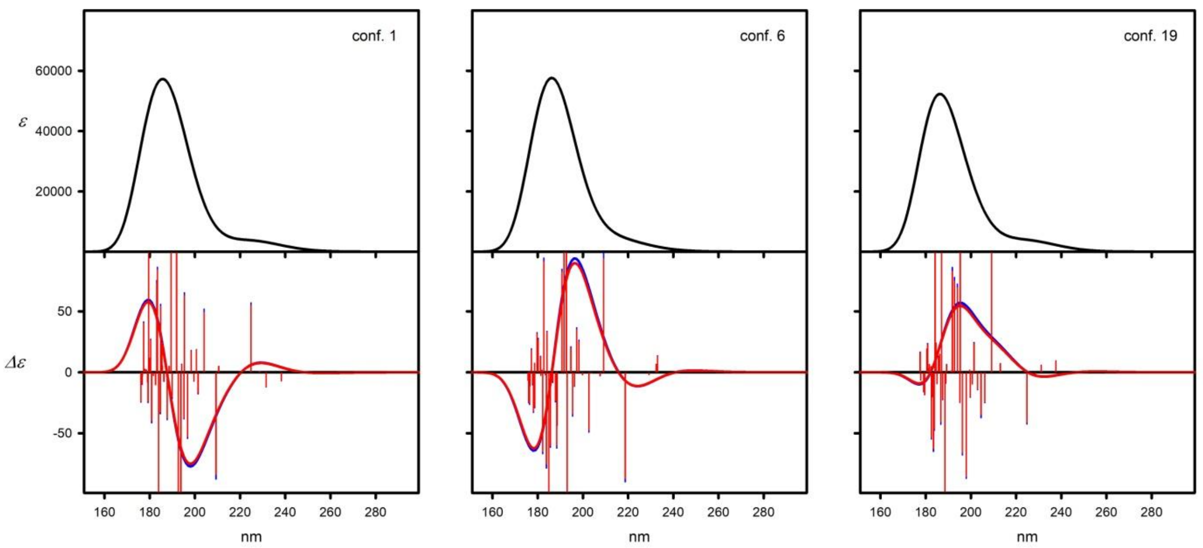

Figure S_23. UV and ECD spectra of the low-energy conformers of compound **3** calculated at the TD-M06-2X/6-311++G(d,p) level for structures optimized at the B3LYP/6-311++G(d,p) level. Wavelengths have not been corrected.

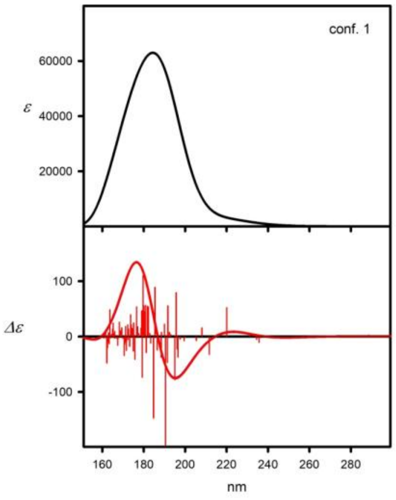

Figure S_24. UV and ECD spectra of the low-energy conformers of compound **4** calculated at the TD-CAM-B3LYP/6-311++G(d,p) level for structures optimized at the B3LYP/6-311++G(d,p) level. Wavelengths have not been corrected.

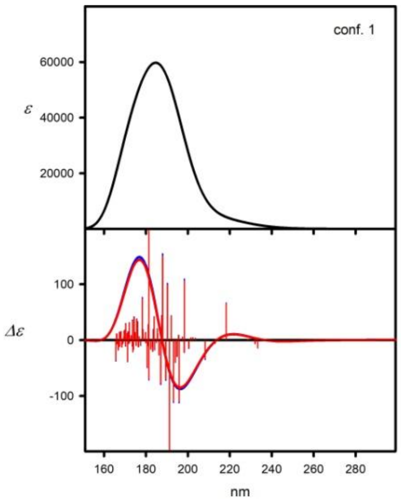

Figure S_25. UV and ECD spectra of the low-energy conformers of compound **4** calculated at the TD-M06-2X/6-311++G(d,p) level for structures optimized at the B3LYP/6-311++G(d,p) level. Wavelengths have not been corrected.

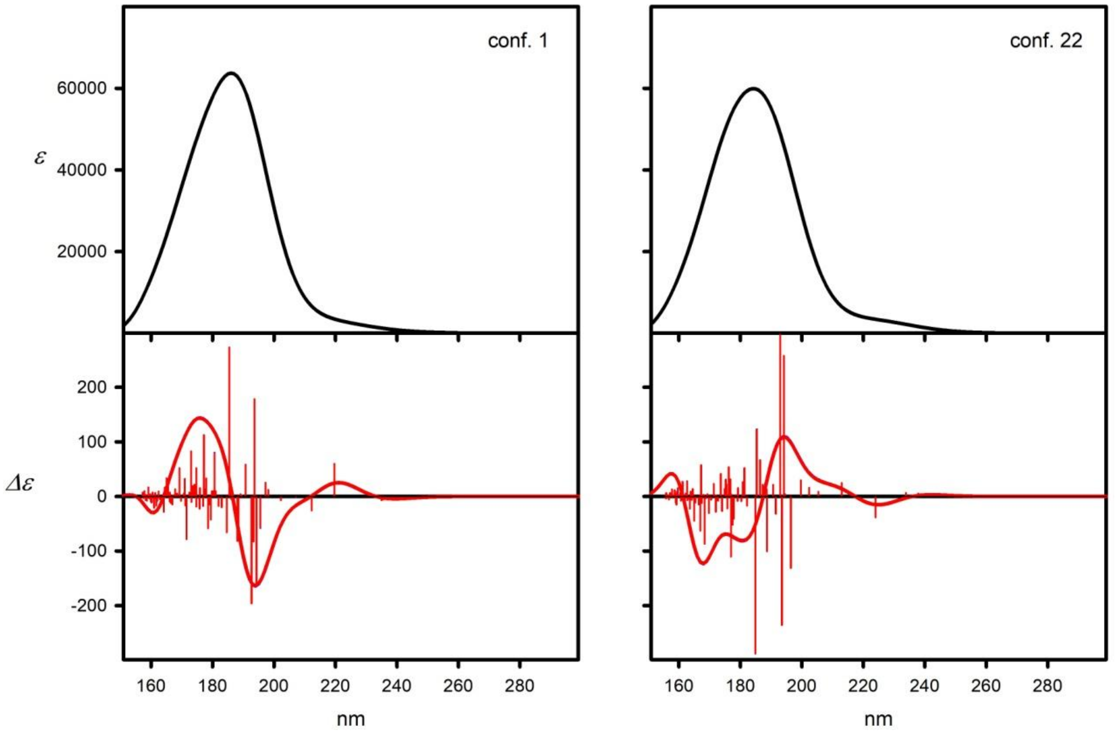

Figure S_26. UV and ECD spectra of the low-energy conformers of compound **5** calculated at the TD-CAM-B3LYP/6-311++G(d,p) level for structures optimized at the B3LYP/6-311++G(d,p) level. Wavelengths have not been corrected.

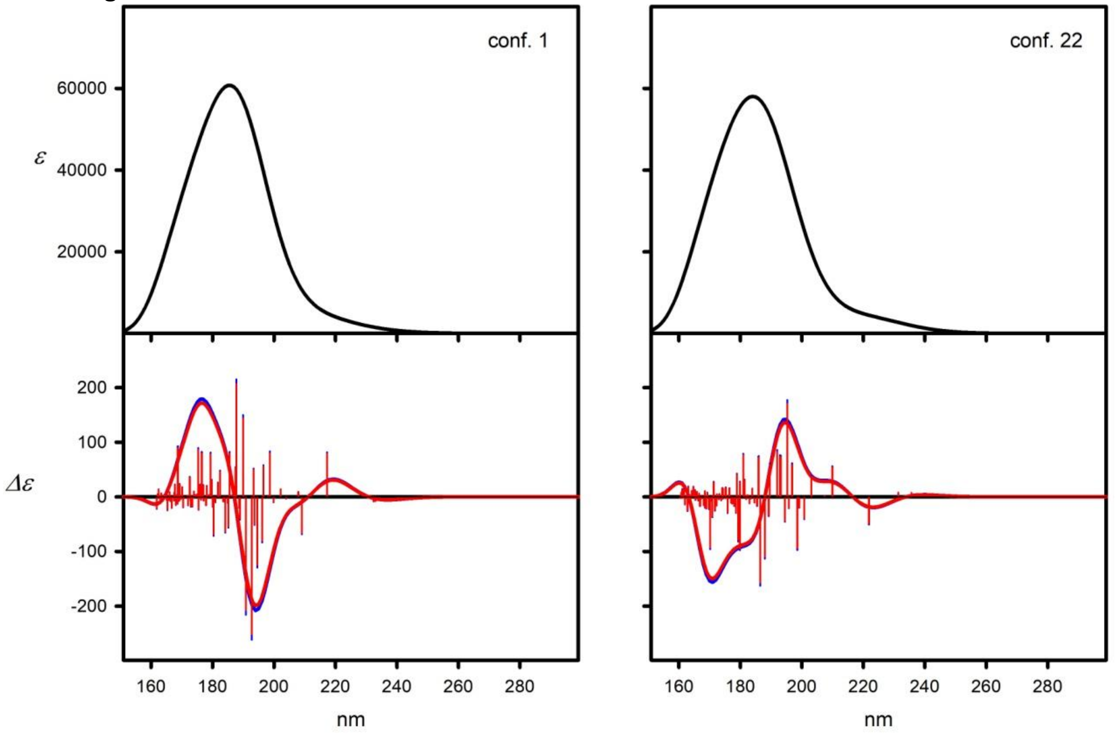

Figure S_27. UV and ECD spectra of the low-energy conformers of compound **5** calculated at the TD-M06-2X/6-311++G(d,p) level for structures optimized at the B3LYP/6-311++G(d,p) level. Wavelengths have not been corrected.

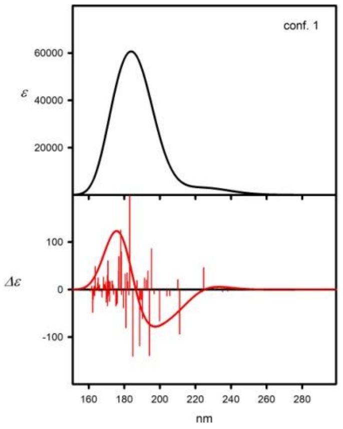

Figure S_28. UV and ECD spectra of the low-energy conformers of compound **6** calculated at the TD-CAM-B3LYP/6-311++G(d,p) level for structures optimized at the B3LYP/6-311++G(d,p) level. Wavelengths have not been corrected.

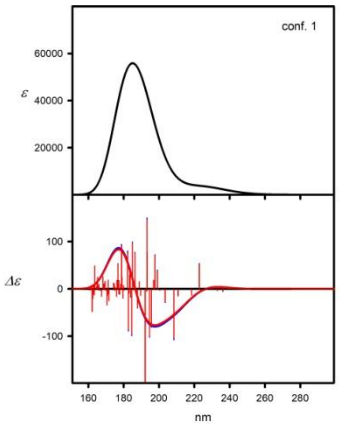

Figure S_29. UV and ECD spectra of the low-energy conformers of compound **6** calculated at the TD-M06-2X/6-311++G(d,p) level for structures optimized at the B3LYP/6-311++G(d,p) level. Wavelengths have not been corrected.

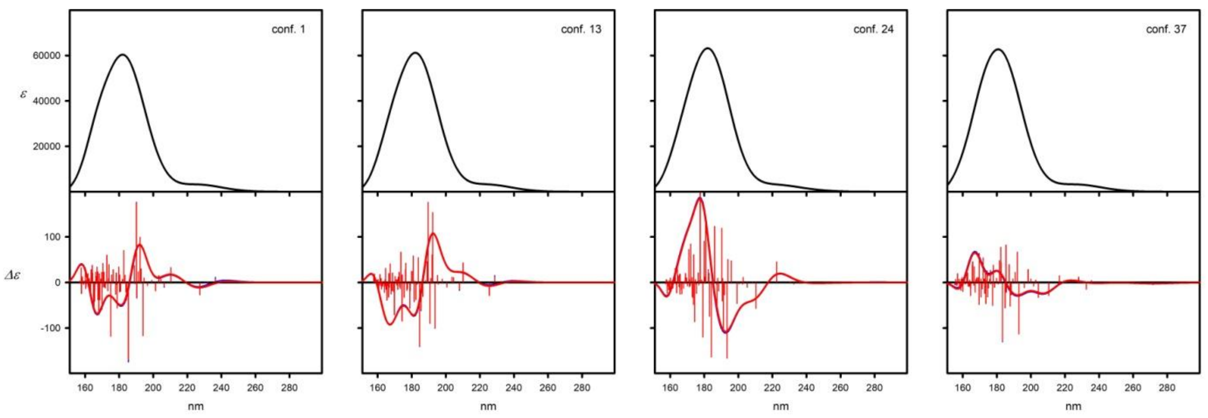

Figure S_30. UV and ECD spectra of the low-energy conformers of compound **7** calculated at the TD-CAM-B3LYP/6-311++G(d,p) level for structures optimized at the B3LYP/6-311++G(d,p) level. Wavelengths have not been corrected.

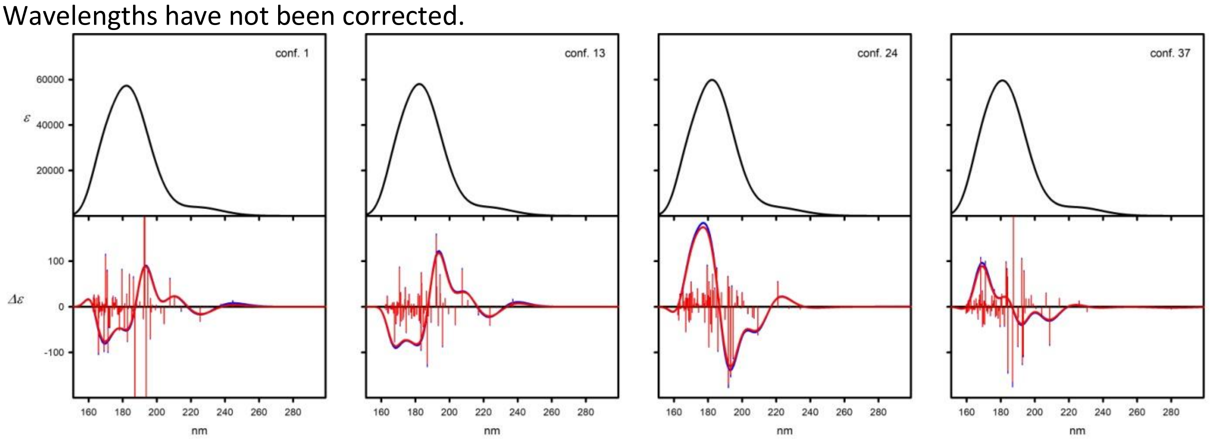

Figure S_31. UV and ECD spectra of the low-energy conformers of compound **7** calculated at the TD-M06-2X/6-311++G(d,p) level for structures optimized at the B3LYP/6-311++G(d,p) level. Wavelengths have not been corrected.

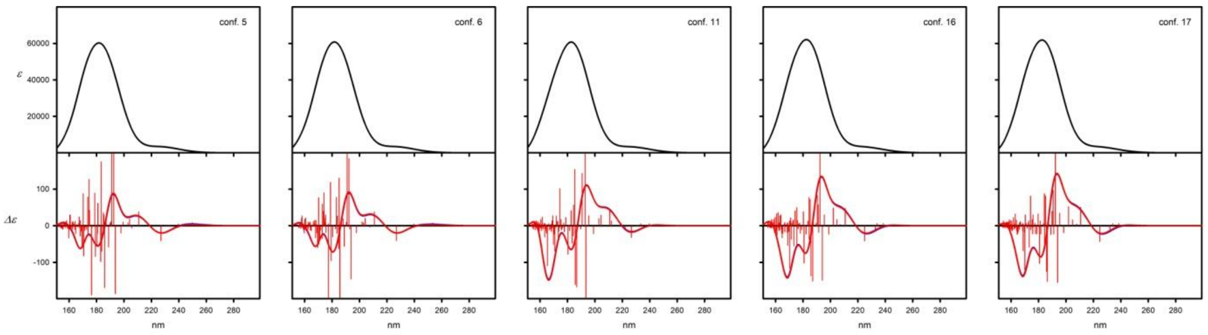

Figure S_32. UV and ECD spectra of the low-energy conformers of compound **8** calculated at the TD-CAM-B3LYP/6-311++G(d,p) level for structures optimized at the B3LYP/6-311++G(d,p) level. Wavelengths have not been corrected.

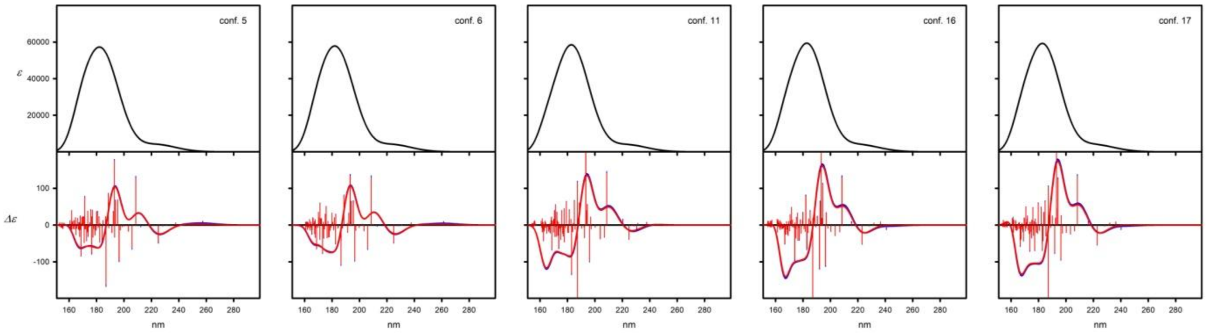

Figure S_33. UV and ECD spectra of the low-energy conformers of compound **8** calculated at the TD-M06-2X/6-311++G(d,p) level for structures optimized at the B3LYP/6-311++G(d,p) level. Wavelengths have not been corrected.

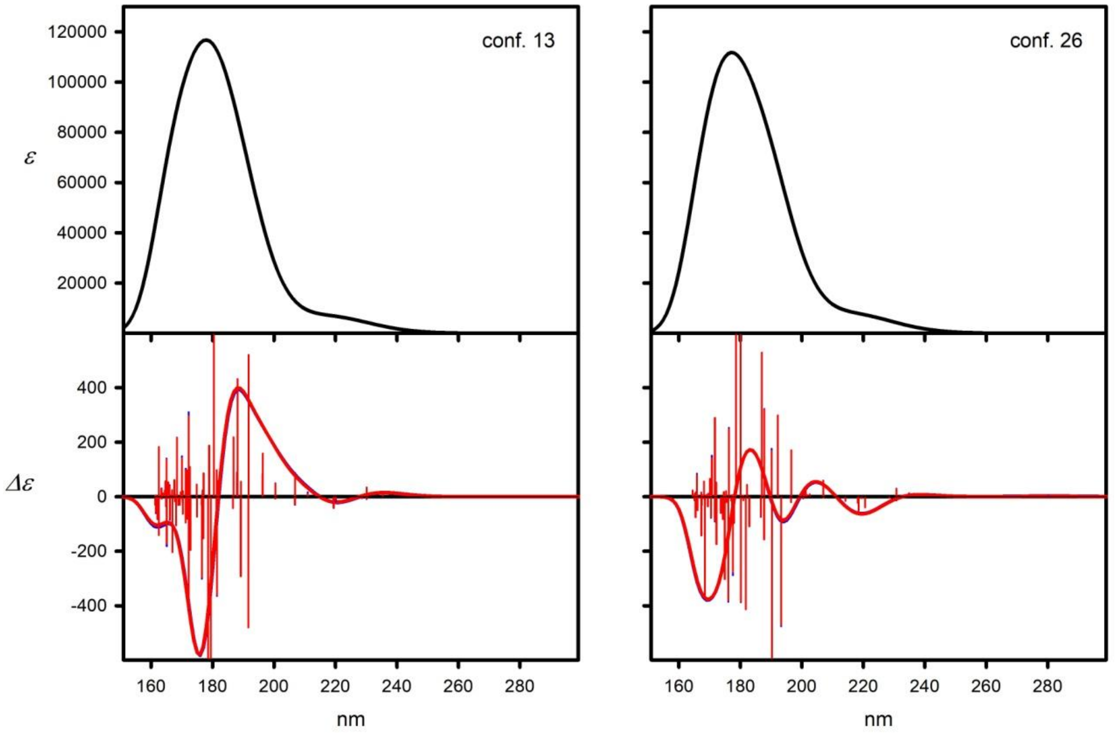

Figure S_34. UV and ECD spectra of the low-energy conformers of compound **9** calculated at the TD-CAM-B3LYP/6-311++G(d,p) level for structures optimized at the B3LYP/6-311G(d,p) level. Wavelengths have not been corrected.

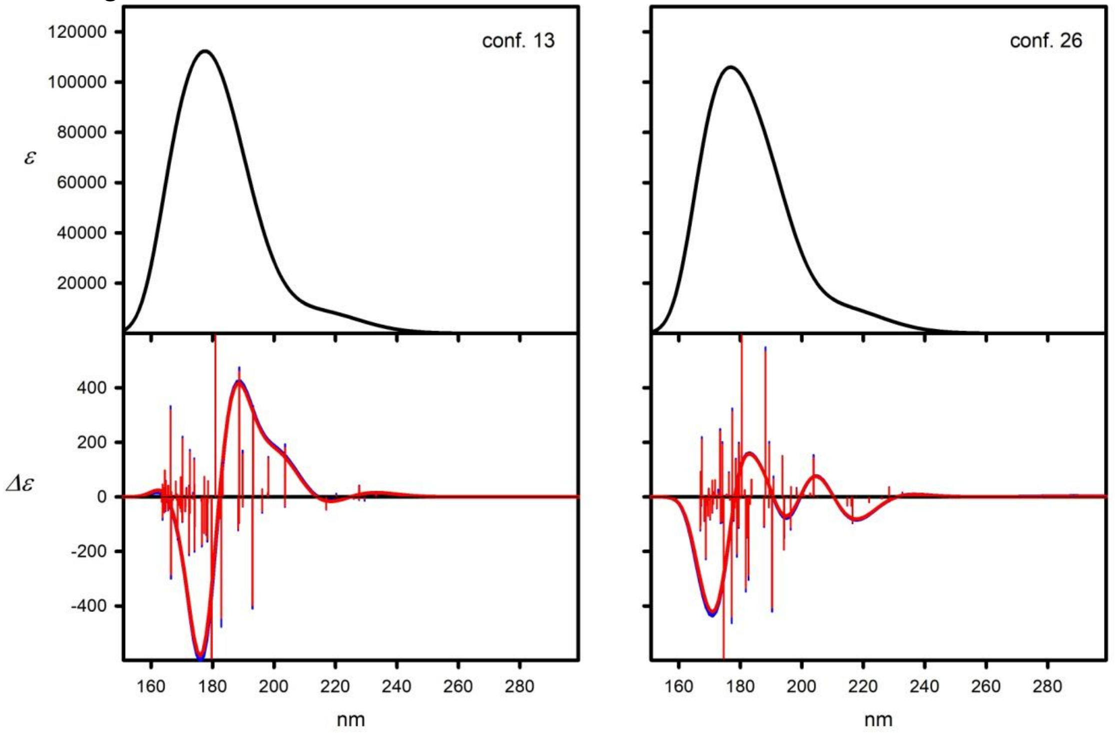

Figure S_35. UV and ECD spectra of the low-energy conformers of compound **9** calculated at the TD-M06-2X/6-311++G(d,p) level for structures optimized at the B3LYP/6-311G(d,p) level. Wavelengths have not been corrected.

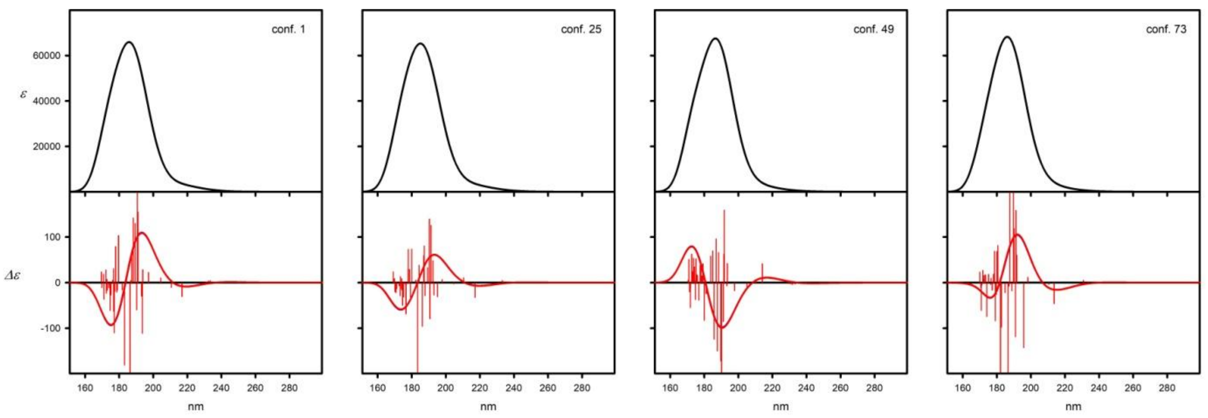

Figure S_36. UV and ECD spectra of the low-energy conformers of compound **11** calculated at the TD-CAM-B3LYP/6-311++G(d,p) level for structures optimized at the B3LYP/6-311++G(d,p) level. Wavelengths have not been corrected.

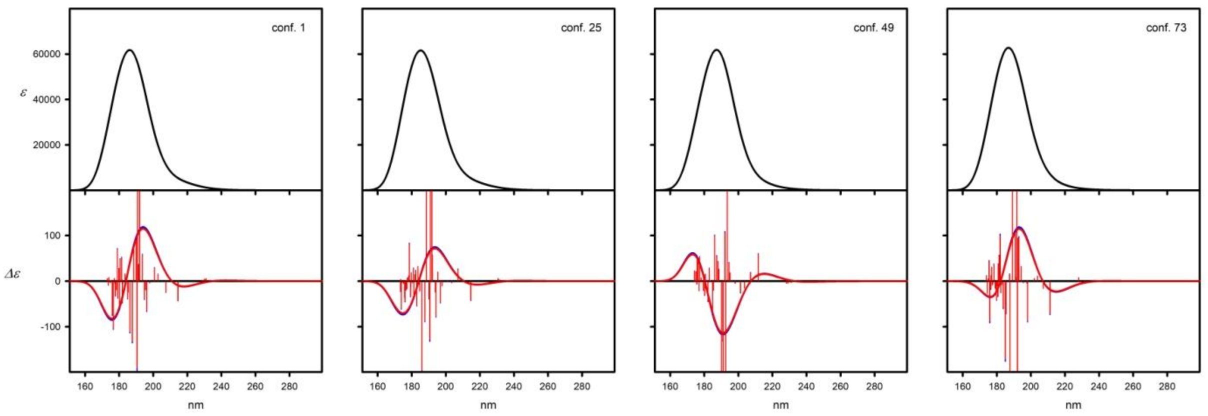

Figure S_37. UV and ECD spectra of the low-energy conformers of compound **11** calculated at the TD-M06-2X/6-311++G(d,p) level for structures optimized at the B3LYP/6-311++G(d,p) level. Wavelengths have not been corrected.

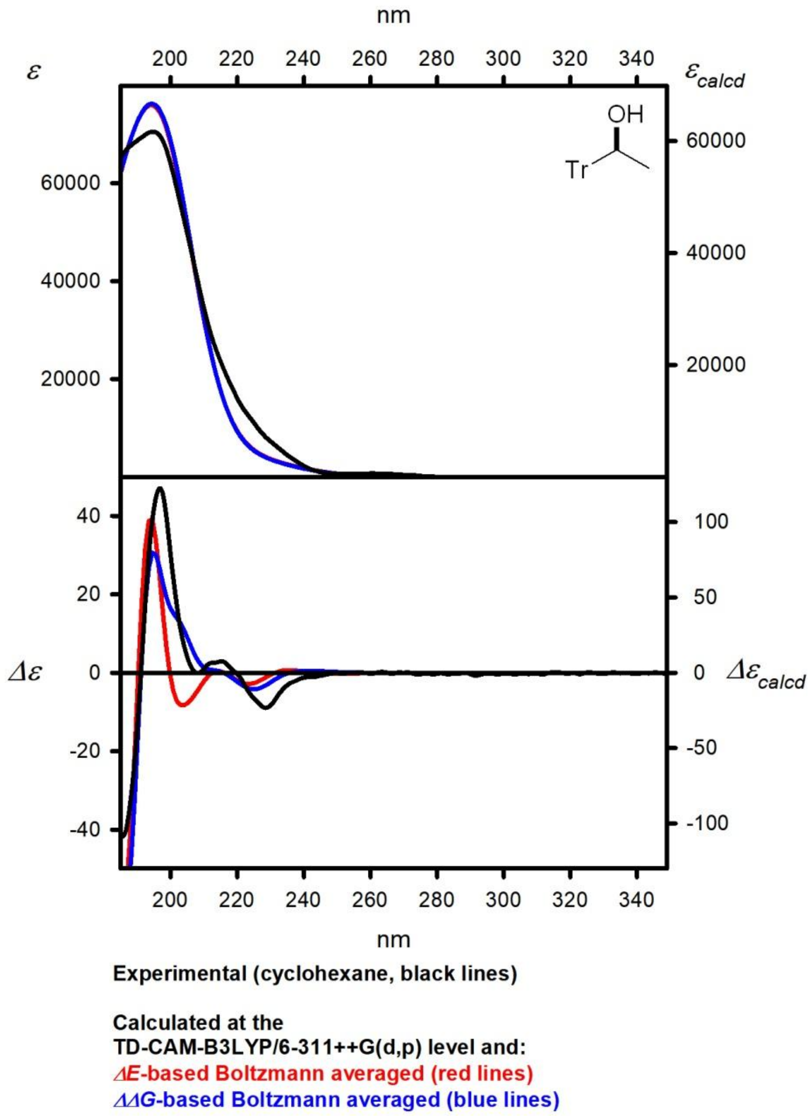

Figure S_38. UV (upper panel) and ECD (lower panel) spectra of **1a**, measured in cyclohexane (solid black lines), calculated at the TD-CAM-B3LYP/6-311++G(d,p) level and *ΔE*-based (solid red lines) and *ΔΔG*-based (solid red lines) Boltzmann averaged. Wavelengths have been corrected to match experimental UV maximum.

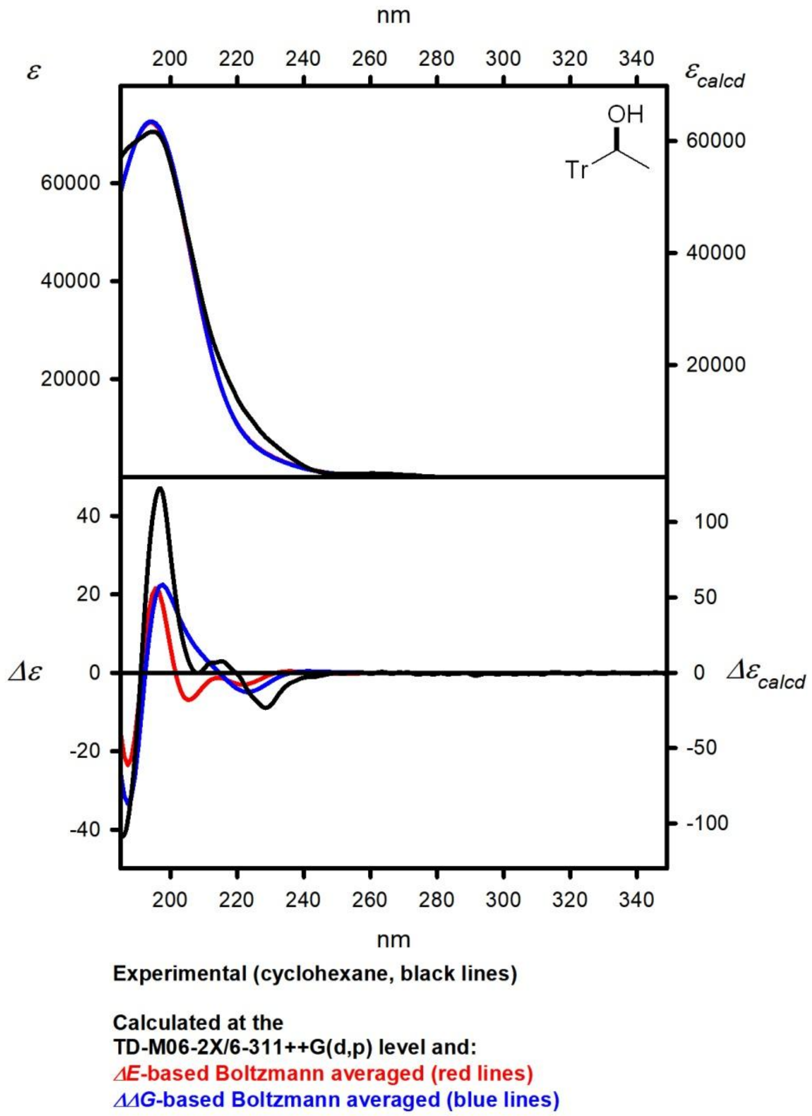

Figure S_39. UV (upper panel) and ECD (lower panel) spectra of **1a**, measured in cyclohexane (solid black lines), calculated at the TD-M06-2X/6-311++G(d,p) level and *ΔE*-based (solid red lines) and *ΔΔG*-based (solid red lines) Boltzmann averaged. Wavelengths have been corrected to match experimental UV maximum.

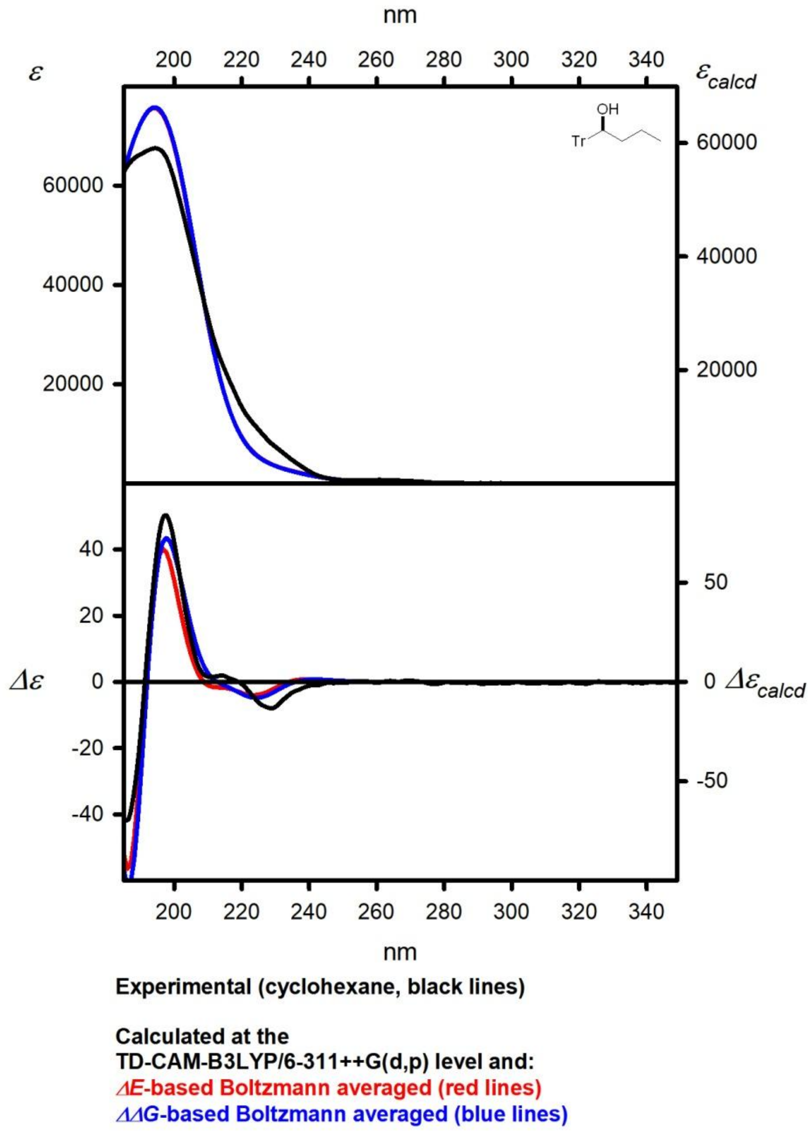

Figure S_40. UV (upper panel) and ECD (lower panel) spectra of **1b**, measured in cyclohexane (solid black lines), calculated at the TD-CAM-B3LYP/6-311++G(d,p) level and *ΔE*-based (solid red lines) and *ΔΔG*-based (solid red lines) Boltzmann averaged. Wavelengths have been corrected to match experimental UV maximum.

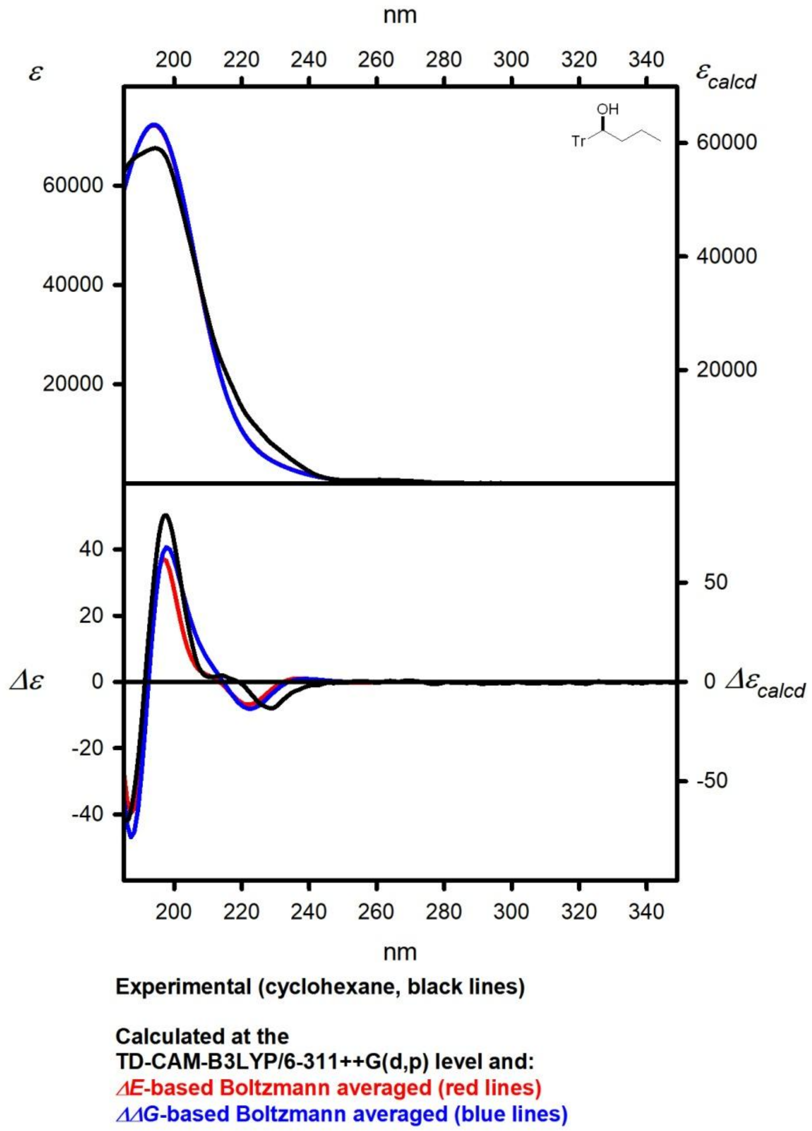

Figure S_41. UV (upper panel) and ECD (lower panel) spectra of **1b**, measured in cyclohexane (solid black lines), calculated at the TD-M06-2X/6-311++G(d,p) level and *ΔE*-based (solid red lines) and *ΔΔG*-based (solid red lines) Boltzmann averaged. Wavelengths have been corrected to match experimental UV maximum.

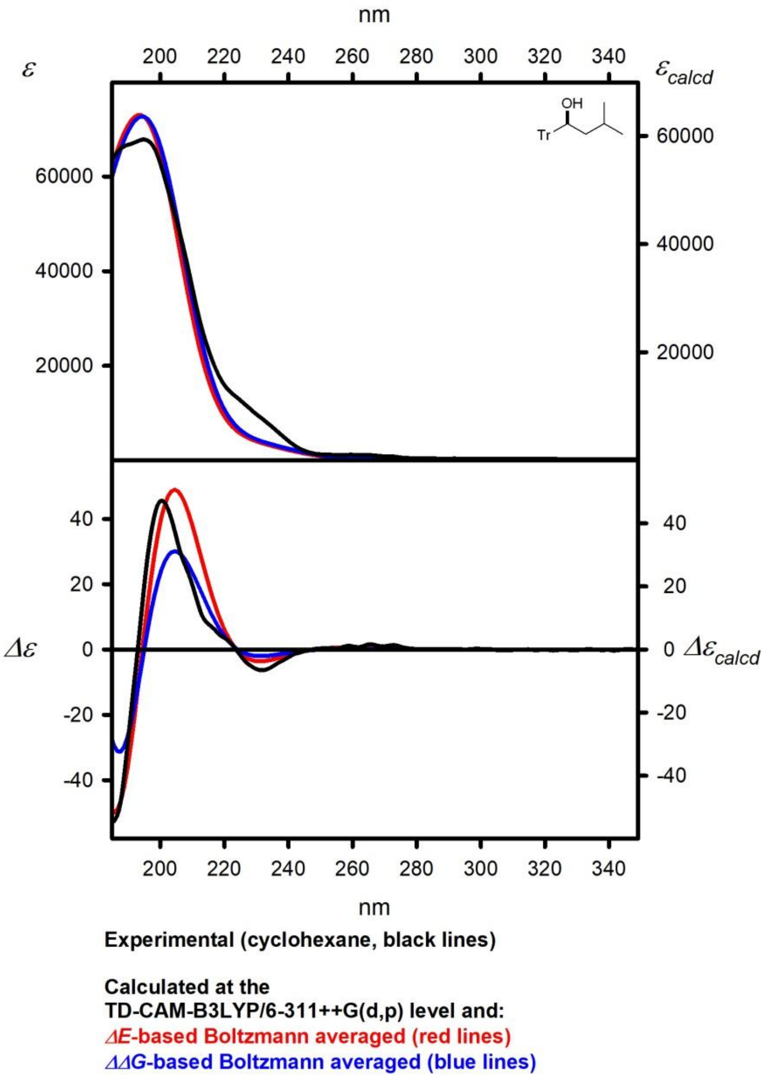

Figure S_42. UV (upper panel) and ECD (lower panel) spectra of **1c**, measured in cyclohexane (solid black lines), calculated at the TD-CAM-B3LYP/6-311++G(d,p) level and *ΔE*-based (solid red lines) and *ΔΔG*-based (solid red lines) Boltzmann averaged. Wavelengths have been corrected to match experimental UV maximum.

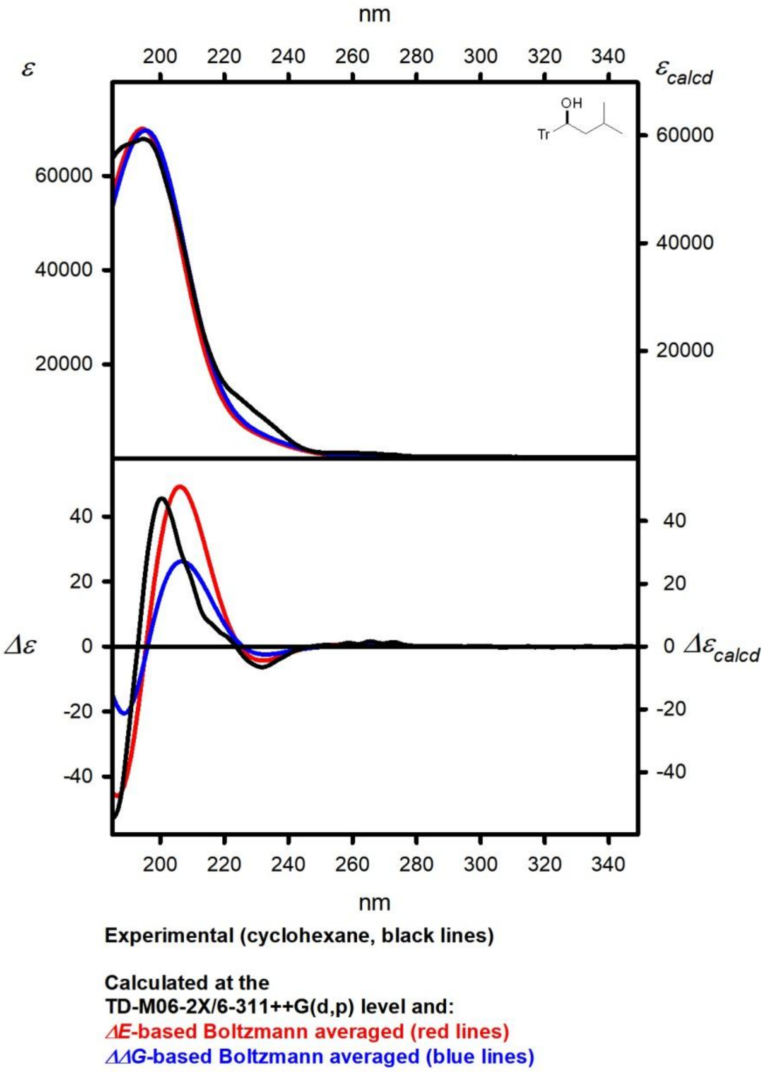

Figure S_43. UV (upper panel) and ECD (lower panel) spectra of **1c**, measured in cyclohexane (solid black lines), calculated at the TD-M06-2X/6-311++G(d,p) level and *ΔE*-based (solid red lines) and *ΔΔG*-based (solid red lines) Boltzmann averaged. Wavelengths have been corrected to match experimental UV maximum.

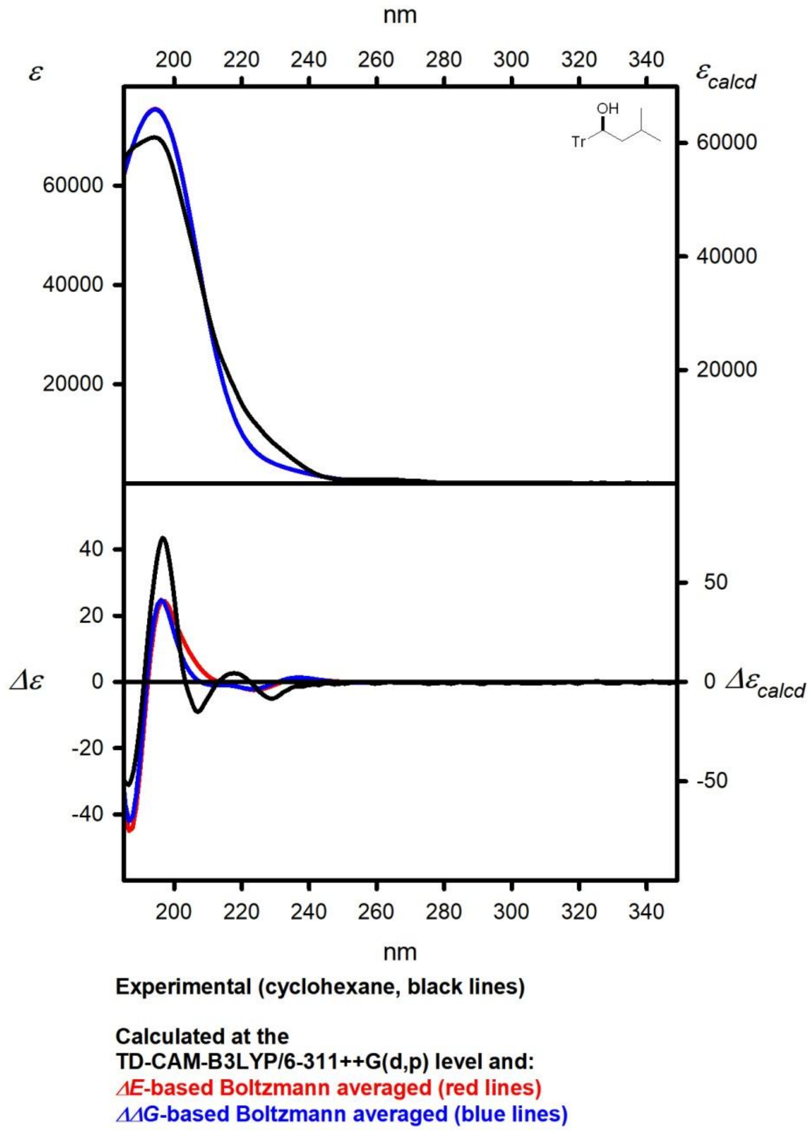

Figure S_44. UV (upper panel) and ECD (lower panel) spectra of **1d**, measured in cyclohexane (solid black lines), calculated at the TD-CAM-B3LYP/6-311++G(d,p) level and *ΔE*-based (solid red lines) and *ΔΔG*-based (solid red lines) Boltzmann averaged. Wavelengths have been corrected to match experimental UV maximum.

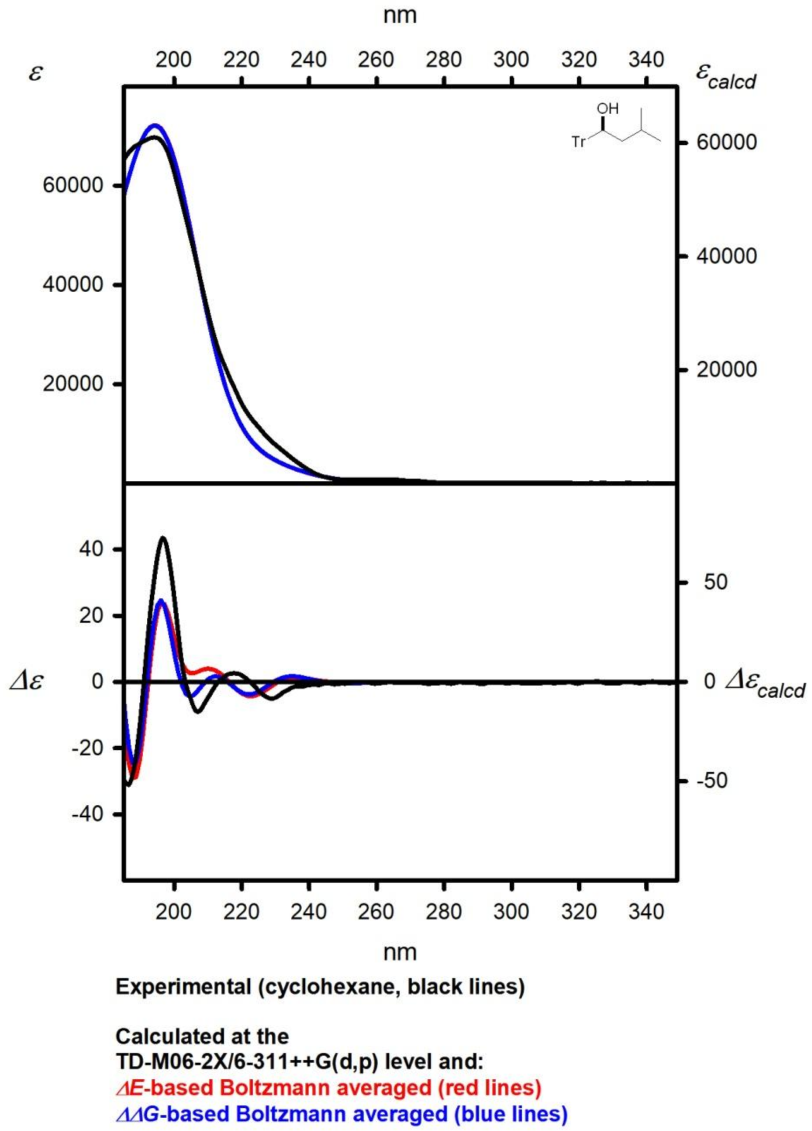

Figure S_45. UV (upper panel) and ECD (lower panel) spectra of **1d**, measured in cyclohexane (solid black lines), calculated at the TD-M06-2X/6-311++G(d,p) level and *ΔE*-based (solid red lines) and *ΔΔG*-based (solid red lines) Boltzmann averaged. Wavelengths have been corrected to match experimental UV maximum.

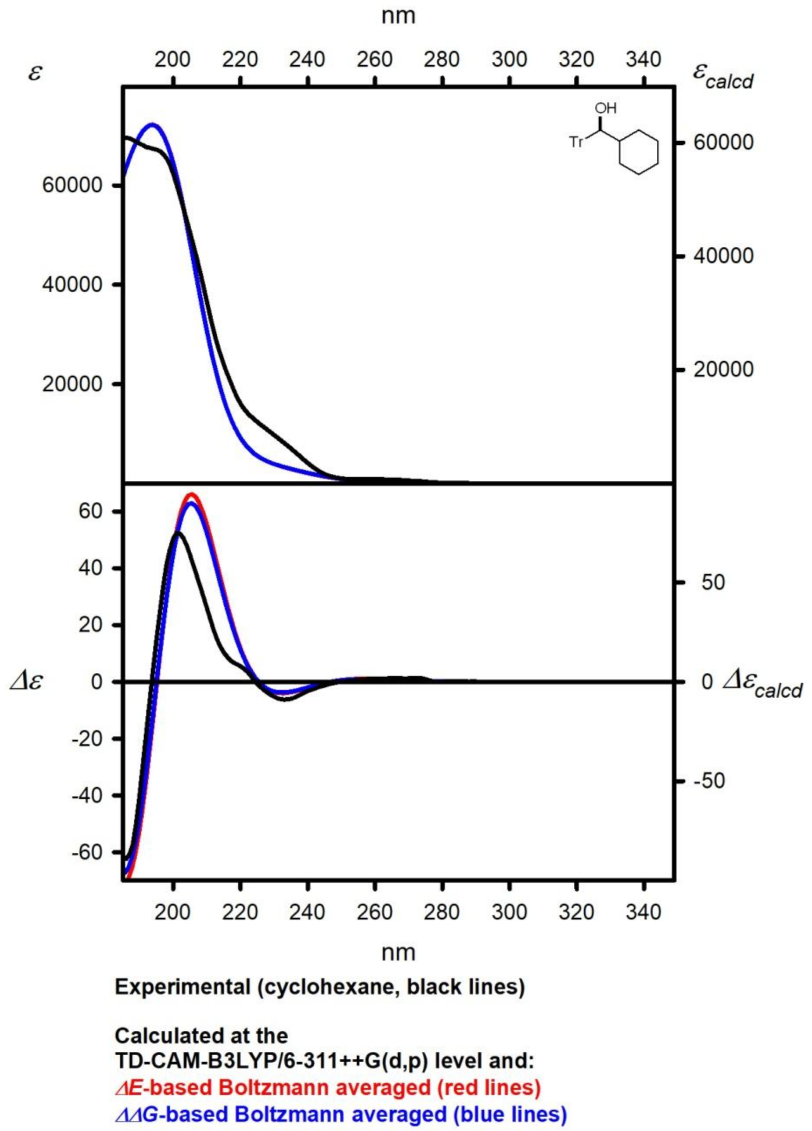

Figure S_46. UV (upper panel) and ECD (lower panel) spectra of **1e**, measured in cyclohexane (solid black lines), calculated at the TD-CAM-B3LYP/6-311++G(d,p) level and *ΔE*-based (solid red lines) and *ΔΔG*-based (solid red lines) Boltzmann averaged. Wavelengths have been corrected to match experimental UV maximum.

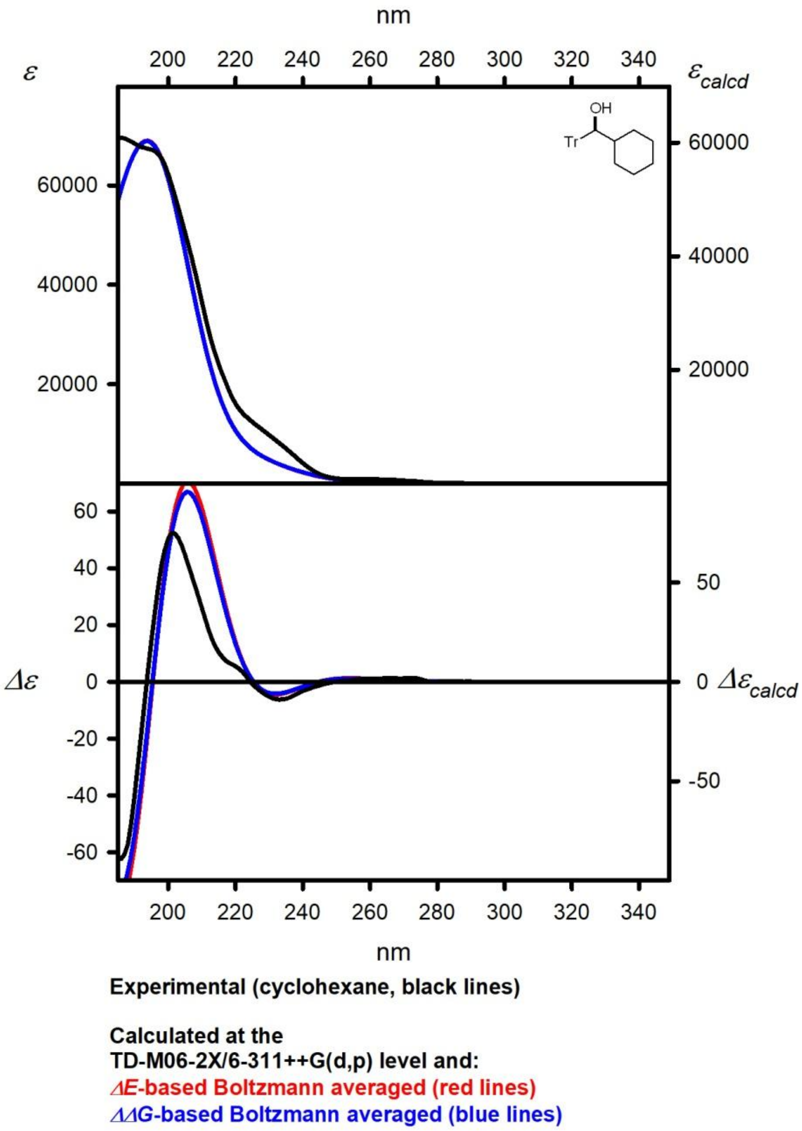

Figure S_47. UV (upper panel) and ECD (lower panel) spectra of **1e**, measured in cyclohexane (solid black lines), calculated at the TD-M06-2X/6-311++G(d,p) level and *ΔE*-based (solid red lines) and *ΔΔG*-based (solid red lines) Boltzmann averaged. Wavelengths have been corrected to match experimental UV maximum.

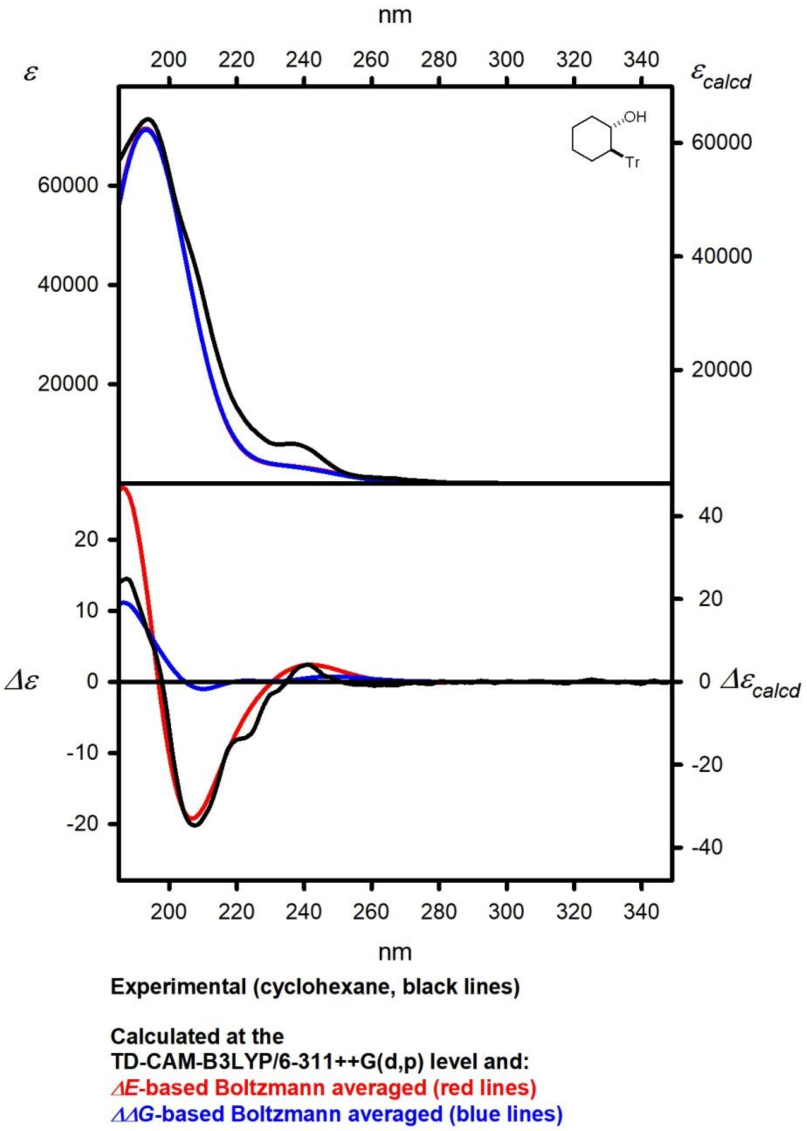

Figure S_48. UV (upper panel) and ECD (lower panel) spectra of **3**, measured in cyclohexane (solid black lines), calculated at the TD-CAM-B3LYP/6-311++G(d,p) level and *ΔE*-based (solid red lines) and *ΔΔG*-based (solid red lines) Boltzmann averaged. Wavelengths have been corrected to match experimental UV maximum.

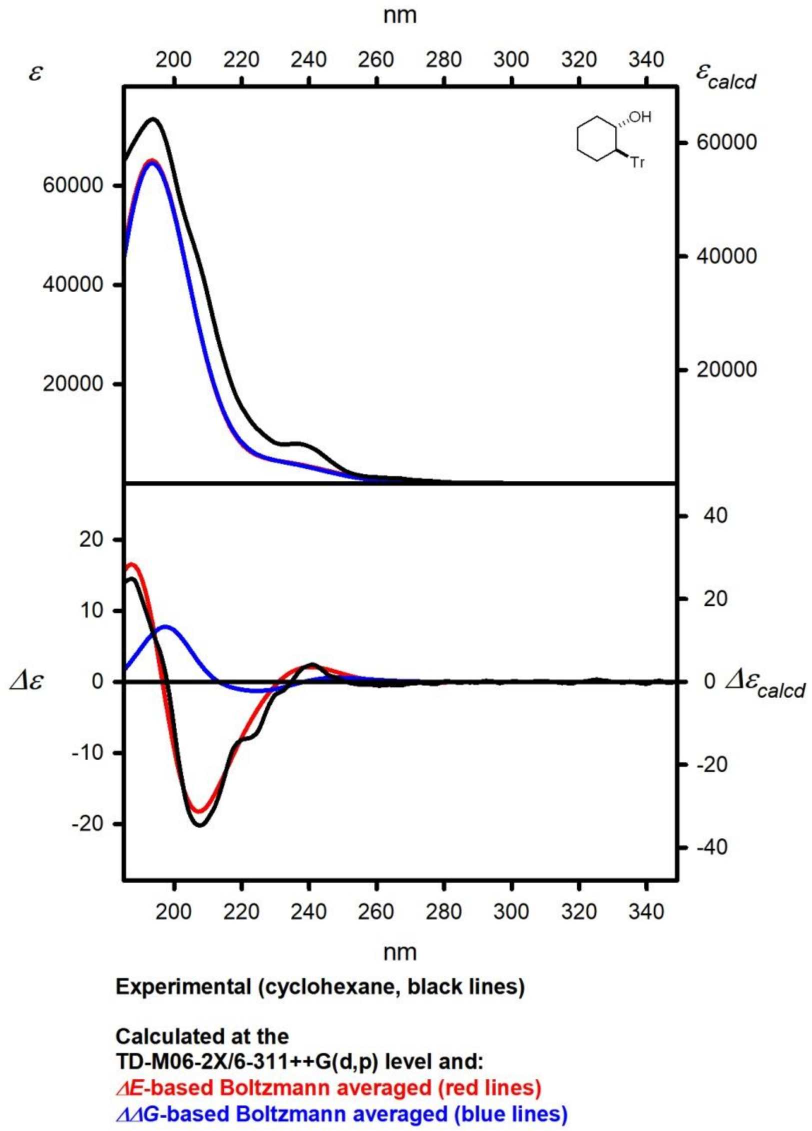

Figure S_49. UV (upper panel) and ECD (lower panel) spectra of **3**, measured in cyclohexane (solid black lines), calculated at the TD-M06-2X/6-311++G(d,p) level and *ΔE*-based (solid red lines) and *ΔΔG*-based (solid red lines) Boltzmann averaged. Wavelengths have been corrected to match experimental UV maximum.

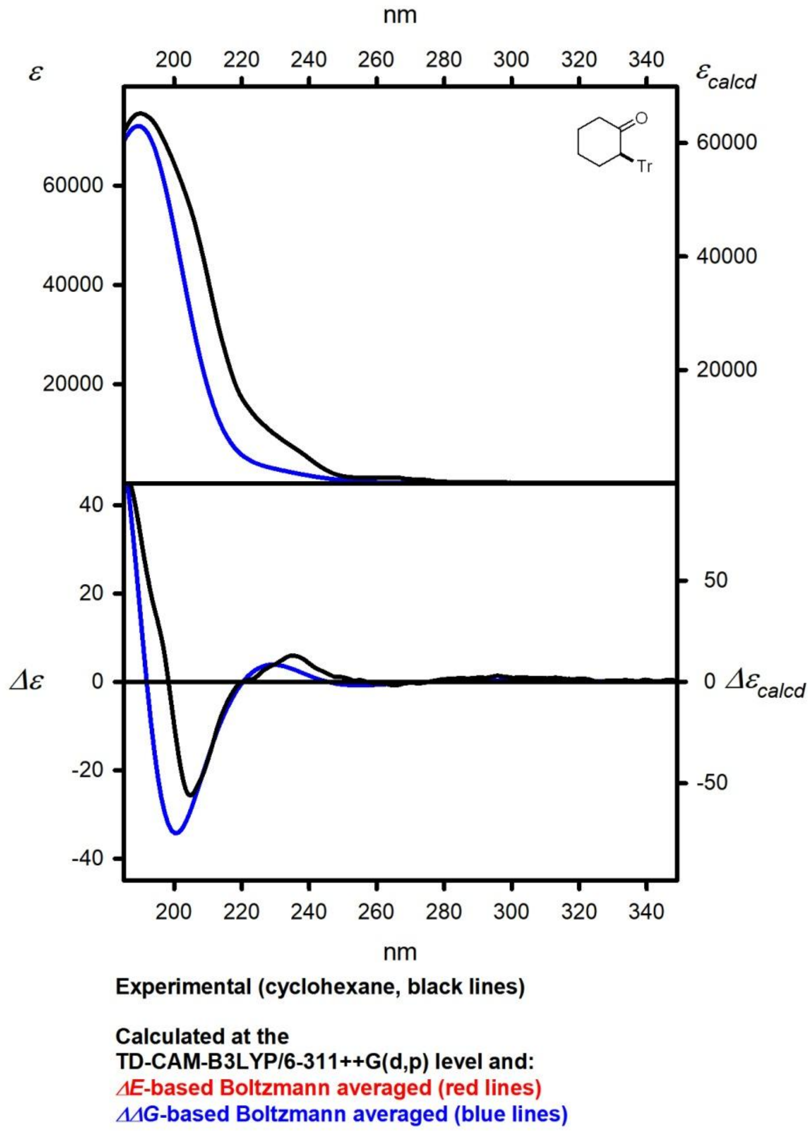

Figure S_50. UV (upper panel) and ECD (lower panel) spectra of **4**, measured in cyclohexane (solid black lines), calculated at the TD-CAM-B3LYP/6-311++G(d,p) level and *ΔE*-based (solid red lines) and *ΔΔG*-based (solid red lines) Boltzmann averaged. Wavelengths have been corrected to match experimental UV maximum.

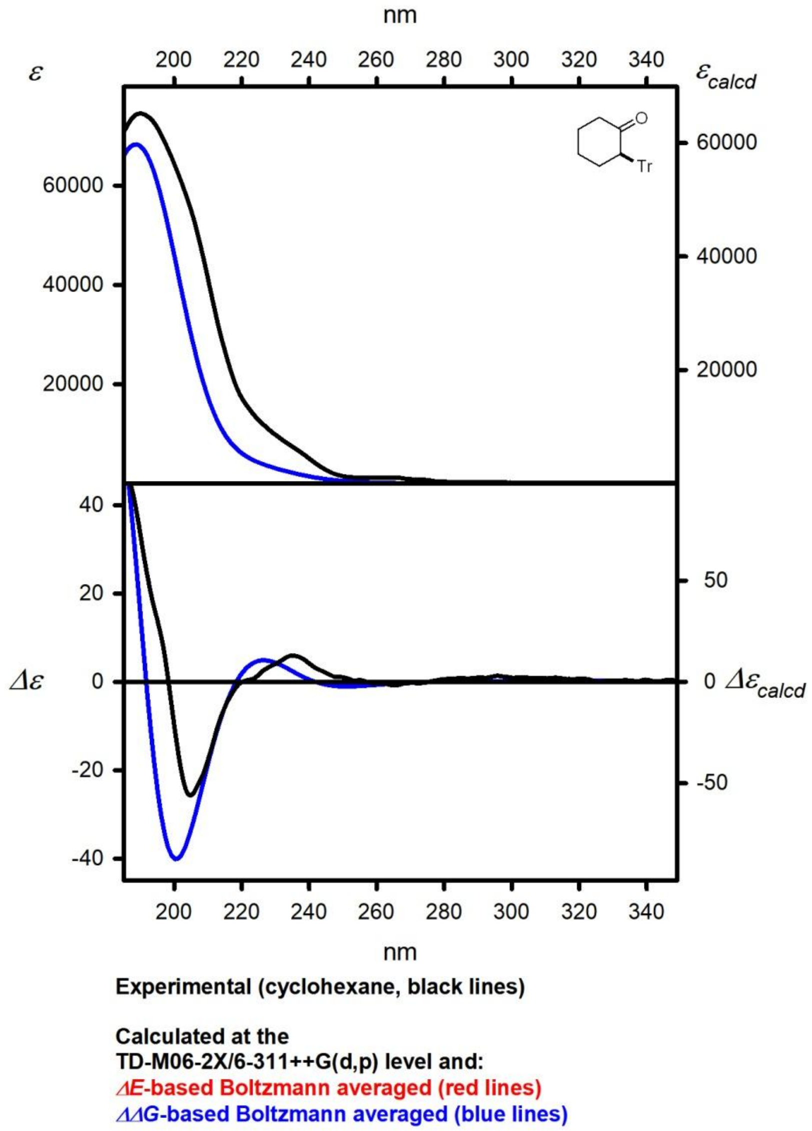

Figure S_51. UV (upper panel) and ECD (lower panel) spectra of **4**, measured in cyclohexane (solid black lines), calculated at the TD-M06-2X/6-311++G(d,p) level and *ΔE*-based (solid red lines) and *ΔΔG*-based (solid red lines) Boltzmann averaged. Wavelengths have been corrected to match experimental UV maximum.

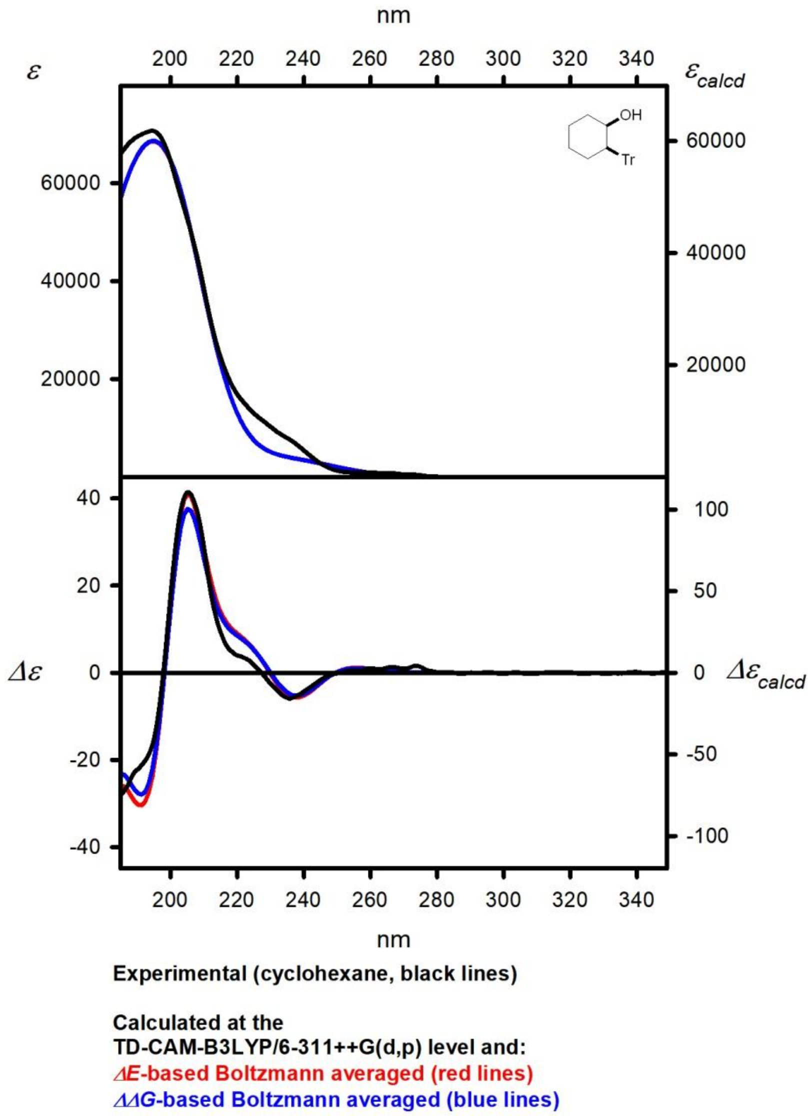

Figure S_52. UV (upper panel) and ECD (lower panel) spectra of **5**, measured in cyclohexane (solid black lines), calculated at the TD-CAM-B3LYP/6-311++G(d,p) level and *ΔE*-based (solid red lines) and *ΔΔG*-based (solid red lines) Boltzmann averaged. Wavelengths have been corrected to match experimental UV maximum.

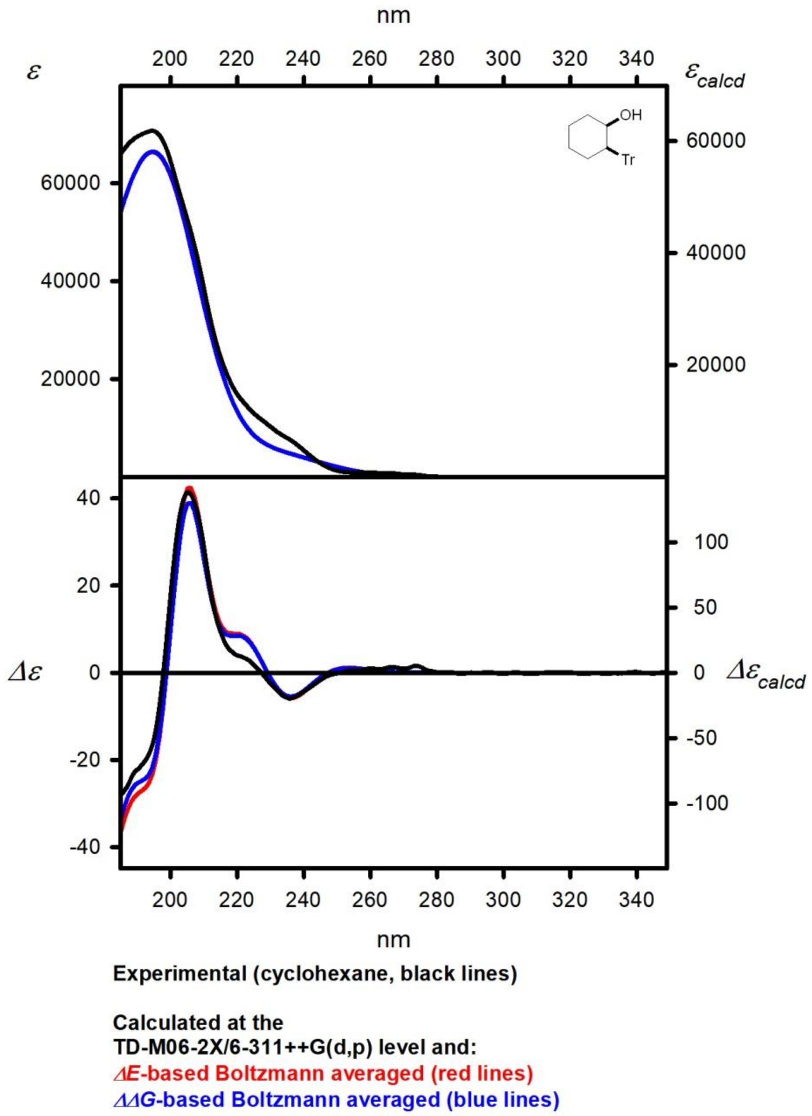

Figure S_53. UV (upper panel) and ECD (lower panel) spectra of **5**, measured in cyclohexane (solid black lines), calculated at the TD-M06-2X/6-311++G(d,p) level and *ΔE*-based (solid red lines) and *ΔΔG*-based (solid red lines) Boltzmann averaged. Wavelengths have been corrected to match experimental UV maximum.

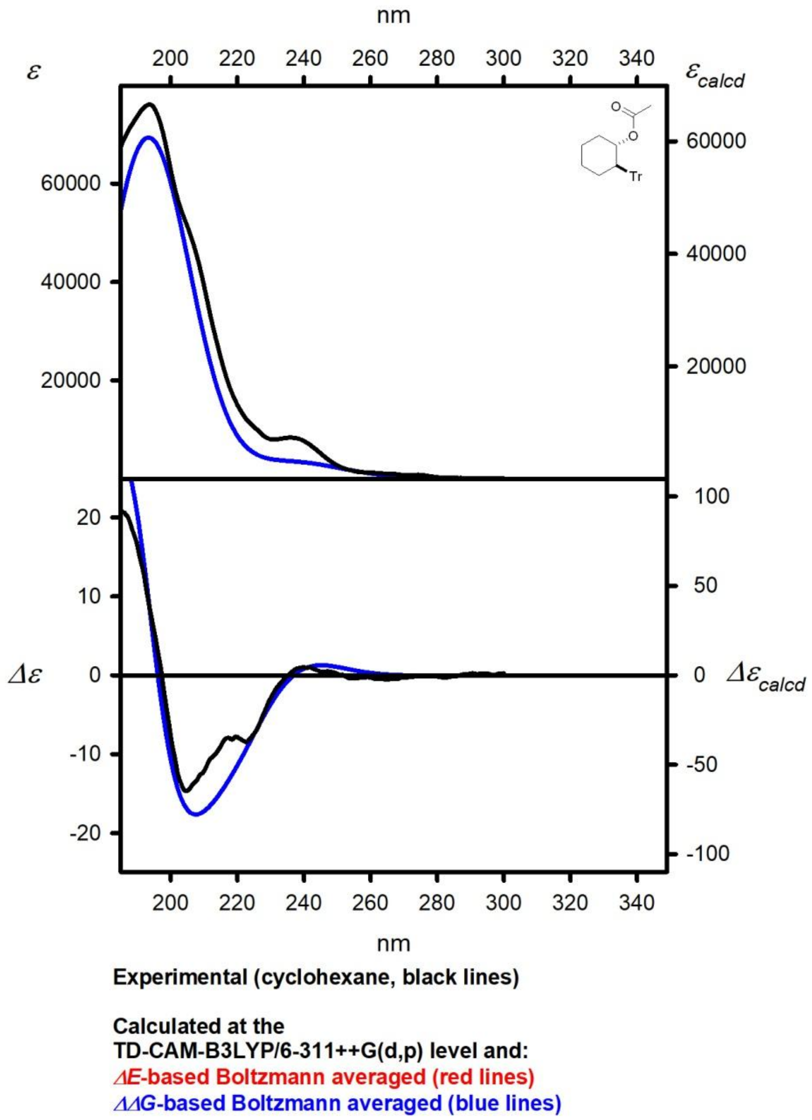

Figure S_54. UV (upper panel) and ECD (lower panel) spectra of **6**, measured in cyclohexane (solid black lines), calculated at the TD-CAM-B3LYP/6-311++G(d,p) level and *ΔE*-based (solid red lines) and *ΔΔG*-based (solid red lines) Boltzmann averaged. Wavelengths have been corrected to match experimental UV maximum.

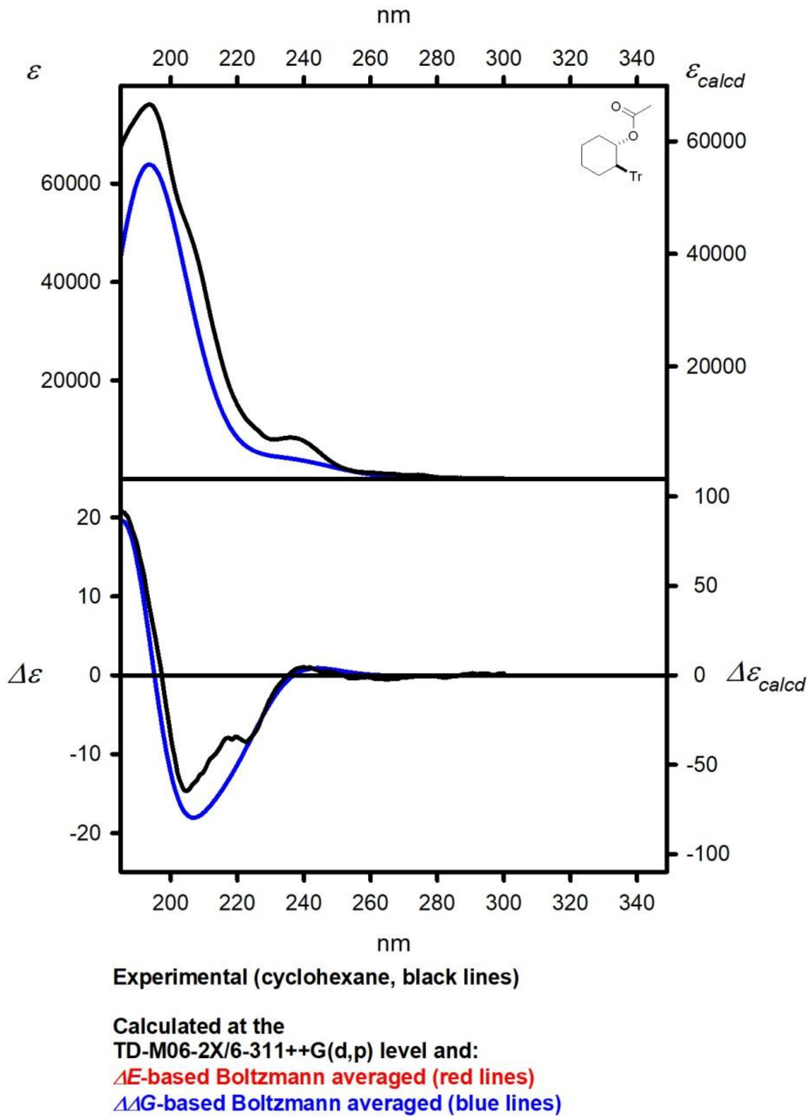

Figure S_55. UV (upper panel) and ECD (lower panel) spectra of **6**, measured in cyclohexane (solid black lines), calculated at the TD-M06-2X/6-311++G(d,p) level and *ΔE*-based (solid red lines) and *ΔΔG*-based (solid red lines) Boltzmann averaged. Wavelengths have been corrected to match experimental UV maximum.

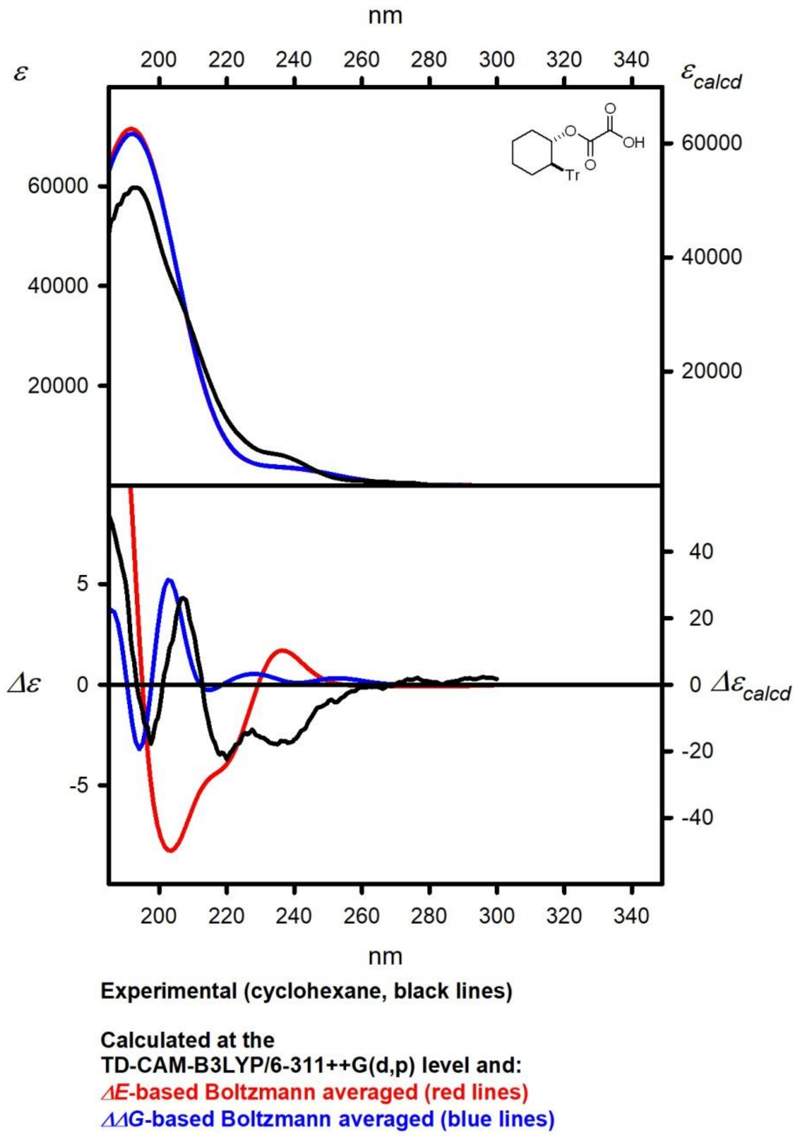

Figure S_56. UV (upper panel) and ECD (lower panel) spectra of **7**, measured in cyclohexane (solid black lines), calculated at the TD-CAM-B3LYP/6-311++G(d,p) level and *ΔE*-based (solid red lines) and *ΔΔG*-based (solid red lines) Boltzmann averaged. Wavelengths have been corrected to match experimental UV maximum.

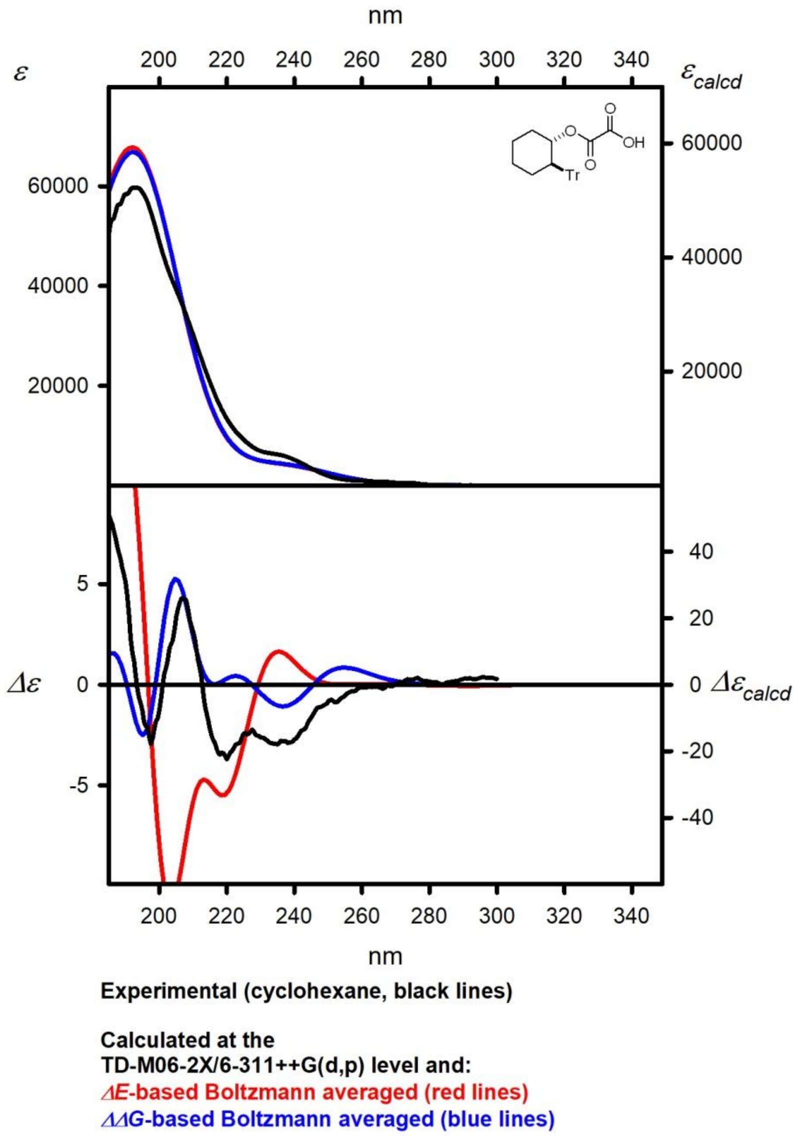

Figure S_57. UV (upper panel) and ECD (lower panel) spectra of **7**, measured in cyclohexane (solid black lines), calculated at the TD-M06-2X/6-311++G(d,p) level and *ΔE*-based (solid red lines) and *ΔΔG*-based (solid red lines) Boltzmann averaged. Wavelengths have been corrected to match experimental UV maximum.

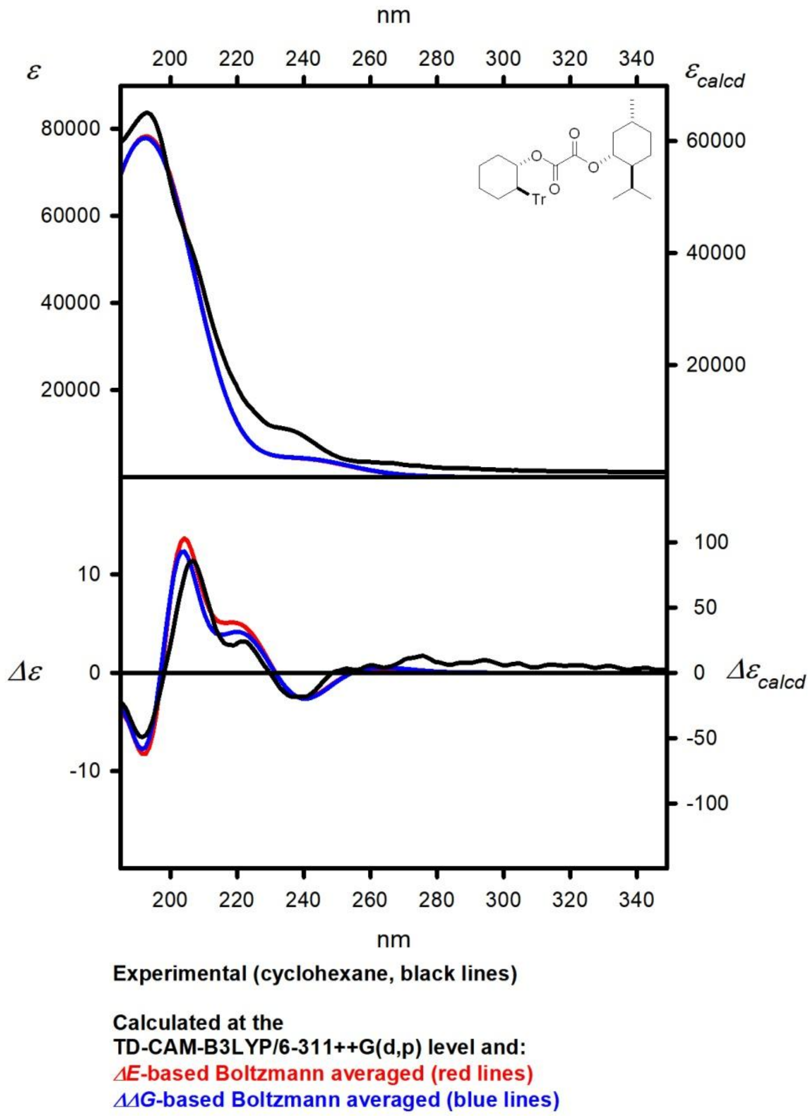

Figure S_58. UV (upper panel) and ECD (lower panel) spectra of **8**, measured in cyclohexane (solid black lines), calculated at the TD-CAM-B3LYP/6-311++G(d,p) level and *ΔE*-based (solid red lines) and *ΔΔG*-based (solid red lines) Boltzmann averaged. Wavelengths have been corrected to match experimental UV maximum.

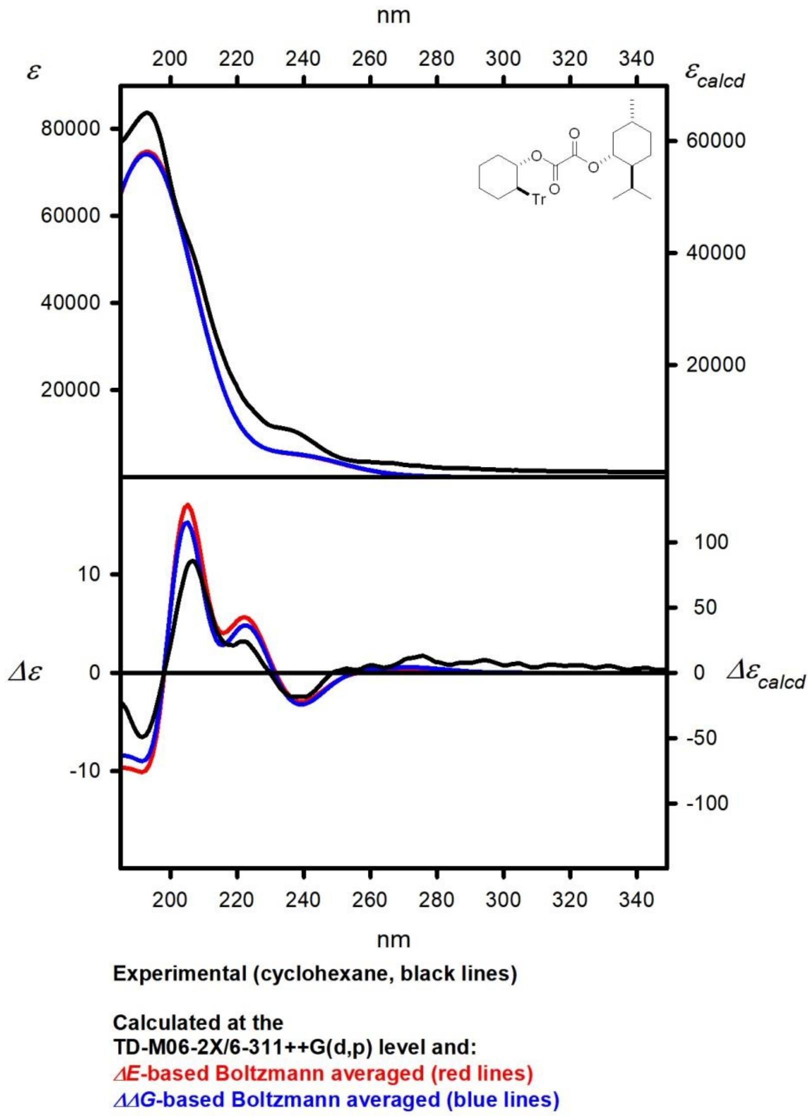

Figure S_59. UV (upper panel) and ECD (lower panel) spectra of **8**, measured in cyclohexane (solid black lines), calculated at the TD-M06-2X/6-311++G(d,p) level and *ΔE*-based (solid red lines) and *ΔΔG*-based (solid red lines) Boltzmann averaged. Wavelengths have been corrected to match experimental UV maximum.

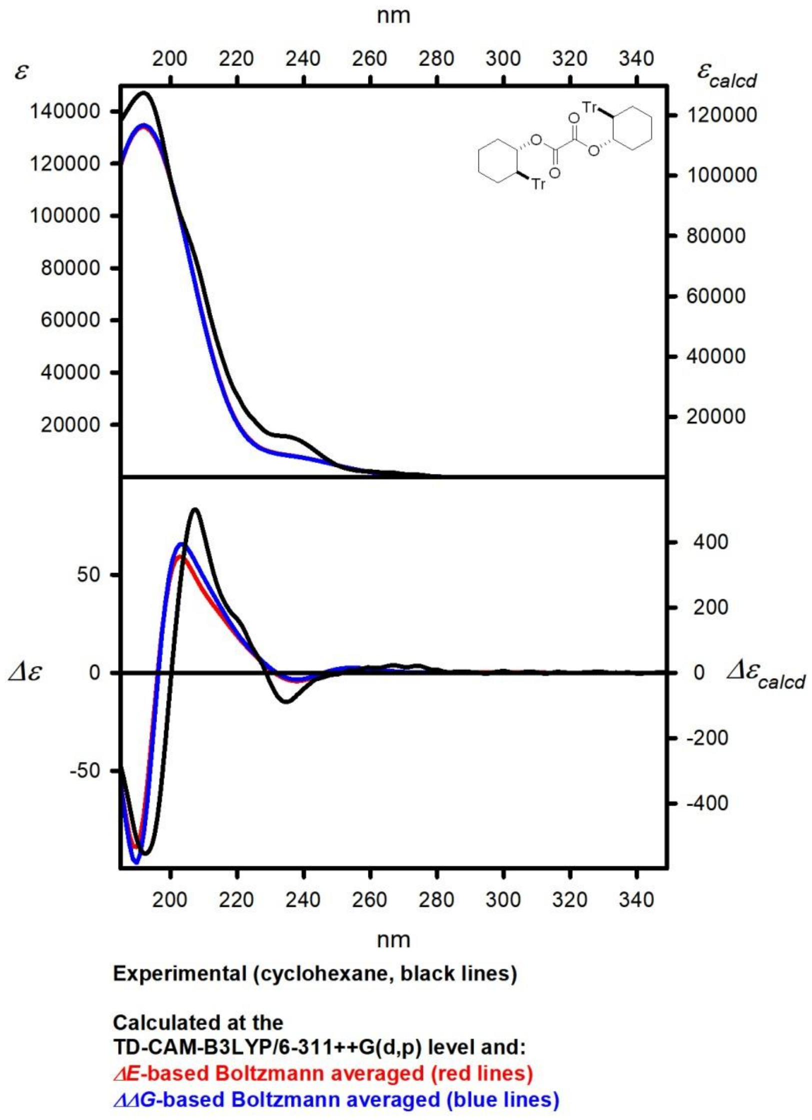

Figure S_60. UV (upper panel) and ECD (lower panel) spectra of **9**, measured in cyclohexane (solid black lines), calculated at the TD-CAM-B3LYP/6-311++G(d,p) level and *ΔE*-based (solid red lines) and *ΔΔG*-based (solid red lines) Boltzmann averaged. Wavelengths have been corrected to match experimental UV maximum.

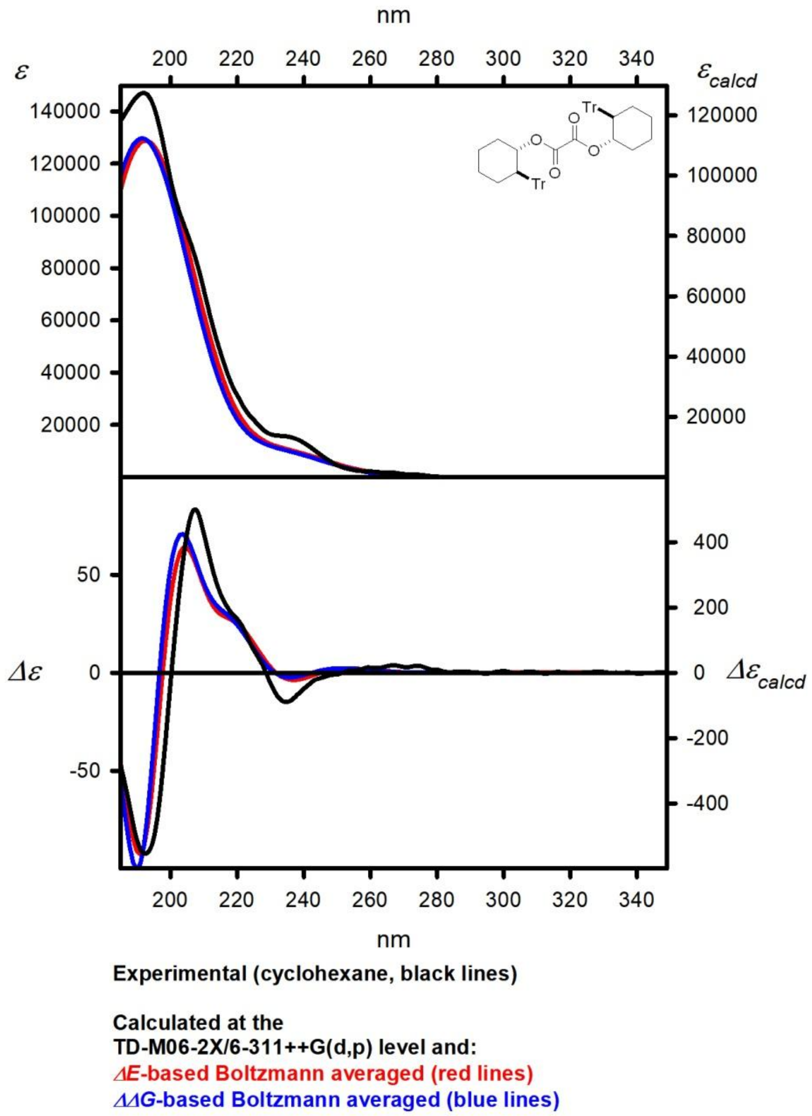

Figure S_61. UV (upper panel) and ECD (lower panel) spectra of **9**, measured in cyclohexane (solid black lines), calculated at the TD-M06-2X/6-311++G(d,p) level and *ΔE*-based (solid red lines) and *ΔΔG*-based (solid red lines) Boltzmann averaged. Wavelengths have been corrected to match experimental UV maximum.

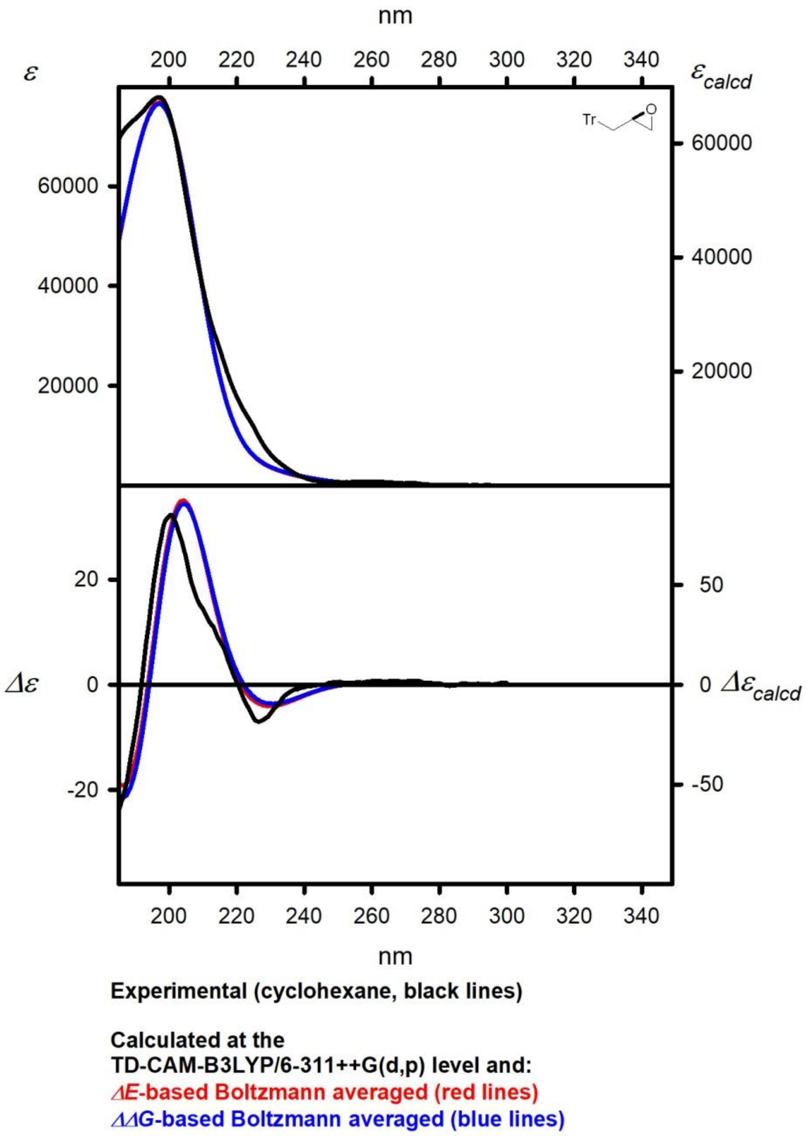

Figure S_62. UV (upper panel) and ECD (lower panel) spectra of **11**, measured in cyclohexane (solid black lines), calculated at the TD-CAM-B3LYP/6-311++G(d,p) level and *ΔE*-based (solid red lines) and *ΔΔG*-based (solid red lines) Boltzmann averaged. Wavelengths have been corrected to match experimental UV maximum.

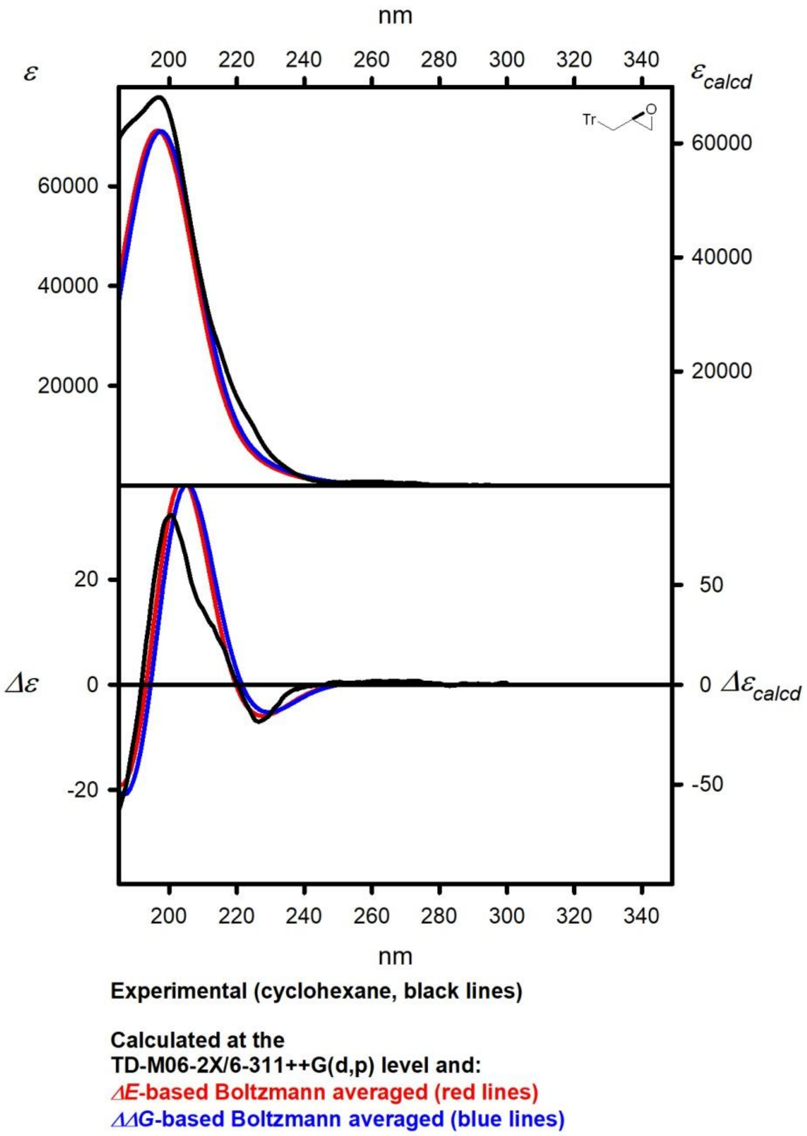

Figure S_63. UV (upper panel) and ECD (lower panel) spectra of **11**, measured in cyclohexane (solid black lines), calculated at the TD-M06-2X/6-311++G(d,p) level and *ΔE*-based (solid red lines) and *ΔΔG*-based (solid red lines) Boltzmann averaged. Wavelengths have been corrected to match experimental UV maximum.

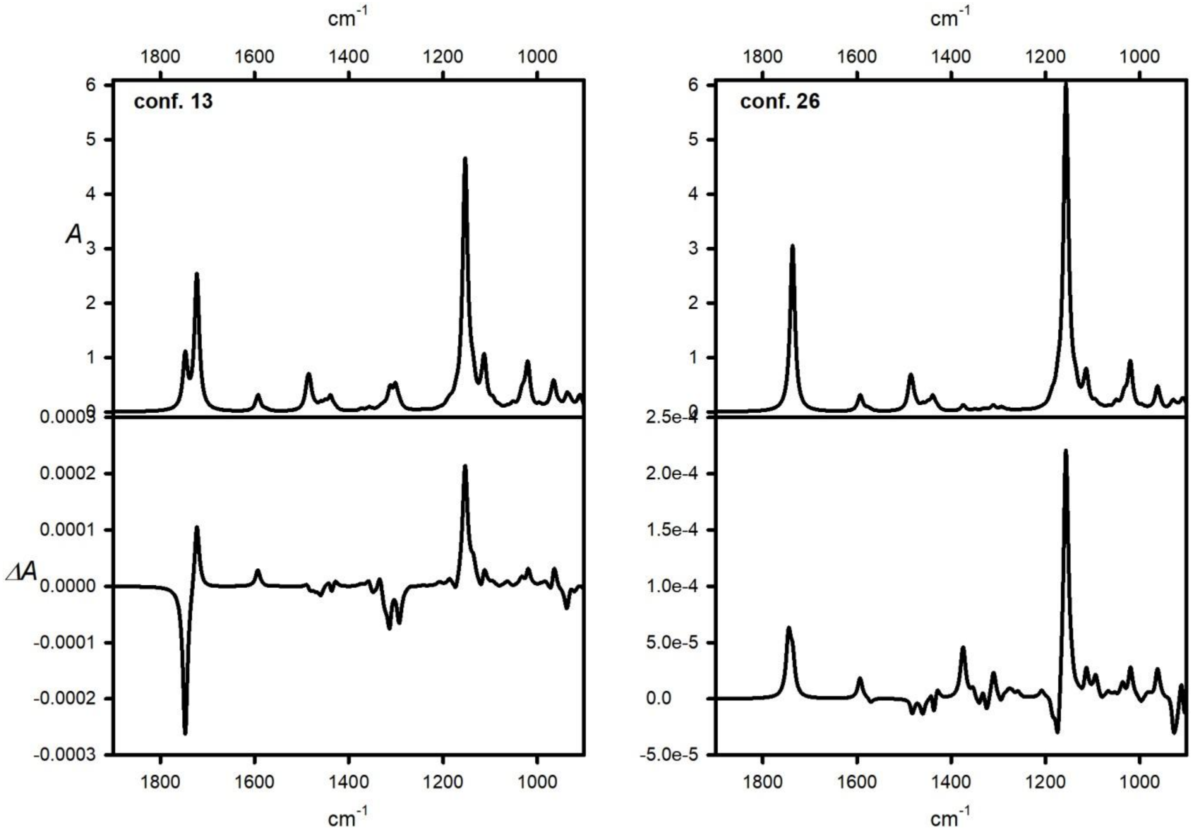

Figure S_64. IR (upper panel) and VCD (lower panel) spectra of **9**, calculated at the IEFPCM/B3LYP/6-311G(d,p) level of theory.

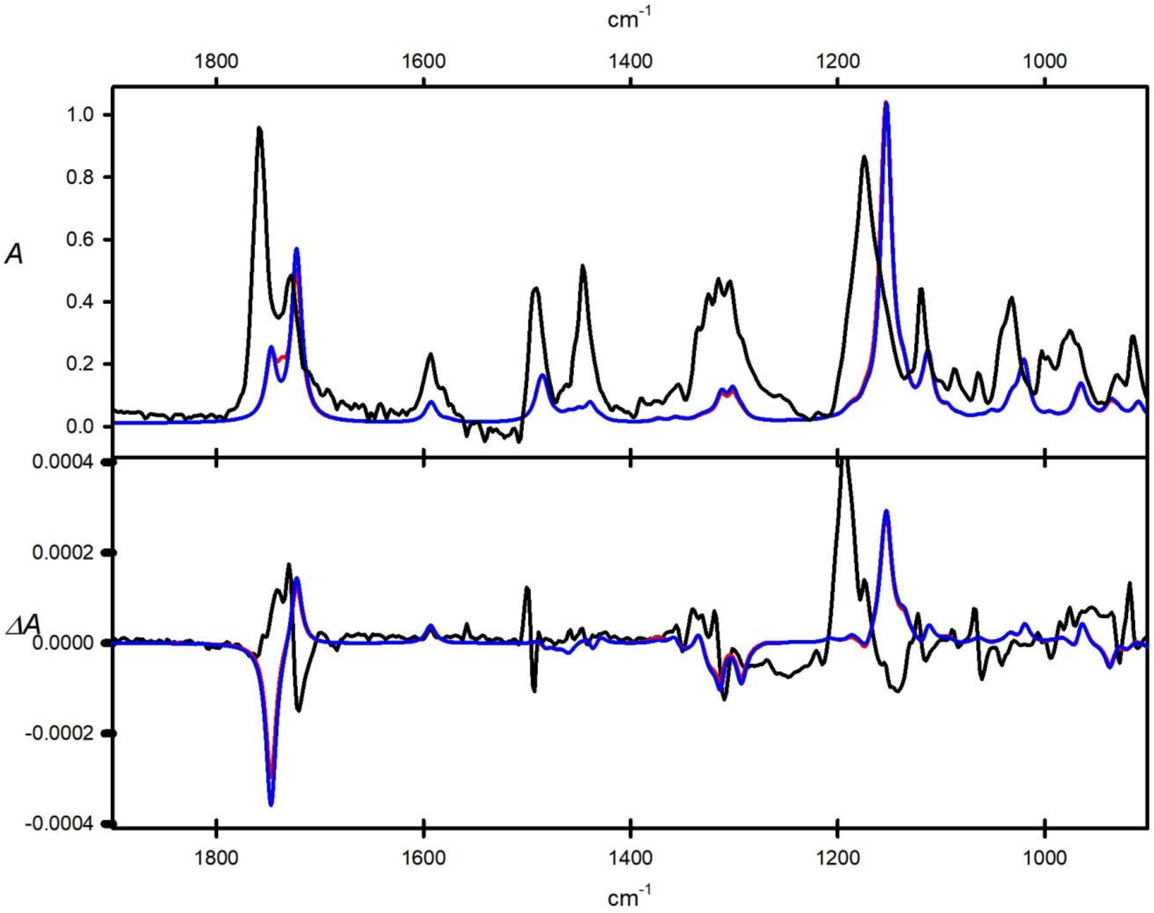

Figure S_65. IR (upper panel) and VCD (lower panel) spectra of **9**, measured in CCl_4 (solid black lines) and calculated (blue dashed lines) for geometries optimized at the IEFPCM (CCl_4)/B3LYP/6-311G(d,p) level. The calculated VCD spectra were Boltzmann-averaged based on *ΔΔG* values and were not scaled.

Copies of ^{1}H and ^{13}C NMR spectra

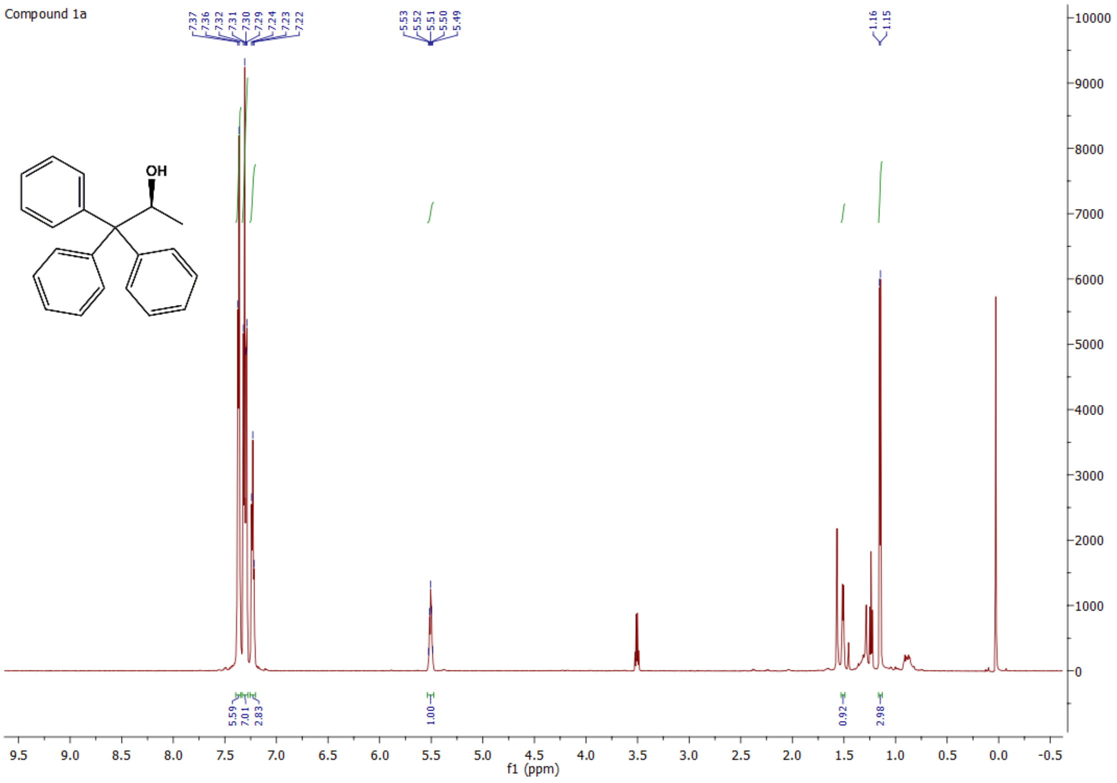

Figure S_66. Copy of ^{1}H NMR spectrum of **1a** measured in $CDCl_3$.

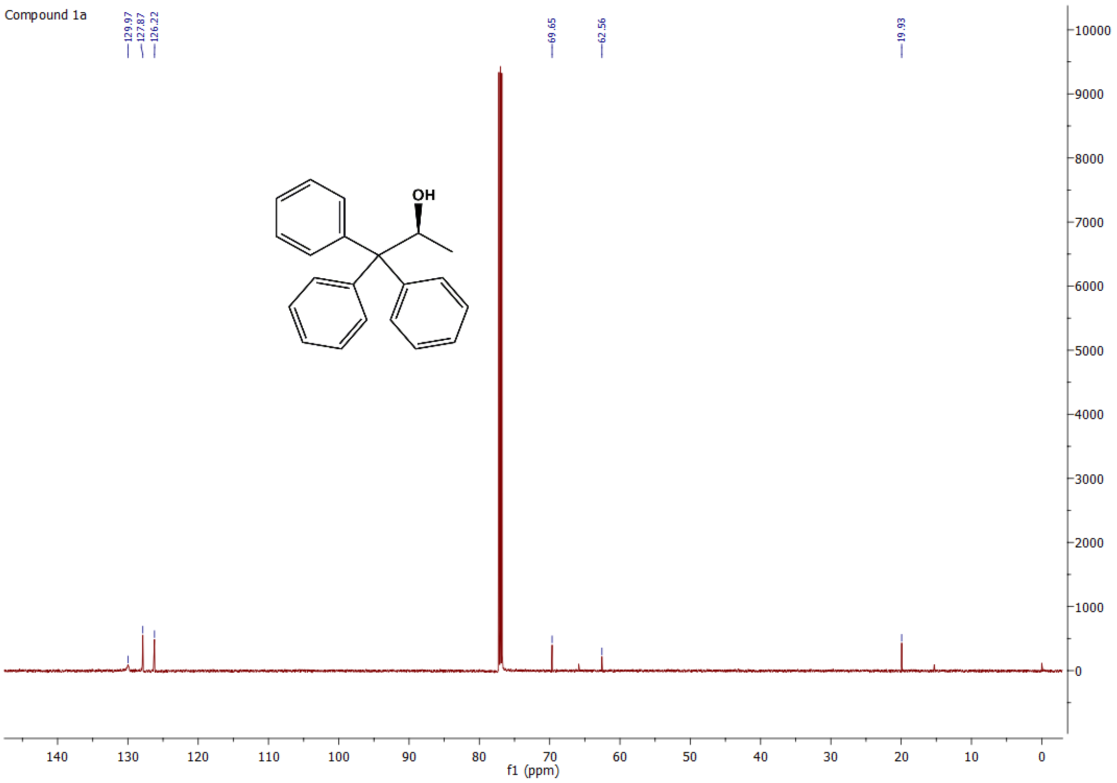

Figure S_67. Copy of 13C NMR spectrum of **1a** measured in $CDCl_3$.

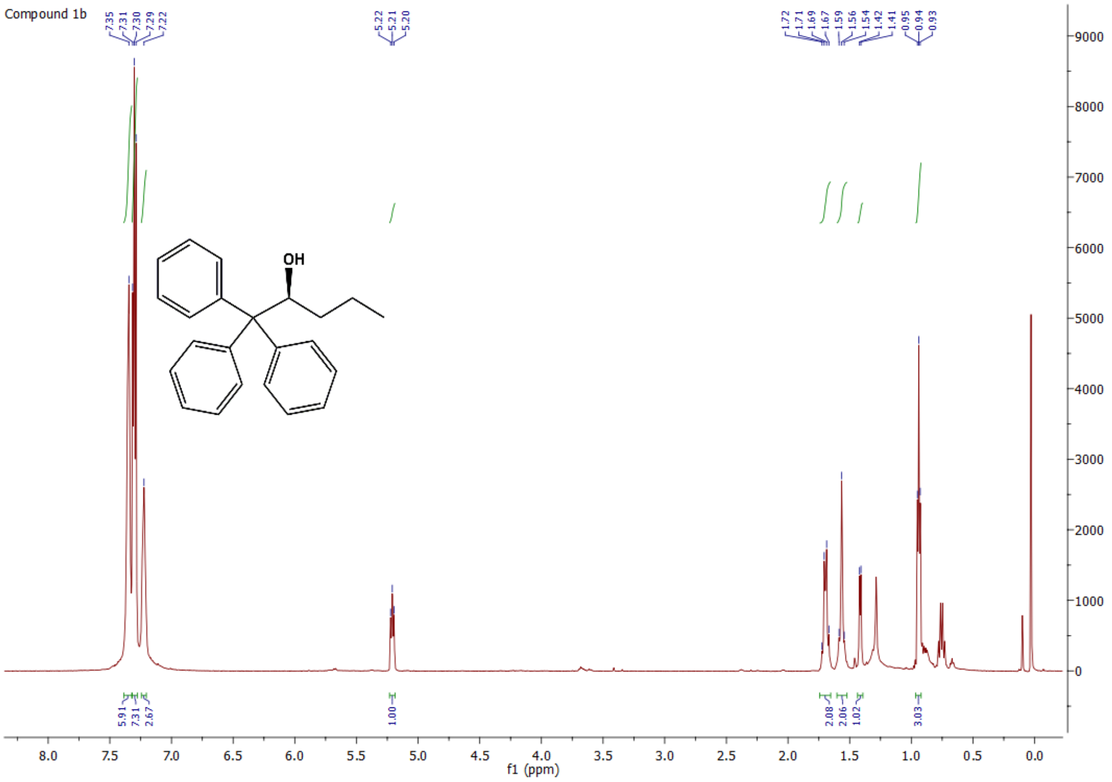

Figure S_68. Copy of ^{1}H NMR spectrum of **1b** measured in $CDCl_3$.

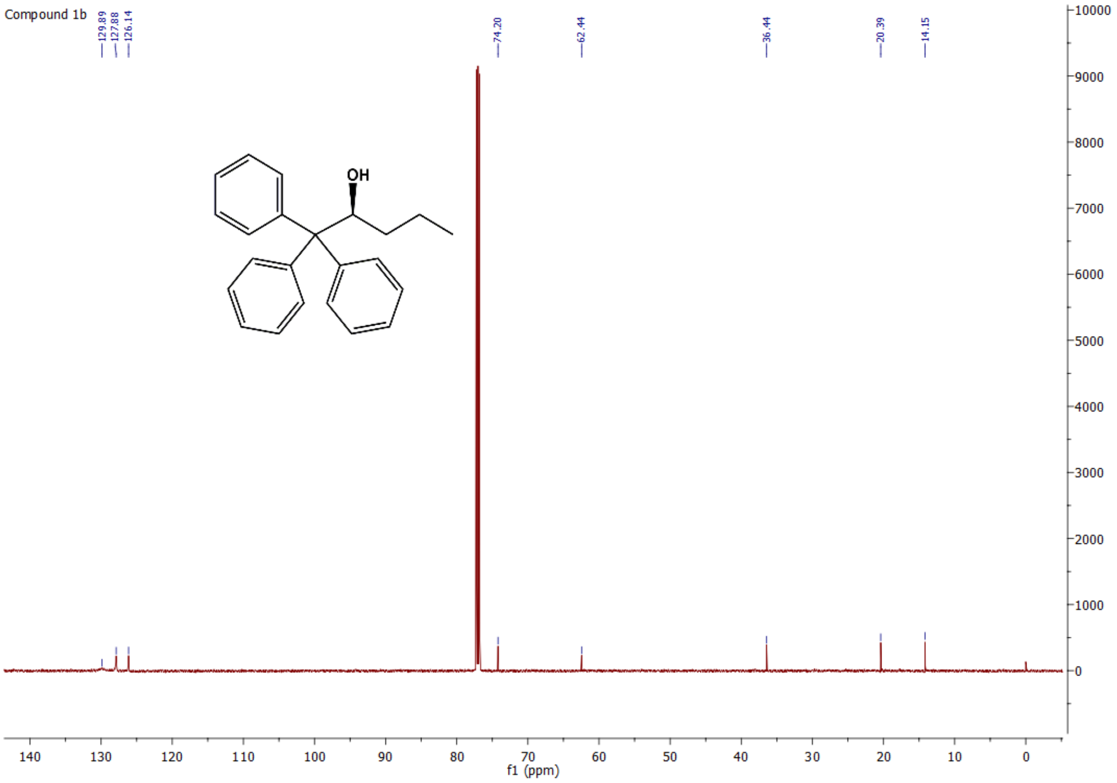

Figure S_69. Copy of ^{13}C NMR spectrum of **1b** measured in $CDCl_3$.

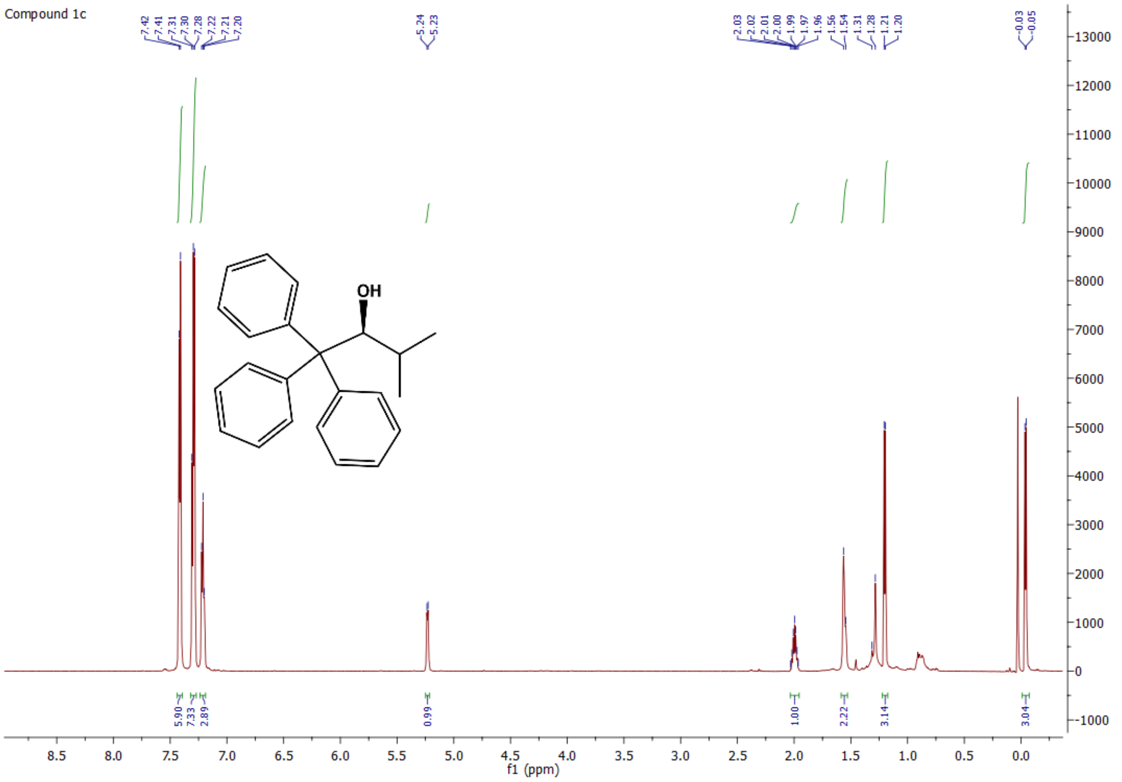

Figure S_70. Copy of ^{1}H NMR spectrum of **1c** measured in $CDCl_3$.

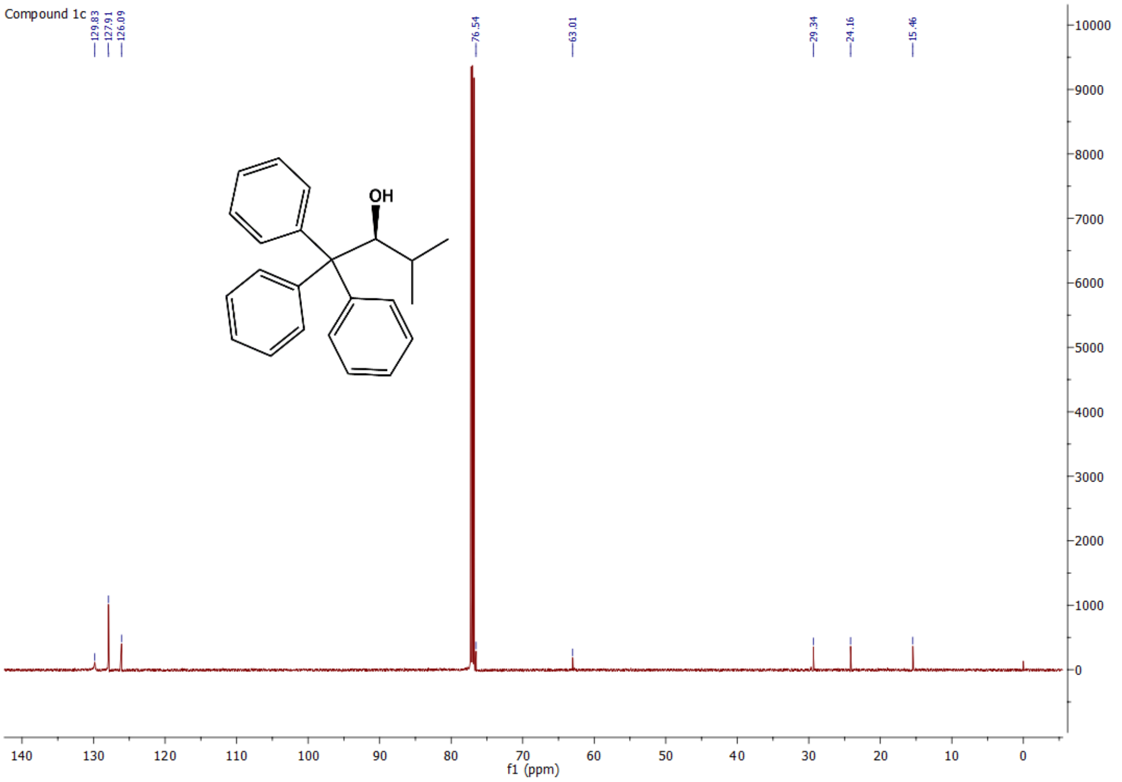

Figure S_71. Copy of ^{13}C NMR spectrum of **1c** measured in $CDCl_3$.

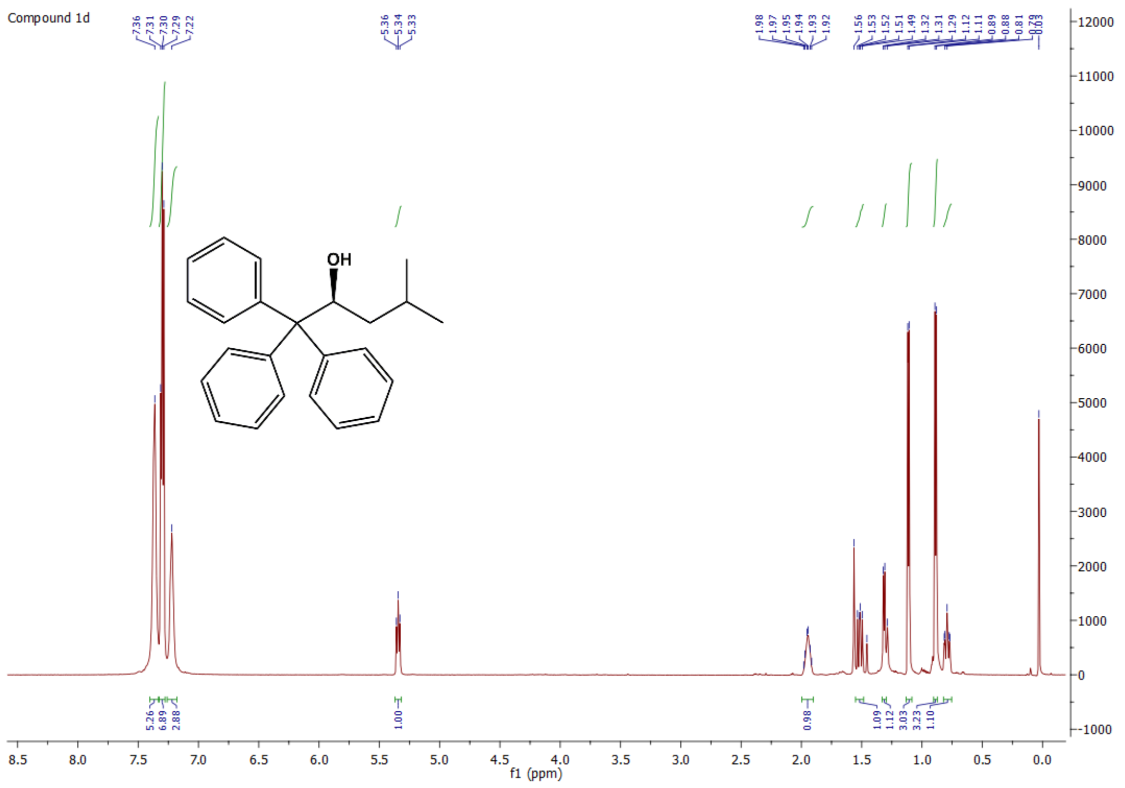

Figure S_72. Copy of ^{1}H NMR spectrum of **1d** measured in $CDCl_3$.

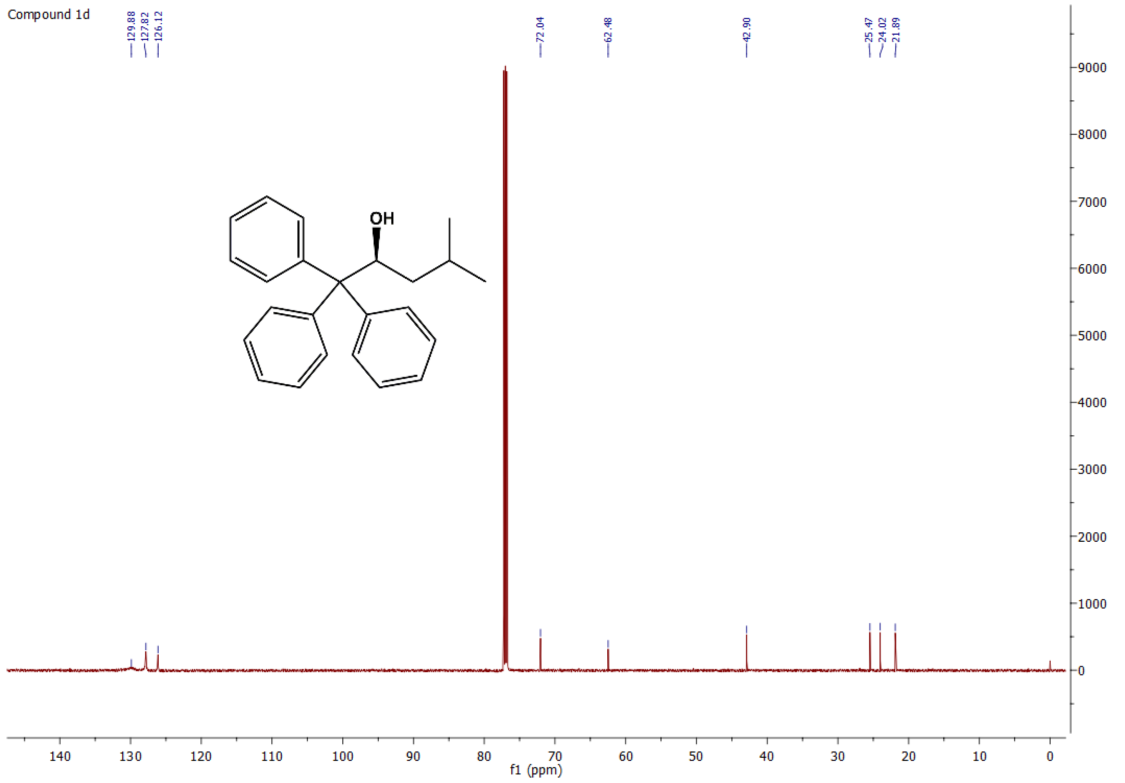

Figure S_73. Copy of ^{13}C NMR spectrum of **1d** measured in $CDCl_3$.

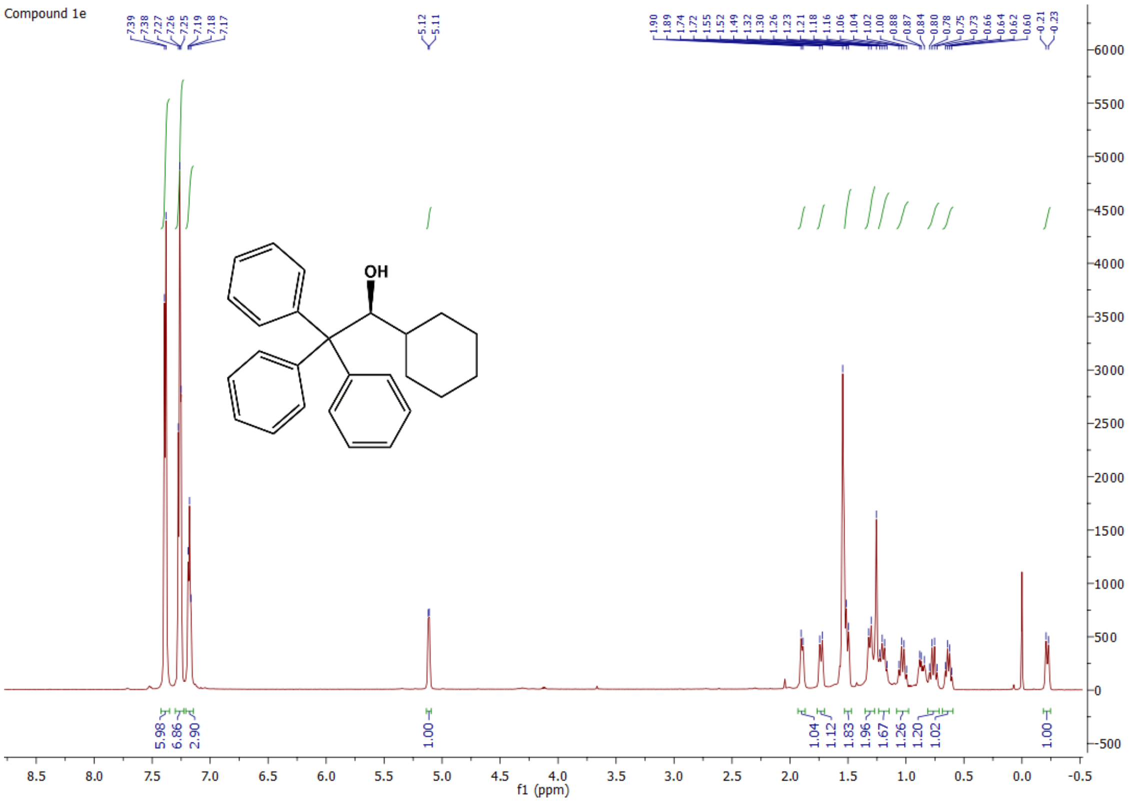

Figure S_74. Copy of ^{1}H NMR spectrum of **1e** measured in $CDCl_3$.

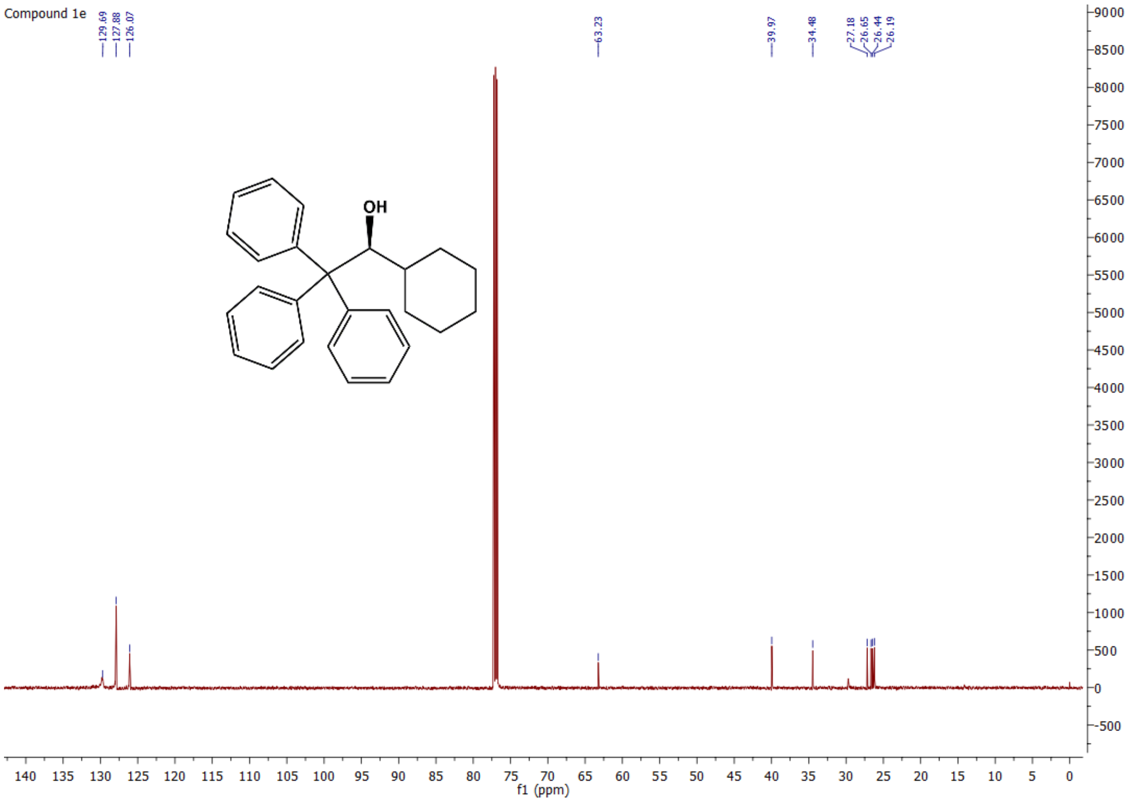

Figure S_75. Copy of ^{13}C NMR spectrum of **1e** measured in $CDCl_3$.

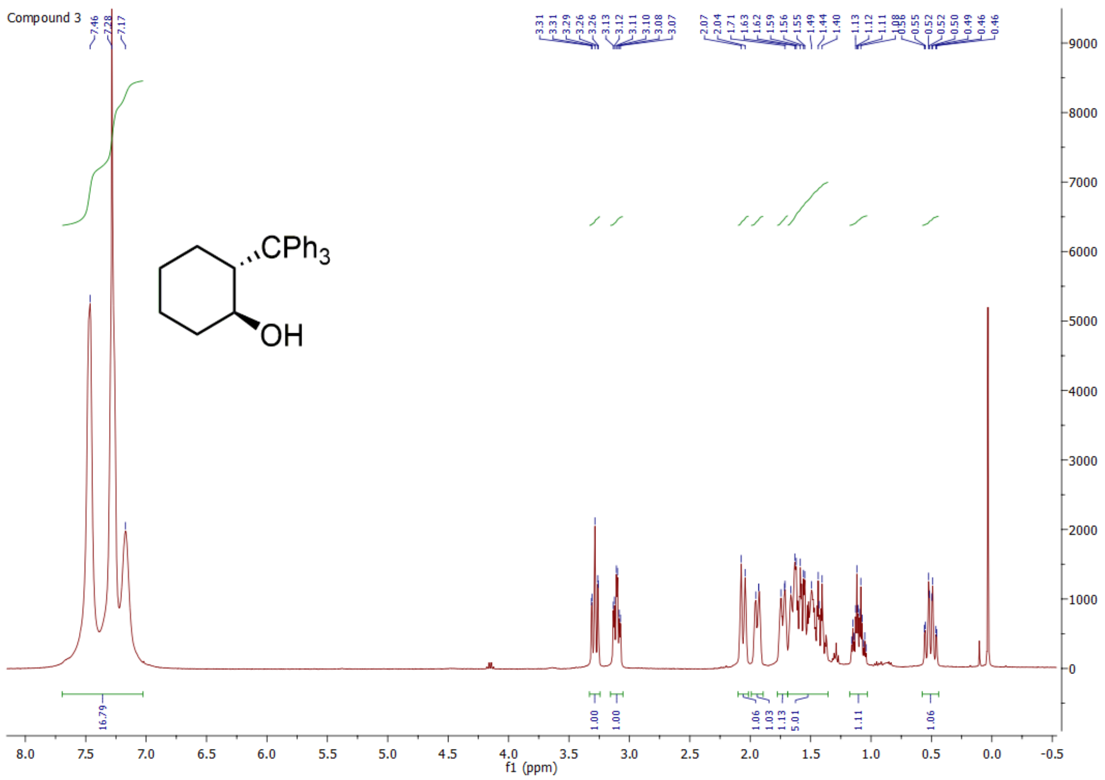

Figure S_76. Copy of ^{1}H NMR spectrum of alcohol **3** measured in $CDCl_3$.

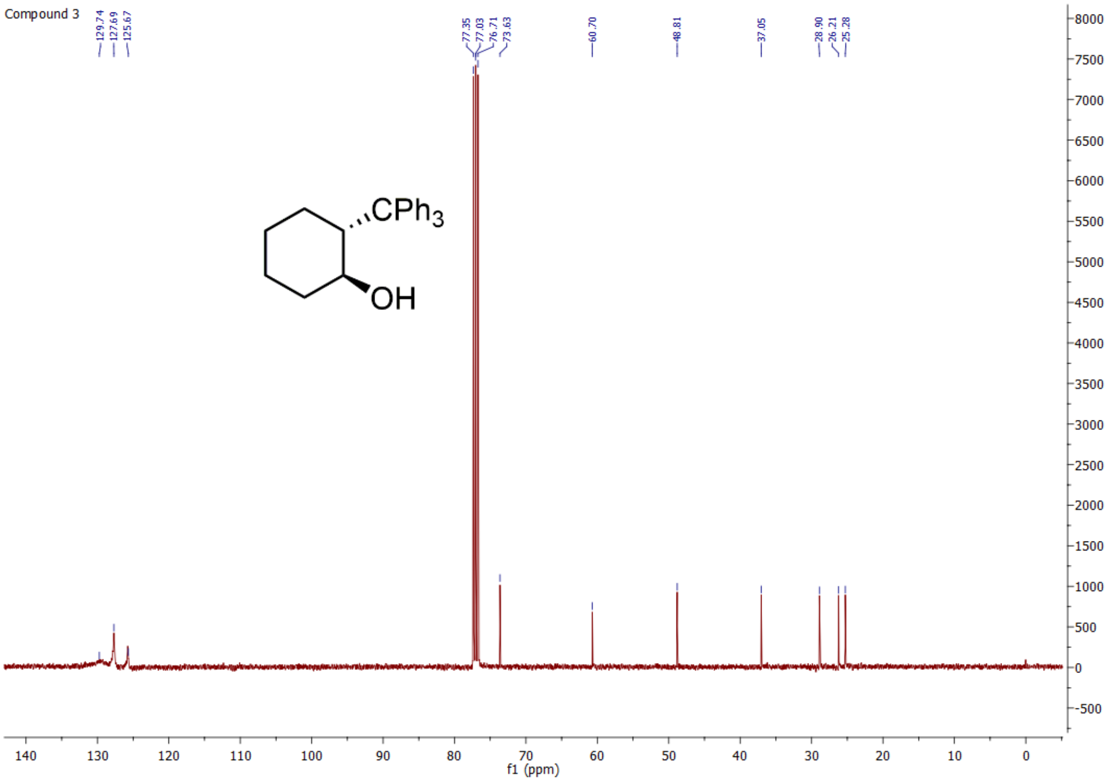

Figure S_77. Copy of ^{13}C NMR spectrum of alcohol **3** measured in $CDCl_3$.

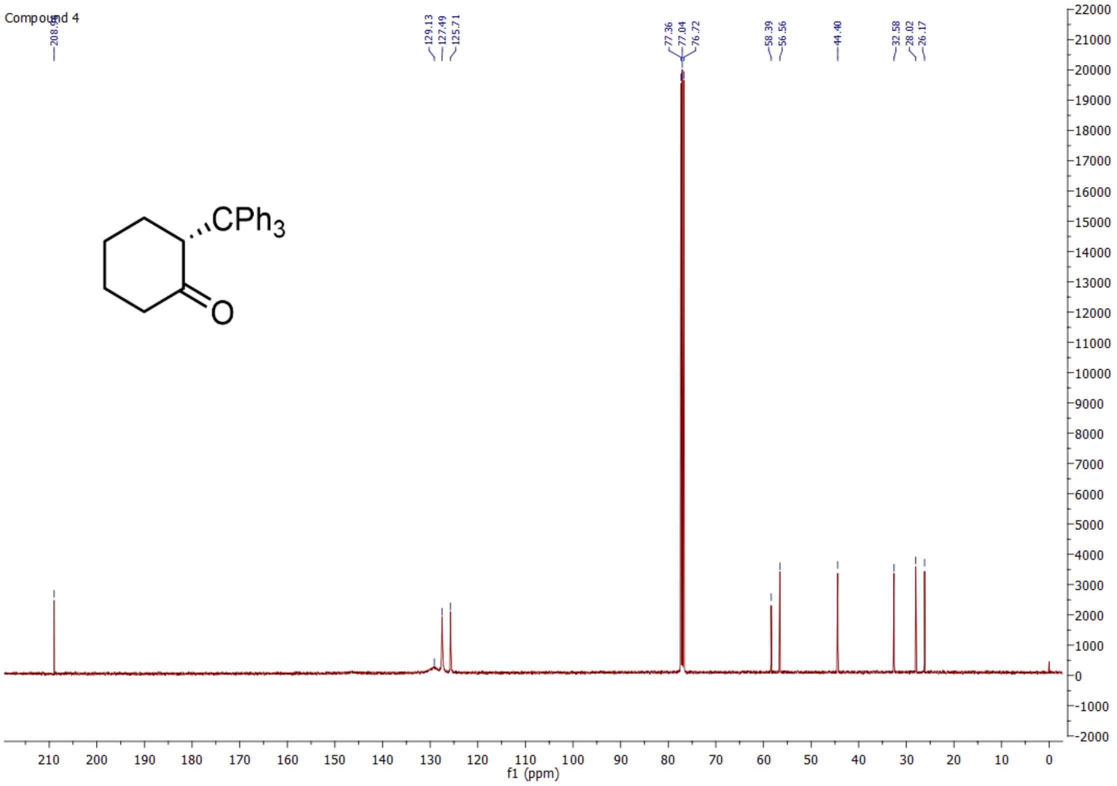

Figure S_78. Copy of ^{1}H NMR spectrum of ketone **4** measured in $CDCl_3$.

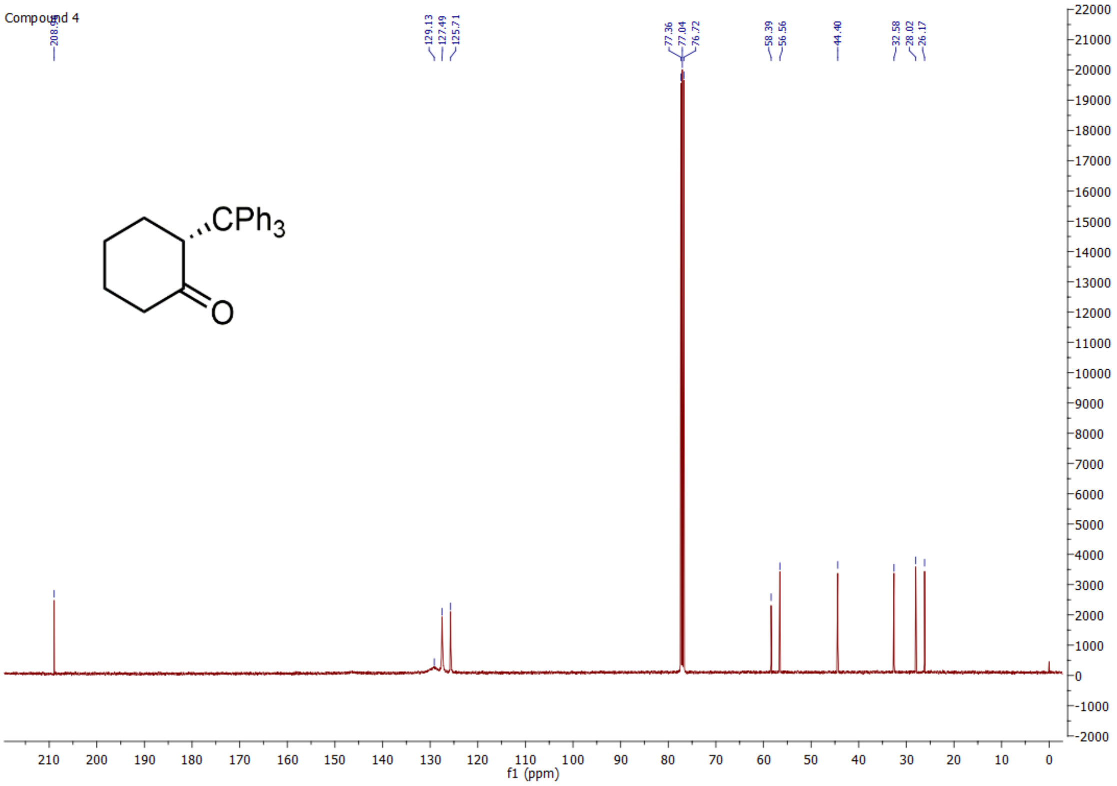

Figure S_79. Copy of ^{13}C NMR spectrum of ketone **4** measured in $CDCl_3$.

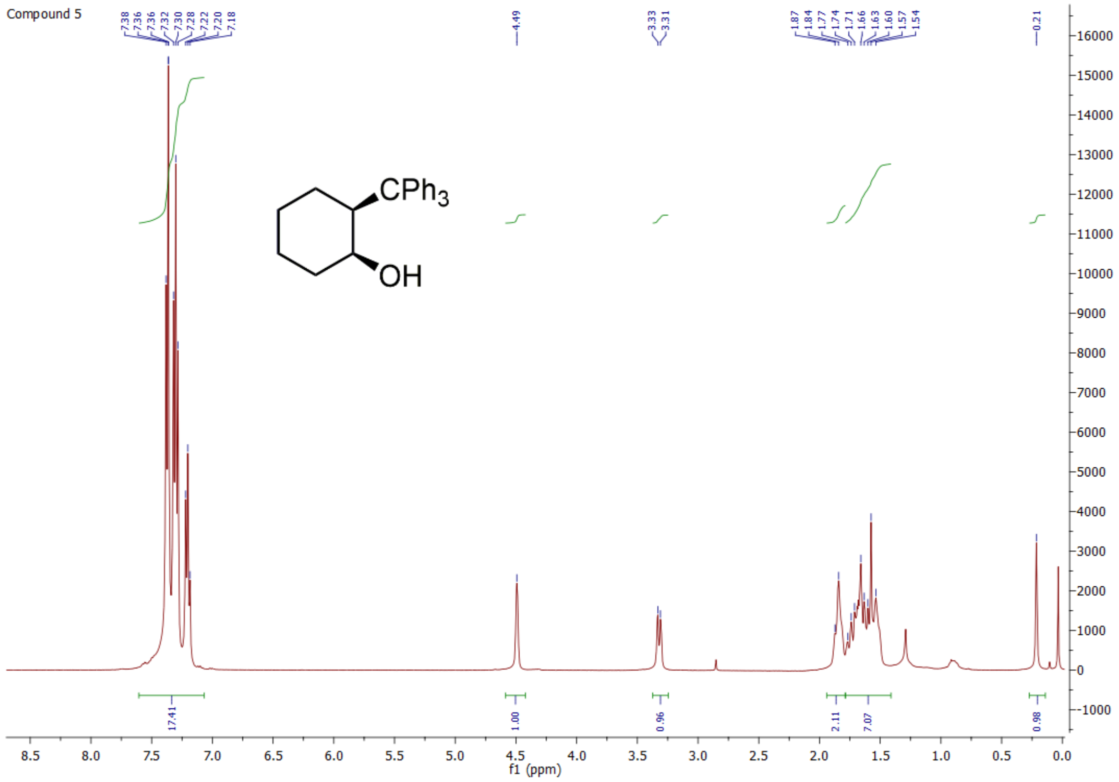

Figure S_80. Copy of ^{1}H NMR spectrum of alcohol **5** measured in $CDCl_3$.

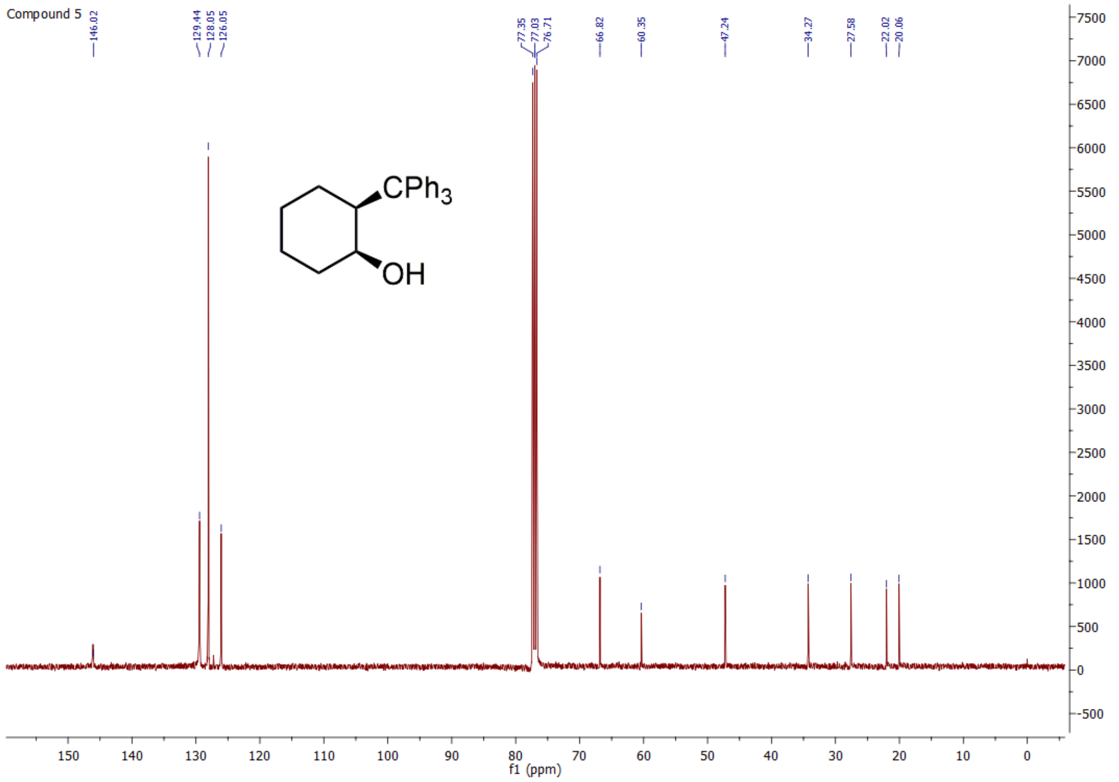

Figure S_81. Copy of ^{13}C NMR spectrum of alcohol **5** measured in $CDCl_3$.

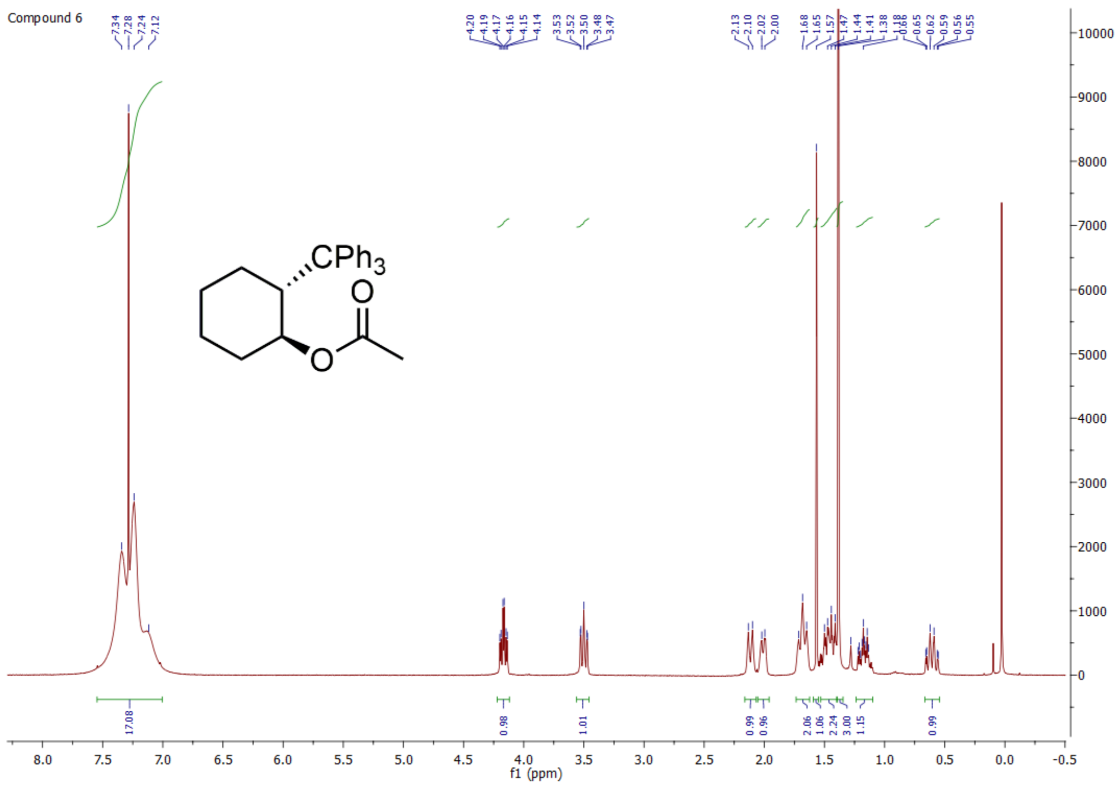

Figure S_82. Copy of ^{1}H NMR spectrum of ester **6** measured in $CDCl_3$.

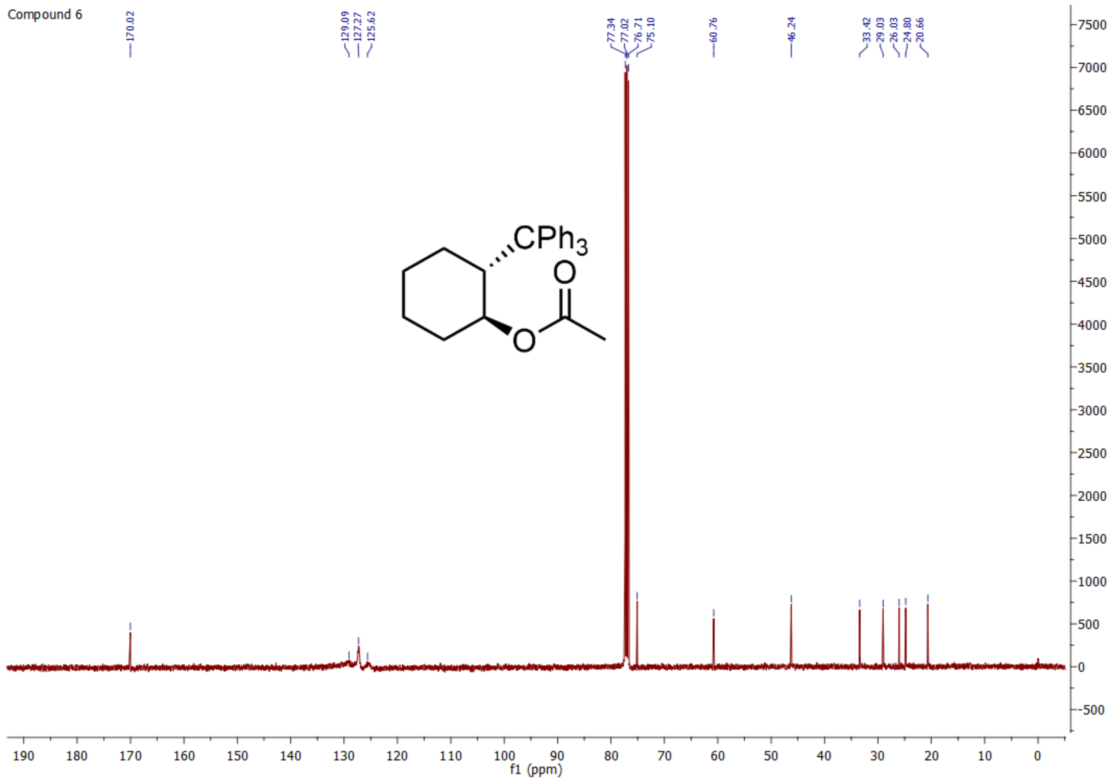

Figure S_83. Copy of ^{13}C NMR spectrum of ester **6** measured in $CDCl_3$.

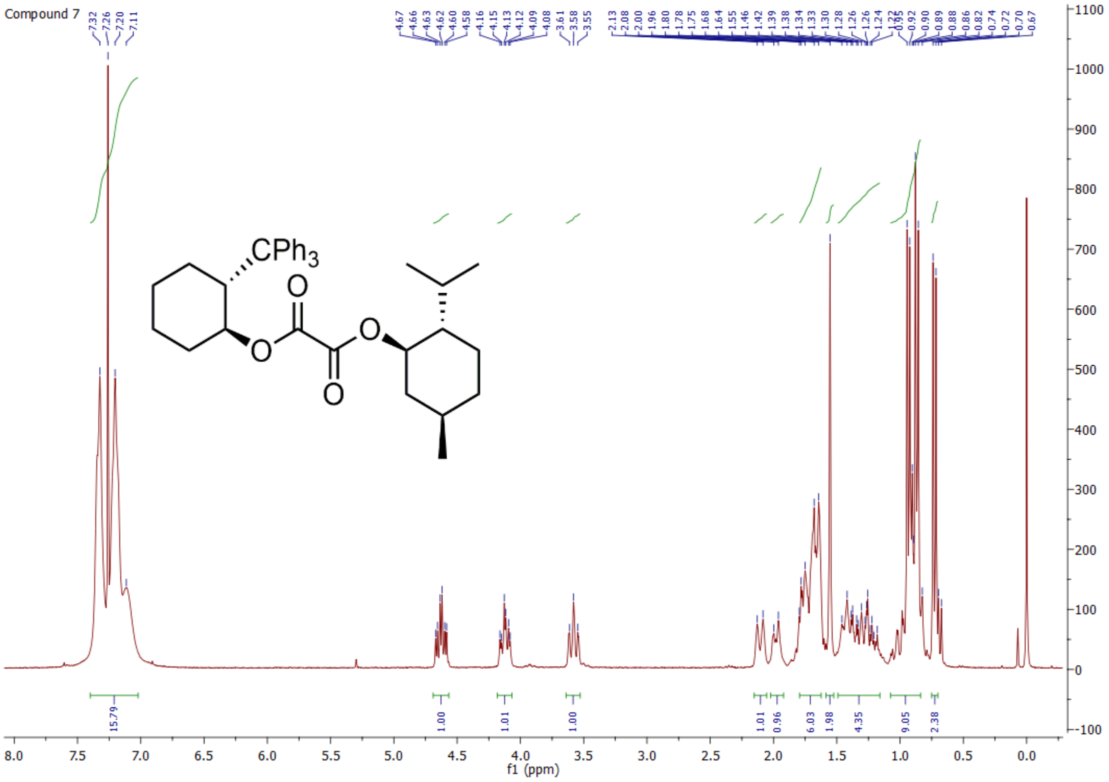

Figure S_84. Copy of ^{1}H NMR spectrum of diester **7** measured in $CDCl_3$.

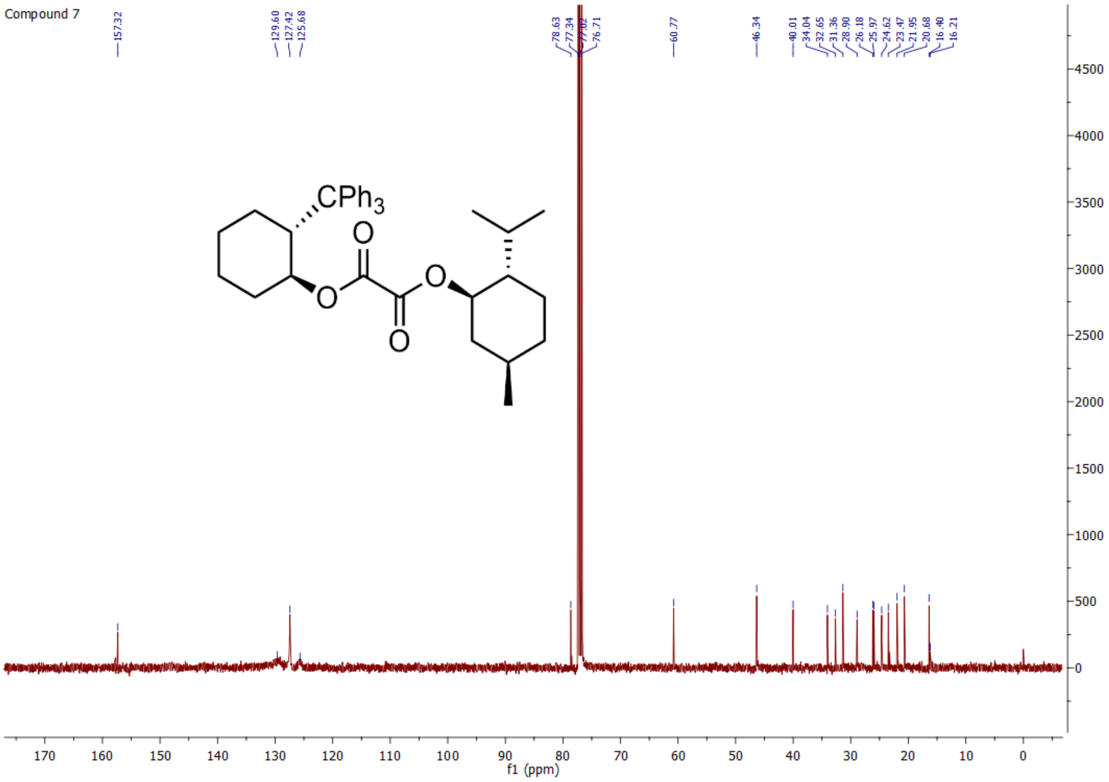

Figure S_85. Copy of ^{13}C NMR spectrum of diester **7** measured in $CDCl_3$.

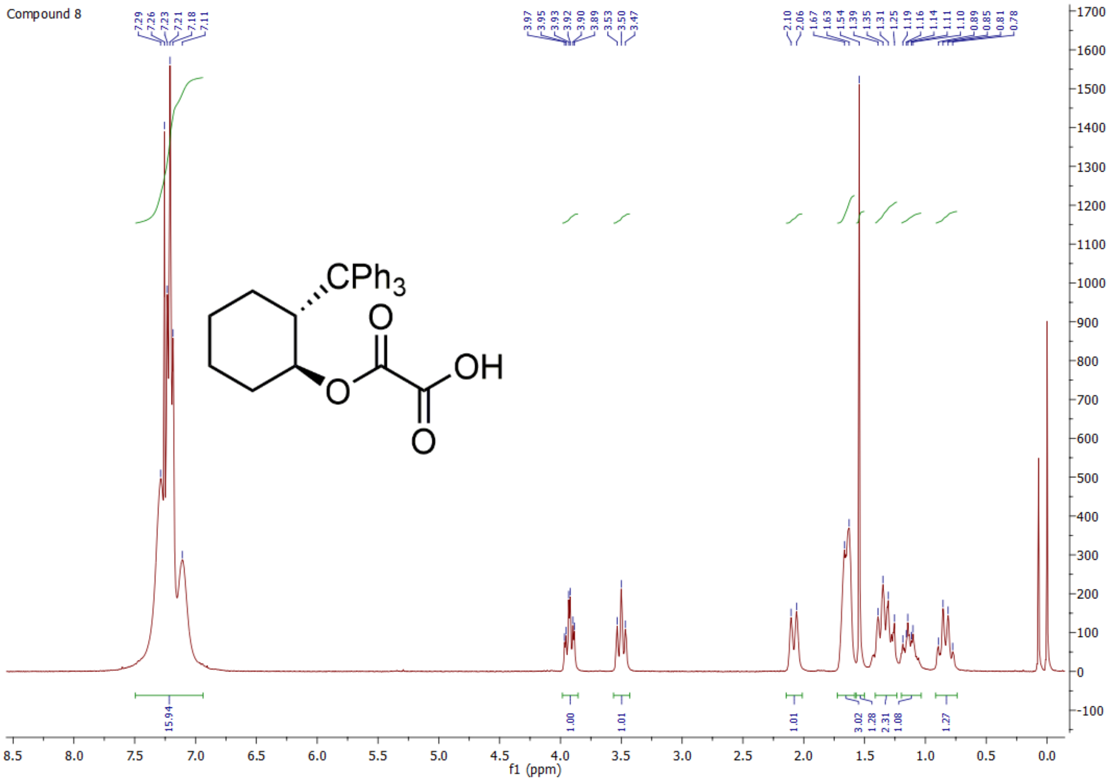

Figure S_86. Copy of 1H NMR spectrum of ester **8** measured in $CDCl_3$.

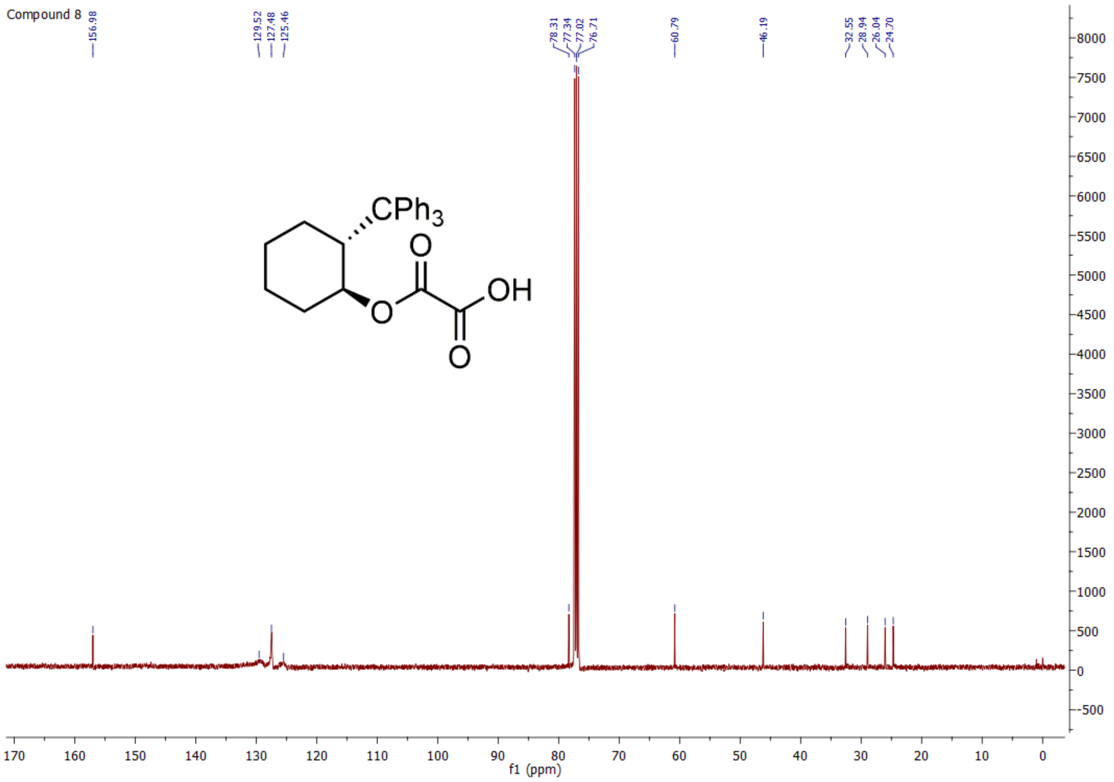

Figure S_87. Copy of ^{13}C NMR spectrum of ester **8** measured in $CDCl_3$.

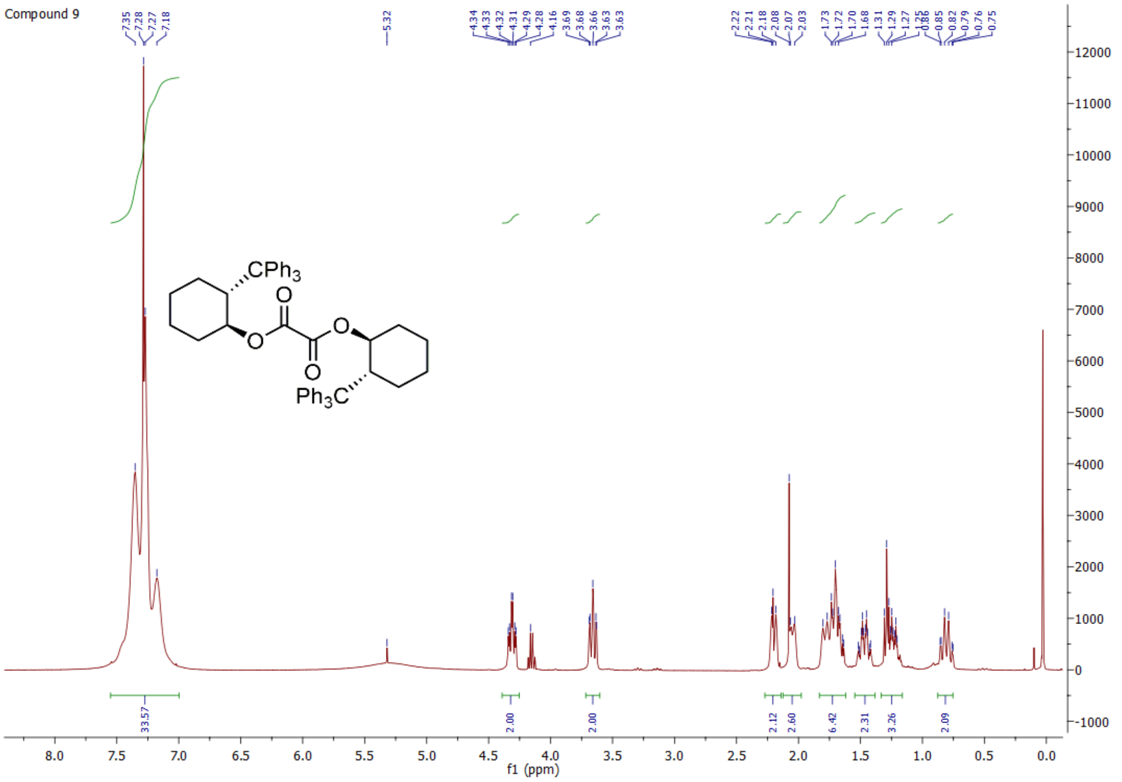

Figure S_88. Copy of ^{1}H NMR spectrum of diester **9** measured in $CDCl_3$.

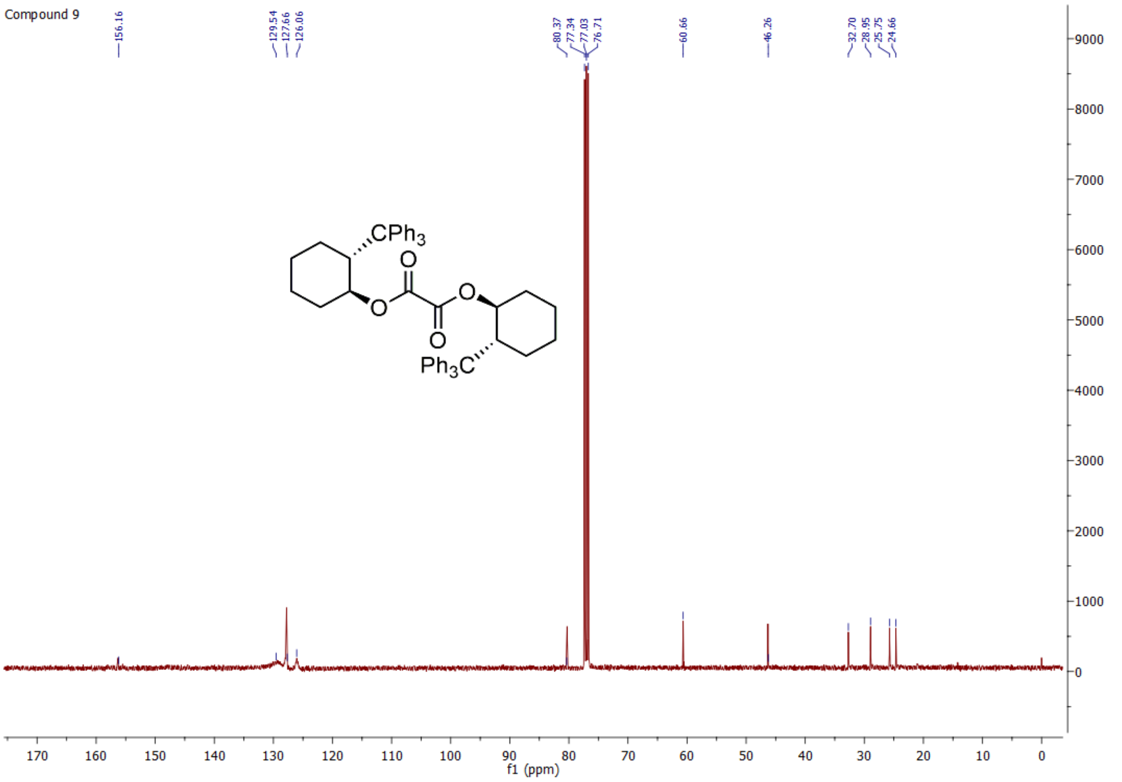

Figure S_89. Copy of ^{13}C NMR spectrum of diester **9** measured in $CDCl_3$.

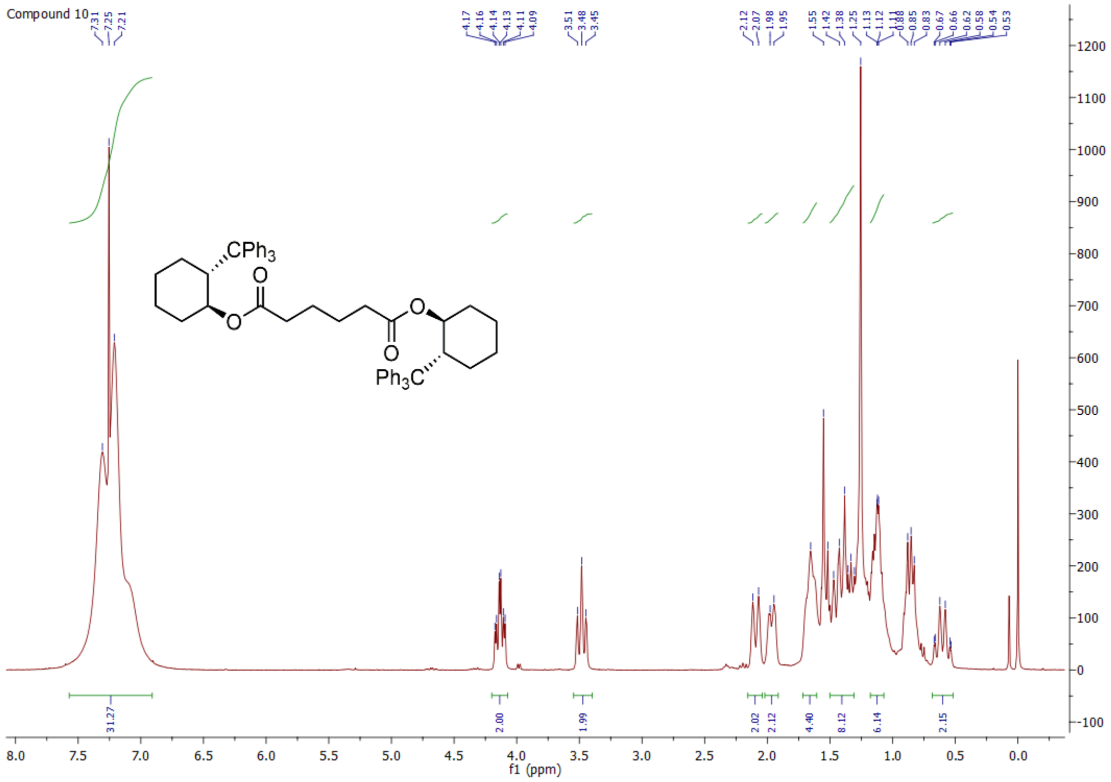

Figure S_90. Copy of ^{1}H NMR spectrum of diester **10** measured in $CDCl_3$.

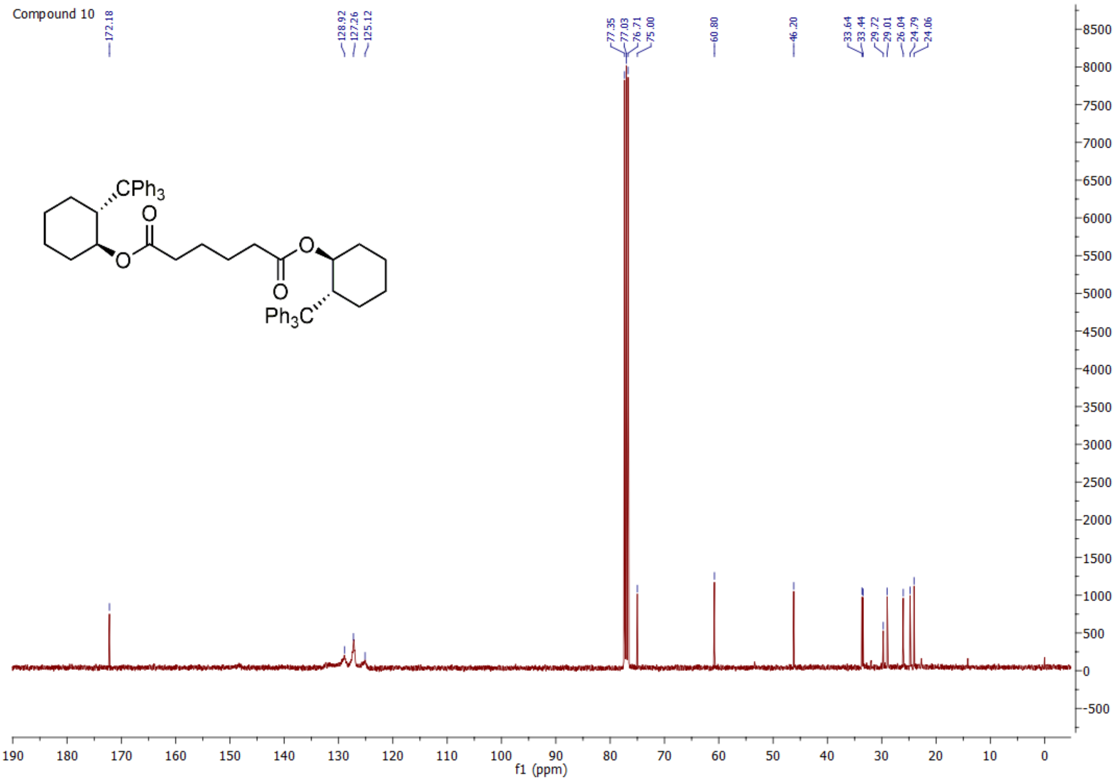

Figure S_91. Copy of ^{13}C NMR spectrum of diester **10** measured in $CDCl_3$.

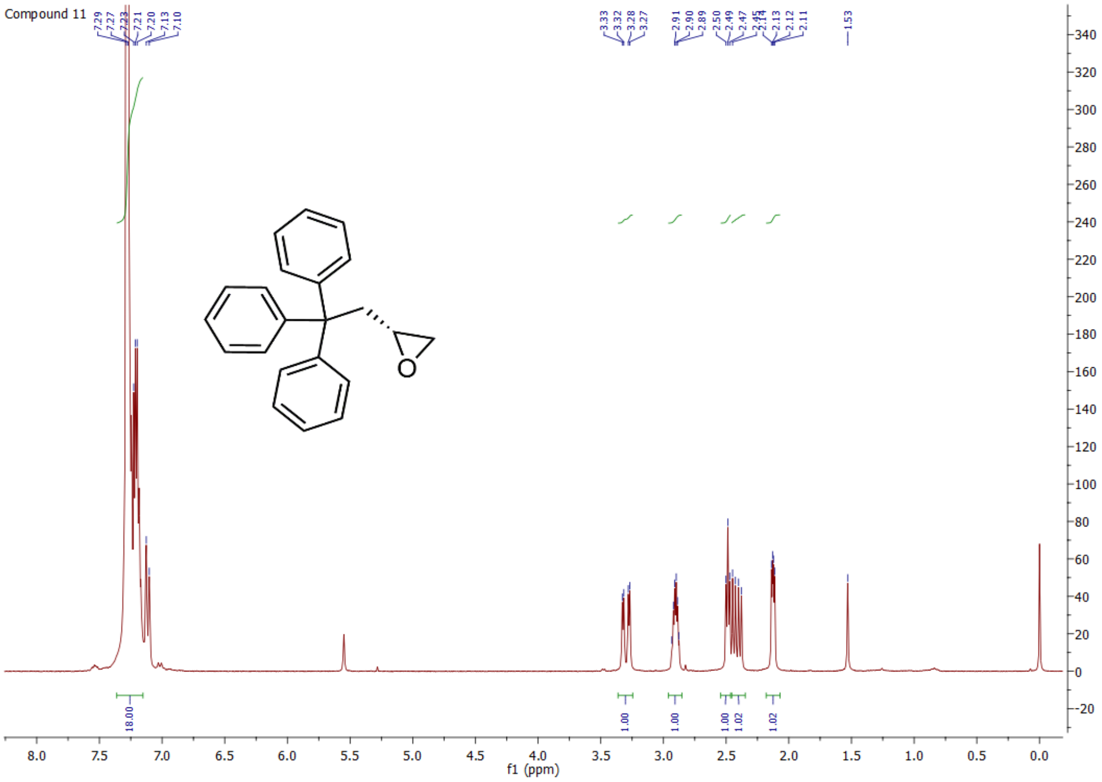

Figure S_92. Copy of ^{1}H NMR spectrum of oxirane **11** measured in $CDCl_3$.

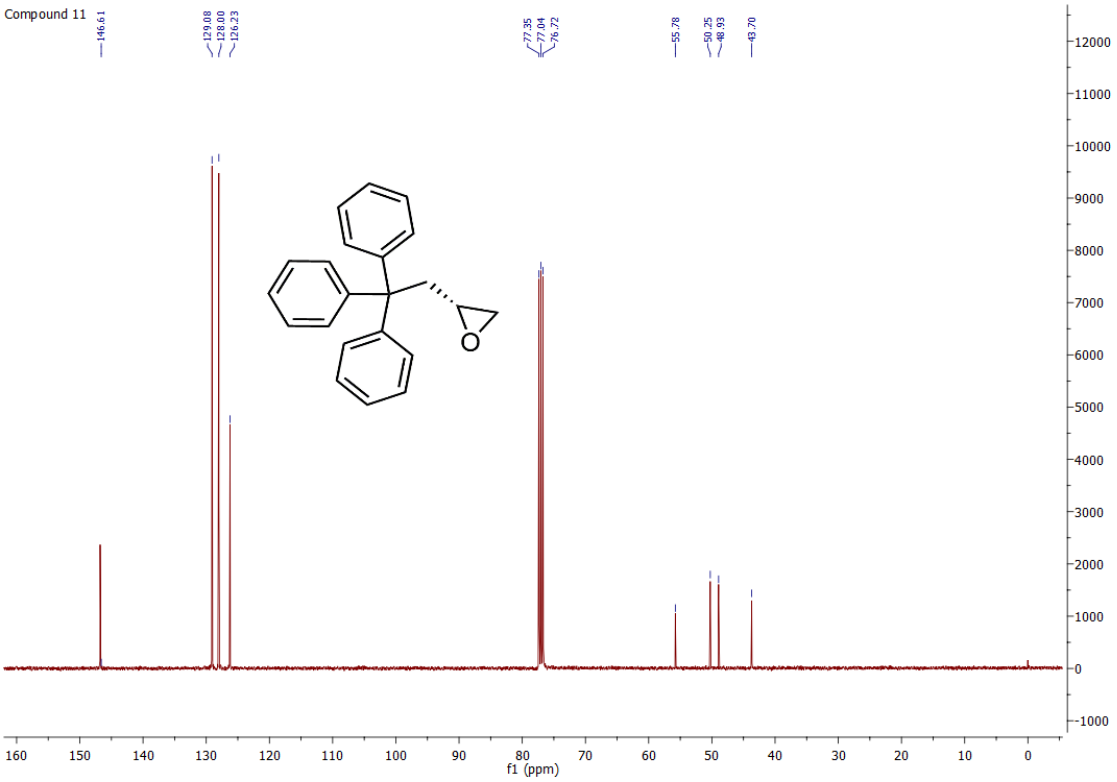

Figure S_93. Copy of ^{13}C NMR spectrum of oxirane **11** measured in $CDCl_3$.

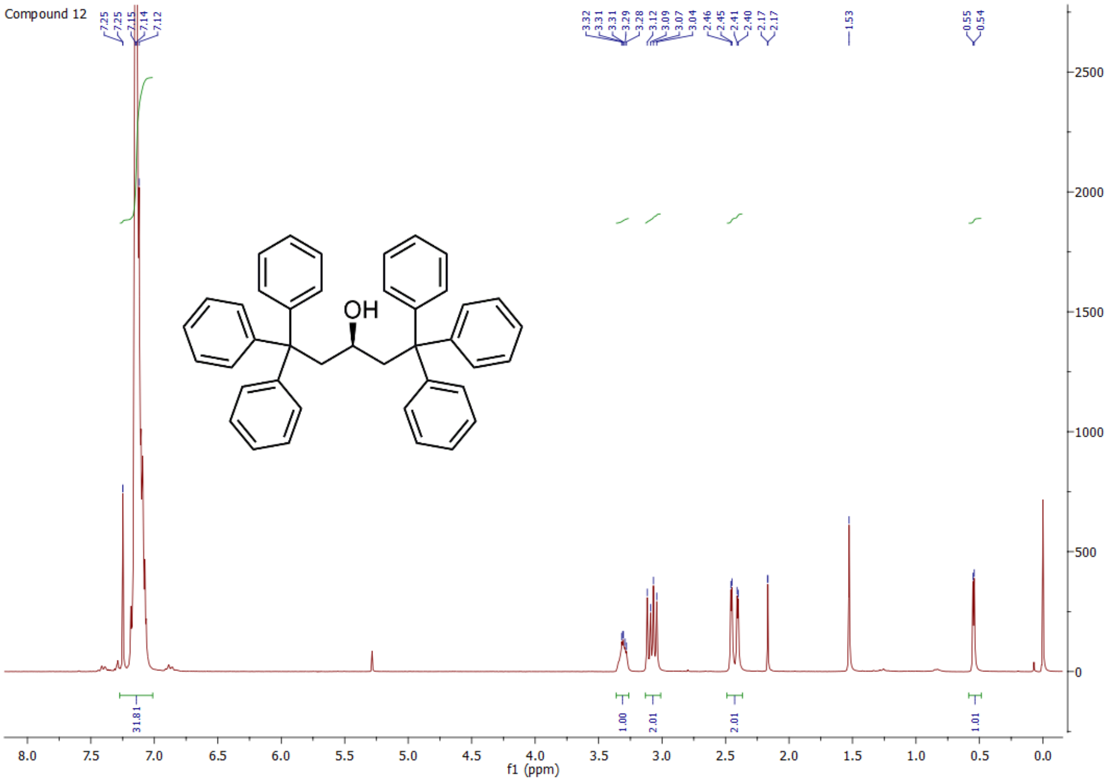

Figure S_94. Copy of ^{1}H NMR spectrum of alcohol **12** measured in $CDCl_3$.

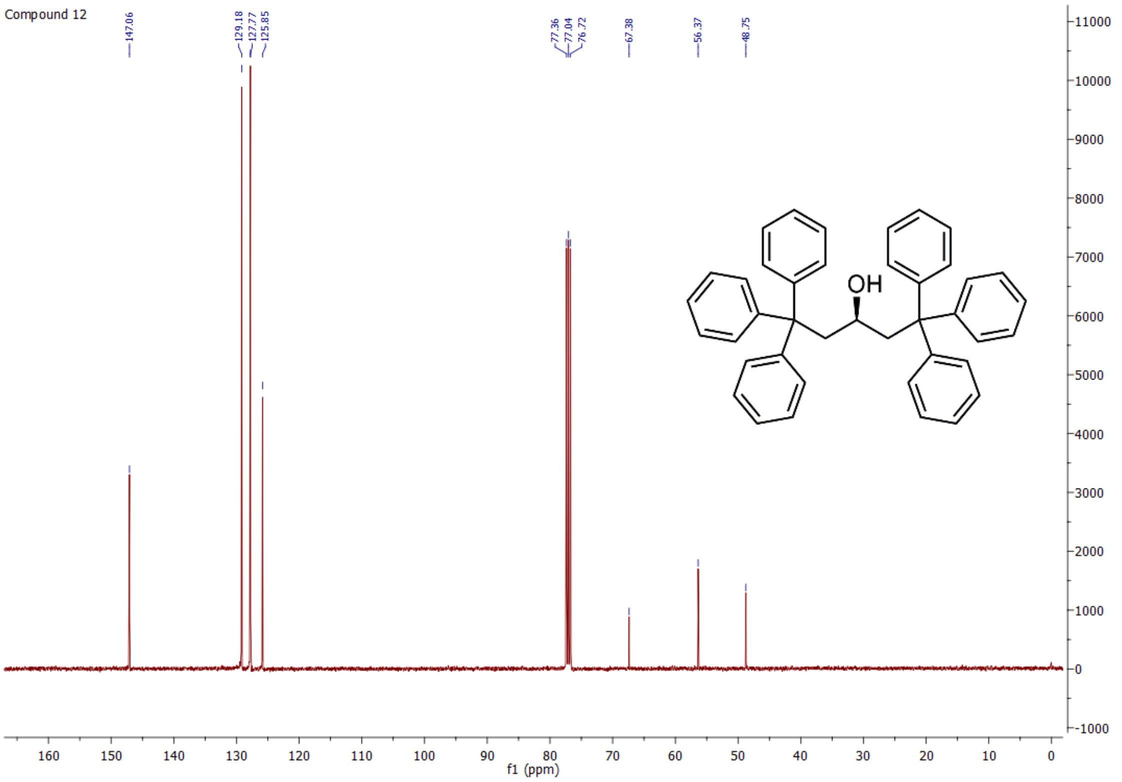

Figure S_95. Copy of ^{13}C NMR spectrum of alcohol **12** measured in $CDCl_3$.

Cartesian coordinates for all calculated structures

**1a** (conf. 1)	C	0.23740000	-0.08830000	-2.03110000
	H	-0.70660000	0.21760000	-2.48340000
	C	0.61910000	-1.45060000	-2.61470000
	H	1.62580000	-1.74670000	-2.31270000
	H	-0.07710000	-2.23810000	-2.32230000
	H	0.61260000	-1.36870000	-3.70400000
	O	1.16440000	0.90140000	-2.47890000
	H	2.01500000	0.74080000	-2.05220000
	C	0.03750000	-0.03210000	-0.45770000
	C	-0.60890000	1.31870000	-0.03270000
	C	-1.84400000	3.70250000	0.85270000
	C	-0.96730000	1.50010000	1.31220000
	C	-0.87940000	2.36480000	-0.92130000
	C	-1.49460000	3.54030000	-0.48310000
	C	-1.57140000	2.67250000	1.75280000
	H	-0.77800000	0.70980000	2.02850000
	H	-0.58350000	2.29330000	-1.95750000
	H	-1.69070000	4.33290000	-1.19710000
	H	-1.83620000	2.77810000	2.79920000
	H	-2.32020000	4.61610000	1.19040000
	C	-0.97530000	-1.12270000	-0.01850000
	C	-2.94860000	-2.97340000	0.81310000
	C	-2.15930000	-1.30740000	-0.74950000
	C	-0.82300000	-1.87120000	1.15470000
	C	-1.79410000	-2.78530000	1.56550000
	C	-3.12750000	-2.22250000	-0.34750000
	H	-2.34670000	-0.71960000	-1.63940000
	H	0.05860000	-1.74130000	1.76810000
	H	-1.64250000	-3.34730000	2.48080000
	H	-4.02830000	-2.34130000	-0.93960000
	H	-3.70260000	-3.68520000	1.12920000
	C	1.43780000	-0.18990000	0.17820000
	C	4.08170000	-0.44000000	1.16010000
	C	2.07280000	-1.44090000	0.26010000
	C	2.17310000	0.93130000	0.58890000
	C	3.47640000	0.80960000	1.07340000
	C	3.37140000	-1.56620000	0.74820000
	H	1.54670000	-2.33450000	-0.04820000
	H	1.72340000	1.91410000	0.53530000
	H	4.01350000	1.69880000	1.38490000
	H	3.82730000	-2.54860000	0.80580000
	H	5.09190000	-0.53710000	1.54150000
**1a** (conf. 72)	C	-0.20200000	-0.10530000	-2.03490000
	H	0.77070000	0.14360000	-2.47240000
	C	-1.26490000	0.80440000	-2.64420000
	H	-1.17690000	1.83490000	-2.29980000
	H	-1.17070000	0.78620000	-3.73240000
	H	-2.26020000	0.44370000	-2.38320000

	O	-0.55650000	-1.42660000	-2.44900000
	H	0.15160000	-2.02880000	-2.19170000
	C	-0.04320000	0.00220000	-0.45690000
	C	0.80280000	-1.21170000	0.02070000
	C	2.45750000	-3.34330000	0.86910000
	C	2.01980000	-1.49440000	-0.62280000
	C	0.45100000	-2.01130000	1.11320000
	C	1.26800000	-3.06220000	1.53350000
	C	2.83150000	-2.54900000	-0.21350000
	H	2.35780000	-0.86870000	-1.44130000
	H	-0.46830000	-1.81820000	1.64980000
	H	0.96670000	-3.66130000	2.38590000
	H	3.76270000	-2.74100000	-0.73510000
	H	3.08870000	-4.16290000	1.19300000
	C	0.78070000	1.25400000	-0.05220000
	C	2.32590000	3.46220000	0.81380000
	C	1.15580000	1.40460000	1.29320000
	C	1.20860000	2.23790000	-0.94990000
	C	1.97250000	3.32800000	-0.52310000
	C	1.91030000	2.48940000	1.72360000
	H	0.85450000	0.65610000	2.01620000
	H	0.95880000	2.18030000	-1.99960000
	H	2.28770000	4.07100000	-1.24760000
	H	2.18030000	2.57200000	2.77070000
	H	2.91880000	4.30740000	1.14460000
	C	-1.45710000	0.02990000	0.16540000
	C	-4.10840000	0.08610000	1.14960000
	C	-2.30630000	-1.08260000	0.03810000
	C	-1.97840000	1.17200000	0.78420000
	C	-3.28570000	1.20070000	1.27300000
	C	-3.61040000	-1.05600000	0.52410000
	H	-1.94950000	-1.97080000	-0.46410000
	H	-1.36710000	2.05840000	0.88810000
	H	-3.65640000	2.10230000	1.74840000
	H	-4.23990000	-1.93180000	0.40890000
	H	-5.12350000	0.10640000	1.53010000
**1b** (conf. 1)	C	-0.88770000	-0.00180000	1.34860000
	H	-0.58190000	-0.82750000	1.99370000
	C	-2.36870000	-0.20790000	0.99350000
	H	-2.76160000	0.71480000	0.55360000
	H	-2.47140000	-0.98930000	0.23520000
	O	-0.74330000	1.15010000	2.18200000
	H	-0.88670000	1.93720000	1.64200000
	C	0.12570000	0.03440000	0.12960000
	C	-0.04650000	-1.24210000	-0.73630000
	C	-0.19690000	-3.63380000	-2.24280000
	C	-0.16160000	-2.49410000	-0.11210000
	C	0.01890000	-1.22700000	-2.13450000
	C	-0.05670000	-2.40500000	-2.87890000
	C	-0.24370000	-3.67090000	-0.85020000
	H	-0.17000000	-2.56520000	0.96840000

	H	0.13700000	-0.28930000	-2.66110000
	H	-0.00250000	-2.35470000	-3.96110000
	H	-0.33560000	-4.61980000	-0.33330000
	H	-0.25890000	-4.54950000	-2.81980000
	C	1.59830000	-0.01590000	0.63180000
	C	4.30980000	-0.17950000	1.41270000
	C	2.62970000	-0.08080000	-0.31800000
	C	1.96020000	-0.02980000	1.98310000
	C	3.30100000	-0.11560000	2.36720000
	C	3.96510000	-0.15720000	0.06130000
	H	2.38640000	-0.07720000	-1.37370000
	H	1.20800000	0.06030000	2.75300000
	H	3.54960000	-0.12350000	3.42290000
	H	4.73640000	-0.20660000	-0.69960000
	H	5.34940000	-0.24440000	1.71380000
	C	-0.14350000	1.35020000	-0.63750000
	C	-0.74420000	3.82360000	-1.87700000
	C	-1.24470000	1.48820000	-1.49950000
	C	0.63620000	2.49230000	-0.40380000
	C	0.34110000	3.71260000	-1.01410000
	C	-1.53780000	2.70260000	-2.11500000
	H	-1.87990000	0.63650000	-1.70290000
	H	1.48900000	2.43050000	0.25950000
	H	0.96800000	4.57410000	-0.81210000
	H	-2.39070000	2.77090000	-2.78140000
	H	-0.97150000	4.76890000	-2.35680000
	C	-3.21170000	-0.57770000	2.22130000
	H	-2.85420000	-1.53190000	2.62790000
	H	-3.05160000	0.17010000	3.00340000
	C	-4.70580000	-0.68820000	1.90490000
	H	-4.89700000	-1.44530000	1.13760000
	H	-5.28050000	-0.96410000	2.79350000
	H	-5.10320000	0.26300000	1.53650000

**1b** (conf. 27)	C	0.77770000	0.07890000	1.42010000
	H	0.42140000	0.94450000	1.99020000
	C	2.27570000	0.25100000	1.15090000
	H	2.67340000	-0.71880000	0.83950000
	H	2.43250000	0.92950000	0.30820000
	O	0.66680000	-1.08060000	2.25100000
	H	-0.24170000	-1.14600000	2.56750000
	C	-0.13330000	-0.02910000	0.12300000
	C	-0.39130000	1.36840000	-0.50150000
	C	-0.98410000	3.86300000	-1.70100000
	C	0.24490000	2.54330000	-0.08640000
	C	-1.33940000	1.48030000	-1.53190000
	C	-1.62990000	2.70220000	-2.12730000
	C	-0.04850000	3.77560000	-0.67730000
	H	0.98230000	2.52830000	0.70360000
	H	-1.86170000	0.59410000	-1.87240000
	H	-2.36700000	2.74880000	-2.92150000
	H	0.46280000	4.66630000	-0.32890000

	H	-1.21130000	4.81900000	-2.15900000
	C	-1.53140000	-0.54620000	0.56500000
	C	-4.11340000	-1.35470000	1.39080000
	C	-2.19760000	0.09930000	1.62060000
	C	-2.20300000	-1.59130000	-0.07710000
	C	-3.47810000	-1.98970000	0.32870000
	C	-3.46460000	-0.30240000	2.03520000
	H	-1.73590000	0.94750000	2.11400000
	H	-1.73550000	-2.10440000	-0.90680000
	H	-3.97190000	-2.80210000	-0.19330000
	H	-3.94890000	0.21660000	2.85510000
	H	-5.10160000	-1.66760000	1.70820000
	C	0.56280000	-0.97660000	-0.88090000
	C	1.95510000	-2.71730000	-2.62270000
	C	0.77990000	-2.32420000	-0.54550000
	C	1.07430000	-0.52570000	-2.10310000
	C	1.75990000	-1.38380000	-2.96500000
	C	1.46350000	-3.18130000	-1.40310000
	H	0.42770000	-2.70030000	0.40500000
	H	0.94590000	0.50770000	-2.39550000
	H	2.14170000	-1.00020000	-3.90500000
	H	1.61450000	-4.21570000	-1.11370000
	H	2.48550000	-3.38570000	-3.29190000
	C	3.04770000	0.75270000	2.37740000
	H	2.66490000	1.73810000	2.67420000
	H	2.85310000	0.08060000	3.21810000
	C	4.55570000	0.84910000	2.12880000
	H	5.08200000	1.21580000	3.01460000
	H	4.97610000	-0.12860000	1.87410000
	H	4.78170000	1.53050000	1.30210000
**1b** (conf. 40)	C	0.91960000	0.17940000	1.29890000
	H	0.43250000	0.89160000	1.97550000
	C	2.31360000	0.71320000	0.93950000
	H	2.92280000	-0.13670000	0.61940000
	H	2.23580000	1.37080000	0.07100000
	O	1.12490000	-1.04570000	2.00910000
	H	0.27070000	-1.35610000	2.33300000
	C	-0.05690000	-0.01800000	0.05810000
	C	-0.66960000	1.33350000	-0.39620000
	C	-1.89240000	3.72650000	-1.28490000
	C	-1.73020000	1.31720000	-1.31690000
	C	-0.24530000	2.58250000	0.07060000
	C	-0.84990000	3.76440000	-0.36690000
	C	-2.33050000	2.49010000	-1.75980000
	H	-2.09470000	0.36780000	-1.69060000
	H	0.56310000	2.66560000	0.78270000
	H	-0.49690000	4.71500000	0.01800000
	H	-3.14690000	2.43790000	-2.47190000
	H	-2.36120000	4.64350000	-1.62360000
	C	-1.25950000	-0.88010000	0.53690000
	C	-3.50290000	-2.33870000	1.45100000

	C	-1.97010000	-0.48570000	1.68350000
	C	-1.71630000	-2.00990000	-0.14870000
	C	-2.82540000	-2.72910000	0.30050000
	C	-3.06920000	-1.20760000	2.14120000
	H	-1.67920000	0.41300000	2.21600000
	H	-1.20870000	-2.33700000	-1.04600000
	H	-3.15610000	-3.59920000	-0.25630000
	H	-3.59320000	-0.87750000	3.03150000
	H	-4.36070000	-2.90080000	1.80260000
	C	0.73710000	-0.68980000	-1.08720000
	C	2.29670000	-1.91330000	-3.10650000
	C	0.98320000	-0.04700000	-2.30580000
	C	1.30660000	-1.96120000	-0.90010000
	C	2.07220000	-2.56400000	-1.89390000
	C	1.75090000	-0.64990000	-3.30420000
	H	0.57920000	0.93940000	-2.48940000
	H	1.16420000	-2.47550000	0.04020000
	H	2.49700000	-3.54620000	-1.71650000
	H	1.91980000	-0.12220000	-4.23660000
	H	2.89120000	-2.38410000	-3.88160000
	C	3.02990000	1.47150000	2.07080000
	H	3.91390000	1.95470000	1.63890000
	H	2.38760000	2.28950000	2.42360000
	C	3.46850000	0.61880000	3.26640000
	H	4.00280000	1.22990000	4.00000000
	H	2.61800000	0.14920000	3.76300000
	H	4.13910000	-0.18440000	2.94660000
**1b** (conf. 82)	C	-0.80750000	0.53800000	1.30750000
	H	-1.23400000	-0.34580000	1.78200000
	C	-1.95400000	1.49590000	0.94530000
	H	-1.52100000	2.45190000	0.63610000
	H	-2.51050000	1.10870000	0.08680000
	O	0.00110000	1.11660000	2.33370000
	H	0.49290000	1.85550000	1.95430000
	C	0.07970000	0.02540000	0.09550000
	C	-0.82350000	-0.62040000	-0.98860000
	C	-2.40360000	-1.97220000	-2.90950000
	C	-1.84710000	-1.50110000	-0.60540000
	C	-0.59710000	-0.46010000	-2.36090000
	C	-1.37610000	-1.12430000	-3.30930000
	C	-2.63080000	-2.16060000	-1.54740000
	H	-2.03420000	-1.69600000	0.44310000
	H	0.20050000	0.18370000	-2.70700000
	H	-1.17080000	-0.97640000	-4.36410000
	H	-3.41600000	-2.83010000	-1.21360000
	H	-3.01150000	-2.48660000	-3.64510000
	C	1.03370000	-1.11670000	0.55450000
	C	2.75360000	-3.25170000	1.24470000
	C	1.11860000	-1.57840000	1.87230000
	C	1.83740000	-1.74840000	-0.40750000
	C	2.68750000	-2.79600000	-0.07190000

	C	1.96560000	-2.63700000	2.21090000
	H	0.55450000	-1.09920000	2.65880000
	H	1.79480000	-1.42100000	-1.43930000
	H	3.29370000	-3.26160000	-0.84150000
	H	2.00910000	-2.97110000	3.24200000
	H	3.41080000	-4.07230000	1.51010000
	C	0.89170000	1.24010000	-0.41170000
	C	2.32850000	3.55920000	-1.16980000
	C	0.30900000	2.23040000	-1.22090000
	C	2.20920000	1.45590000	0.01780000
	C	2.91870000	2.59870000	-0.35480000
	C	1.01650000	3.36860000	-1.59990000
	H	-0.70780000	2.11080000	-1.57050000
	H	2.69370000	0.72060000	0.64690000
	H	3.93700000	2.73030000	-0.00590000
	H	0.53810000	4.10810000	-2.23270000
	H	2.88010000	4.44450000	-1.46570000
	C	-2.92590000	1.75140000	2.11090000
	H	-2.35550000	2.07490000	2.98670000
	H	-3.57100000	2.59250000	1.83410000
	C	-3.80950000	0.55460000	2.48080000
	H	-4.39540000	0.21290000	1.62150000
	H	-3.22280000	-0.29470000	2.84330000
	H	-4.51150000	0.82120000	3.27580000
**1c** (conf. 4)	C	1.80840000	1.26360000	1.61620000
	C	0.55320000	0.33250000	1.60060000
	C	-0.06220000	-0.01890000	0.19320000
	O	0.80600000	-0.90870000	2.26660000
	C	1.05560000	-0.62670000	-0.68510000
	C	1.70330000	0.11260000	-1.68170000
	C	2.76890000	-0.42400000	-2.40600000
	C	3.21270000	-1.71730000	-2.14860000
	C	2.58300000	-2.46480000	-1.15380000
	C	1.52260000	-1.92610000	-0.43090000
	C	-1.25830000	-1.00210000	0.38240000
	C	-1.54000000	-2.00200000	-0.55550000
	C	-2.66720000	-2.81490000	-0.43720000
	C	-3.54740000	-2.64760000	0.62670000
	C	-3.28810000	-1.65010000	1.56470000
	C	-2.16430000	-0.83870000	1.43890000
	C	-0.70340000	1.23050000	-0.47380000
	C	-1.24610000	1.10430000	-1.76320000
	C	-1.88450000	2.16450000	-2.39510000
	C	-2.01170000	3.39530000	-1.75070000
	C	-1.49180000	3.53920000	-0.47060000
	C	-0.84730000	2.47020000	0.15810000
	C	1.78280000	2.14410000	2.88000000
	C	3.15790000	0.53050000	1.54160000
	H	1.74050000	1.92540000	0.74810000
	H	-0.23220000	0.85540000	2.15480000
	H	1.03390000	-0.72560000	3.18290000

	H	1.38170000	1.12210000	-1.90200000
	H	3.24890000	0.17540000	-3.17190000
	H	4.03790000	-2.13790000	-2.71260000
	H	2.92070000	-3.47200000	-0.93470000
	H	1.06110000	-2.51350000	0.35060000
	H	-0.87340000	-2.16030000	-1.39290000
	H	-2.85200000	-3.58060000	-1.18300000
	H	-4.42280000	-3.27990000	0.72330000
	H	-3.96410000	-1.49900000	2.39940000
	H	-2.00670000	-0.06700000	2.18140000
	H	-1.17280000	0.15690000	-2.28200000
	H	-2.28950000	2.02660000	-3.39160000
	H	-2.51220000	4.22380000	-2.23900000
	H	-1.58340000	4.48500000	0.05230000
	H	-0.46600000	2.63460000	1.15550000
	H	2.64040000	2.82180000	2.89950000
	H	0.87680000	2.75400000	2.94460000
	H	1.83600000	1.53520000	3.79080000
	H	3.30990000	-0.11520000	2.41050000
	H	3.96690000	1.26700000	1.53220000
	H	3.25500000	-0.08700000	0.65130000
**1c** (conf. 9)	C	1.80230000	1.10050000	1.74170000
	C	0.51760000	0.23140000	1.62450000
	C	-0.06710000	-0.01860000	0.16810000
	O	0.77640000	-0.99760000	2.30350000
	C	1.06440000	-0.55310000	-0.73440000
	C	1.57500000	-1.84810000	-0.54780000
	C	2.64890000	-2.31740000	-1.29870000
	C	3.25220000	-1.50160000	-2.25480000
	C	2.76800000	-0.21120000	-2.44420000
	C	1.68770000	0.25440000	-1.69350000
	C	-1.25630000	-1.01980000	0.29280000
	C	-1.47180000	-2.05530000	-0.62480000
	C	-2.58340000	-2.89240000	-0.52920000
	C	-3.51310000	-2.71990000	0.49150000
	C	-3.32590000	-1.68570000	1.40570000
	C	-2.22020000	-0.84480000	1.29980000
	C	-0.70800000	1.26720000	-0.42300000
	C	-0.75660000	2.49730000	0.24080000
	C	-1.38800000	3.60640000	-0.32970000
	C	-1.98670000	3.51240000	-1.57980000
	C	-1.95210000	2.29200000	-2.25550000
	C	-1.32710000	1.19150000	-1.68180000
	C	1.75610000	1.89910000	3.05750000
	C	3.12280000	0.31470000	1.67890000
	H	1.79780000	1.81120000	0.91020000
	H	-0.26720000	0.76120000	2.17340000
	H	-0.06040000	-1.46100000	2.42330000
	H	1.14230000	-2.49090000	0.20580000
	H	3.01940000	-3.32250000	-1.12900000
	H	4.08900000	-1.86680000	-2.83970000

	H	3.22780000	0.44190000	-3.17800000
	H	1.33480000	1.26320000	-1.86240000
	H	-0.76810000	-2.21750000	-1.43010000
	H	-2.71770000	-3.68290000	-1.25960000
	H	-4.37440000	-3.37360000	0.56930000
	H	-4.04710000	-1.52140000	2.19880000
	H	-2.13220000	-0.02460000	2.00290000
	H	-0.30550000	2.62260000	1.21400000
	H	-1.40580000	4.54460000	0.21410000
	H	-2.47630000	4.37260000	-2.02210000
	H	-2.41850000	2.19520000	-3.22980000
	H	-1.32250000	0.25250000	-2.22170000
	H	2.64540000	2.52670000	3.16150000
	H	0.88070000	2.55400000	3.11570000
	H	1.72320000	1.22010000	3.91460000
	H	3.95830000	1.01300000	1.78660000
	H	3.25350000	-0.21850000	0.73940000
	H	3.17920000	-0.41200000	2.49110000

**1c** (conf. 24)	C	1.99630000	0.63840000	1.85060000
	C	0.56410000	0.07600000	1.66640000
	C	-0.02150000	-0.03350000	0.18040000
	O	0.61910000	-1.22450000	2.26540000
	C	-0.68050000	1.27870000	-0.32970000
	C	-0.85320000	2.42790000	0.44730000
	C	-1.53540000	3.54430000	-0.04400000
	C	-2.06630000	3.53890000	-1.32780000
	C	-1.91560000	2.39710000	-2.11520000
	C	-1.24250000	1.28780000	-1.61830000
	C	1.11420000	-0.52780000	-0.74650000
	C	1.67100000	0.26880000	-1.75370000
	C	2.73970000	-0.18000000	-2.53150000
	C	3.28480000	-1.44150000	-2.31880000
	C	2.75240000	-2.24550000	-1.31210000
	C	1.68630000	-1.79520000	-0.53860000
	C	-1.22280000	-1.02960000	0.22120000
	C	-2.23800000	-0.84290000	1.17530000
	C	-3.35220000	-1.67640000	1.22520000
	C	-3.49560000	-2.71260000	0.30400000
	C	-2.51300000	-2.89540000	-0.66350000
	C	-1.39060000	-2.06640000	-0.70240000
	C	2.25740000	2.03910000	1.27870000
	C	2.36830000	0.63100000	3.34460000
	H	2.66260000	-0.06220000	1.33860000
	H	-0.12670000	0.69330000	2.25200000
	H	-0.27080000	-1.59510000	2.27860000
	H	-0.45950000	2.48200000	1.45110000
	H	-1.64670000	4.41850000	0.58830000
	H	-2.59500000	4.40510000	-1.70930000
	H	-2.33190000	2.36620000	-3.11620000
	H	-1.15830000	0.40580000	-2.24140000
	H	1.27530000	1.25710000	-1.94450000

	H	3.14350000	0.46590000	-3.30360000
	H	4.11360000	-1.79280000	-2.92340000
	H	3.16930000	-3.22880000	-1.12290000
	H	1.30490000	-2.42750000	0.25070000
	H	-2.17610000	-0.01920000	1.87800000
	H	-4.11410000	-1.50600000	1.97780000
	H	-4.36410000	-3.36040000	0.33660000
	H	-2.61280000	-3.68850000	-1.39660000
	H	-0.64360000	-2.23720000	-1.46570000
	H	1.99980000	2.13300000	0.22480000
	H	1.71270000	2.81000000	1.83170000
	H	3.32240000	2.26770000	1.37770000
	H	1.70960000	1.29730000	3.91440000
	H	3.39250000	0.99200000	3.47680000
	H	2.29540000	-0.36690000	3.77450000
**1c** (conf. 25)	C	1.92250000	1.10120000	1.70890000
	C	0.64310000	0.22010000	1.60500000
	C	-0.00730000	-0.06620000	0.17920000
	O	0.90280000	-1.02820000	2.25400000
	C	-1.31370000	-0.90670000	0.33270000
	C	-1.89730000	-1.23870000	1.55940000
	C	-3.11320000	-1.92490000	1.61530000
	C	-3.76950000	-2.30320000	0.44970000
	C	-3.19720000	-1.98460000	-0.78180000
	C	-1.99270000	-1.29260000	-0.83390000
	C	-0.50710000	1.23560000	-0.50240000
	C	-0.32760000	1.50510000	-1.86380000
	C	-0.87210000	2.64570000	-2.45640000
	C	-1.62170000	3.54290000	-1.70330000
	C	-1.83960000	3.27250000	-0.35300000
	C	-1.29940000	2.13220000	0.23140000
	C	1.05130000	-0.84950000	-0.63150000
	C	2.14390000	-0.20640000	-1.23700000
	C	3.14950000	-0.92590000	-1.87770000
	C	3.10330000	-2.31810000	-1.92060000
	C	2.04080000	-2.97690000	-1.31080000
	C	1.03040000	-2.25180000	-0.67660000
	C	1.82460000	2.52910000	1.15110000
	C	2.35000000	1.18410000	3.18760000
	H	2.71820000	0.57680000	1.16710000
	H	-0.12420000	0.68970000	2.22460000
	H	1.49980000	-1.54160000	1.69540000
	H	-1.40140000	-0.99730000	2.48750000
	H	-3.53870000	-2.16870000	2.58280000
	H	-4.71140000	-2.83810000	0.49670000
	H	-3.69280000	-2.26790000	-1.70400000
	H	-1.57630000	-1.04190000	-1.80240000
	H	0.23630000	0.82120000	-2.48360000
	H	-0.71020000	2.82400000	-3.51400000
	H	-2.04320000	4.43020000	-2.16200000
	H	-2.44130000	3.94650000	0.24700000

	H	-1.51850000	1.93480000	1.27350000
	H	2.21540000	0.87240000	-1.21450000
	H	3.97310000	-0.39390000	-2.34110000
	H	3.88590000	-2.87880000	-2.41930000
	H	1.98840000	-4.05990000	-1.32690000
	H	0.21050000	-2.78920000	-0.21820000
	H	1.04450000	3.10200000	1.66070000
	H	2.77410000	3.04520000	1.32260000
	H	1.61140000	2.57030000	0.08420000
	H	1.59630000	1.71670000	3.77890000
	H	3.28860000	1.73860000	3.27530000
	H	2.48660000	0.19800000	3.62930000
**1c** (conf. 36)	C	1.64620000	1.50640000	1.56500000
	C	0.55750000	0.38070000	1.57800000
	C	-0.05160000	-0.03690000	0.17180000
	O	0.98720000	-0.75880000	2.31770000
	C	-1.29340000	-0.95950000	0.36180000
	C	-1.81020000	-1.32740000	1.60840000
	C	-2.96010000	-2.11510000	1.70680000
	C	-3.61470000	-2.56090000	0.56440000
	C	-3.10700000	-2.20930000	-0.68630000
	C	-1.96740000	-1.41870000	-0.78070000
	C	-0.60650000	1.21600000	-0.55930000
	C	-1.33760000	2.17790000	0.15570000
	C	-1.91970000	3.27230000	-0.47760000
	C	-1.80400000	3.43280000	-1.85650000
	C	-1.11060000	2.47420000	-2.58830000
	C	-0.52420000	1.38240000	-1.94810000
	C	1.05540000	-0.78110000	-0.60900000
	C	2.05400000	-0.10000000	-1.32390000
	C	3.09500000	-0.78200000	-1.94890000
	C	3.18060000	-2.17040000	-1.86410000
	C	2.21200000	-2.86380000	-1.14510000
	C	1.16360000	-2.17790000	-0.53010000
	C	1.37320000	2.50670000	2.70110000
	C	3.08620000	0.98160000	1.69280000
	H	1.56230000	2.05050000	0.62050000
	H	-0.27760000	0.75570000	2.16880000
	H	1.66730000	-1.22100000	1.81350000
	H	-1.30780000	-1.03680000	2.51900000
	H	-3.33430000	-2.38460000	2.68850000
	H	-4.50550000	-3.17400000	0.64370000
	H	-3.60190000	-2.54620000	-1.59070000
	H	-1.59840000	-1.15110000	-1.76360000
	H	-1.48290000	2.07140000	1.22270000
	H	-2.47390000	3.99560000	0.11050000
	H	-2.25740000	4.28370000	-2.35220000
	H	-1.02470000	2.56780000	-3.66550000
	H	-0.00400000	0.65130000	-2.55200000
	H	2.02450000	0.97890000	-1.39870000
	H	3.84380000	-0.22290000	-2.49950000

	H	3.99090000	-2.70180000	-2.35050000
	H	2.26200000	-3.94400000	-1.06370000
	H	0.41330000	-2.74220000	0.00880000
	H	1.38490000	2.00000000	3.67150000
	H	2.13690000	3.28960000	2.72450000
	H	0.40210000	2.99530000	2.58470000
	H	3.34190000	0.24340000	0.93080000
	H	3.25020000	0.53140000	2.67490000
	H	3.78910000	1.81290000	1.58680000
**1d** (conf. 2)	C	-0.94640000	0.02140000	1.08650000
	H	-0.89380000	-0.93220000	1.61140000
	O	-0.77150000	0.98950000	2.12260000
	H	-0.69490000	1.86190000	1.71670000
	C	-2.34710000	0.16300000	0.47020000
	H	-2.45470000	1.18450000	0.09230000
	H	-2.45120000	-0.50430000	-0.39140000
	C	-3.49880000	-0.11090000	1.46050000
	H	-3.30240000	0.47540000	2.36490000
	C	-3.59460000	-1.59030000	1.86110000
	H	-4.42210000	-1.74940000	2.55870000
	H	-3.77300000	-2.22340000	0.98470000
	H	-2.68620000	-1.94720000	2.35390000
	C	-4.82990000	0.36740000	0.86310000
	H	-5.65720000	0.20440000	1.56020000
	H	-4.79950000	1.43490000	0.62480000
	H	-5.06420000	-0.17370000	-0.06080000
	C	0.26900000	0.04830000	0.06380000
	C	1.59480000	-0.35600000	0.77360000
	C	4.05000000	-1.15340000	1.92600000
	C	1.69010000	-0.65090000	2.13770000
	C	2.76280000	-0.46500000	0.00290000
	C	3.97320000	-0.85350000	0.56600000
	C	2.90330000	-1.04950000	2.70500000
	H	0.83130000	-0.54230000	2.78360000
	H	2.72610000	-0.24900000	-1.05810000
	H	4.85580000	-0.92840000	-0.05990000
	H	2.94420000	-1.27070000	3.76600000
	H	4.99050000	-1.46180000	2.36870000
	C	0.06210000	-1.02590000	-1.03640000
	C	-0.17540000	-3.09110000	-2.95810000
	C	0.38860000	-0.81090000	-2.38070000
	C	-0.36000000	-2.31550000	-0.67680000
	C	-0.48540000	-3.33030000	-1.62040000
	C	0.27010000	-1.82720000	-3.32980000
	H	0.75010000	0.15660000	-2.70260000
	H	-0.58130000	-2.54750000	0.35740000
	H	-0.81800000	-4.31340000	-1.30570000
	H	0.53350000	-1.62390000	-4.36220000
	H	-0.27010000	-3.88100000	-3.69450000
	C	0.36520000	1.49170000	-0.48240000
	C	0.42200000	4.19230000	-1.33290000

	C	-0.51510000	1.96740000	-1.46920000
	C	1.26230000	2.41430000	0.07480000
	C	1.29180000	3.74580000	-0.34320000
	C	-0.48440000	3.29380000	-1.89280000
	H	-1.23070000	1.29520000	-1.92370000
	H	1.95400000	2.09060000	0.84170000
	H	2.00180000	4.42980000	0.10860000
	H	-1.17270000	3.62370000	-2.66330000
	H	0.44750000	5.22450000	-1.66360000
**1d** (conf. 20)	C	-0.94440000	0.13120000	1.06370000
	H	-0.84920000	-0.70670000	1.75660000
	O	-0.82750000	1.27990000	1.90440000
	H	-0.81580000	2.06730000	1.34670000
	C	-2.34070000	0.05320000	0.41780000
	H	-2.61550000	1.04410000	0.03800000
	H	-2.29800000	-0.61150000	-0.44820000
	C	-3.46210000	-0.46340000	1.34490000
	H	-3.12140000	-1.41870000	1.76840000
	C	-4.72700000	-0.74920000	0.52120000
	H	-5.52490000	-1.15230000	1.15180000
	H	-5.10520000	0.16850000	0.05710000
	H	-4.53280000	-1.47150000	-0.27740000
	C	-3.78500000	0.48200000	2.51090000
	H	-4.14360000	1.44710000	2.13540000
	H	-4.57360000	0.05810000	3.14060000
	H	-2.91170000	0.67820000	3.13320000
	C	0.27880000	0.03950000	0.05330000
	C	1.62130000	-0.14090000	0.82020000
	C	4.11320000	-0.55680000	2.08820000
	C	2.80040000	-0.30960000	0.07750000
	C	1.72370000	-0.18050000	2.21480000
	C	2.95560000	-0.39110000	2.83990000
	C	4.02880000	-0.51040000	0.69680000
	H	2.75860000	-0.29000000	-1.00490000
	H	0.85420000	-0.01450000	2.83340000
	H	3.00200000	-0.41590000	3.92330000
	H	4.91960000	-0.63790000	0.09140000
	H	5.06800000	-0.71880000	2.57600000
	C	0.14740000	-1.23280000	-0.82420000
	C	0.03780000	-3.62930000	-2.32590000
	C	-0.20250000	-2.45450000	-0.22780000
	C	0.46830000	-1.25320000	-2.18670000
	C	0.41360000	-2.43370000	-2.92900000
	C	-0.26530000	-3.63270000	-0.96540000
	H	-0.41370000	-2.50180000	0.83320000
	H	0.77220000	-0.34190000	-2.68440000
	H	0.67030000	-2.41180000	-3.98260000
	H	-0.54340000	-4.55740000	-0.47170000
	H	-0.00850000	-4.54650000	-2.90200000
	C	0.28460000	1.35930000	-0.75320000
	C	0.16140000	3.85590000	-2.08320000

	C	-0.62150000	1.58510000	-1.80340000
	C	1.11370000	2.42710000	-0.38030000
	C	1.05450000	3.65850000	-1.03510000
	C	-0.67900000	2.81060000	-2.46240000
	H	-1.28830000	0.79460000	-2.12050000
	H	1.81980000	2.29730000	0.42950000
	H	1.71360000	4.46050000	-0.72130000
	H	-1.38450000	2.94690000	-3.27480000
	H	0.11800000	4.80950000	-2.59730000
**1d** (conf. 25)	C	-0.88630000	-0.23250000	1.11670000
	H	-0.73580000	-1.25370000	1.47990000
	O	-0.80750000	0.65840000	2.23380000
	H	0.03210000	0.51160000	2.68470000
	C	-2.31350000	-0.08810000	0.57890000
	H	-2.47950000	0.97320000	0.37870000
	H	-2.40710000	-0.59880000	-0.38430000
	C	-3.41530000	-0.58520000	1.53780000
	H	-3.23430000	-0.11810000	2.51150000
	C	-3.40540000	-2.10930000	1.72830000
	H	-2.45890000	-2.47260000	2.13880000
	H	-4.19470000	-2.41830000	2.41990000
	H	-3.58240000	-2.62580000	0.77770000
	C	-4.79020000	-0.12100000	1.03580000
	H	-5.58630000	-0.43330000	1.71840000
	H	-4.83320000	0.96820000	0.94640000
	H	-5.01300000	-0.54540000	0.05020000
	C	0.26620000	0.05370000	0.05680000
	C	1.60190000	0.20860000	0.84060000
	C	4.05450000	0.34290000	2.24350000
	C	1.96720000	-0.77840000	1.77210000
	C	2.50770000	1.25060000	0.61760000
	C	3.71940000	1.31630000	1.30780000
	C	3.16950000	-0.70990000	2.47070000
	H	1.31890000	-1.63140000	1.93840000
	H	2.27510000	2.02150000	-0.10450000
	H	4.40080000	2.13620000	1.10820000
	H	3.41910000	-1.48780000	3.18400000
	H	4.99340000	0.39720000	2.78260000
	C	0.49460000	-1.15910000	-0.88450000
	C	1.05970000	-3.32600000	-2.61670000
	C	1.59050000	-1.13000000	-1.76320000
	C	-0.30360000	-2.30750000	-0.89530000
	C	-0.02430000	-3.37850000	-1.74930000
	C	1.86880000	-2.18930000	-2.61860000
	H	2.24040000	-0.26320000	-1.77530000
	H	-1.15940000	-2.39720000	-0.24240000
	H	-0.66430000	-4.25390000	-1.72880000
	H	2.72350000	-2.12980000	-3.28360000
	H	1.27600000	-4.15660000	-3.27900000
	C	-0.11190000	1.32320000	-0.73890000
	C	-0.95120000	3.64590000	-2.11900000

	C	-0.46140000	1.27770000	-2.09360000
	C	-0.20810000	2.56460000	-0.08720000
	C	-0.61870000	3.70820000	-0.76640000
	C	-0.87410000	2.42300000	-2.77660000
	H	-0.41980000	0.34060000	-2.63250000
	H	0.02120000	2.63290000	0.96700000
	H	-0.68170000	4.65070000	-0.23330000
	H	-1.13730000	2.35060000	-3.82640000
	H	-1.26920000	4.53690000	-2.64880000
**1d** (conf. 26)	C	-1.15490000	0.04630000	0.83720000
	H	-1.16070000	-0.86800000	1.43010000
	O	-1.16210000	1.08650000	1.81600000
	H	-1.08560000	1.93440000	1.36180000
	C	-2.43400000	0.08610000	-0.01660000
	H	-2.55340000	1.09770000	-0.41790000
	H	-2.30620000	-0.57620000	-0.87720000
	C	-3.75550000	-0.31430000	0.68830000
	H	-4.51080000	-0.28790000	-0.10850000
	C	-4.21700000	0.67360000	1.77010000
	H	-3.53790000	0.68300000	2.62430000
	H	-4.26690000	1.69450000	1.37890000
	H	-5.21660000	0.40490000	2.12670000
	C	-3.73460000	-1.75280000	1.22720000
	H	-4.73040000	-2.04670000	1.57160000
	H	-3.42490000	-2.46670000	0.45790000
	H	-3.05760000	-1.85860000	2.08090000
	C	0.20980000	0.05210000	0.01970000
	C	0.22450000	-1.11020000	-1.00810000
	C	0.37920000	-3.32450000	-2.76500000
	C	0.74990000	-0.97990000	-2.29920000
	C	-0.19470000	-2.39000000	-0.61170000
	C	-0.12810000	-3.47840000	-1.47590000
	C	0.82540000	-2.07010000	-3.16720000
	H	1.11600000	-0.02090000	-2.64060000
	H	-0.56380000	-2.55500000	0.39270000
	H	-0.46450000	-4.45170000	-1.13560000
	H	1.23930000	-1.93160000	-4.16020000
	H	0.43480000	-4.17160000	-3.43920000
	C	0.34090000	1.45510000	-0.61750000
	C	0.43400000	4.08820000	-1.65600000
	C	1.09480000	2.46020000	0.00580000
	C	-0.37970000	1.81390000	-1.76950000
	C	-0.32980000	3.10670000	-2.28510000
	C	1.14200000	3.75840000	-0.50480000
	H	1.65910000	2.22800000	0.89940000
	H	-0.98300000	1.07550000	-2.28030000
	H	-0.89180000	3.34550000	-3.18140000
	H	1.73870000	4.50850000	0.00250000
	H	0.47420000	5.09430000	-2.05800000
	C	1.41800000	-0.23540000	0.95880000
	C	3.68770000	-0.82650000	2.53710000

	C	1.30480000	-0.43920000	2.33810000
	C	2.69860000	-0.33000000	0.39170000
	C	3.81830000	-0.61630000	1.16440000
	C	2.42700000	-0.73650000	3.11590000
	H	0.34970000	-0.33480000	2.83140000
	H	2.82380000	-0.18270000	-0.67440000
	H	4.79260000	-0.68190000	0.69240000
	H	2.30610000	-0.88820000	4.18310000
	H	4.55700000	-1.05520000	3.14340000
**1e** (conf. 1)	C	-0.56530000	0.18730000	1.20050000
	H	-0.44460000	-0.66200000	1.87620000
	C	-2.06090000	0.22770000	0.77020000
	C	-4.42880000	0.53070000	1.69850000
	C	-4.07240000	-0.81000000	-0.40700000
	C	-4.94410000	-0.62910000	0.84010000
	C	-2.58290000	-0.96440000	-0.05700000
	C	-2.94060000	0.37230000	2.03630000
	H	-4.57690000	1.47500000	1.15790000
	H	-4.19880000	0.06010000	-1.06530000
	H	-4.92420000	-1.55370000	1.43200000
	H	-2.44350000	-1.88920000	0.51650000
	H	-2.59850000	1.21830000	2.63270000
	H	-2.20140000	1.13860000	0.17310000
	H	-5.01090000	0.60980000	2.62280000
	H	-4.40350000	-1.68180000	-0.98120000
	H	-5.98870000	-0.46340000	0.55570000
	H	-2.01270000	-1.09620000	-0.97640000
	H	-2.80620000	-0.52320000	2.65980000
	C	0.57710000	0.08150000	0.09490000
	O	-0.30210000	1.32830000	2.02380000
	H	-0.30680000	2.11380000	1.46270000
	C	1.98340000	0.00000000	0.76780000
	C	4.58760000	-0.29800000	1.82920000
	C	3.11450000	-0.02530000	-0.06340000
	C	2.19160000	-0.12960000	2.14450000
	C	3.47850000	-0.28070000	2.66700000
	C	4.39730000	-0.16560000	0.45390000
	H	2.99130000	0.05960000	-1.13670000
	H	1.36190000	-0.08380000	2.83360000
	H	3.60560000	-0.37600000	3.74000000
	H	5.24780000	-0.17930000	-0.21910000
	H	5.58530000	-0.41270000	2.23800000
	C	0.49730000	-1.24900000	-0.70030000
	C	0.53400000	-3.74070000	-2.04100000
	C	0.70090000	-1.32760000	-2.08280000
	C	0.35570000	-2.45810000	-0.00140000
	C	0.36360000	-3.68360000	-0.65850000
	C	0.71360000	-2.55550000	-2.74620000
	H	0.86060000	-0.42720000	-2.66020000
	H	0.25630000	-2.45340000	1.07690000
	H	0.24590000	-4.59740000	-0.08630000

	H	0.87180000	-2.57860000	-3.81910000
	H	0.54250000	-4.69490000	-2.55560000
	C	0.46160000	1.35720000	-0.77060000
	C	0.11770000	3.78190000	-2.19620000
	C	1.23750000	2.49110000	-0.48510000
	C	-0.50590000	1.48260000	-1.78120000
	C	-0.67090000	2.67080000	-2.48870000
	C	1.06900000	3.68660000	-1.18580000
	H	1.99250000	2.44080000	0.28870000
	H	-1.14050000	0.64250000	-2.02880000
	H	-1.42220000	2.72670000	-3.26890000
	H	1.68990000	4.54060000	-0.93810000
	H	-0.01030000	4.70700000	-2.74690000
**1e** (conf. 14)	C	0.52690000	0.25350000	1.17170000
	H	0.23750000	1.18800000	1.66290000
	C	1.98220000	0.45630000	0.66760000
	C	4.25710000	-0.53110000	0.09310000
	C	4.13480000	1.77650000	1.10900000
	C	4.96650000	0.49430000	0.98480000
	C	2.70430000	1.47750000	1.57640000
	C	2.83110000	-0.83050000	0.57980000
	H	4.21940000	-0.15040000	-0.93640000
	H	4.09700000	2.27700000	0.13220000
	H	5.11620000	0.06280000	1.98310000
	H	2.72650000	1.07670000	2.59750000
	H	2.36650000	-1.55720000	-0.08540000
	H	1.92970000	0.88350000	-0.34050000
	H	4.83250000	-1.46220000	0.05560000
	H	4.61370000	2.47900000	1.79950000
	H	5.96230000	0.72360000	0.59040000
	H	2.13330000	2.41270000	1.62480000
	H	2.87160000	-1.29330000	1.57030000
	C	-0.59670000	-0.02850000	0.08210000
	O	0.55620000	-0.77200000	2.16560000
	H	-0.28700000	-0.76350000	2.63300000
	C	-1.93200000	-0.31570000	0.83590000
	C	-4.40490000	-0.69080000	2.16400000
	C	-2.81860000	-1.32880000	0.45140000
	C	-2.33290000	0.52210000	1.89010000
	C	-3.54420000	0.33370000	2.55160000
	C	-4.03760000	-1.51400000	1.10400000
	H	-2.56360000	-1.98530000	-0.36940000
	H	-1.70960000	1.35680000	2.18840000
	H	-3.81850000	0.99890000	3.36300000
	H	-4.69980000	-2.30850000	0.77730000
	H	-5.34990000	-0.83800000	2.67440000
	C	-0.90390000	1.23190000	-0.77190000
	C	-1.63110000	3.47950000	-2.33200000
	C	-0.26720000	2.46640000	-0.60800000
	C	-1.92320000	1.15750000	-1.73590000
	C	-2.28020000	2.25670000	-2.50720000

	C	-0.62610000	3.57690000	-1.37770000
	H	0.52100000	2.59320000	0.11930000
	H	-2.44980000	0.22230000	-1.88250000
	H	-3.07150000	2.16040000	-3.24260000
	H	-0.11030000	4.51830000	-1.22340000
	H	-1.90920000	4.34070000	-2.92890000
	C	-0.14570000	-1.21200000	-0.79910000
	C	0.83470000	-3.37650000	-2.33380000
	C	0.32500000	-1.02380000	-2.10430000
	C	-0.10100000	-2.51530000	-0.27730000
	C	0.37670000	-3.58190000	-1.03320000
	C	0.81030000	-2.09000000	-2.86270000
	H	0.31890000	-0.03510000	-2.54380000
	H	-0.42360000	-2.69720000	0.73810000
	H	0.39720000	-4.57580000	-0.59940000
	H	1.16820000	-1.90750000	-3.87020000
	H	1.20660000	-4.20710000	-2.92340000
**1e** (conf. 29)	C	0.52440000	0.25430000	1.18540000
	H	0.24180000	1.18770000	1.68260000
	C	1.98200000	0.45310000	0.66120000
	C	4.24540000	-0.54180000	0.05500000
	C	4.14190000	1.78050000	1.04600000
	C	4.97140000	0.49760000	0.91710000
	C	2.71930000	1.48760000	1.54270000
	C	2.82990000	-0.83360000	0.57670000
	H	4.18330000	-0.17680000	-0.97850000
	H	4.63450000	2.49010000	1.71920000
	H	5.14710000	0.07850000	1.91690000
	H	2.77290000	1.11010000	2.57510000
	H	2.34730000	-1.57420000	-0.05890000
	H	1.91960000	0.86910000	-0.35060000
	H	4.82120000	-1.47250000	0.01890000
	H	4.08150000	2.27150000	0.06610000
	H	5.95710000	0.72420000	0.49740000
	H	2.14860000	2.42200000	1.59190000
	H	2.90540000	-1.28720000	1.57190000
	C	-0.59600000	-0.02870000	0.11260000
	O	0.44160000	-0.79600000	2.15450000
	H	1.04290000	-0.59400000	2.87770000
	C	-0.16020000	-1.24710000	-0.73330000
	C	0.78710000	-3.47930000	-2.18510000
	C	0.36120000	-1.10490000	-2.02450000
	C	-0.18660000	-2.53770000	-0.18080000
	C	0.27670000	-3.63770000	-0.89690000
	C	0.83020000	-2.20570000	-2.74330000
	H	0.40960000	-0.12630000	-2.48370000
	H	-0.55770000	-2.67750000	0.82500000
	H	0.24240000	-4.62230000	-0.44310000
	H	1.22790000	-2.06050000	-3.74190000
	H	1.14620000	-4.33690000	-2.74320000
	C	-1.95330000	-0.27140000	0.84160000

	C	-4.48970000	-0.55230000	2.06420000
	C	-2.33170000	0.52810000	1.92880000
	C	-2.88760000	-1.20180000	0.37370000
	C	-4.13870000	-1.34280000	0.97530000
	C	-3.57580000	0.38840000	2.53590000
	H	-1.65650000	1.28080000	2.31560000
	H	-2.64480000	-1.83520000	-0.46930000
	H	-4.83640000	-2.07660000	0.58630000
	H	-3.83140000	1.01980000	3.38000000
	H	-5.45930000	-0.66270000	2.53650000
	C	-0.86220000	1.21920000	-0.77560000
	C	-1.53020000	3.44980000	-2.38940000
	C	-0.26650000	2.46860000	-0.57420000
	C	-1.81340000	1.12320000	-1.80500000
	C	-2.14090000	2.21350000	-2.60160000
	C	-0.59470000	3.57000000	-1.36970000
	H	0.46270000	2.61790000	0.20860000
	H	-2.31240000	0.17900000	-1.98330000
	H	-2.88030000	2.09880000	-3.38670000
	H	-0.11170000	4.52280000	-1.18190000
	H	-1.78610000	4.30370000	-3.00640000
**3** (conf. 1)	C	-3.07120000	1.43030000	-1.74810000
	C	-3.76690000	0.14850000	-2.22140000
	C	-2.74640000	-0.95470000	-2.52100000
	C	-1.78640000	-1.15830000	-1.34100000
	C	-1.02770000	0.14600000	-0.98510000
	C	-2.11570000	1.17660000	-0.57120000
	C	0.16790000	-0.08240000	0.03180000
	C	-0.45070000	-0.62430000	1.34270000
	C	-0.51520000	-1.99820000	1.61110000
	C	-1.15910000	-2.48880000	2.74670000
	C	-1.76300000	-1.61470000	3.64590000
	C	-1.72670000	-0.24650000	3.38590000
	C	-1.08540000	0.24040000	2.24860000
	C	1.23960000	-1.05490000	-0.55130000
	C	2.22990000	-1.57250000	0.29850000
	C	3.26900000	-2.36000000	-0.18540000
	C	3.35940000	-2.64780000	-1.54670000
	C	2.39990000	-2.12870000	-2.40850000
	C	1.35760000	-1.34120000	-1.91620000
	C	1.00590000	1.21860000	0.23100000
	C	1.54430000	1.58410000	1.46990000
	C	2.36520000	2.70340000	1.60460000
	C	2.68240000	3.48770000	0.50010000
	C	2.18470000	3.12100000	-0.74850000
	C	1.37050000	1.99820000	-0.87930000
	H	-2.50180000	1.87560000	-2.57550000
	H	-3.80460000	2.17740000	-1.43220000
	H	-4.45040000	-0.19890000	-1.43650000
	H	-4.38440000	0.35530000	-3.10130000
	H	-3.25880000	-1.89550000	-2.74740000

	H	-2.17280000	-0.69020000	-3.41920000
	H	-2.35760000	-1.48700000	-0.46660000
	H	-1.08750000	-1.96430000	-1.56700000
	H	-0.58630000	0.52420000	-1.91320000
	H	-2.69490000	0.74640000	0.25090000
	O	-1.63960000	2.41580000	-0.04370000
	H	-0.05890000	-2.70280000	0.92820000
	H	-1.18620000	-3.55850000	2.92380000
	H	-2.25960000	-1.99350000	4.53220000
	H	-2.20250000	0.45040000	4.06740000
	H	-1.08880000	1.30540000	2.05590000
	H	2.19260000	-1.35180000	1.35810000
	H	4.01370000	-2.74380000	0.50340000
	H	4.16890000	-3.26010000	-1.92770000
	H	2.45550000	-2.33250000	-3.47240000
	H	0.64020000	-0.95230000	-2.62610000
	H	1.32420000	0.99590000	2.35030000
	H	2.75710000	2.95900000	2.58310000
	H	3.31620000	4.36060000	0.60600000
	H	2.43930000	3.70100000	-1.62900000
	H	1.04230000	1.71980000	-1.87350000
	H	-0.91000000	2.74350000	-0.58080000
**3** (conf. 6)	C	3.26570000	0.12150000	-1.99450000
	C	3.77440000	1.52300000	-1.64700000
	C	2.60570000	2.48230000	-1.40550000
	C	1.62740000	1.90490000	-0.37410000
	C	1.04000000	0.54680000	-0.83880000
	C	2.25300000	-0.42040000	-0.97940000
	C	-0.18450000	0.02150000	0.04480000
	C	0.39760000	-0.62630000	1.32580000
	C	0.98830000	0.15940000	2.32990000
	C	1.60580000	-0.41470000	3.43810000
	C	1.65660000	-1.80000000	3.58050000
	C	1.07300000	-2.59790000	2.60240000
	C	0.45030000	-2.01720000	1.49630000
	C	-1.15810000	1.20000000	0.34920000
	C	-1.71490000	1.43350000	1.61010000
	C	-2.65140000	2.45010000	1.81390000
	C	-3.05700000	3.25810000	0.75820000
	C	-2.52570000	3.02740000	-0.51090000
	C	-1.59960000	2.00990000	-0.70970000
	C	-1.10470000	-0.98170000	-0.72190000
	C	-2.22220000	-1.50540000	-0.04870000
	C	-3.12800000	-2.34870000	-0.68080000
	C	-2.95200000	-2.68760000	-2.02290000
	C	-1.85830000	-2.17320000	-2.70790000
	C	-0.94550000	-1.33180000	-2.06550000
	H	2.78580000	0.12880000	-2.98120000
	H	4.09310000	-0.59070000	-2.05610000
	H	4.39090000	1.47290000	-0.74060000
	H	4.42550000	1.89380000	-2.44510000

	H	2.97360000	3.45430000	-1.06060000
	H	2.07660000	2.66580000	-2.34950000
	H	2.16650000	1.77010000	0.56930000
	H	0.83170000	2.62400000	-0.18060000
	H	0.64260000	0.71240000	-1.84440000
	H	2.74040000	-0.48980000	0.00230000
	O	1.93730000	-1.73810000	-1.43430000
	H	0.95700000	1.23770000	2.25710000
	H	2.04690000	0.22620000	4.19380000
	H	2.13760000	-2.24760000	4.44280000
	H	1.09210000	-3.67820000	2.69600000
	H	-0.02250000	-2.66790000	0.77080000
	H	-1.43130000	0.81690000	2.45230000
	H	-3.06310000	2.60200000	2.80590000
	H	-3.78180000	4.04880000	0.91570000
	H	-2.84030000	3.63590000	-1.35180000
	H	-1.22590000	1.83740000	-1.71200000
	H	-2.39300000	-1.24150000	0.98810000
	H	-3.97720000	-2.73400000	-0.12700000
	H	-3.65850000	-3.34060000	-2.52290000
	H	-1.69960000	-2.42720000	-3.75040000
	H	-0.09340000	-0.98360000	-2.62840000
	H	1.51390000	-2.22140000	-0.71770000
**3** (conf. 19)	C	3.21940000	-0.09310000	-2.07750000
	C	3.80740000	1.28480000	-1.75790000
	C	2.68570000	2.30700000	-1.55770000
	C	1.67420000	1.81600000	-0.51380000
	C	1.02680000	0.46470000	-0.91320000
	C	2.18100000	-0.55510000	-1.04310000
	C	-0.17380000	0.02950000	0.04050000
	C	0.45430000	-0.44160000	1.37480000
	C	0.88180000	0.47580000	2.34670000
	C	1.53320000	0.05970000	3.50710000
	C	1.78110000	-1.29260000	3.72780000
	C	1.37510000	-2.21780000	2.76920000
	C	0.72460000	-1.79710000	1.61040000
	C	-1.17560000	1.21210000	0.22360000
	C	-1.85740000	1.43750000	1.42570000
	C	-2.83150000	2.43000000	1.53630000
	C	-3.15700000	3.22510000	0.44230000
	C	-2.50430000	3.00250000	-0.76870000
	C	-1.53700000	2.00740000	-0.87420000
	C	-1.09720000	-1.06340000	-0.58330000
	C	-2.04210000	-1.69910000	0.23650000
	C	-2.97070000	-2.59830000	-0.27800000
	C	-2.99250000	-2.88060000	-1.64260000
	C	-2.07130000	-2.25090000	-2.47300000
	C	-1.13790000	-1.35590000	-1.94960000
	H	2.73730000	-0.07840000	-3.06250000
	H	4.01840000	-0.84330000	-2.13060000
	H	4.40860000	1.22390000	-0.84210000

	H	4.48650000	1.59900000	-2.55680000
	H	3.09920000	3.27100000	-1.24390000
	H	2.17390000	2.48310000	-2.51270000
	H	2.19090000	1.70340000	0.44470000
	H	0.90480000	2.57370000	-0.36570000
	H	0.61140000	0.60190000	-1.91540000
	H	2.66950000	-0.63600000	-0.06270000
	O	1.66930000	-1.84060000	-1.41490000
	H	0.69750000	1.53290000	2.20870000
	H	1.84270000	0.79760000	4.23930000
	H	2.28260000	-1.61980000	4.63180000
	H	1.56340000	-3.27550000	2.91990000
	H	0.43530000	-2.53160000	0.87180000
	H	-1.63870000	0.83100000	2.29430000
	H	-3.33740000	2.57470000	2.48480000
	H	-3.91250000	3.99800000	0.52690000
	H	-2.75310000	3.59940000	-1.63950000
	H	-1.07140000	1.84580000	-1.83840000
	H	-2.05850000	-1.48710000	1.29840000
	H	-3.68220000	-3.07270000	0.38930000
	H	-3.71610000	-3.57770000	-2.05010000
	H	-2.06750000	-2.45830000	-3.53770000
	H	-0.42600000	-0.91370000	-2.63030000
	H	2.40900000	-2.45600000	-1.45230000
**4** (conf. 1)	C	-1.97680000	-1.02570000	-1.17470000
	C	-3.01570000	-0.76220000	-2.27140000
	C	-3.92030000	0.41980000	-1.90930000
	C	-3.09050000	1.67140000	-1.55970000
	C	-2.02800000	1.36190000	-0.51020000
	C	-1.08160000	0.22210000	-0.91580000
	O	-1.99070000	1.96420000	0.53900000
	H	-1.35000000	-1.87870000	-1.44100000
	H	-2.48640000	-1.30180000	-0.24670000
	H	-3.61950000	-1.66150000	-2.42880000
	H	-2.50640000	-0.55870000	-3.22270000
	H	-4.53840000	0.15180000	-1.04460000
	H	-4.60900000	0.65020000	-2.72750000
	H	-3.71700000	2.47880000	-1.17740000
	H	-2.58310000	2.03380000	-2.46320000
	C	0.17590000	-0.04970000	-0.00200000
	H	-0.70610000	0.53530000	-1.89560000
	C	0.85580000	1.29190000	0.39430000
	C	1.37920000	1.52890000	1.66870000
	C	2.06040000	2.71050000	1.96280000
	C	2.24090000	3.68430000	0.98620000
	C	1.74380000	3.45450000	-0.29530000
	C	1.07070000	2.27220000	-0.58500000
	C	-0.27240000	-0.90210000	1.21580000
	C	-0.03800000	-2.28250000	1.26770000
	C	-0.51150000	-3.06300000	2.32300000
	C	-1.23510000	-2.48080000	3.35860000

	C	-1.48450000	-1.11020000	3.31990000
	C	-1.01430000	-0.33330000	2.26360000
	C	1.28240000	-0.80140000	-0.80780000
	C	2.49670000	-1.08870000	-0.16200000
	C	3.54310000	-1.72240000	-0.82010000
	C	3.41390000	-2.08820000	-2.16060000
	C	2.22400000	-1.80930000	-2.82010000
	C	1.17280000	-1.17530000	-2.15060000
	H	1.25610000	0.79190000	2.45080000
	H	2.44830000	2.86520000	2.96390000
	H	2.76570000	4.60480000	1.21580000
	H	1.88530000	4.19450000	-1.07550000
	H	0.72350000	2.11550000	-1.59980000
	H	0.52130000	-2.76710000	0.47850000
	H	-0.30920000	-4.12860000	2.32960000
	H	-1.60050000	-3.08410000	4.18230000
	H	-2.05070000	-0.63730000	4.11520000
	H	-1.23560000	0.72400000	2.24440000
	H	2.62770000	-0.80450000	0.87470000
	H	4.46530000	-1.92500000	-0.28660000
	H	4.22980000	-2.57890000	-2.67900000
	H	2.10070000	-2.08310000	-3.86230000
	H	0.26830000	-0.98610000	-2.71200000
**5** (conf. 1)	C	-1.93510000	-0.78490000	-1.31700000
	C	-2.95310000	-0.34690000	-2.38240000
	C	-3.83780000	0.80270000	-1.88430000
	C	-2.99290000	1.96370000	-1.34290000
	C	-1.99490000	1.51040000	-0.26040000
	C	-1.07050000	0.40270000	-0.84000000
	O	-2.68900000	1.15560000	0.94100000
	H	-1.30630000	-1.58630000	-1.70940000
	H	-2.46250000	-1.23110000	-0.46520000
	H	-3.57010000	-1.20080000	-2.68050000
	H	-2.41520000	-0.02370000	-3.28380000
	H	-4.50600000	0.43490000	-1.09470000
	H	-4.49250000	1.15740000	-2.68650000
	H	-3.63310000	2.73970000	-0.91410000
	H	-2.43440000	2.42630000	-2.16650000
	C	0.20580000	-0.03250000	0.00560000
	H	-0.66920000	0.87650000	-1.74060000
	C	0.89450000	1.21730000	0.62580000
	C	1.39310000	1.25990000	1.93190000
	C	2.07910000	2.37750000	2.41210000
	C	2.29110000	3.48220000	1.59530000
	C	1.82270000	3.44930000	0.28250000
	C	1.14470000	2.33160000	-0.19180000
	C	-0.20710000	-1.10460000	1.05110000
	C	-0.00790000	-2.47010000	0.80290000
	C	-0.44210000	-3.44820000	1.69770000
	C	-1.08720000	-3.08650000	2.87580000
	C	-1.30010000	-1.73470000	3.13910000

	C	-0.87120000	-0.76120000	2.23910000
	C	1.30940000	-0.62190000	-0.93320000
	C	2.52610000	-1.02300000	-0.35620000
	C	3.57100000	-1.52150000	-1.12380000
	C	3.43670000	-1.63190000	-2.50860000
	C	2.24400000	-1.23670000	-3.10060000
	C	1.19470000	-0.73860000	-2.32170000
	H	1.25950000	0.41470000	2.59230000
	H	2.44880000	2.37510000	3.43180000
	H	2.82070000	4.35110000	1.96920000
	H	1.99300000	4.29230000	-0.37840000
	H	0.82250000	2.32420000	-1.22640000
	H	0.49430000	-2.78690000	-0.10160000
	H	-0.26990000	-4.49410000	1.46790000
	H	-1.42060000	-3.84360000	3.57690000
	H	-1.80560000	-1.43010000	4.04930000
	H	-1.08130000	0.27540000	2.45590000
	H	2.65890000	-0.93830000	0.71550000
	H	4.49530000	-1.81880000	-0.64060000
	H	4.25100000	-2.01650000	-3.11220000
	H	2.11730000	-1.31200000	-4.17510000
	H	0.28780000	-0.44660000	-2.83280000
	H	-1.39270000	2.36850000	0.03560000
	H	-3.15520000	0.32370000	0.80970000
**5** (conf. 22)	C	1.72070000	-1.54880000	-0.95830000
	C	2.62550000	-2.71810000	-0.52970000
	C	3.64260000	-2.30060000	0.54280000
	C	2.96700000	-1.58370000	1.72160000
	C	2.09740000	-0.41270000	1.24490000
	C	1.00580000	-0.95610000	0.27270000
	O	3.00580000	0.51620000	0.64130000
	H	0.99650000	-1.89830000	-1.69700000
	H	2.33250000	-0.77970000	-1.43510000
	H	3.14780000	-3.12100000	-1.40360000
	H	1.99720000	-3.53200000	-0.14240000
	H	4.37900000	-1.62420000	0.09930000
	H	4.19190000	-3.17580000	0.90510000
	H	3.72040000	-1.20010000	2.41560000
	H	2.33930000	-2.28680000	2.28280000
	C	-0.21560000	0.01140000	-0.02690000
	H	0.56910000	-1.80890000	0.79930000
	C	-1.07000000	0.27640000	1.25210000
	C	-2.10030000	1.22960000	1.19750000
	C	-2.94400000	1.45910000	2.27800000
	C	-2.79070000	0.73030000	3.45740000
	C	-1.78760000	-0.22850000	3.52820000
	C	-0.94150000	-0.45270000	2.43910000
	C	0.33710000	1.33150000	-0.61510000
	C	0.77160000	1.39850000	-1.94860000
	C	1.35760000	2.55080000	-2.46580000
	C	1.53530000	3.67520000	-1.66190000

	C	1.11960000	3.62810000	-0.33480000
	C	0.52750000	2.47250000	0.17920000
	C	-1.23260000	-0.68320000	-0.98490000
	C	-1.95950000	0.02930000	-1.94700000
	C	-2.93870000	-0.58780000	-2.72540000
	C	-3.22230000	-1.93970000	-2.56080000
	C	-2.52140000	-2.66190000	-1.59740000
	C	-1.54880000	-2.03970000	-0.81980000
	H	-2.25040000	1.80140000	0.29020000
	H	-3.72740000	2.20460000	2.19590000
	H	-3.44740000	0.90470000	4.30220000
	H	-1.65420000	-0.81240000	4.43230000
	H	-0.18300000	-1.21530000	2.54270000
	H	0.64930000	0.54110000	-2.59680000
	H	1.68000000	2.56630000	-3.50110000
	H	1.99240000	4.57220000	-2.06400000
	H	1.25170000	4.49000000	0.30990000
	H	0.21130000	2.46620000	1.21420000
	H	-1.76820000	1.08330000	-2.09960000
	H	-3.47970000	-0.00310000	-3.46150000
	H	-3.98110000	-2.42180000	-3.16670000
	H	-2.73420000	-3.71420000	-1.44360000
	H	-1.04210000	-2.63000000	-0.06700000
	H	1.62760000	0.06370000	2.11140000
	H	2.52870000	1.32430000	0.42420000
**6** (conf. 1)	C	-2.54700000	-1.51270000	-2.01660000
	C	-2.08080000	-2.95590000	-2.22340000
	C	-0.55990000	-3.00710000	-2.38350000
	C	0.12950000	-2.31030000	-1.20480000
	C	-0.28340000	-0.82090000	-1.06310000
	C	-1.81480000	-0.80980000	-0.86440000
	C	0.60180000	-0.06290000	0.02300000
	C	0.25810000	1.45560000	0.12530000
	C	0.22670000	2.13910000	1.34500000
	C	0.04600000	3.52280000	1.39940000
	C	-0.09520000	4.26200000	0.23140000
	C	-0.04450000	3.59910000	-0.99480000
	C	0.13470000	2.22240000	-1.04260000
	C	0.43240000	-0.82140000	1.36310000
	C	-0.72330000	-0.66040000	2.14260000
	C	-0.93400000	-1.40740000	3.29930000
	C	0.00720000	-2.34580000	3.71240000
	C	1.15470000	-2.53040000	2.94780000
	C	1.36010000	-1.78140000	1.79040000
	C	2.10020000	-0.01120000	-0.41720000
	C	2.53680000	-0.25580000	-1.72380000
	C	3.87540000	-0.09580000	-2.08650000
	C	4.81530000	0.31800000	-1.15120000
	C	4.39760000	0.58170000	0.15230000
	C	3.06310000	0.42510000	0.50670000
	H	-2.37840000	-0.93120000	-2.93100000

	H	-3.61940000	-1.48020000	-1.80640000
	H	-2.37410000	-3.56350000	-1.35870000
	H	-2.58300000	-3.38840000	-3.09430000
	H	-0.21610000	-4.04420000	-2.44940000
	H	-0.27190000	-2.52200000	-3.32540000
	H	-0.13280000	-2.83960000	-0.28360000
	H	1.21190000	-2.39420000	-1.30250000
	H	-0.10320000	-0.32980000	-2.02400000
	H	-2.08390000	-1.28470000	0.07920000
	O	-2.30360000	0.55790000	-0.83330000
	H	0.35100000	1.59890000	2.27340000
	H	0.02510000	4.01770000	2.36440000
	H	-0.23040000	5.33700000	0.27130000
	H	-0.13820000	4.15760000	-1.91990000
	H	0.18610000	1.74430000	-2.01220000
	H	-1.47770000	0.05860000	1.85650000
	H	-1.84170000	-1.25280000	3.87160000
	H	-0.15350000	-2.92660000	4.61370000
	H	1.89760000	-3.26190000	3.24660000
	H	2.25920000	-1.95370000	1.21440000
	H	1.84260000	-0.57260000	-2.49010000
	H	4.17600000	-0.29760000	-3.10890000
	H	5.85500000	0.44150000	-1.43220000
	H	5.11150000	0.91860000	0.89590000
	H	2.76150000	0.65450000	1.52110000
	C	-3.37910000	0.83520000	-0.06230000
	C	-3.77640000	2.28240000	-0.20580000
	O	-3.93130000	0.02750000	0.64450000
	H	-4.05170000	2.48810000	-1.24290000
	H	-2.92980000	2.92610000	0.04120000
	H	-4.61880000	2.49200000	0.45020000
**7** (conf. 1)	C	-3.18900000	-0.36350000	-0.11460000
	O	-3.62530000	-1.31860000	0.47270000
	O	-2.02870000	-0.26550000	-0.75480000
	C	-1.16430000	-1.44800000	-0.80530000
	C	-1.67420000	-2.33330000	-1.94780000
	C	0.30240000	-1.01620000	-1.01540000
	C	1.11650000	-2.33420000	-1.10930000
	C	0.65810000	-3.22080000	-2.27310000
	C	-0.81900000	-3.59040000	-2.12830000
	H	-1.27300000	-1.96490000	0.14930000
	H	-2.71390000	-2.60310000	-1.74690000
	H	-1.66940000	-1.74080000	-2.87060000
	H	0.35370000	-0.54910000	-2.00200000
	C	0.89950000	0.02190000	0.03590000
	H	2.17850000	-2.11030000	-1.21190000
	H	1.00730000	-2.89750000	-0.17730000
	H	0.81770000	-2.69690000	-3.22480000
	H	1.27670000	-4.12370000	-2.31210000
	H	-1.17110000	-4.15470000	-2.99780000
	H	-0.94720000	-4.24900000	-1.26030000

	C	2.42050000	0.24880000	-0.23440000
	C	2.91240000	0.32850000	-1.54550000
	C	4.24390000	0.63960000	-1.80860000
	C	5.12750000	0.89610000	-0.76270000
	C	4.65360000	0.84490000	0.54470000
	C	3.32010000	0.52910000	0.80200000
	C	0.62400000	-0.55170000	1.44730000
	C	1.44360000	-1.54920000	1.99880000
	C	1.14630000	-2.14350000	3.22360000
	C	0.01110000	-1.76160000	3.93470000
	C	-0.81940000	-0.77990000	3.40230000
	C	-0.51480000	-0.18600000	2.17820000
	C	0.33830000	1.46860000	-0.13340000
	C	-0.21630000	1.94240000	-1.32730000
	C	-0.58930000	3.27800000	-1.47770000
	C	-0.41140000	4.18340000	-0.43640000
	C	0.15170000	3.73420000	0.75660000
	C	0.52490000	2.40110000	0.89840000
	H	2.25290000	0.16000000	-2.38870000
	H	4.58740000	0.68780000	-2.83750000
	H	6.16520000	1.14130000	-0.96500000
	H	5.32040000	1.05520000	1.37540000
	H	2.98490000	0.50830000	1.83130000
	H	2.33790000	-1.86350000	1.47570000
	H	1.80800000	-2.90720000	3.62070000
	H	-0.22280000	-2.22350000	4.88850000
	H	-1.71410000	-0.47230000	3.93390000
	H	-1.17750000	0.58020000	1.79730000
	H	-0.38700000	1.27730000	-2.16280000
	H	-1.02600000	3.60430000	-2.41650000
	H	-0.70140000	5.22280000	-0.55340000
	H	0.30920000	4.42340000	1.58060000
	H	0.97320000	2.08570000	1.83300000
	C	-3.98040000	0.94190000	-0.26000000
	O	-4.44800000	1.32910000	-1.29610000
	O	-4.12430000	1.55180000	0.92720000
	H	-4.65620000	2.35400000	0.79390000
**7** (conf. 13)	C	-3.18630000	-0.35810000	-0.09780000
	O	-3.64930000	-1.33940000	0.42010000
	O	-2.02370000	-0.24960000	-0.73590000
	C	-1.17360000	-1.44140000	-0.81660000
	C	-1.68340000	-2.30040000	-1.97850000
	C	0.30160000	-1.03010000	-1.00810000
	C	1.09150000	-2.36400000	-1.08310000
	C	0.63920000	-3.23690000	-2.26030000
	C	-0.84940000	-3.57310000	-2.15430000
	H	-1.29270000	-1.97710000	0.12650000
	H	-2.73120000	-2.55240000	-1.79940000
	H	-1.64890000	-1.69870000	-2.89500000
	H	0.37870000	-0.57350000	-1.99790000
	C	0.89230000	0.01200000	0.04170000

	H	2.16010000	-2.16340000	-1.16250000
	H	0.94900000	-2.92690000	-0.15540000
	H	0.83520000	-2.71370000	-3.20550000
	H	1.23670000	-4.15430000	-2.28800000
	H	-1.19270000	-4.12090000	-3.03770000
	H	-1.01330000	-4.23730000	-1.29670000
	C	0.39310000	1.47260000	-0.19400000
	C	-0.15990000	1.91830000	-1.39880000
	C	-0.46960000	3.26350000	-1.60450000
	C	-0.23390000	4.20420000	-0.60770000
	C	0.32380000	3.78120000	0.59760000
	C	0.63730000	2.44020000	0.79290000
	C	2.43020000	0.17330000	-0.17140000
	C	3.31330000	0.37990000	0.89520000
	C	4.66830000	0.63320000	0.68050000
	C	5.17950000	0.69250000	-0.61200000
	C	4.31270000	0.50900000	-1.68760000
	C	2.96080000	0.26190000	-1.46730000
	C	0.53380000	-0.50600000	1.45650000
	C	-0.58610000	-0.02970000	2.15160000
	C	-0.96720000	-0.56920000	3.37970000
	C	-0.23450000	-1.60500000	3.95140000
	C	0.88180000	-2.09490000	3.27720000
	C	1.25640000	-1.55430000	2.04870000
	H	-0.37780000	1.22600000	-2.20060000
	H	-0.90020000	3.56960000	-2.55330000
	H	-0.47610000	5.25010000	-0.76660000
	H	0.52230000	4.49710000	1.38910000
	H	1.08470000	2.14440000	1.73450000
	H	2.94910000	0.35020000	1.91420000
	H	5.32200000	0.78810000	1.53340000
	H	6.23350000	0.88850000	-0.78070000
	H	4.68640000	0.56640000	-2.70540000
	H	2.31390000	0.15260000	-2.33010000
	H	-1.17380000	0.78070000	1.74090000
	H	-1.84470000	-0.17530000	3.88200000
	H	-0.52830000	-2.02530000	4.90790000
	H	1.46880000	-2.90100000	3.70680000
	H	2.13640000	-1.94880000	1.55670000
	C	-3.89210000	1.00180000	-0.04090000
	O	-4.01540000	1.63970000	0.96830000
	O	-4.37950000	1.35690000	-1.24160000
	H	-4.83230000	2.21140000	-1.14460000
**7** (conf. 24)	C	-3.08260000	-0.43040000	0.00880000
	O	-3.22460000	-1.18770000	0.94910000
	O	-2.10110000	-0.41020000	-0.85910000
	C	-1.09640000	-1.48610000	-0.85060000
	C	-1.54240000	-2.42210000	-1.98310000
	C	0.31300000	-0.90130000	-1.08290000
	C	1.26060000	-2.11020000	-1.31170000
	C	0.84320000	-2.99730000	-2.48710000

	C	-0.55740000	-3.56120000	-2.24960000
	H	-1.15880000	-1.99470000	0.11010000
	H	-2.53320000	-2.81530000	-1.73460000
	H	-1.66250000	-1.81810000	-2.88970000
	H	0.24590000	-0.34220000	-2.02040000
	C	0.89910000	0.09020000	0.02130000
	H	2.28230000	-1.75640000	-1.45120000
	H	1.28150000	-2.72330000	-0.40540000
	H	0.85920000	-2.42260000	-3.42260000
	H	1.56860000	-3.80820000	-2.61000000
	H	-0.90050000	-4.14930000	-3.10670000
	H	-0.53530000	-4.24540000	-1.39230000
	C	2.21020000	0.76930000	-0.48920000
	C	2.96300000	1.53790000	0.41370000
	C	4.09250000	2.24080000	0.01170000
	C	4.50490000	2.20820000	-1.32050000
	C	3.76480000	1.46790000	-2.23420000
	C	2.63240000	0.76030000	-1.82290000
	C	1.16480000	-0.74660000	1.29870000
	C	2.43720000	-1.25560000	1.59520000
	C	2.65480000	-2.08590000	2.69400000
	C	1.59970000	-2.43870000	3.53090000
	C	0.32530000	-1.95370000	3.24910000
	C	0.11400000	-1.12250000	2.15020000
	C	-0.02130000	1.32320000	0.27570000
	C	-0.52540000	2.04900000	-0.81390000
	C	-1.26140000	3.21410000	-0.63570000
	C	-1.50090000	3.70660000	0.64660000
	C	-0.98430000	3.01890000	1.73890000
	C	-0.25010000	1.84510000	1.55410000
	H	2.65480000	1.59530000	1.45120000
	H	4.64750000	2.82310000	0.74080000
	H	5.38390000	2.75890000	-1.63940000
	H	4.06100000	1.43540000	-3.27820000
	H	2.08740000	0.20720000	-2.57690000
	H	3.27880000	-1.00720000	0.96110000
	H	3.65580000	-2.45670000	2.89140000
	H	1.76730000	-3.08190000	4.38890000
	H	-0.51410000	-2.21770000	3.88470000
	H	-0.88970000	-0.76030000	1.97270000
	H	-0.35140000	1.70430000	-1.82600000
	H	-1.65670000	3.73340000	-1.50230000
	H	-2.07900000	4.61400000	0.78800000
	H	-1.14590000	3.39160000	2.74590000
	H	0.15010000	1.34690000	2.42730000
	C	-4.14910000	0.66080000	-0.27560000
	O	-4.17280000	1.35280000	-1.24890000
	O	-5.06450000	0.70510000	0.70130000
	H	-4.84180000	0.02250000	1.36020000
**7** (conf. 37)	C	-3.27590000	-0.30160000	-0.08280000
	O	-3.69030000	-1.31720000	0.39800000

O	-2.09030000	-0.13940000	-0.69990000
C	-1.24590000	-1.32980000	-0.86620000
C	-1.78640000	-2.10850000	-2.07270000
C	0.23250000	-0.93420000	-1.06260000
C	0.99610000	-2.27410000	-1.24510000
C	0.50970000	-3.06510000	-2.46410000
C	-0.98130000	-3.38240000	-2.34290000
H	-1.36230000	-1.92120000	0.04260000
H	-2.83550000	-2.35400000	-1.89320000
H	-1.75410000	-1.45090000	-2.95020000
H	0.28420000	-0.40010000	-2.01490000
C	0.89630000	-0.00540000	0.04410000
H	2.06670000	-2.08740000	-1.32600000
H	0.86320000	-2.88760000	-0.34850000
H	0.69310000	-2.48810000	-3.38030000
H	1.09130000	-3.98780000	-2.56010000
H	-1.35610000	-3.86930000	-3.24860000
H	-1.13790000	-4.09350000	-1.52250000
C	0.28060000	1.42740000	0.08060000
C	-0.04740000	2.09920000	-1.10830000
C	-0.44940000	3.43360000	-1.10660000
C	-0.53230000	4.14470000	0.08970000
C	-0.19620000	3.50130000	1.27560000
C	0.20950000	2.16620000	1.26790000
C	2.38130000	0.31860000	-0.31750000
C	2.86780000	0.29220000	-1.63040000
C	4.16620000	0.70190000	-1.93540000
C	5.01550000	1.15810000	-0.93280000
C	4.54450000	1.20750000	0.37790000
C	3.24790000	0.79940000	0.67530000
C	0.78060000	-0.75640000	1.39260000
C	1.78650000	-1.62860000	1.83310000
C	1.63710000	-2.38600000	2.99370000
C	0.47010000	-2.29800000	3.74830000
C	-0.54680000	-1.44780000	3.32230000
C	-0.39020000	-0.69250000	2.16160000
H	0.02420000	1.59320000	-2.06330000
H	-0.69650000	3.91730000	-2.04650000
H	-0.85230000	5.18100000	0.09330000
H	-0.24550000	4.03600000	2.21890000
H	0.47940000	1.70530000	2.20940000
H	2.24210000	-0.04540000	-2.44710000
H	4.50840000	0.66310000	-2.96500000
H	6.02560000	1.47690000	-1.16840000
H	5.18580000	1.57070000	1.17490000
H	2.90650000	0.86220000	1.70170000
H	2.70530000	-1.72190000	1.26790000
H	2.43960000	-3.04750000	3.30520000
H	0.35310000	-2.88590000	4.65290000
H	-1.46930000	-1.36960000	3.88830000
H	-1.19690000	-0.03680000	1.86050000
C	-4.08030000	1.02880000	-0.06580000

	O	-5.21650000	1.05280000	0.30340000
	O	-3.42770000	2.11750000	-0.49250000
	H	-2.50400000	1.92070000	-0.73020000
**8** (conf. 5)	C	-2.81000000	-3.91450000	1.80470000
	C	-4.06500000	-3.04090000	1.76900000
	C	-3.95380000	-1.97300000	0.67380000
	C	-2.72920000	-1.04020000	0.87650000
	C	-1.49170000	-1.96210000	0.84600000
	C	-1.55560000	-3.04540000	1.93090000
	C	-2.71690000	0.20360000	-0.12110000
	C	-4.09760000	0.93210000	-0.08570000
	C	-4.76380000	1.13030000	1.13300000
	C	-5.94220000	1.86710000	1.20950000
	C	-6.48720000	2.44520000	0.06480000
	C	-5.82820000	2.28110000	-1.14930000
	C	-4.65000000	1.53810000	-1.22080000
	C	-2.35960000	-0.33980000	-1.52600000
	C	-1.04040000	-0.32810000	-1.99920000
	C	-0.69680000	-0.90820000	-3.21920000
	C	-1.66790000	-1.51920000	-4.00680000
	C	-2.98450000	-1.54770000	-3.55360000
	C	-3.32180000	-0.96960000	-2.33130000
	C	-1.75520000	1.35020000	0.32390000
	C	-1.45910000	2.37820000	-0.58490000
	C	-0.70950000	3.48940000	-0.21340000
	C	-0.23890000	3.61430000	1.09270000
	C	-0.53120000	2.61160000	2.01090000
	C	-1.28080000	1.49780000	1.63090000
	O	-0.27790000	-1.18010000	1.08640000
	H	-2.84990000	-4.62750000	2.63380000
	H	-2.75250000	-4.50790000	0.88400000
	H	-4.95450000	-3.65410000	1.59290000
	H	-4.20650000	-2.55790000	2.74440000
	H	-3.86960000	-2.47620000	-0.29430000
	H	-4.86880000	-1.38180000	0.64060000
	H	-2.80340000	-0.64320000	1.89190000
	H	-1.39000000	-2.42280000	-0.13810000
	H	-0.65580000	-3.66080000	1.86440000
	H	-1.54030000	-2.55100000	2.90940000
	H	-4.35850000	0.71890000	2.04900000
	H	-6.43030000	1.99460000	2.16960000
	H	-7.40360000	3.02150000	0.12180000
	H	-6.22600000	2.73450000	-2.05080000
	H	-4.15960000	1.43980000	-2.17990000
	H	-0.26380000	0.14260000	-1.41180000
	H	0.33660000	-0.88450000	-3.54650000
	H	-1.40320000	-1.96880000	-4.95720000
	H	-3.75680000	-2.01960000	-4.15130000
	H	-4.35480000	-1.00290000	-2.01160000
	H	-1.82430000	2.31500000	-1.60220000
	H	-0.50050000	4.26180000	-0.94580000

	H	0.34160000	4.48090000	1.38890000
	H	-0.17190000	2.68400000	3.03150000
	H	-1.46560000	0.73950000	2.37740000
	C	0.87870000	-1.68170000	0.65750000
	O	1.03730000	-2.69690000	0.02890000
	C	2.05160000	-0.80890000	1.13470000
	O	2.23210000	-0.53900000	2.29490000
	O	2.83100000	-0.48380000	0.10580000
	C	4.06940000	0.25860000	0.37380000
	C	5.14930000	-0.29410000	-0.56450000
	C	6.42440000	0.55740000	-0.39430000
	C	6.16530000	2.05230000	-0.62150000
	C	5.06720000	2.59220000	0.30680000
	C	3.79310000	1.74500000	0.15350000
	H	4.33140000	0.08060000	1.41820000
	H	4.77950000	-0.13550000	-1.58740000
	C	5.37420000	-1.82320000	-0.40990000
	H	7.19380000	0.20630000	-1.08710000
	H	6.82950000	0.41730000	0.61490000
	H	5.86480000	2.21940000	-1.66470000
	H	7.09140000	2.61790000	-0.47040000
	C	4.78330000	4.07730000	0.06160000
	H	5.42170000	2.48260000	1.34180000
	H	3.38470000	1.87790000	-0.85610000
	H	3.02470000	2.07910000	0.85690000
	C	5.98020000	-2.23920000	0.94030000
	C	6.20680000	-2.38810000	-1.57130000
	H	4.38270000	-2.28200000	-0.48040000
	H	5.68650000	4.67890000	0.20040000
	H	4.42520000	4.24400000	-0.95990000
	H	4.02020000	4.45540000	0.74810000
	H	5.39540000	-1.87740000	1.79030000
	H	6.01440000	-3.32990000	1.01440000
	H	7.00510000	-1.87390000	1.05730000
	H	6.24550000	-3.47980000	-1.51830000
	H	5.77410000	-2.11880000	-2.53970000
	H	7.23910000	-2.02530000	-1.55110000
**8** (conf. 6)	C	-2.75930000	-3.83790000	1.91760000
	C	-4.00740000	-2.95430000	1.88230000
	C	-3.90550000	-1.91490000	0.75910000
	C	-2.67030000	-0.98830000	0.91820000
	C	-1.44070000	-1.92070000	0.88980000
	C	-1.49550000	-2.97750000	2.00140000
	C	-2.66590000	0.23070000	-0.11010000
	C	-4.04040000	0.97070000	-0.06810000
	C	-4.68130000	1.20610000	1.15740000
	C	-5.85270000	1.95380000	1.23680000
	C	-6.41500000	2.50610000	0.08780000
	C	-5.78050000	2.30550000	-1.13380000
	C	-4.60950000	1.55140000	-1.20800000
	C	-2.33770000	-0.35040000	-1.50710000

C	-3.31820000	-0.99410000	-2.27870000
C	-3.00660000	-1.60520000	-3.49180000
C	-1.69820000	-1.59720000	-3.96880000
C	-0.70930000	-0.97260000	-3.21480000
C	-1.02730000	-0.35950000	-2.00400000
C	-1.68800000	1.37990000	0.28950000
C	-1.18800000	1.55400000	1.58340000
C	-0.42570000	2.67190000	1.92420000
C	-0.14510000	3.65160000	0.97810000
C	-0.64010000	3.49960000	-0.31620000
C	-1.40260000	2.38480000	-0.64840000
O	-0.21690000	-1.14340000	1.09190000
H	-2.79070000	-4.53030000	2.76430000
H	-2.72290000	-4.45380000	1.01080000
H	-4.90500000	-3.56370000	1.73680000
H	-4.12760000	-2.44610000	2.84770000
H	-3.84170000	-2.44330000	-0.19710000
H	-4.81590000	-1.31690000	0.72590000
H	-2.72400000	-0.56580000	1.92470000
H	-1.35860000	-2.40550000	-0.08450000
H	-0.60240000	-3.60260000	1.93440000
H	-1.45910000	-2.46000000	2.96730000
H	-4.26130000	0.81550000	2.07590000
H	-6.32130000	2.11010000	2.20220000
H	-7.32590000	3.09090000	0.14690000
H	-6.19190000	2.73860000	-2.03910000
H	-4.13780000	1.42450000	-2.17300000
H	-4.34550000	-1.01210000	-1.94010000
H	-3.79240000	-2.08710000	-4.06330000
H	-1.45330000	-2.07290000	-4.91180000
H	0.31810000	-0.96420000	-3.56100000
H	-0.23760000	0.12110000	-1.44280000
H	-1.36220000	0.81350000	2.34990000
H	-0.04690000	2.76510000	2.93600000
H	0.44560000	4.52130000	1.24330000
H	-0.43990000	4.25360000	-1.06970000
H	-1.78670000	2.30110000	-1.65730000
C	0.92750000	-1.66230000	0.65360000
O	1.06680000	-2.69230000	0.04430000
C	2.11480000	-0.78520000	1.08950000
O	2.27780000	-0.42800000	2.22840000
O	2.92670000	-0.56440000	0.05780000
C	4.16220000	0.19170000	0.30380000
C	5.23780000	-0.31390000	-0.66490000
C	6.49410000	0.56870000	-0.48560000
C	6.21180000	2.06650000	-0.64720000
C	5.11000000	2.54890000	0.30670000
C	3.85590000	1.67930000	0.12650000
H	4.45360000	-0.00230000	1.33770000
H	4.85960000	-0.14100000	-1.68160000
C	5.59900000	-1.82610000	-0.55920000
H	7.24850000	0.25140000	-1.21230000

	H	6.92840000	0.38880000	0.50600000
	H	5.90160000	2.27090000	-1.68090000
	H	7.13040000	2.63950000	-0.47920000
	C	4.79250000	4.03550000	0.11740000
	H	5.47150000	2.40900000	1.33560000
	H	3.44420000	1.83450000	-0.87870000
	H	3.08240000	1.97430000	0.84090000
	C	4.65560000	-2.73320000	-1.36590000
	C	5.74630000	-2.33470000	0.88400000
	H	6.58830000	-1.91210000	-1.02760000
	H	5.68330000	4.65130000	0.27270000
	H	4.42420000	4.23130000	-0.89530000
	H	4.02590000	4.37140000	0.82160000
	H	5.03170000	-3.76120000	-1.36980000
	H	3.64440000	-2.74620000	-0.95700000
	H	4.59150000	-2.40230000	-2.40740000
	H	6.41660000	-1.70810000	1.47980000
	H	6.16080000	-3.34670000	0.88440000
	H	4.78260000	-2.38160000	1.39880000
**8** (conf. 11)	C	1.93470000	-1.65020000	-3.96450000
	C	3.18880000	-0.80920000	-3.71590000
	C	3.53250000	-0.76600000	-2.22120000
	C	2.37770000	-0.17360000	-1.36940000
	C	1.17710000	-1.11580000	-1.60050000
	C	0.77820000	-1.17280000	-3.08010000
	C	2.78290000	0.11170000	0.14460000
	C	2.87050000	-1.25550000	0.86550000
	C	3.98870000	-2.08870000	0.70300000
	C	4.03750000	-3.36180000	1.26720000
	C	2.96200000	-3.84360000	2.00970000
	C	1.84160000	-3.03520000	2.17700000
	C	1.79770000	-1.76070000	1.61320000
	C	1.81130000	1.09610000	0.86700000
	C	0.94280000	1.95880000	0.19180000
	C	0.19410000	2.91730000	0.87530000
	C	0.29220000	3.03950000	2.25680000
	C	1.15700000	2.19270000	2.94700000
	C	1.90730000	1.24510000	2.25910000
	C	4.12240000	0.91260000	0.19880000
	C	4.35270000	1.96510000	-0.69990000
	C	5.48790000	2.76520000	-0.61240000
	C	6.42720000	2.54650000	0.39340000
	C	6.20460000	1.52360000	1.30960000
	C	5.06740000	0.72120000	1.21370000
	O	0.01090000	-0.65860000	-0.83790000
	H	1.63680000	-1.60980000	-5.01670000
	H	2.15150000	-2.70180000	-3.74130000
	H	4.03770000	-1.21430000	-4.27600000
	H	3.02900000	0.21140000	-4.08700000
	H	3.74790000	-1.78430000	-1.88290000
	H	4.44280000	-0.18660000	-2.06940000

H	2.12130000	0.78710000	-1.82290000
H	1.41570000	-2.11720000	-1.23870000
H	-0.08270000	-1.83590000	-3.18730000
H	0.46000000	-0.17000000	-3.38980000
H	4.84440000	-1.73870000	0.14100000
H	4.92110000	-3.97480000	1.12540000
H	2.99710000	-4.83400000	2.44960000
H	0.98960000	-3.39180000	2.74490000
H	0.91100000	-1.15980000	1.76550000
H	0.81970000	1.89450000	-0.87980000
H	-0.47130000	3.56730000	0.31770000
H	-0.29200000	3.78270000	2.78760000
H	1.25420000	2.27130000	4.02430000
H	2.58320000	0.61210000	2.82010000
H	3.63270000	2.18220000	-1.47910000
H	5.63300000	3.56660000	-1.32880000
H	7.31160000	3.16930000	0.46600000
H	6.91510000	1.34440000	2.10930000
H	4.92160000	-0.05890000	1.94880000
C	-0.91950000	-1.56640000	-0.53990000
O	-0.91390000	-2.73330000	-0.83730000
C	-2.03100000	-0.95440000	0.31980000
O	-1.94510000	-0.86350000	1.51800000
O	-3.07430000	-0.61800000	-0.43790000
C	-4.29360000	-0.13370000	0.22720000
C	-4.97680000	0.84890000	-0.73230000
C	-6.33890000	1.25500000	-0.13270000
C	-7.22540000	0.04490000	0.18570000
C	-6.52790000	-0.93510000	1.14080000
C	-5.16540000	-1.34480000	0.55820000
H	-3.98240000	0.36700000	1.14620000
H	-5.17530000	0.28740000	-1.65570000
C	-4.07530000	2.05080000	-1.12570000
H	-6.85780000	1.92290000	-0.82560000
H	-6.18050000	1.82750000	0.78850000
H	-7.48120000	-0.48150000	-0.74360000
H	-8.17160000	0.38180000	0.62300000
C	-7.39400000	-2.16110000	1.44600000
H	-6.34460000	-0.40540000	2.08650000
H	-5.32010000	-1.92160000	-0.36200000
H	-4.63110000	-1.99230000	1.25950000
C	-3.82600000	3.04950000	0.01550000
C	-4.61780000	2.77510000	-2.36790000
H	-3.10670000	1.62380000	-1.40550000
H	-8.35220000	-1.86690000	1.88460000
H	-7.60500000	-2.72840000	0.53330000
H	-6.89630000	-2.83470000	2.14950000
H	-3.42610000	2.56810000	0.91170000
H	-3.09770000	3.80240000	-0.29880000
H	-4.73940000	3.58230000	0.29610000
H	-4.75920000	2.08220000	-3.20290000
H	-5.57720000	3.26310000	-2.17070000

	H	-3.92090000	3.55210000	-2.69570000
**8** (conf. 16)	C	-2.06860000	0.90670000	-4.20310000
	C	-3.35600000	0.20270000	-3.76710000
	C	-3.63490000	0.43820000	-2.27650000
	C	-2.47650000	-0.07450000	-1.37950000
	C	-1.24060000	0.74490000	-1.81030000
	C	-0.90090000	0.52080000	-3.28860000
	C	-2.81860000	-0.08740000	0.17640000
	C	-1.88920000	-1.03310000	1.00020000
	C	-1.07170000	-2.01290000	0.43010000
	C	-0.35940000	-2.91600000	1.22090000
	C	-0.44520000	-2.86280000	2.60730000
	C	-1.25810000	-1.89460000	3.19450000
	C	-1.97110000	-1.00320000	2.40100000
	C	-4.21470000	-0.74580000	0.40870000
	C	-5.11510000	-0.28190000	1.37440000
	C	-6.31080000	-0.95240000	1.63610000
	C	-6.63820000	-2.11100000	0.94030000
	C	-5.74430000	-2.60000000	-0.01110000
	C	-4.55100000	-1.93090000	-0.26380000
	C	-2.75000000	1.37480000	0.68100000
	C	-1.61550000	1.87190000	1.33710000
	C	-1.51990000	3.21390000	1.70480000
	C	-2.55700000	4.09900000	1.42670000
	C	-3.69120000	3.62570000	0.77120000
	C	-3.78290000	2.28490000	0.40330000
	O	-0.07130000	0.36990000	-1.01020000
	H	-1.82130000	0.66480000	-5.24130000
	H	-2.21600000	1.99260000	-4.16200000
	H	-4.20460000	0.55780000	-4.36040000
	H	-3.27070000	-0.87480000	-3.95850000
	H	-3.77710000	1.51090000	-2.11260000
	H	-4.56800000	-0.04880000	-1.99520000
	H	-2.29530000	-1.11010000	-1.67750000
	H	-1.41840000	1.80570000	-1.62550000
	H	-0.01230000	1.10420000	-3.53860000
	H	-0.64850000	-0.53730000	-3.42910000
	H	-0.95850000	-2.08520000	-0.64180000
	H	0.26840000	-3.66000000	0.74220000
	H	0.10950000	-3.56290000	3.22180000
	H	-1.34330000	-1.83480000	4.27400000
	H	-2.60760000	-0.27220000	2.88390000
	H	-4.88890000	0.61010000	1.94280000
	H	-6.98390000	-0.56210000	2.39190000
	H	-7.56770000	-2.63150000	1.14120000
	H	-5.97110000	-3.51130000	-0.55400000
	H	-3.86820000	-2.35740000	-0.98880000
	H	-0.78770000	1.21440000	1.56620000
	H	-0.62430000	3.56160000	2.20730000
	H	-2.48320000	5.14190000	1.71420000
	H	-4.51160000	4.29840000	0.54530000

	H	-4.68210000	1.94600000	-0.09370000
	C	0.90850000	1.26720000	-0.89970000
	O	0.95730000	2.35370000	-1.41660000
	C	1.99220000	0.77520000	0.06790000
	O	1.82150000	0.73210000	1.26020000
	O	3.11310000	0.48340000	-0.58890000
	C	4.29760000	0.11620000	0.20100000
	C	5.17320000	-0.78310000	-0.67860000
	C	6.48910000	-1.07200000	0.07400000
	C	7.21590000	0.20950000	0.50230000
	C	6.32420000	1.11110000	1.36780000
	C	5.00760000	1.40050000	0.62900000
	H	3.94160000	-0.42380000	1.08070000
	H	5.42620000	-0.19090000	-1.56930000
	C	4.43790000	-2.05400000	-1.18620000
	H	7.14570000	-1.67480000	-0.55910000
	H	6.28060000	-1.67510000	0.96560000
	H	7.53240000	0.76910000	-0.38820000
	H	8.12980000	-0.04730000	1.04910000
	C	7.03220000	2.40790000	1.77230000
	H	6.08020000	0.55800000	2.28610000
	H	5.21410000	1.99810000	-0.26760000
	H	4.33860000	1.98830000	1.26350000
	C	4.06490000	-3.05140000	-0.07790000
	C	5.22980000	-2.75630000	-2.30020000
	H	3.50440000	-1.70030000	-1.63630000
	H	7.95320000	2.19850000	2.32430000
	H	7.29940000	2.99960000	0.89020000
	H	6.39420000	3.02800000	2.40870000
	H	3.47340000	-3.87120000	-0.49590000
	H	4.95100000	-3.49610000	0.38470000
	H	3.46640000	-2.59230000	0.71350000
	H	4.63670000	-3.56310000	-2.74050000
	H	5.49200000	-2.06060000	-3.10320000
	H	6.15610000	-3.20310000	-1.92640000
**8** (conf. 17)	C	2.02720000	-1.32840000	-4.07190000
	C	3.32030000	-0.59900000	-3.70040000
	C	3.58810000	-0.68700000	-2.19190000
	C	2.43170000	-0.07540000	-1.35620000
	C	1.18580000	-0.91460000	-1.71430000
	C	0.85970000	-0.83570000	-3.21040000
	C	2.76610000	0.07950000	0.19440000
	C	2.66310000	-1.32520000	0.83690000
	C	3.67530000	-2.28150000	0.65490000
	C	3.55240000	-3.57680000	1.15370000
	C	2.40720000	-3.95720000	1.84920000
	C	1.39020000	-3.02570000	2.03410000
	C	1.51670000	-1.72970000	1.53470000
	C	1.85250000	1.11830000	0.91740000
	C	1.04920000	2.04810000	0.25160000
	C	0.35120000	3.03670000	0.94750000

C	0.43660000	3.12090000	2.33230000
C	1.23640000	2.20510000	3.01400000
C	1.93600000	1.22880000	2.31430000
C	4.17430000	0.72860000	0.37250000
C	4.53500000	1.84230000	-0.40210000
C	5.74090000	2.50790000	-0.20640000
C	6.62350000	2.08870000	0.78790000
C	6.27230000	1.00360000	1.58340000
C	5.06390000	0.33580000	1.37880000
O	0.01590000	-0.44170000	-0.96710000
H	1.78930000	-1.19150000	-5.13120000
H	2.16050000	-2.40610000	-3.91820000
H	4.16820000	-1.02190000	-4.24870000
H	3.24960000	0.45460000	-4.00010000
H	3.71860000	-1.73930000	-1.92110000
H	4.52510000	-0.18380000	-1.95560000
H	2.26730000	0.93000000	-1.75130000
H	1.34360000	-1.95380000	-1.42070000
H	-0.03620000	-1.42790000	-3.40800000
H	0.62530000	0.20660000	-3.45850000
H	4.58390000	-2.01470000	0.13180000
H	4.35780000	-4.28680000	0.99860000
H	2.30930000	-4.96450000	2.23840000
H	0.48580000	-3.30120000	2.56500000
H	0.70320000	-1.03480000	1.69400000
H	0.93630000	2.01550000	-0.82220000
H	-0.26440000	3.73900000	0.39600000
H	-0.10780000	3.88690000	2.87310000
H	1.32230000	2.25330000	4.09410000
H	2.56360000	0.54190000	2.86840000
H	3.86210000	2.21520000	-1.16500000
H	5.98610000	3.36230000	-0.82790000
H	7.56270000	2.60710000	0.94450000
H	6.93630000	0.66960000	2.37340000
H	4.81910000	-0.49650000	2.02470000
C	-0.97960000	-1.30800000	-0.77620000
O	-1.04090000	-2.44120000	-1.17860000
C	-2.06650000	-0.70200000	0.12030000
O	-1.91760000	-0.55970000	1.30790000
O	-3.16400000	-0.43300000	-0.58500000
C	-4.35200000	0.03180000	0.14790000
C	-5.22510000	0.84290000	-0.81610000
C	-6.52850000	1.22150000	-0.07510000
C	-7.26610000	0.01170000	0.50980000
C	-6.36910000	-0.80910000	1.44600000
C	-5.07500000	-1.19260000	0.71140000
H	-3.99540000	0.65810000	0.96830000
H	-5.50230000	0.16920000	-1.63820000
C	-4.56310000	2.09980000	-1.45410000
H	-7.18250000	1.75480000	-0.77220000
H	-6.29870000	1.92830000	0.73230000
H	-7.61370000	-0.63470000	-0.30750000

	H	-8.16190000	0.34500000	1.04500000
	C	-7.08540000	-2.04560000	1.99740000
	H	-6.09630000	-0.16850000	2.29690000
	H	-5.31060000	-1.87120000	-0.11790000
	H	-4.40100000	-1.72610000	1.38670000
	C	-3.72130000	1.77930000	-2.70000000
	C	-3.76440000	2.96010000	-0.46230000
	H	-5.40380000	2.71410000	-1.80220000
	H	-7.99500000	-1.76600000	2.53730000
	H	-7.37350000	-2.72490000	1.18790000
	H	-6.44430000	-2.60230000	2.68700000
	H	-2.82620000	1.20740000	-2.45080000
	H	-4.29690000	1.19700000	-3.42580000
	H	-3.40580000	2.70450000	-3.19260000
	H	-3.46510000	3.89910000	-0.93700000
	H	-2.85090000	2.45790000	-0.13190000
	H	-4.34670000	3.21760000	0.42690000
**9** (conf. 13)	C	2.80320000	1.83920000	-1.22440000
	C	4.02350000	2.76620000	-1.24910000
	C	3.61470000	4.19420000	-1.61940000
	C	2.48040000	4.69620000	-0.71510000
	C	1.22250000	3.79100000	-0.80160000
	C	1.69030000	2.39800000	-0.33060000
	C	-0.05920000	4.39180000	-0.07010000
	C	-0.26460000	5.88070000	-0.49000000
	C	-0.71850000	6.85720000	0.40380000
	C	-0.99210000	8.15820000	-0.01830000
	C	-0.82380000	8.51850000	-1.35020000
	C	-0.39380000	7.55450000	-2.25940000
	C	-0.12800000	6.25700000	-1.83480000
	C	-1.39710000	3.74540000	-0.54810000
	C	-1.52250000	2.98780000	-1.71570000
	C	-2.76680000	2.54060000	-2.16150000
	C	-3.92210000	2.83840000	-1.44860000
	C	-3.81770000	3.59570000	-0.28380000
	C	-2.57680000	4.04600000	0.15010000
	C	0.15420000	4.21760000	1.45350000
	C	1.01510000	5.06670000	2.16730000
	C	1.29180000	4.85540000	3.51610000
	C	0.71850000	3.78170000	4.19200000
	C	-0.13320000	2.92640000	3.50060000
	C	-0.41190000	3.14280000	2.15220000
	H	3.08200000	0.84790000	-0.86240000
	H	2.40370000	1.71700000	-2.23840000
	H	4.76970000	2.37840000	-1.94910000
	H	4.49570000	2.76810000	-0.25930000
	H	3.28880000	4.22400000	-2.66710000
	H	4.47260000	4.86950000	-1.54120000
	H	2.22210000	5.72050000	-0.98190000
	H	2.84160000	4.72130000	0.31760000
	H	0.97870000	3.70740000	-1.86380000

H	2.03760000	2.45370000	0.70250000
O	0.57140000	1.45380000	-0.34490000
H	-0.86850000	6.60840000	1.44560000
H	-1.34120000	8.88830000	0.70380000
H	-1.03370000	9.53000000	-1.67890000
H	-0.27210000	7.80910000	-3.30660000
H	0.17600000	5.52640000	-2.57440000
H	-0.65200000	2.71290000	-2.29290000
H	-2.82430000	1.95320000	-3.07150000
H	-4.88890000	2.49020000	-1.79440000
H	-4.70560000	3.84320000	0.28760000
H	-2.52640000	4.64640000	1.04970000
H	1.46740000	5.91600000	1.67290000
H	1.95730000	5.53540000	4.03690000
H	0.93400000	3.61480000	5.24140000
H	-0.58550000	2.07810000	4.00150000
H	-1.07730000	2.45550000	1.64840000
C	0.65290000	0.39960000	0.46700000
C	-0.65290000	-0.39960000	0.46700000
O	1.57800000	0.10800000	1.18040000
O	-1.57800000	-0.10800000	1.18040000
O	-0.57140000	-1.45380000	-0.34490000
C	-1.69030000	-2.39800000	-0.33060000
C	-1.22250000	-3.79100000	-0.80160000
C	-2.48040000	-4.69620000	-0.71510000
C	-3.61470000	-4.19420000	-1.61940000
C	-4.02350000	-2.76620000	-1.24910000
C	-2.80320000	-1.83920000	-1.22440000
H	-2.03760000	-2.45370000	0.70250000
H	-0.97870000	-3.70740000	-1.86380000
C	0.05920000	-4.39180000	-0.07010000
H	-2.22210000	-5.72050000	-0.98190000
H	-2.84160000	-4.72130000	0.31760000
H	-3.28880000	-4.22400000	-2.66710000
H	-4.47260000	-4.86950000	-1.54120000
H	-4.76970000	-2.37840000	-1.94910000
H	-4.49570000	-2.76810000	-0.25930000
H	-2.40370000	-1.71700000	-2.23840000
H	-3.08200000	-0.84790000	-0.86240000
C	1.39710000	-3.74540000	-0.54810000
C	2.57680000	-4.04600000	0.15010000
C	3.81770000	-3.59570000	-0.28380000
C	3.92210000	-2.83840000	-1.44860000
C	2.76680000	-2.54060000	-2.16150000
C	1.52250000	-2.98780000	-1.71570000
C	-0.15420000	-4.21760000	1.45350000
C	0.41190000	-3.14280000	2.15220000
C	-1.01510000	-5.06670000	2.16730000
C	-1.29180000	-4.85540000	3.51610000
C	-0.71850000	-3.78170000	4.19200000
C	0.13320000	-2.92640000	3.50060000
C	0.26460000	-5.88070000	-0.49000000

	C	0.12800000	-6.25700000	-1.83480000
	C	0.39380000	-7.55450000	-2.25940000
	C	0.82380000	-8.51850000	-1.35020000
	C	0.99210000	-8.15820000	-0.01830000
	C	0.71850000	-6.85720000	0.40380000
	H	2.52640000	-4.64640000	1.04970000
	H	4.70560000	-3.84320000	0.28760000
	H	4.88890000	-2.49020000	-1.79440000
	H	2.82430000	-1.95320000	-3.07150000
	H	0.65200000	-2.71290000	-2.29290000
	H	1.07730000	-2.45550000	1.64840000
	H	-1.46740000	-5.91600000	1.67290000
	H	-1.95730000	-5.53540000	4.03690000
	H	-0.93400000	-3.61480000	5.24140000
	H	0.58550000	-2.07810000	4.00150000
	H	-0.17600000	-5.52640000	-2.57440000
	H	0.27210000	-7.80910000	-3.30660000
	H	1.03370000	-9.53000000	-1.67890000
	H	1.34120000	-8.88830000	0.70380000
	H	0.86850000	-6.60840000	1.44560000
**9** (conf. 26)	C	-1.16370000	3.04020000	3.40860000
	C	-2.07170000	4.22370000	3.75240000
	C	-1.55000000	5.50400000	3.09880000
	C	-1.35160000	5.29930000	1.59220000
	C	-0.34320000	4.16240000	1.27100000
	C	-0.94770000	2.88450000	1.89530000
	C	0.04720000	4.09710000	-0.27530000
	C	-1.15040000	3.46790000	-1.02510000
	C	-1.16640000	2.10300000	-1.34030000
	C	-2.28210000	1.49840000	-1.91550000
	C	-3.41850000	2.24920000	-2.19890000
	C	-3.42560000	3.60760000	-1.89540000
	C	-2.30980000	4.20540000	-1.31340000
	C	0.41410000	5.52690000	-0.78320000
	C	1.26160000	6.34080000	-0.01550000
	C	1.68700000	7.58410000	-0.46970000
	C	1.29040000	8.04940000	-1.72180000
	C	0.47560000	7.24530000	-2.50980000
	C	0.04650000	6.00080000	-2.04730000
	C	1.36980000	3.31930000	-0.55820000
	C	2.27970000	2.93130000	0.42720000
	C	3.50270000	2.34670000	0.09460000
	C	3.85130000	2.14320000	-1.23410000
	C	2.95610000	2.52490000	-2.23330000
	C	1.73890000	3.10420000	-1.89650000
	H	-1.58770000	2.11190000	3.79650000
	H	-0.18220000	3.17420000	3.87860000
	H	-2.13910000	4.33400000	4.83890000
	H	-3.08820000	4.02050000	3.39450000
	H	-0.59810000	5.79420000	3.56150000
	H	-2.24480000	6.33260000	3.26800000

H	-1.01880000	6.23020000	1.13490000
H	-2.32150000	5.05850000	1.14620000
H	0.57170000	4.38730000	1.82590000
H	-1.89030000	2.63570000	1.40500000
O	-0.04720000	1.74890000	1.70750000
H	-0.29340000	1.50040000	-1.12940000
H	-2.26250000	0.43640000	-2.13060000
H	-4.28730000	1.78180000	-2.64860000
H	-4.30160000	4.21020000	-2.11020000
H	-2.34530000	5.26430000	-1.09590000
H	1.61420000	5.99710000	0.94920000
H	2.33820000	8.18650000	0.15440000
H	1.62200000	9.01740000	-2.07980000
H	0.16960000	7.57960000	-3.49520000
H	-0.57540000	5.39610000	-2.69310000
H	2.04350000	3.04330000	1.47390000
H	4.17290000	2.03820000	0.88850000
H	4.80350000	1.69240000	-1.49020000
H	3.20850000	2.38060000	-3.27840000
H	1.06710000	3.40530000	-2.69070000
C	-0.56700000	0.52690000	1.83100000
C	0.56700000	-0.52690000	1.83100000
O	-1.72850000	0.25470000	1.97990000
O	1.72850000	-0.25470000	1.97990000
O	0.04720000	-1.74890000	1.70750000
C	0.94770000	-2.88450000	1.89530000
C	0.34320000	-4.16240000	1.27100000
C	1.35160000	-5.29930000	1.59220000
C	1.55000000	-5.50400000	3.09880000
C	2.07170000	-4.22370000	3.75240000
C	1.16370000	-3.04020000	3.40860000
H	1.89030000	-2.63570000	1.40500000
H	-0.57170000	-4.38730000	1.82590000
C	-0.04720000	-4.09710000	-0.27530000
H	1.01880000	-6.23020000	1.13490000
H	2.32150000	-5.05850000	1.14620000
H	0.59810000	-5.79420000	3.56150000
H	2.24480000	-6.33260000	3.26800000
H	2.13910000	-4.33400000	4.83890000
H	3.08820000	-4.02050000	3.39450000
H	0.18220000	-3.17420000	3.87860000
H	1.58770000	-2.11190000	3.79650000
C	-1.36980000	-3.31930000	-0.55820000
C	-1.73890000	-3.10420000	-1.89650000
C	-2.95610000	-2.52490000	-2.23330000
C	-3.85130000	-2.14320000	-1.23410000
C	-3.50270000	-2.34670000	0.09460000
C	-2.27970000	-2.93130000	0.42720000
C	1.15040000	-3.46790000	-1.02510000
C	1.16640000	-2.10300000	-1.34030000
C	2.30980000	-4.20540000	-1.31340000
C	3.42560000	-3.60760000	-1.89540000

	C	3.41850000	-2.24920000	-2.19890000
	C	2.28210000	-1.49840000	-1.91550000
	C	-0.41410000	-5.52690000	-0.78320000
	C	-1.26160000	-6.34080000	-0.01550000
	C	-1.68700000	-7.58410000	-0.46970000
	C	-1.29040000	-8.04940000	-1.72180000
	C	-0.47560000	-7.24530000	-2.50980000
	C	-0.04650000	-6.00080000	-2.04730000
	H	-1.06710000	-3.40530000	-2.69070000
	H	-3.20850000	-2.38060000	-3.27840000
	H	-4.80350000	-1.69240000	-1.49020000
	H	-4.17290000	-2.03820000	0.88850000
	H	-2.04350000	-3.04330000	1.47390000
	H	0.29340000	-1.50040000	-1.12940000
	H	2.34530000	-5.26430000	-1.09590000
	H	4.30160000	-4.21020000	-2.11020000
	H	4.28730000	-1.78180000	-2.64860000
	H	2.26250000	-0.43640000	-2.13060000
	H	-1.61420000	-5.99710000	0.94920000
	H	-2.33820000	-8.18650000	0.15440000
	H	-1.62200000	-9.01740000	-2.07980000
	H	-0.16960000	-7.57960000	-3.49520000
	H	0.57540000	-5.39610000	-2.69310000
**11** (conf. 1)	C	3.11850000	0.31700000	2.15780000
	C	1.83650000	0.93190000	1.79890000
	C	0.54490000	0.14110000	1.72650000
	C	-0.07020000	-0.02250000	0.29660000
	C	-0.72940000	1.28650000	-0.21160000
	C	-0.77410000	2.46510000	0.53970000
	C	-1.41090000	3.61090000	0.05560000
	C	-2.01990000	3.60380000	-1.19350000
	C	-1.99030000	2.43480000	-1.95460000
	C	-1.35850000	1.29660000	-1.46670000
	C	1.09060000	-0.48330000	-0.61850000
	C	1.67570000	0.34950000	-1.57660000
	C	2.78070000	-0.06980000	-2.32100000
	C	3.32730000	-1.33260000	-2.12040000
	C	2.76130000	-2.17280000	-1.16120000
	C	1.66190000	-1.75040000	-0.42080000
	C	-1.21010000	-1.07630000	0.35770000
	C	-1.42900000	-1.99730000	-0.67440000
	C	-2.49800000	-2.89190000	-0.63230000
	C	-3.37830000	-2.88770000	0.44580000
	C	-3.18090000	-1.97160000	1.47650000
	C	-2.11480000	-1.07670000	1.42850000
	O	2.38040000	1.08120000	3.12300000
	H	4.04940000	0.76800000	1.82380000
	H	3.15790000	-0.75210000	2.34990000
	H	1.87980000	1.84690000	1.21290000
	H	0.72510000	-0.85370000	2.14040000
	H	-0.17570000	0.61960000	2.39300000

	H	-0.31990000	2.51650000	1.51970000
	H	-1.42710000	4.50750000	0.66540000
	H	-2.51520000	4.49190000	-1.56920000
	H	-2.46710000	2.40660000	-2.92820000
	H	-1.36040000	0.39760000	-2.07090000
	H	1.27310000	1.33870000	-1.75110000
	H	3.21080000	0.59900000	-3.05850000
	H	4.18300000	-1.66020000	-2.70020000
	H	3.17590000	-3.16030000	-0.98960000
	H	1.23550000	-2.42140000	0.31610000
	H	-0.75670000	-2.02820000	-1.52240000
	H	-2.63790000	-3.59390000	-1.44720000
	H	-4.20780000	-3.58480000	0.48140000
	H	-3.86020000	-1.94770000	2.32160000
	H	-2.00380000	-0.36580000	2.23760000
**11** (conf. 25)	C	2.87750000	-1.28010000	2.00530000
	C	2.09800000	-0.04580000	1.84420000
	C	0.58910000	-0.01990000	1.73750000
	C	-0.03560000	0.06210000	0.30370000
	C	0.28820000	-1.20730000	-0.52990000
	C	0.21430000	-2.47420000	0.06700000
	C	0.41150000	-3.63990000	-0.66830000
	C	0.67780000	-3.57430000	-2.03440000
	C	0.72890000	-2.32820000	-2.65070000
	C	0.53290000	-1.16340000	-1.90850000
	C	-1.58740000	0.10290000	0.40570000
	C	-2.34680000	0.16200000	-0.77310000
	C	-3.73580000	0.19260000	-0.74150000
	C	-4.41260000	0.15900000	0.47850000
	C	-3.67760000	0.09740000	1.65600000
	C	-2.28040000	0.07100000	1.61950000
	C	0.49370000	1.37480000	-0.33000000
	C	-0.23480000	2.56640000	-0.22510000
	C	0.27570000	3.77430000	-0.70010000
	C	1.53250000	3.82240000	-1.29550000
	C	2.27670000	2.64920000	-1.40020000
	C	1.76550000	1.44580000	-0.91910000
	O	2.60370000	-0.44010000	3.13470000
	H	3.91780000	-1.30560000	1.68950000
	H	2.36970000	-2.23980000	2.01060000
	H	2.61780000	0.81790000	1.44100000
	H	0.20600000	-0.89100000	2.27180000
	H	0.24810000	0.85910000	2.29340000
	H	-0.02340000	-2.56660000	1.11920000
	H	0.34790000	-4.60180000	-0.17130000
	H	0.83230000	-4.48040000	-2.60910000
	H	0.91930000	-2.25450000	-3.71590000
	H	0.56980000	-0.21190000	-2.42190000
	H	-1.84420000	0.18370000	-1.73220000
	H	-4.29180000	0.23950000	-1.67150000
	H	-5.49610000	0.17870000	0.50660000

	H	-4.18460000	0.07040000	2.61440000
	H	-1.74900000	0.02890000	2.56030000
	H	-1.21680000	2.55800000	0.23020000
	H	-0.31690000	4.67750000	-0.60390000
	H	1.92860000	4.75940000	-1.67040000
	H	3.26110000	2.66710000	-1.85520000
	H	2.36920000	0.55150000	-1.00890000
**11** (conf. 49)	C	-2.54580000	0.94140000	3.14130000
	C	-1.84150000	0.86360000	1.85340000
	C	-0.52730000	0.11110000	1.73450000
	C	0.07170000	-0.02090000	0.29160000
	C	0.75610000	1.29170000	-0.16720000
	C	1.52340000	1.29160000	-1.34290000
	C	2.16180000	2.44090000	-1.79520000
	C	2.05870000	3.63240000	-1.07670000
	C	1.31030000	3.65100000	0.09410000
	C	0.66720000	2.49380000	0.54180000
	C	1.18410000	-1.10500000	0.33100000
	C	1.29980000	-2.10920000	-0.63620000
	C	2.34100000	-3.03810000	-0.59330000
	C	3.29330000	-2.98420000	0.41930000
	C	3.20040000	-1.98150000	1.38340000
	C	2.16440000	-1.05370000	1.33360000
	C	-1.08960000	-0.41880000	-0.65150000
	C	-1.78350000	-1.61640000	-0.41780000
	C	-2.86530000	-1.99220000	-1.20580000
	C	-3.29150000	-1.17320000	-2.25110000
	C	-2.62450000	0.02290000	-2.48870000
	C	-1.53630000	0.39580000	-1.69610000
	O	-3.02240000	0.08090000	2.10130000
	H	-3.17930000	1.79640000	3.36570000
	H	-2.12840000	0.43110000	4.00720000
	H	-2.01460000	1.66780000	1.14330000
	H	0.21210000	0.57970000	2.38820000
	H	-0.69640000	-0.89130000	2.13370000
	H	1.62830000	0.37480000	-1.91040000
	H	2.74760000	2.40390000	-2.70720000
	H	2.55950000	4.52900000	-1.42400000
	H	1.22170000	4.56570000	0.67010000
	H	0.09990000	2.55540000	1.46070000
	H	0.57180000	-2.17650000	-1.43410000
	H	2.40150000	-3.80520000	-1.35770000
	H	4.09980000	-3.70780000	0.45520000
	H	3.94020000	-1.91590000	2.17370000
	H	2.13620000	-0.27010000	2.08090000
	H	-1.47750000	-2.26740000	0.39260000
	H	-3.38130000	-2.92290000	-0.99750000
	H	-4.13690000	-1.46300000	-2.86510000
	H	-2.94740000	0.67730000	-3.29110000
	H	-1.04010000	1.33490000	-1.90240000

**11** (conf. 73)	C	-2.63030000	-0.60030000	3.18340000
	C	-2.05050000	-0.26580000	1.87550000
	C	-0.55230000	-0.07940000	1.72810000
	C	0.05240000	0.05210000	0.28000000
	C	-0.85160000	1.03960000	-0.49740000
	C	-1.02070000	2.35040000	-0.02040000
	C	-1.87530000	3.24840000	-0.65190000
	C	-2.59420000	2.85770000	-1.78180000
	C	-2.44570000	1.56160000	-2.26240000
	C	-1.58580000	0.66320000	-1.62570000
	C	1.50040000	0.59790000	0.43920000
	C	2.38200000	-0.04000000	1.32540000
	C	3.69740000	0.38860000	1.47390000
	C	4.17550000	1.46460000	0.72700000
	C	3.32200000	2.09450000	-0.17240000
	C	2.00100000	1.66480000	-0.31430000
	C	0.17450000	-1.30540000	-0.45380000
	C	-0.43400000	-2.47880000	0.00150000
	C	-0.28670000	-3.68150000	-0.69570000
	C	0.46760000	-3.73690000	-1.86170000
	C	1.07900000	-2.57310000	-2.32930000
	C	0.93570000	-1.37890000	-1.63180000
	O	-2.55520000	-1.58980000	2.15010000
	H	-3.63440000	-0.26750000	3.43460000
	H	-1.96960000	-0.77100000	4.03080000
	H	-2.67780000	0.29140000	1.18610000
	H	-0.29610000	0.84500000	2.25410000
	H	-0.05630000	-0.88630000	2.27120000
	H	-0.47060000	2.68130000	0.85310000
	H	-1.97930000	4.25470000	-0.26090000
	H	-3.26100000	3.55520000	-2.27620000
	H	-3.00070000	1.23770000	-3.13610000
	H	-1.49640000	-0.34150000	-2.01620000
	H	2.04960000	-0.89920000	1.89530000
	H	4.35220000	-0.12530000	2.16910000
	H	5.20040000	1.79950000	0.84020000
	H	3.67900000	2.92480000	-0.77200000
	H	1.36200000	2.17160000	-1.02520000
	H	-1.03880000	-2.47880000	0.89710000
	H	-0.77080000	-4.57490000	-0.31650000
	H	0.58220000	-4.67140000	-2.39970000
	H	1.67550000	-2.59640000	-3.23490000
	H	1.43030000	-0.49080000	-2.00670000

Single crystals X-ray analysis

The colourless single crystals of compounds (*rac*)-**3**, (*rac*)-**4**,(*R*)-**4**, (*rac*)-**5**, (*rac*)-**7**, **8**, **9**, **11** and **12** suitable for X-ray structural analysis were obtained by slow evaporation of solvent. The diffraction data were collected at 130K with an Oxford Diffraction SuperNova diffractometer, using Cu Kα radiation (λ = 1.54184 Å). The intensity data were collected and processed using CrysAlisPro software.[10] The structures were solved by direct methods with the program SHELXT 2018/2[11] and refined by full-matrix least-squares method on F^2 with SHELXL 2018/3.[12] The carbon-bound hydrogen atoms were refined as riding on their carriers and their displacement parameters were set equal to 1.5Ueq(C) for the methyl groups and 1.2Ueq(C) for the remaining H atoms. The hydrogen atoms of OH groups were located in electron-density difference maps. In the final cycles of refinement they were included in calculated position and treated as riding atoms, their displacement parameters were set equal to 1.5Ueq(O). Absolute structures of the compounds were specified by the synthetic procedure and confirmed using Flack parameter.[13]

A summary of the crystallographic data is given in **Table S_4.** Molecular graphics were generated with Olex2[14] and Mercury 4.2.0 software.[15] ORTEP representation of the molecular structures of the reported compounds are presented in **Figure S_94**-**S_102**. Selected geometrical data are given in **Table S_5**.

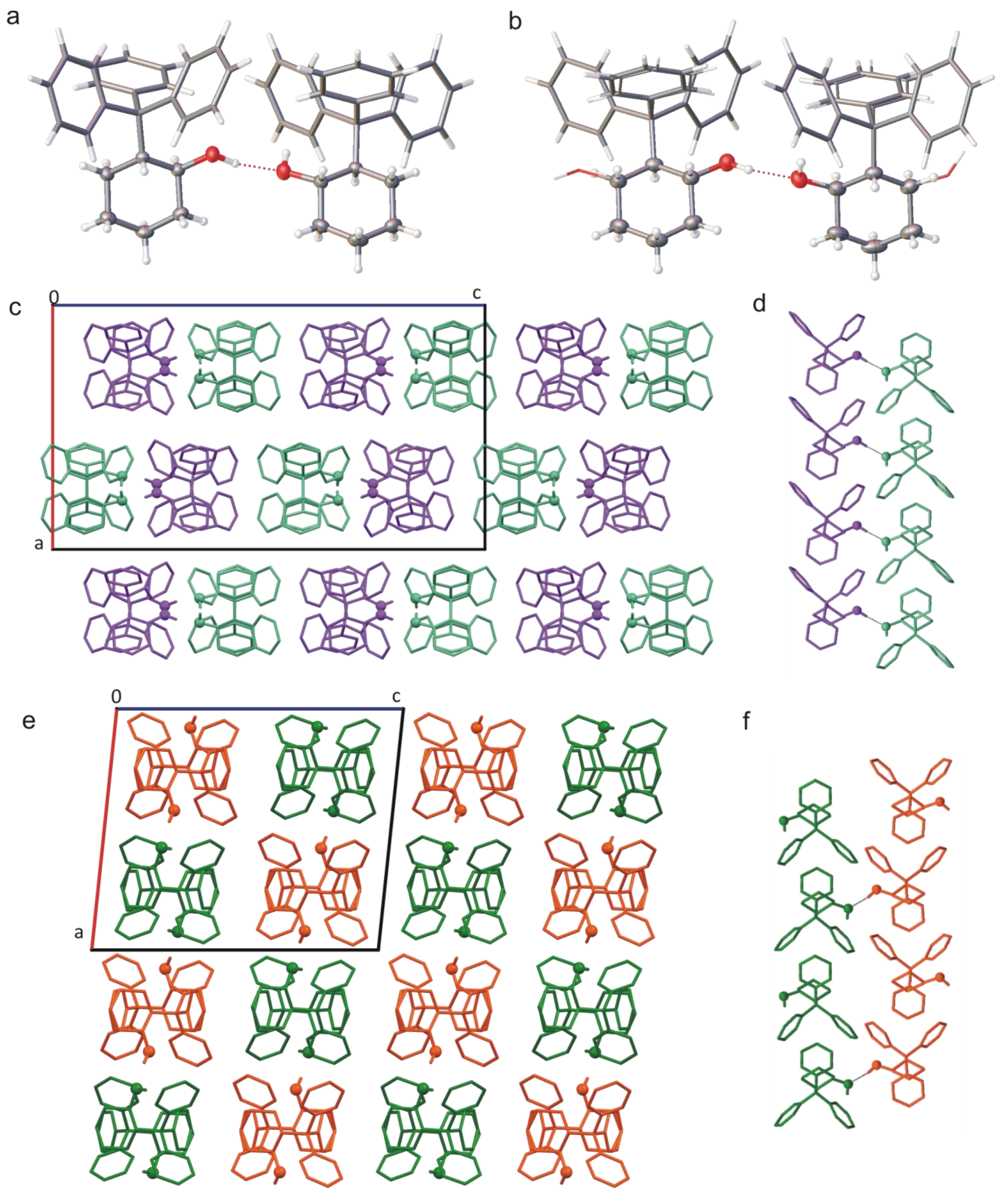

Figure S_94. a) The molecular structure of asymmetric unit of compound *rac*-**3**: a) and polymorph α, b) polymorph *β*. c) Molecular packing (view along b-axis) and d) supramolecular column in crystal structure of *rac*-**3**- α and e) molecular packing (view along b-axis) and d) supramolecular column in crystal structure of *rac*-**3**- *β*. Symmetrically independent molecules are indicated with different colors and the O-atoms are shown as balls.

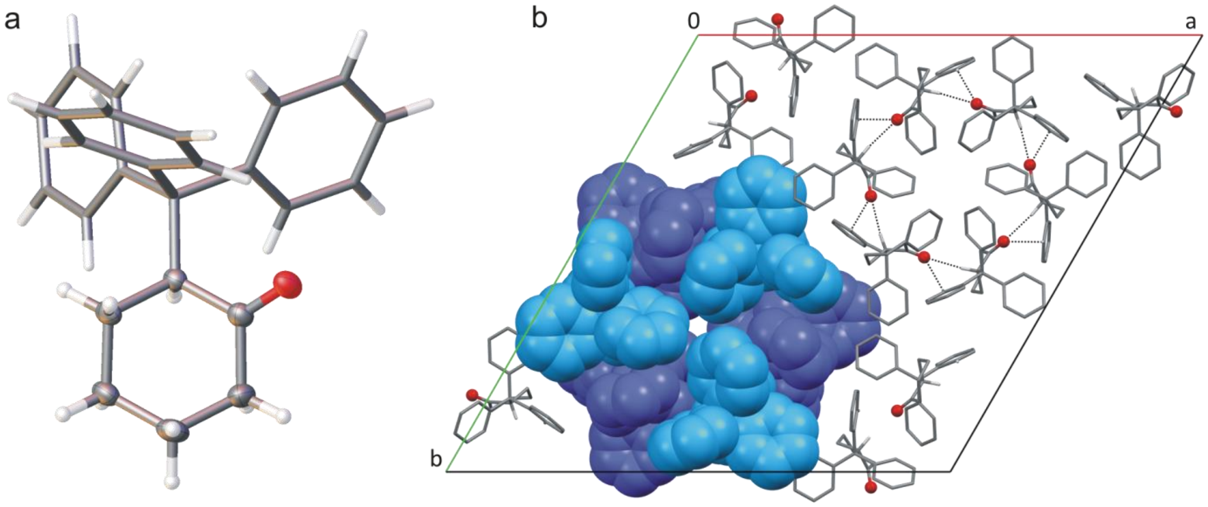

Figure S_95. a) The molecular structure of compound *rac*-**4** and b) molecular packing (view along c-axis). The cyclic motif *via* hydrogen bonds is indicated with blue colored spacefill model. The O-atoms are shown as balls and hydrogen bonds are shown as dashed lines.

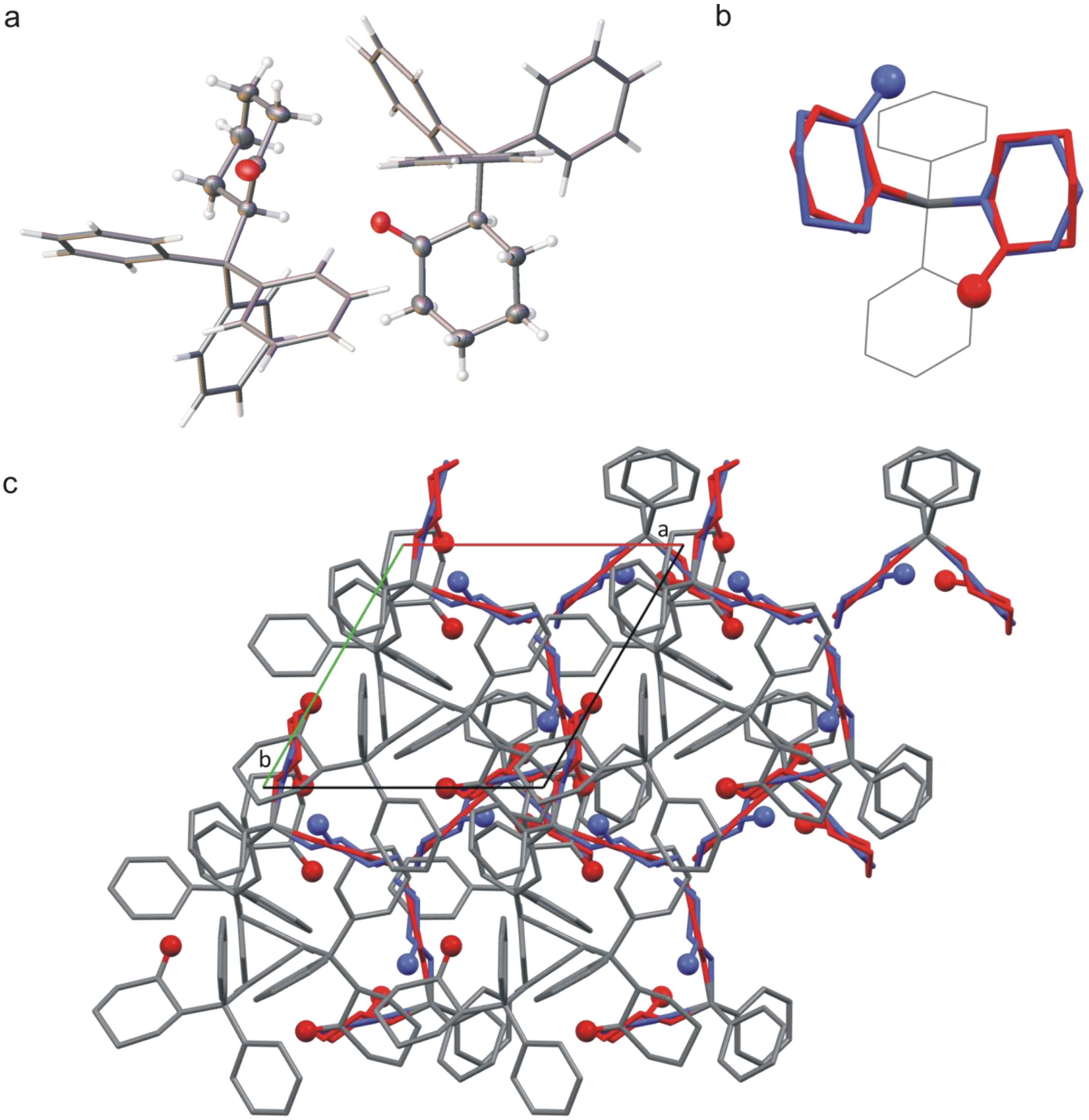

Figure S_96. a) The molecular structure of compound (R)-**4**, b) one molecule is disordered and different orientations of the molecule are represented by different colors (refined occupancy factors for disordered parts are 0.88 and 0.12; O atoms are shown as balls) and c) molecular packing in crystal structure (view along c-axis).

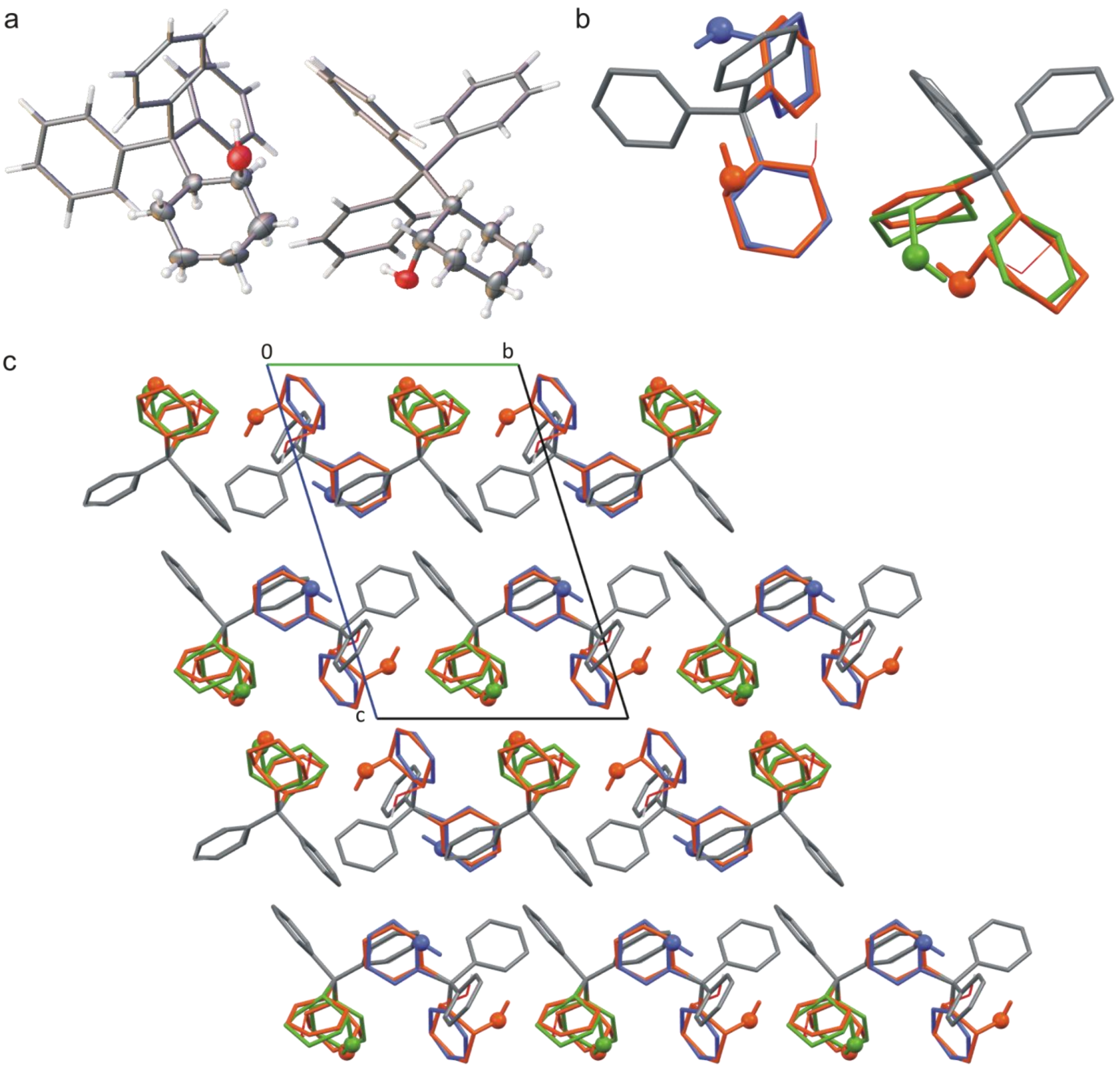

Figure S_97. a) Molecular structure of asymmetric unit of compound *rac*-**5**, b) molecules are disordered and different orientations of the molecule are represented by different colors and O atoms are shown as balls; c) molecular packing in crystal structure (view along a-axis).

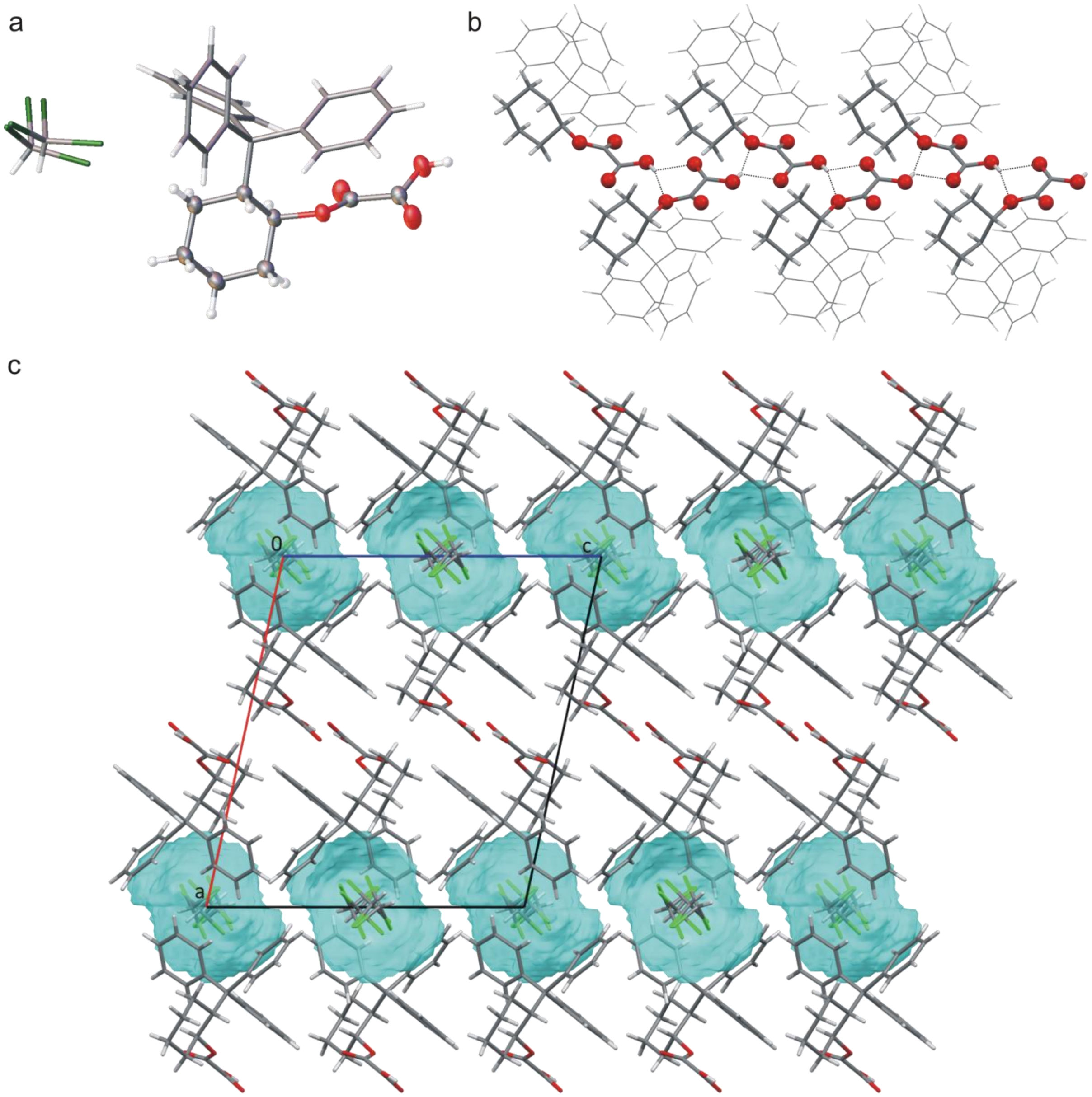

Figure S_98. a) Molecular structure of asymmetric unit of compound *rac*-**7**; b) supramolecular chain *via* hydrogen bonds; c) molecular packing in crystal structure (view along b-axis), the solvent accessible space is indicated with bluish-colour surface. Hydrogen bonds are shown as dashed lines and the O atoms are shown as balls.

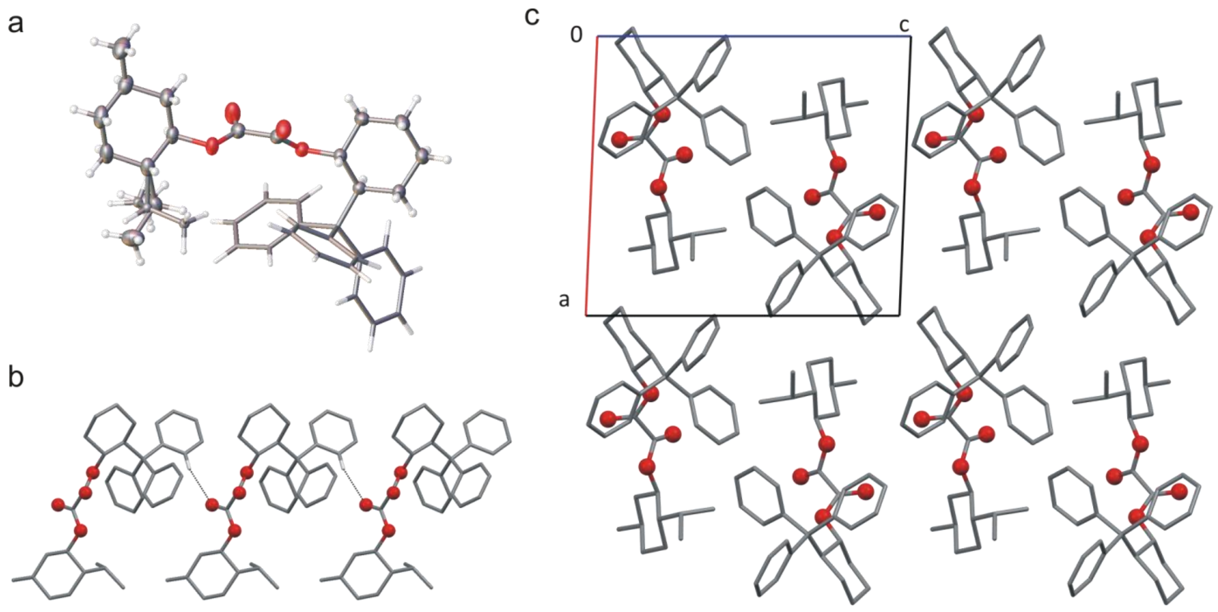

Figure S_99. a) Molecular structure of compound **8**; b) supramolecular chain *via* C-H···O interactions; c) molecular packing in crystal structure (view along b-axis), hydrogen bonds are shown as dashed lines and the O atoms are shown as balls and hydrogen atoms are omitted for clarity.

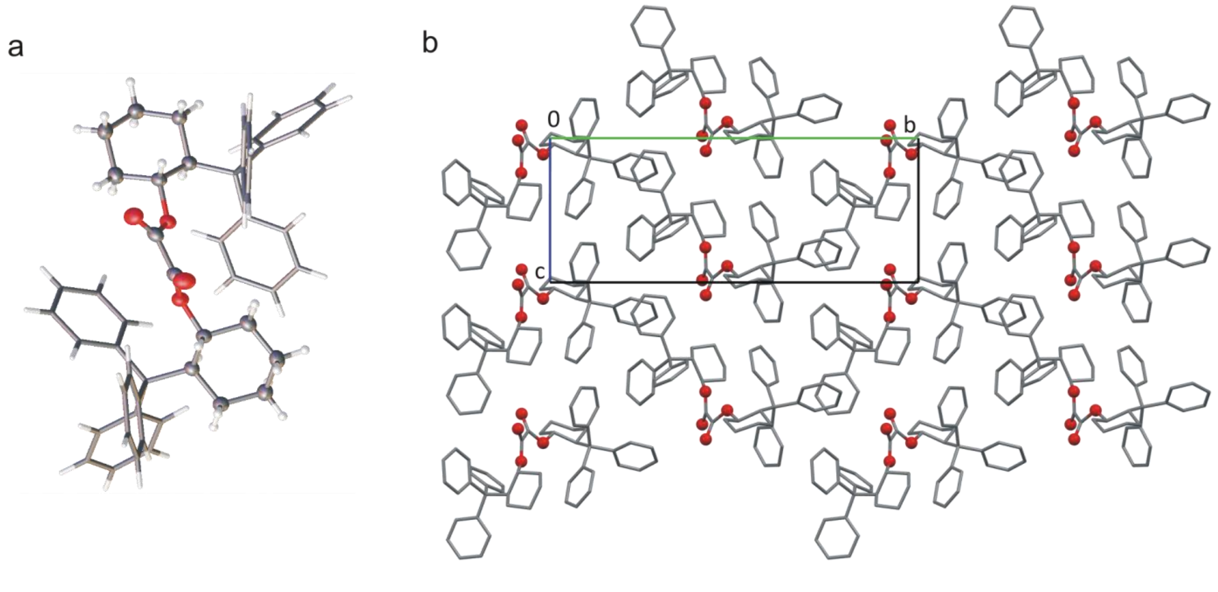

Figure S_100. a) Molecular structure of compound **9**; b) molecular packing in crystal structure (view along a-axis; The O atoms are shown as balls and hydrogen atoms are omitted for clarity).

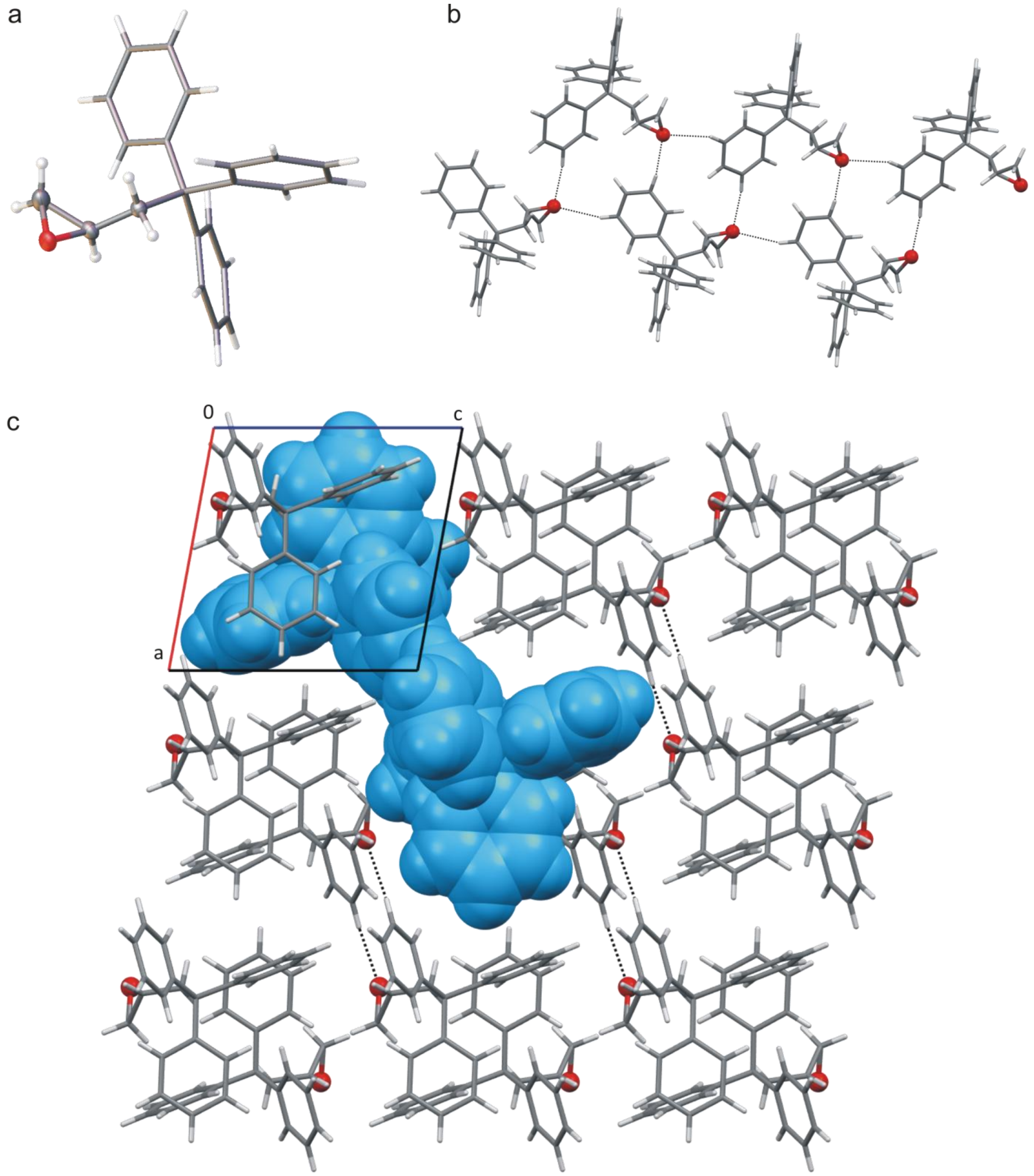

Figure S_101. a) Molecular structure of asymmetric unit of compound **11**, b) molecular ladder-like structural motif *via* hydrogen bonds (hydrogen bonds are shown as dashed lines, the O-atoms are shown as balls) and c) molecular packing in crystal structure (view along a-axis) – supramolecular ladder-like motif is indicated with blue colored spacefill model.

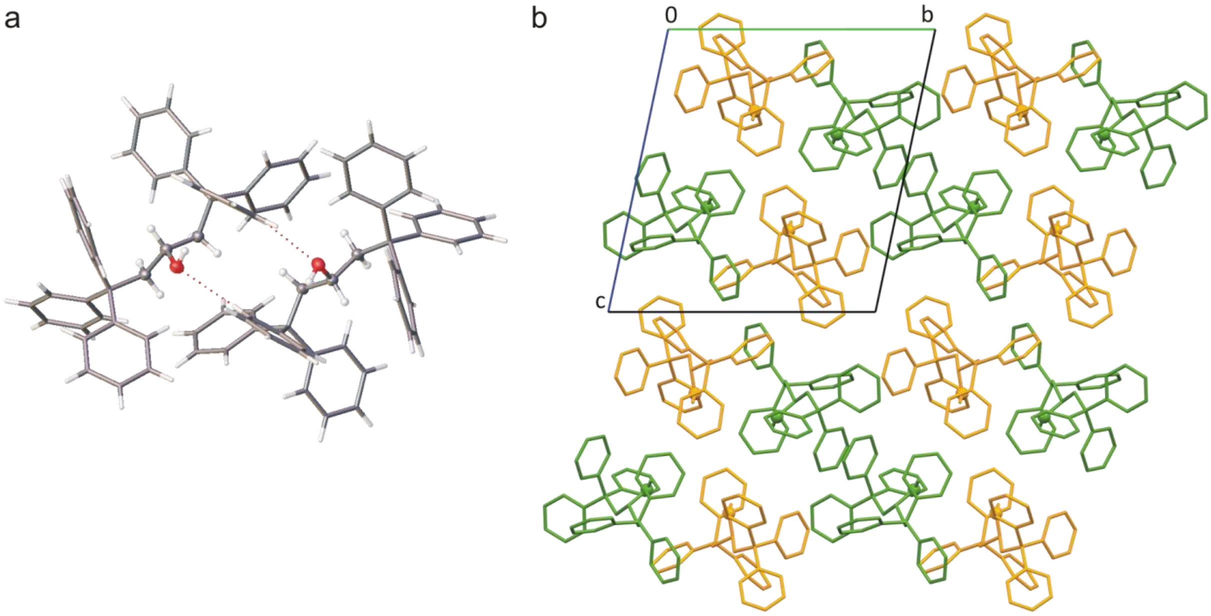

Figure S_102. a) Molecular structure of asymmetric unit of compound **12** (hydrogen bonds are shown as dashed lines), and b) molecular packing (view along a-axis) in crystal structure (symmetrically independent molecules are indicated with different colors and the O-atoms are shown as balls).

Table S_3. Selected crystal data and structure refinement details.

	rac-**3-α**	*rac*-**3-β**	*rac*-**4**	(*R*)-**4**	*rac*-**5**	*rac*-**7**	**8**	**9**	**11**	**12**
CCDC number	1977921	1977922	1977923	1977924	1977925	1977926	1977927	1977928	1977929	1977930
Chemical formula	$C_{25}H_{26}O$	$C_{25}H_{26}O$	$C_{25}H_{24}O$	$C_{25}H_{24}O$	$C_{25}H_{26}O$	$C_{27}H_{26}O_4 \cdot 0.5(CHCl_3)$	$C_{37}H_{44}O_4$	$C_{52}H_{50}O_4$	$C_{22}H_{20}O$	$C_{41}H_{36}O$
Mr	342.46	342.46	340.44	340.44	342.46	474.16	552.72	738.92	300.38	544.70
Crystal system, space group	Orthorhombic, *Pbca*	Monoclinic, $P2_1/n$	Trigonal, $R\bar{3}$	Trigonal, $P3_2$	Triclinic, $P\bar{1}$	Monoclinic, $P2_1/c$	Monoclinic, $P2_1$	Monoclinic, $P2_1$	Monoclinic, $P2_1$	Triclinic, $P\bar{1}$
Temperature (K)	130	130	130	130	130	130	130	130	130	130
a, *b*, *c* (Å)	17.2246 (3), 14.3249 (2), 30.5314 (7)	15.05174 (14), 14.22982 (15), 17.79674 (16)	30.9771 (5), 10.1399 (2)	9.7431 (1), 34.4543 (4)	10.4348 (4), 11.3879 (4), 16.7545 (7)	17.8007 (4), 8.42687 (16), 15.7496 (4)	12.5193 (1), 8.6892 (1), 14.0345 (1)	9.7156 (2), 23.0002 (4), 9.9771 (3)	9.16723 (9), 9.71283 (8), 9.22678 (8)	11.9181 (2), 15.5305 (3), 17.6263 (3)
α, β, γ (°)		95.9413 (8)			72.557 (3), 86.787 (3), 85.089 (3)	102.379 (2)	92.363 (1)	115.540 (3)	100.4883 (9)	99.9110 (16), 108.4018 (16), 94.3959 (15)
V ($Å^3$)	7533.3 (3)	3791.29 (6)	8426.5 (3)	2832.49 (7)	1891.45 (13)	2307.59 (9)	1525.41 (2)	2011.64 (9)	807.82 (1)	3019.42 (10)
Z	16	8	18	6	4	4	2	2	2	4
D_x (Mg m^{-3})	1.208	1.200	1.208	1.211	1.203	1.365	1.203	1.220	1.235	1.198
Radiation type	Cu *K*α	Cu *K*α	Cu *K*α	Cu *K*α	Cu *K*α	Cu *K*α	Cu *K*α	Cu *K*α	Cu *K*α	Cu *K*α
μ (mm^{-1})	0.55	0.54	0.55	0.54	0.54	2.27	0.60	0.59	0.57	0.53
Crystal size (mm)	0.3 × 0.2 × 0.08	0.5 × 0.3 × 0.2	0.25 × 0.02 × 0.02	0.2 × 0.1 × 0.02	0.2 × 0.15 × 0.1	0.25 × 0.15 × 0.05	0.25 × 0.2 × 0.1		0.25 × 0.15 × 0.05	0.30 × 0.15 × 0.05
No. of measured, independent and observed [$I > 2\sigma(I)$] reflections	45553, 7354, 6830	24128, 7200, 6501	17857, 3702, 3154	25175, 7166, 6765	13982, 7117, 6787	30115, 4064, 3730	20615, 580, 5620	14809, 7108, 6682	15519, 3313, 3264	75408, 11453, 10014
R_{int}	0.030	0.021	0.029	0.041	0.026	0.045	0.021	0.027	0.021	0.032
$R[F^2 > 2\sigma(F^2)]$, $wR(F^2)$, *S*	0.037, 0.096, 1.05	0.041, 0.106, 1.07	0.033, 0.089, 1.05	0.033, 0.084, 1.04	0.078, 0.189, 1.17	0.044, 0.124, 1.07	0.029, 0.080, 1.05	0.031, 0.073, 1.03	0.028, 0.071, 1.06	0.035, 0.090, 1.03
No. of parameters	492	599	235	576	605	342	404	506	208	765
$\Delta\rangle_{max}$, $\Delta\rangle_{min}$ (e $Å^{-3}$)	0.28, -0.17	0.22, -0.17	0.23, -0.15	0.15, -0.11	0.81, -0.30	0.41, -0.37	0.17, -0.13	0.27, -0.13	0.14, -0.17	0.22, -0.23
Absolute structure parameter	-	-	-	0.07 (17)	-	-	-0.05 (5)	0.06 (9)	0.04 (7)	-

Table S_4. Dihedral angles *α*, *β*, *γ* and *δ* (in degrees) and selected interatomic distances l_1 and l_2 (in Å) observed in the crystal structures **3**α, **3**β, **4a**, **4b**, **5**, **7**, **8**, **9**, **11** and **12**.

Compound	α^{b}	β_1^{c}	β_2^{c}	β_3^{c}	γ_1^{d}	γ_2^{d}	γ_3^{d}	δ^{e}	ζ^{f}	ϕ^{g}	l_1^{h}	l_2^{i}
**3**α (mol A)	49.6 (1)	62.8 (1)	-174.3 (1)	-62.1 (1)	-77.8 (14)	19.0 (2)	-45.0 (1)	175.5	-	-	2.788	2.656
**3**α (mol B)	53.6 (2)	59.0 (1)	-178.0 (1)	-65.7 (1)	-86.1 (1)	24.4 (2)	-37.5 (2)	-9.6	-	-	2.963	2.752
**3**β (mol A)	-48.7 (1)	-61.7 (5)	65.5 (1)	178.4 (1)	-87.4 (7)	34.2 (2)	-33.2 (1)	174.0	-	-	2.875	2.713
**3**β (mol A’)	38.6 (2)	66.1 (4)	-171.6 (1)	-58.8 (1)	-84.6 (6)			179.7	-	-	2.940	2.600
**3**β (mol B)	-51.2 (2)	-56.2 (5)	64.3 (1)	176.6 (1)	83.4 (7)	37.2 (1)	-25.9 (2)	2.2	-	-	2.973	2.635
**3**β (mol B’)	46.8 (2)	58.9 (5)	-173.9 (9)	-61.6 (1)	84.7 (6)			-73.2	-	-	2.963	2.640
(*rac*)-**4**	10.4 (2)	-81.4 (1)	43.0 (1)	156.8 (1)	76.7 (1)	37.0 (1)	-3.3 (1)	-	-	-	2.887	2.345
(*R*)-**4** (mol A)	0.8 (3)	70.7 (2)	-168.8 (2)	-54.9 (3)	85.1 (2)	22.8 (3)	-29.0 (3)	-	-	-	2.971	2.491
(*R*)-**4** (mol B)	3.5 (7)	75.9 (4)	-162.2 (3)	-49.5 (4)	-80.4 (6)	14.6 (3)	-39.1 (3)	-	-	-	2.882	2.387
(*R*)-**4** (mol B’)	7 (7)	70 (4)	-56 (4)	-166 (3)	-83 (3)	-45 (2)	19 (2)	-	-	-	2.961	2.385
**5** (mol A)	79.4 (4)	−59.3 (4)	60.9 (10)	178.1 (3)	−80.1 (5)	20.0 (19)	−31.6 (4)	-64.2	-	-	2.390	-
**5** (mol A’)	-83.4 (14)	69.4 (4)	−170.3 (9)	−53.2 (4)				41.8	-	-	2.166	-
**5** (mol C)	-75 (4)	71 (4)	−54 (4)	−168 (3)	86 (3)	−36 (4)	29 (2)	6.6	-	-	2.784	-
**5** (mol B)	79.5 (5)	−59.4 (5)	62.4 (7)	178.2 (4)	−73.0 (4)	18.2 (10)	−41.7 (4)	-78.9	-	-	2.458	-
**5** (mol B’)	-77 (3)	73.3 (6)	−164.9 (6)	−49.1 (6)				65.8	-	-	2.412	-
**5** (mol D)	-76 (3)	−48 (2)	−167 (2)	74.2 (19)	−22.5 (10)	42.1 (15)	73.2 (9)	53.3	-	-	2.085	-
**7**	60.2 (2)	72.4 (2)	-54.4 (2)	-167.1 (1)	-72.6 (2)	-56.8 (2)	5.7 (2)	-	-	-152.6 (2)	2.805	2.503

**8**	-54.1 (2)	-56.0 (2)	68.2 (2)	-178.1 (2)	-86.2 (2)	35.0 (2)	-31.4 (2)	-	29.6	127.8 (2)	2.887	2.721
**9**	-46.4 (2)	-42.5 (3)	81.5 (2)	-164.8 (2)	-72.5 (3)	10.1 (3)	-44.6 (3)	-	36.1	-32.1 (4)	3.010	2.643
	-60.2 (2)	-62.4 (2)	63.9 (2)	177.6 (2)	83.1 (3)	45.4 (3)	-14.5 (3)	-	34.8		2.838	2.874
**11**	-174.9 (1)	71.1 (2)	-168.6 (1)	-51.1 (2)	-69.7 (2)	-45.1 (2)	-27.6 (2)	-	-	-	-	-
**12** (mol A)	-90.5 (1)	54.5 (1)	173.8 (1)	-67.5 (1)	78.0 (1)	21.3 (1)	-11.0 (1)	-	-	-	-	-
	62.5 (1)	37.4 (1)	156.6 (1)	-86.3 (1)	69.6 (1)	40.4 (1)	-1.2 (2)	-	-	-	-	2.447
**12** (mol B)	-93.3 (1)	49.0 (1)	170.2 (1)	-73.1 (1)	75.1 (1)	38.0 (1)	-9.7 (2)	-	-	-	-	-
	57.7 (1)	30.6 (1)	149.5 (1)	-92.8 (1)	62.4 (1)	39.8 (1)	9.3 (1)	-	-	-	-	2.444

[a] – conformers are numbered according to their appearance during conformational search; [b] – α = O-C*-C-C_{ipso} or O-C*-C*-C(Ph_3); [c] – β = C-C*-C-C_{ipso}; [d] – γ = C-C_{Tr}-C_{ipso}-C_{ortho} (of the two possibilities the absolute values ≤90° has been chosen); [e] – δ = H-O-C*-C; [f] – ζ = H-C*-O-C or (O=)C-C(=O)-O-H [g] – ϕ = O=C-C=O; [h] – l_1 = (O)H···C_{ipso}; [h] – l_2 = C_{ortho}H···O-C or C_{ortho}H···O=C.

References

[1] A.M. Al Hazmi, N.S. Sheikh, C.J. Bataille, A.A. Al-Hadedi , S.V. Watkin, T.J. Luker, N.P. Camp, R.C. Brown, *Org Lett.* **2014**, *16,* 5104-5107.

[2] A. Zhang, L. Yang, N. Yang, Y. Liu, *Tetrahedron: Asymm.* **2014**, *25*, 289–297.

[3] SCIGRESS 2.5, Fujitsu Ltd

[4] a) A. D. Becke, *J. Chem. Phys.* **1993**, 98, 5648; b) C. Lee, W. Yang, R. G. Parr, *Phys. Rev. B*, **1988**, 37, 785; c) A. D. Becke, *Phys. Rev. A*, **1988**, 38, 3098; d) J. P. Perdew, *Phys. Rev. B*, **1986**, 33, 8822.

[5] M. J. Frish, G. W. Trucks, H. B. Schlegel, G. E. Scuseria, M. A. Robb, J. R. Cheeseman, G. Scalmani, V. Barone, B. Mennucci, G. A. Petersson, H. Nakatsuji, M. Caricato, H. Li, H. P. Hratchian, A. F. Izmaylov, J. Bloino, G. Zheng, J. L. Sonnenberg, M. Hada, M. Ehara, K. Toyota, R. Fukuda, J. Hasegawa, M. Ishida, T. Nakajima, Y. Honda, O. Kitao, H. Nakai, T. Vereven, J. A. J. Montgomery, J. E. Peralta, F. Ogliaro, M. Bearpark, J. J. Heyd, E. Brothers, K. N. Kudin, V. N. Staroverov, R. Kobayashi, J. Normand, K. Raghavachari, A. Rendell, J. C. Burant, S. S. Iyengar, J. Tomasi, M. Cossi, N. Rega, J. M. Millam, M. Klene, J. E. Knox, J. B. Cross, V. Bakken, C. Adamo, J. Jaramillo, R. Gomperts, R. E. Stratmann, O. Yazyev, A. J. Austin, R. Cammi, C. Pomelli, J. W. Ochterski, R. L. Martin, K. Morokuma, V. G. Zakrzewski, G. A. Voth, P. Salvadur, J. J. Dannenberg, S. Dapprich, A. D. Daniels, Ö. Farkas, J. B. Foresman, J. V. Ortiz, J. Cioslowski and D. J. Fox, Gaussian 09, Gaussian Inc., Wallingford, CT, **2009**.

[6] a) Y. Zhao, D. G. Truhlar, *Theor. Chem. Acc*. **2008**, 120, 215; b) D. Jacquemin, E. A. Perpète, I. Ciofini, C. Adamo, R. Valero, Y. Zhao, D. G. Truhlar, *J. Chem. Theor*. **2010**, 6, 2071.

[7] T. Yanai, D. Tew, N. Handy, *Chem. Phys. Lett*. **2004**, 393, 51.

[8] M. Kwit, M. D. Rozwadowska, J. Gawronski, A. Grajewska, *J. Org. Chem*. **2009**, 74, 8051 and references therein.

[9] N. Harada, P. Stephens, *Chirality*, **2010**, 22, 229.

[10] CrysAlis PRO 1.171.40.57a, Rigaku Oxford Diffraction, **2019**.

[11] G. M.Sheldrick, *Acta Crystallogr.* **2015**, *A71*, 3-8.

[12] G. M. Sheldrick, *Acta Crystallogr.* **2015**, *C71*, 3-8.

[13] S. Parsons, H. D. Flack, T. Wagner, *Acta Crystallogr*. **2013**, *B69*, 249-259.

[14] O. V. Dolomanov, L. J. Bourhis, R. J. Gildea, J. A. K. Howard, H. Puschmann, *J. Appl. Cryst.* **2009**, *42*, 339—341.

[15] C. F. Macrae, I. J. Bruno, J. A. Chisholm, P. R. Edgington, P. McCabe, E. Pidcock, L. Rodriguez-Monge, R. Taylor, J. van de Streek and P. A. Wood, *J. Appl. Cryst.* **2008**, 41, 466-470.
